# Machine learning in non-small cell lung cancer radiotherapy: A bibliometric analysis

**DOI:** 10.3389/fonc.2023.1082423

**Published:** 2023-03-17

**Authors:** Jiaming Zhang, Huijun Zhu, Jue Wang, Yulu Chen, Yihe Li, Xinyu Chen, Menghua Chen, Zhengwen Cai, Wenqi Liu

**Affiliations:** ^1^ Department of Radiation Oncology, The Second Affiliated Hospital of Guangxi Medical University, Nanning, China; ^2^ Department of Oncology, The Second Affiliated Hospital of Guangxi Medical University, Nanning, China

**Keywords:** non-small cell lung cancer, radiotherapy, machine learning, computer science, bibliometric analysis

## Abstract

**Background:**

Machine learning is now well-developed in non-small cell lung cancer (NSCLC) radiotherapy. But the research trend and hotspots are still unclear. To investigate the progress in machine learning in radiotherapy NSCLC, we performed a bibliometric analysis of associated research and discuss the current research hotspots and potential hot areas in the future.

**Methods:**

The involved researches were obtained from the Web of Science Core Collection database (WoSCC). We used R-studio software, the Bibliometrix package and VOSviewer (Version 1.6.18) software to perform bibliometric analysis.

**Results:**

We found 197 publications about machine learning in radiotherapy for NSCLC in the WoSCC, and the journal Medical Physics contributed the most articles. The University of Texas MD Anderson Cancer Center was the most frequent publishing institution, and the United States contributed most of the publications. In our bibliometric analysis, “radiomics” was the most frequent keyword, and we found that machine learning is mainly applied to analyze medical images in the radiotherapy of NSCLC.

**Conclusion:**

The research we identified about machine learning in NSCLC radiotherapy was mainly related to the radiotherapy planning of NSCLC and the prediction of treatment effects and adverse events in NSCLC patients who were under radiotherapy. Our research has added new insights into machine learning in NSCLC radiotherapy and could help researchers better identify hot research areas in the future.

## 1 Introduction

Lung cancer is one of the most common malignant tumors (1, 2). According to *Cancer Statistics, 2020*, the 5-year survival rate of lung cancer is only 19% (1). Non-small cell lung cancer (NSCLC) is defined as a subtype of lung cancer that accounts for over 80% of lung cancer cases (3). Although there are many treatments for NSCLC, the long-term survival rate of advanced NSCLC patients is still poor (4, 5).

Radiotherapy is part of the treatment of almost all stages of NSCLC. Approximately 30% of NSCLC patients are diagnosed with unresectable stage III cancer, also called locally advanced NSCLC (6). The standard treatment for unresectable stage III NSCLC patients is concurrent chemoradiotherapy according to the NCCN guidelines (7, 8). Stereotactic body radiotherapy (SBRT) is widely used in the treatment of patients with early-stage unresectable NSCLC. In addition, palliative radiotherapy is also recommended in patients with advanced or progressive NSCLC. However, problems such as target delineation, dose and fractionated irradiation mode still trouble clinicians (9–12). Manual contouring of target volumes and organs at risk (OARs) is an important process for radiotherapy planning. These tasks may be repetitive and time-consuming. Furthermore, some questions, such as which kind of patients can benefit more from radiotherapy and when is the optimal time to receive radiotherapy, are still in dispute (13). Therefore, it is necessary to reduce the burden of clinicians and improve the efficacy of radiotherapy.

Researchers are now exploring the use of machine learning in radiotherapy (14, 15). Machine learning is a multidisciplinary interdisciplinary field that involves statistics, convex analysis, probability theory, approximation theory, and algorithm complexity theory. Today, hundreds of articles on machine learning in NSCLC radiotherapy have been published. These articles span research areas such as radiotherapy planning, prognosis prediction, adverse event prediction and clinical decisions. Keeping an eye on the most influential articles and the research hotspots is important to researchers. Therefore, it is necessary to comprehensively analyze and summarize the current research trends and hotspots of machine learning in radiotherapy for NSCLC.

Bibliometric analysis aims to analyze the annual volume of articles and journals, countries, and regions using the database of citation reports. In addition, it can also expose hot topics and potential research directions of annual research so that researchers can better understand the current research trends and hot spots in the research field. We performed a bibliometric analysis on machine learning in radiotherapy for NSCLC. We highlighted the research progress and research trend of machine learning in radiotherapy for NSCLC. We comprehensively analyzed relevant publications and provide references for further research on machine learning in radiotherapy for NSCLC.

## 2 Method

### 2.1 Database and systematic search strategy

**Figure 1:**
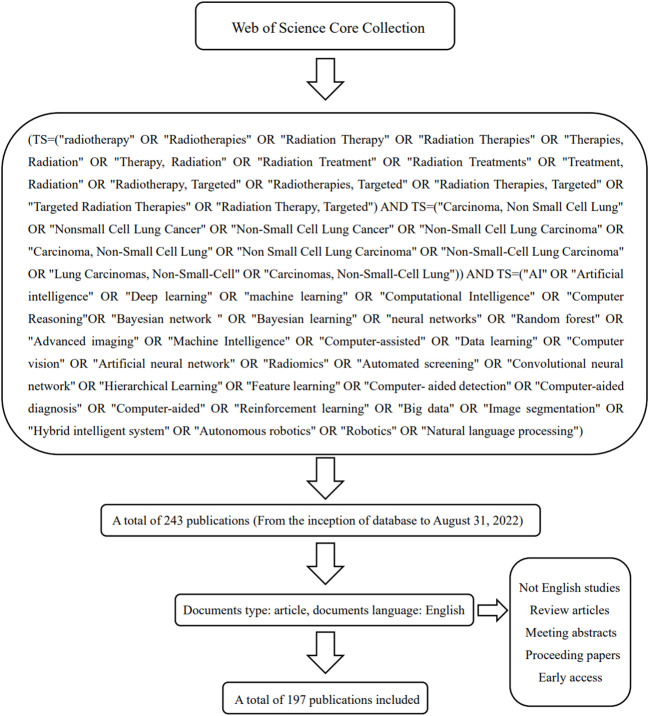
The search strategies of our bibliometric analysis, TS: Topic, which includes title, abstract, author keywords and additional keywords.

We obtained citation reports using the core collection of the Web of Science database (WoSCC). We searched all articles on machine learning in radiation therapy for NSCLC. The time limit for the included studies was from the inception of WoSCC to August 31, 2022. In our process of study screening, the only type of publication we included was article. We excluded review articles, meeting abstracts, proceeding papers and early access. The search strategies and inclusion criteria are listed in **Figure 1**.

### 2.2 Statistical analysis

We used R-studio software, the Bibliometrix package and VOSviewer (Version 1.6.18) software to analyze the collected data and Biblioshiny to visualize the data. The Bibliometrix package is a kind of workflow for performing bibliometric analysis and was programmed by R (16). Then, we analyzed the number of publications, citations and collaborations, annual publications and author publications in a country/region. We also analyzed the quality of the author’s academic production using the H-index. It was defined that if the scientist has index h, there are at least h citations (N_P_ – h) between the papers whose index is hN_p_, and each paper has ≤ h citations (17). Then, we analyzed the hotspots of machine learning in NSCLC radiotherapy and the annual trend topics. We constructed the keyword co-occurrence network using VOSviewer. Different clusters in a network diagram are represented by different colors, and collaborations, cooccurrence, are represented by connectors. The thickness of the connecting line indicates the strength of the association. The size of the circle indicates the number of publications, references, or keywords.

## 3 Results

### 3.1 Annual publication analysis

**Figure 2:**
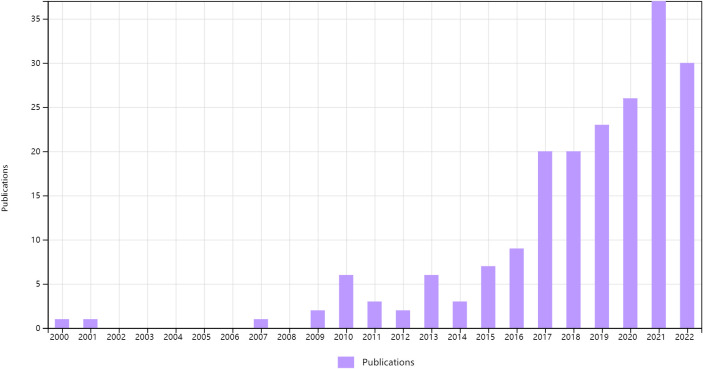
Annual publications about machine learning in NSCLC radiotherapy.

We found 197 publications about machine learning in radiotherapy for NSCLC in the WoS core database, and the data generated by the WoS database are listed in the **Supplementary material**. We obtained all the publications we found for bibliometric analysis. The overall trend of annual publications is shown in **Figure 2**. These results indicate that machine learning in radiotherapy is still an important frontier field in the treatment of NSCLC patients.

### 3.2 Bibliometric analysis of top journals

**Figure 3:**
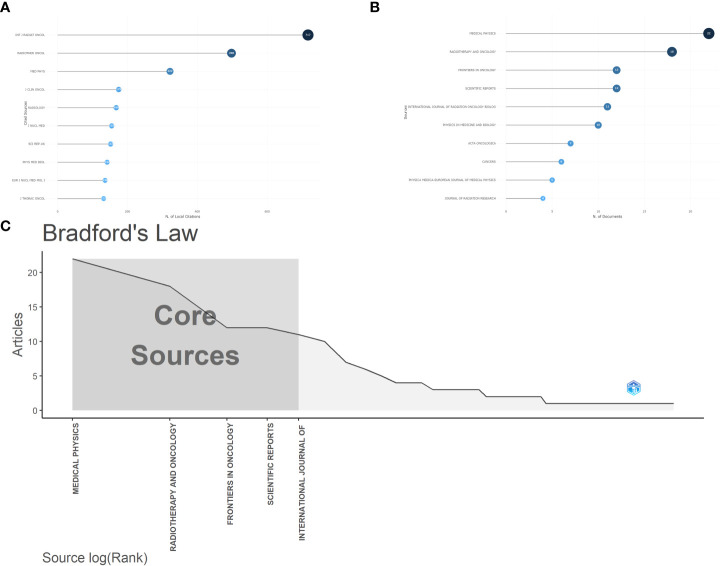
**(A)** The top 10 most locally cited journals in the bibliometric analysis. **(B)** The top 10 most published journals in bibliometric analysis. **(C)** The core sources of bibliometric analysis according to Bradford’s law.

The articles included in the bibliometric analysis were from a total of 72 journals. In our analysis, the journal *Medical Physics* contributed the most articles at 22 and accounting for 11% of the total articles. According to Bradford’s Law, most of the articles are from *Medical Physics*, *Radiotherapy and Oncology, Frontiers in Oncology, Scientific Reports* and *International Journal of Radiation Oncology*. The most frequently cited journal is *International Journal of Radiation Oncology*, with a total of 717 local citations, followed by *Radiother Oncol* and *Medical Physics*, with a total of 12 journals cited more than 100 times. The results are shown in **Figure 3**.

### 3.3 Bibliometric analysis of authors, institutions and countries/regions

**Figure 4:**
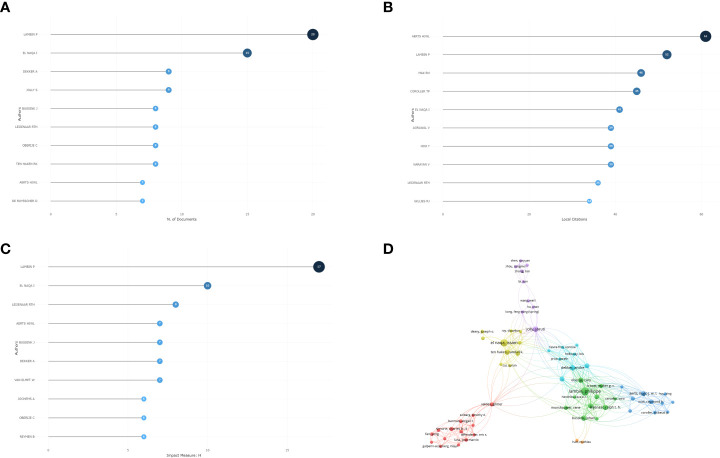
**(A)** The top 10 most published authors in the bibliometric analysis. **(B)** The top 10 most local cited authors in bibliometric analysis. **(C)** The H-index of authors in bibliometric analysis. **(D)** The co-authorship in bibliometric analysis.

The included articles are from 1350 different authors. The author with the most published articles is Lambin P from the Maastricht University Medical Center, who has participated in the publication of 20 included articles, followed by El Naqa I and Dekker A. The author with the most local citations is Aerts HJWL, who with a total of 61 local citations. According to the author’s influence calculated by the H-index, Lambin P has the highest influence, followed by El Naqa I and Leijennar Rth. We also analyzed the coauthor relationship and constructed the coauthor network. The results are shown in **Figure 4**.

**Figure 5:**
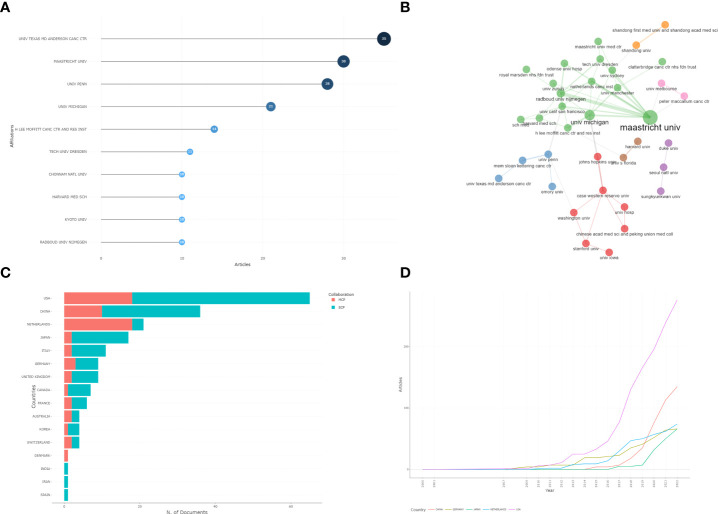
**(A)** The most published institution in bibliometric analysis. **(B)** The cooperative relationship of institutions in bibliometric analysis. **(C)** Country publications. MCP: Multicenter publishing, SCP: Single-center publishing. **(D)** The annual publication by country.

The included articles were from a total of 380 institutions. Eleven institutions had more than 10 publications. The University of Texas MD Anderson Cancer Center was the most frequent publishing institution, contributing 35 articles. Then, we analyzed the cooperative relationship among institutions. Bibliometric analysis for national/regional published research is also essential. The articles included are from 16 different countries or regions. The United States has published the most articles, with 65 articles in total, followed by China and Netherlands. The results are shown in **Figure 5**.

### 3.4 Bibliometric analysis of citations and references

**Figure 6:**
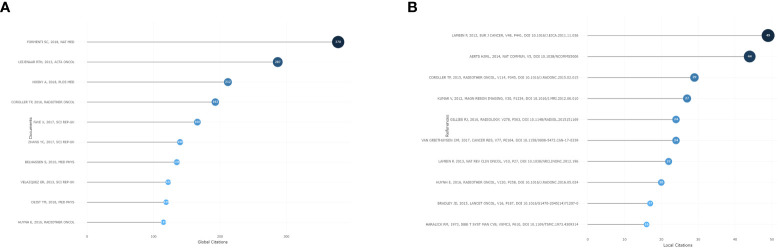
**(A)** The most globally cited articles in bibliometric analysis. **(B)** The most locally cited references in bibliometric analysis.

Citation analysis revealed that a total of 13 articles were cited more than 100 times. The article by Silvia C Formenti published in 2018, *Radiotherapy induces responses of lung cancer to CTLA-4 blockade*, has the most global citations. Then, we analyzed the references of the included publications. We found that the most cited reference was *Radiomics: extracting more information from medical images using advanced feature analysis* from Philippe Lambin, which has been cited 49 times. The results are shown in **Figure 6**.

### 3.5 Bibliometric analysis of keywords

**Figure 7:**
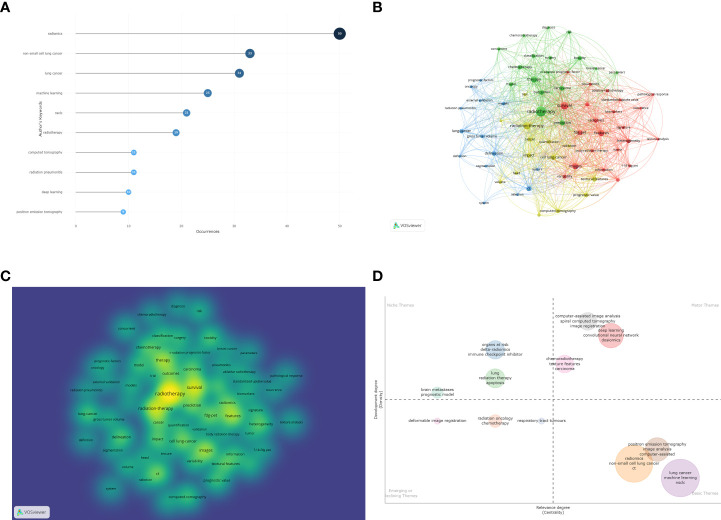
**(A)** The most frequent keywords in bibliometric analysis. **(B)** Keyword co-occurrence analysis in bibliometric analysis. **(C)** Density map of keyword analysis. **(D)** Thematic map through keyword analysis.

To analyze the current research hotspots and trends, the keywords were analyzed. We used Vosviewer and Bibliometrix to analyze the co-occurrence keywords included in the dataset. There were 434 author keywords in total, of which 9 appeared more than 10 times. The top three keywords were “radiomics”, “non-small cell lung cancer” and “lung cancer”. Then, we used Vosviewer to visualize the co-occurrence keyword network. The results are shown in **Figure 7**, and the keywords are listed in **Table 1**.

**Table 1:** The top 10 most frequent keywords in bibliometric analysis.

Words	Occurrences
radiomics	50
non-small cell lung cancer	33
lung cancer	31
machine learning	25
NSCLC	21
radiotherapy	19
computed tomography	11
radiation pneumonitis	11
deep learning	10
positron emission tomography	9

We used the Bibliomtrix package to construct a thematic map through the bibliometric analysis of keywords. The thematic map was divided into 4 quadrants. The first quadrant is the motor themes, which represent important and well-developed topics. The second quadrant is the highly developed and isolated themes, which represent well-developed but not very important recent topics. The third quadrant is the emerging or disappearing themes, which represent those topics that may have just emerged or may soon disappear. The fourth quadrant is basic themes, which represent those topics that are very important to the field but have not been well developed. The bubble size depends on the occurrence frequency of cluster keywords. The bubble location depends on the development degree and relevance degree of the clusters (16, 18). In our research, there are three clusters located in the first quadrant, and the keywords “deep learning” and “convolutional neural network” and “dosiomics” and “computer-assisted image analysis” could be considered the current research frontiers. “Radiomics” and “machine learning” may be the basis of current research on machine learning in NSCLC radiotherapy. The results are shown in **Figure 7**.

## 4 Discussion

Radiotherapy is one of the main treatment methods for locally advanced or advanced NSCLC, and it still has rich research value at present (19–22). Machine learning is also one of the research hot fields in the radiotherapy of NSCLC. Although a large number of relevant studies on machine learning in radiotherapy for NSCLC emerge every year, there is still a lack of corresponding bibliometric analysis to summarize the research trends and current hotspots of machine learning in radiotherapy for NSCLC. In our study, we used the Bibliometrix package and Vosviewer software to perform a bibliometric analysis of machine learning in NSCLC radiotherapy.

The studies on machine learning in NSCLC radiotherapy that we included in the bibliometric analysis were mainly about several subjects as discussed below.

### 4.1 Machine learning in NSCLC radiotherapy planning

Radiotherapy planning is one of the most important steps in NSCLC radiotherapy. In the research that we analyzed, machine learning mainly participated in radiotherapy planning, dosimetric assessment, delineation of tumor volumes and OARs of NSCLC radiotherapy. Volumetric modulated arc therapy (VMAT) is one of the treatments for locally advanced NSCLC. Giuseppe Della Gala’s team developed an autoVMAT treatment planning system for NSCLC using data from a previous patient database. The autoVMAT planning system showed statistically significant improvements in planning target volume (PTV) coverage, with an increase in V95% of 1.1% ± 1.1%, and higher dose conformity, with a reduction in R50 of 12.2% ± 12.7%, when compared to manually created intensity-modulated radiation therapy (IMRT) plans. Additionally, the autoVMAT planning system also reduced the mean doses of organs at risk (OARs) including lung, heart, and esophagus with reductions of 0.9 Gy ± 1.0 Gy, 1.5 Gy ± 1.8 Gy, and 3.6 Gy ± 2.8 Gy, respectively (p < 0.001) (23). In dosimetric assessment, automated dose adaptation based on deep reinforcement learning was also a potential research hotspot. Huan‐Hsin Tseng have constructed an automated radiation adaptation protocols based on multicomponent reinforcement learning for deep learning. Then, they chose the optimal dose generated by the trained model. Their results considered that automated dose adaptation could achieve similar results to those chosen by clinical doctors(the estimated root‐mean‐square error (RMSE)≈0.76 Gy when compared to the clinical data) (24). Besides, delineation of tumor volumes and organs at risk such as esophagus, heart and aorta on medical images is important but time-consuming. A recent study compared the performance between automatic segmentation of OARs based on a convolutional neural network (AS-CNN) and atlas (AS-Atlas). The results showed that both AS-CNN and AS-Atlas could reduce substantial time for OAR segmentations when compared to manual delineation (the average time of AS-CNN and AS-Atlas was 1.6 minutes and 2.4 minutes when manual delineation costed 25.4 minutes). And the accuracy of auto segmentation of OARs was similar to manual delineation (25). Furthermore, the analysis of single-modality medical images may lack some important information, as they only partially reflect the tumor’s characteristics and may reduce the accuracy of tumor volume and OARs delineation. Therefore, recent research has focused on multimodal medical image analysis to improve the accuracy and effectiveness of tumor segmentation. Jue Jiangs presented a cross-modality model based on data from CT and MRI images for deep learning lung tumor segmentation. They transformed the CT images to MR images resembling T2w MRI. And then they performed the auto segmentation through the deep learning model base on the data from CT scanning and expert-segmented T2w MR. The tumor segmentation generated by this model was proven to be highly similar to expert segmentation (Dice similarity coefficient (DSC)= 0.75 ± 0.12) (26).

### 4.2 Machine learning in NSCLC radiotherapy efficacy evaluation

Although radiotherapy is an important treatment for unresectable NSCLC, the response to radiation within different NSCLC patients exhibits wide heterogeneity. Some NSCLC patients have long-term control after receiving radiotherapy, while others relapse or metastasize despite receiving a high dose of radiation (27). Thus, it is important to evaluate the efficacy of radiotherapy for NSCLC patients, including short-term efficacy and long-term efficacy. For short-term efficacy, Lameck Mbangula Amugongo has developed an automatic method through cone beam computed tomography (CBCT) images. And they constructed 4 models (linear, Gaussian, quadratic and cubic methods) that could predict the tumor volume and shape in weeks 3 and 4 of radiotherapy. And results showed that the linear model performed best at predicting tumor volume changes with a sensitivity of 84% and specificity of 99% (28). Besides, researchers also presented a prediction model based on radiomic phenotype features. This model could predict the tumor pathological response after neoadjuvant chemoradiation in NSCLC patients, particularly in pathologic gross residual disease and pathologic complete response (29). For long-term efficacy, a recent multicentric study developed a radiomics predictive model that could predict local relapse in NSCLC patients treated with SBRT. The model was based on radiomics from the PET/CT features, and the results of the study revealed that the area under curve (AUC) of model combining 2 PET feature has reached 0.94, and the sensitivity=100%; specificity=88% (30). In addition, to improve the performance of the prediction model, researchers presented a model based on multiple factors, which included CT imaging and clinical and hematological features. The results showed that the multifactor prediction model performed better than radiomic, clinical, or hematological models alone for survival prediction of patients with locally advanced NSCLC (31). However, many studies have investigated the prognostic factors from medical images by identifying the optimum value or a cutoff value from the continuous values of the image-derived index, and this method is called the ‘optimum cutoff approach’. Then, researchers divided the patients into a high-risk group or a low-risk group based on the cutoff value. However, a systematic review revealed that the use of the optimum cutoff approach may lead to type-I errors. In addition, this review also reported that the texture features extracted from PET and CT images represented a positive patient prognosis in some studies but a negative prognosis in other studies. Thus, researchers considered that the conclusions conflicted and evidence was insufficient to support a relationship between patient prognosis and features extracted from CT and PET (32).

### 4.3 Machine learning in NSCLC radiotherapy adverse event prediction

Prediction of radiation-induced adverse events through machine learning is also one of the hotspots of current research. Radiation pneumonitis is one of the most common and important adverse events of patients who receive radiotherapy, with an incidence of 29% in some studies (33). José Marcio Luna reported that lung V20 (the percentage of the total lung volume that receives a radiation dose of 20 Gy or higher)> 27.4%, lung mean dose >15.4 Gy, lung V10 >36.3% and lung V5 >43.6% were the predictor of radiation pneumonitis in univariate analysis. And their multivariate analysis based on random forest method also revealed that esophagus max, lung V20, lung mean and pack-year as the predictor of radiation pneumonitis (AUC=0.66) (34). In addition, radiation-induced esophagitis was also frequent in patients who receive chest radiation. A recently published study compared the predictive performance of different modeling methods in radiation-induced esophagitis. The researcher reported that the least absolute shrinkage and selection operator (LASSO-MLR) performed best in radiation-induced esophagitis prediction (AUC=0.79) (35). Furthermore, differentiation between radiation pneumonitis and immune checkpoint inhibitor (ICI)-induced pneumonitis is also very important when radiotherapy is combined with ICI treatment. Jun Cheng constructed a model that could identify patients with radiation pneumonitis or ICI-caused pneumonitis based on radiological characteristics using machine learning, and achieving an AUC of 0.896 (36). The adverse events of tumor radiotherapy are closely related to the radiation dose. It was reported that lung V5, V20, and V30 were closely associated with the incidence of radiation pneumonitis in chest radiotherapy (37, 38). To enhance the accuracy of predicting radiation pneumonitis, researchers are investigating the use of dosiomics, a novel concept emerged in recent years. Dosiomics can extract the dose distribution characteristics in the radiotherapy plan (39). And a recent study predicted the incidence of radiation pneumonitis through dosiomics. Researchers constructed the model based on dosiomics for predicting the incidence of radiation pneumonitis. They reported that the AUC of dosiomics prediction model based on multivariate logistic regression was 0.782 and performed better than the dosimetric factors(mean dose of ipsilateral lung and contralateral lung V5, AUC=0.676) (40).

### 4.4 The limitation of included studies

Although machine learning has been widely reported to be involved in NSCLC radiotherapy planning, efficacy prediction, and adverse reaction assessment in clinical practice, there are still some limitations in current studies. Firstly, most of the included studies were based on data analysis from single center, with small sample sizes and a lack of external validation. These issues may lead to a decrease in the efficacy of the predictive model, make it unsuitable for different populations, and result in false research findings (41). Secondly, many of the studies we included were retrospective studies and lack of prospective studies. The data they used to train and validate the prediction models came from patients who had already received treatment and had known outcomes. That may increase the risk of reporting biased. Thirdly, the radiomics prediction models rely on quantitative imaging features extracted from medical images (42, 43). Which can be influenced by scanning devices, different acquisition modes and reconstruction parameters (32). The limitation hinders the development of universal imaging radiomics models. While there have been numerous studies on the application of machine learning in NSCLC radiotherapy, the current research still has certain limitations in terms of depth or effectiveness. To address these limitations, the following potential research hotspots should be considered.

### 4.5 Future hotspots of machine learning in NSCLC radiotherapy

Our research was based on 197 articles published from 2000 to 2022. According to recently published articles and the discussion among the authors, the future hotspots of machine learning in NSCLC radiotherapy are as follows:

1) Improve the accuracy of data analysis and model construction and reduce misdiagnoses or missed diagnoses. There is a common denominator in most machine learning models: the more data used to train the model, the better it will perform (43). Therefore, machine learning models based on multicenter data may be a future research focus of NSCLC radiotherapy.
2) Machine learning-based clinical decision support system for individualized treatment. In the precision medicine era, precision and individualized radiotherapy is more suitable for patients. In the past, clinical decisions were made by clinical experts. Recently, clinical decision support systems (CDSSs) were considered that could lead to the development of precision and individualized radiotherapy (44, 45). This kind of system will provide suggestions to clinicians and help them make decisions on and plan patients’ treatment.
3) Multiple clinical factors prediction model. Prediction models based on radiomics occupied most of our included studies. However, a multiple clinical factors prediction model was reported to be better than radiomics alone in outcome prediction (31). In the era of precision treatment, clinicians may provide individualized treatment based on the predictive outcomes of patients. Therefore, it may be disastrous to incorrectly predict the outcomes of patients with NSCLC. Thus, in the future, prediction models based on multiple clinical factors will be a research hotspot.

## 5 Limitations of our analysis

1) Due to the limitations of bibliometric analysis software and visualization software, it is difficult to merge and analyze data from different databases (such as PubMed, Embase, Cochrane Library), which limits the comprehensiveness of the analysis.
2) We only included English articles, which will lead to a selective bias.
3) The included articles were published from database inception to August 31, 2022. However, the database is still being updated, and we failed to include most recent publications. This may affect the results of the analysis.

## 6 Conclusion

Radiotherapy for NSCLC is widely used, and machine learning is widely used in NSCLC radiotherapy. However, compared with the number of studies on radiotherapy for NSCLC, the number of studies related to machine learning in radiotherapy for NSCLC is still small. Further studies in these areas are needed to benefit patients. Therefore, it is necessary to analyze the relevant studies and determine the possible research directions and hotspots. In conclusion, our bibliometric analysis analyzed the research trends, hotspots and other information. This could help researchers to further study the fields of machine learning in NSCLC radiotherapy.

## Data availability statement

The original contributions presented in the study are included in the article/**Supplementary Material**. Further inquiries can be directed to the corresponding authors.

## Author contributions

WL and ZC contributed to the conception of the study, and supervision. YL and XC contributed to the collection of data. YC and MC contributed to statistics of research data and the arrangement of research pictures. JZ and HZ performed the bibliometric analysis and wrote the manuscript, should be considered as the co-first author. HZ and JW helped to perform the analysis with constructive discussions and editing. All authors contributed to the article and approved the submitted version.
