# Corrigendum: Machine learning in non-small cell lung cancer radiotherapy: a bibliometric analysis

**DOI:** 10.3389/fonc.2023.1197217

**Published:** 2023-04-18

**Authors:** Jiaming Zhang, Huijun Zhu, Jue Wang, Yulu Chen, Yihe Li, Xinyu Chen, Menghua Chen, Zhengwen Cai, Wenqi Liu

**Affiliations:** ^1^ Department of Radiation Oncology, The Second Affiliated Hospital of Guangxi Medical University, Nanning, China; ^2^ Department of Oncology, The Second Affiliated Hospital of Guangxi Medical University, Nanning, China

**Keywords:** non-small cell lung cancer, radiotherapy, machine learning, computer science, bibliometric analysis

In the published article, there was an error in the Funding statement. **The funding from Guangxi key research and development program was omitted.** The correct Funding statement appears below:

**The authors declare that this study received funding from Guangxi key research and development program No. (GK) AB18221080, The Basic Ability Enhancement Program for Young and Middle-aged Teachers of Guangxi (2020KY03036), Youth Foundation of Guangxi Medical University(GXMUYSF201918), Foundation of Guangxi Health and Family Planning Commission (Z20190581), Foundation of the Second Affiliate Hospital of Guangxi Medical University (hbrc202104). The funders were not involved in the study design, collection, analysis, interpretation of data, the writing of this article or the decision to submit it for publication.**

The authors apologize for this error and state that this does not change the scientific conclusions of the article in any way. The original article has been updated.

