# Supplementary material for: Machine learning in non-small cell lung cancer radiotherapy: A bibliometric analysis
Source: Front Oncol. 2023 Mar 17;13:1082423. doi: 10.3389/fonc.2023.1082423 (PMC10072228; doi:10.3389/fonc.2023.1082423)
Supplement: Supplementary file 1 [file Table_1.docx]

FN Clarivate Analytics Web of Science

VR 1.0

PT J

AU Grgic, A

Nestle, U

Schaefer-Schuler, A

Kremp, S

Kirsch, CM

Hellwig, D

AF Grgic, Aleksandar

Nestle, Ursula

Schaefer-Schuler, Andrea

Kremp, Stephanie

Kirsch, Carl-Martin

Hellwig, Dirk

TI FDG-PET-BASED RADIOTHERAPY PLANNING IN LUNG CANCER: OPTIMUM BREATHING

PROTOCOL AND PATIENT POSITIONING-AN INTRAINDIVIDUAL COMPARISON

SO INTERNATIONAL JOURNAL OF RADIATION ONCOLOGY BIOLOGY PHYSICS

LA English

DT Article

DE Non-small-cell lung carcinoma (NSCLC); Spiral computed tomography;

Positron emission tomography; Computer-assisted radiotherapy planning;

Computer-assisted image analysis

ID POSITRON-EMISSION-TOMOGRAPHY; TARGET VOLUME DEFINITION;

COMPUTED-TOMOGRAPHY; CO-REGISTRATION; CT; DELINEATION; IMPACT; MOTION

AB Purpose: Fluoro-2-deoxy-D-glucose (FDG)-positron emission tomography (PET) and PET/computed tomography (CT) are increasingly used for radiotherapy (RT) planning in patients with non-small-cell lung carcinoma. The planning process often is based on separately acquired FDG-PET/CT and planning CT scans. We compared intraindividual differences between PET acquired in diagnostic (D-PET) and RT treatment position (RT-PET) coregistered with planning CTs acquired using different breathing protocols.

Methods and Materials: Sixteen patients with non-small-cell lung carcinoma underwent two PET acquisitions (DPET and RT-PET) and three planning CT acquisitions (expiration [EXP], inspiration [INS], and mid-breath hold [MID]) on the same day. All scans were rigidly coregistered, resulting in six fused data sets: D-INS, D-EXP, D-MID, RT-INS, RT-EXP, and RT-MID. Fusion accuracy was assessed by three readers at eight anatomic landmarks, lung apices, aortic arch, heart, spine, sternum, carina, diaphragm, and tumor, by using an alignment score ranging from 1 (no alignment) to 5 (exact alignment).

Results: The RT-PET showed better alignment with any CT than D-PET (p < 0.001). With regard to breathing, RT-MID showed the best mean alignment score (3.7 +/- 1.0), followed by RT-EXP (3.5 +/- 0.9) and RT-INS (3.0 +/- 11.8), with all differences significant (p < 0.001). Comparing alignment scores with regard to anatomic landmarks, the largest deviations were found at the diaphragm, heart, and apices. Overall, there was fair agreement (K = 0.48; p < 0.001) among the three readers.

Conclusions: Significantly better fusion of PET and planning CT can be reached with PET acquired in the RT position. The best intraindividual fusion results are obtained with the planning CT performed during mid-breath hold. Our data justify the acquisition of a separate planning PET in RT treatment position if only a diagnostic PET scan is available. (C) 2009 Elsevier Inc.

C1 [Grgic, Aleksandar; Nestle, Ursula; Schaefer-Schuler, Andrea; Kirsch, Carl-Martin; Hellwig, Dirk] Univ Saarland, Med Ctr, Dept Nucl Med, D-66421 Homburg, Germany.

RP Grgic, A (通讯作者)，Univ Saarland, Med Ctr, Dept Nucl Med, Geb 50, D-66421 Homburg, Germany.

EM aleksandar.grgic@gmx.de

RI Hellwig, Dirk/O-8617-2019; Nestle, Ursula/ABG-2339-2021; Hellwig,

Dirk/A-4128-2008

OI Hellwig, Dirk/0000-0002-3056-0143;

CR Aquino SL, 2003, J COMPUT ASSIST TOMO, V27, P479, DOI 10.1097/00004728-200307000-00004

Beyer T, 2000, J NUCL MED, V41, P1369

Beyer T, 2003, EUR J NUCL MED MOL I, V30, P588, DOI 10.1007/s00259-002-1097-6

Bradley J, 2004, INT J RADIAT ONCOL, V59, P78, DOI 10.1016/j.ijrobp.2003.10.044

De Ruysscher D, 2005, INT J RADIAT ONCOL, V62, P988, DOI 10.1016/j.ijrobp.2004.12.019

Dwamena BA, 1999, RADIOLOGY, V213, P530, DOI 10.1148/radiology.213.2.r99nv46530

Fitton I, 2008, INT J RADIAT ONCOL, V70, P1403, DOI 10.1016/j.ijrobp.2007.08.063

Fitton I, 2007, RADIOTHER ONCOL, V83, P42, DOI 10.1016/j.radonc.2007.02.010

Fleiss J. L, 1981, STAT METHODS RATES P

Gilman MD, 2006, AM J ROENTGENOL, V187, P1357, DOI 10.2214/AJR.05.1427

Goerres GW, 2002, EUR J NUCL MED MOL I, V29, P351, DOI 10.1007/s00259-001-0710-4

Gould MK, 2003, ANN INTERN MED, V139, P879, DOI 10.7326/0003-4819-139-11-200311180-00013

Gould MK, 2003, ANN INTERN MED, V138, P724, DOI 10.7326/0003-4819-138-9-200305060-00009

Grosu AL, 2005, STRAHLENTHER ONKOL, V181, P483, DOI 10.1007/s00066-005-1422-7

Hellwig D, 2001, Pneumologie, V55, P367, DOI 10.1055/s-2001-16201

Jarritt PH, 2006, BRIT J RADIOL, V79, pS27, DOI 10.1259/bjr/35628509

Krishnasetty V, 2005, RADIOLOGY, V237, P635, DOI 10.1148/radiol.2372041719

Lardinois D, 2003, NEW ENGL J MED, V348, P2500, DOI 10.1056/NEJMoa022136

Nestle U, 2005, J NUCL MED, V46, P1342

Nestle U, 2007, EUR J NUCL MED MOL I, V34, P453, DOI 10.1007/s00259-006-0252-x

Nestle U, 2006, RADIOTHER ONCOL, V81, P209, DOI 10.1016/j.radonc.2006.09.011

Shekhar R, 2005, J NUCL MED, V46, P1488

Sonke JJ, 2008, INT J RADIAT ONCOL, V70, P590, DOI 10.1016/j.ijrobp.2007.08.067

Underberg RWM, 2006, RADIAT ONCOL, V1, DOI 10.1186/1748-717X-1-8

Vanuytsel LJ, 2000, RADIOTHER ONCOL, V55, P317, DOI 10.1016/S0167-8140(00)00138-9

Wolthaus JWH, 2005, PHYS MED BIOL, V50, P1569, DOI 10.1088/0031-9155/50/7/017

Wolz G, 2007, NUKLEARMED-NUCL MED, V46, P43, DOI 10.1055/s-0037-1616625

NR 27

TC 42

Z9 42

U1 0

U2 0

PU ELSEVIER SCIENCE INC

PI NEW YORK

PA STE 800, 230 PARK AVE, NEW YORK, NY 10169 USA

SN 0360-3016

EI 1879-355X

J9 INT J RADIAT ONCOL

JI Int. J. Radiat. Oncol. Biol. Phys.

PD JAN 1

PY 2009

VL 73

IS 1

BP 103

EP 111

DI 10.1016/j.ijrobp.2008.03.063

PG 9

WC Oncology; Radiology, Nuclear Medicine & Medical Imaging

WE Science Citation Index Expanded (SCI-EXPANDED)

SC Oncology; Radiology, Nuclear Medicine & Medical Imaging

GA 385NF

UT WOS:000261820200017

PM 18632217

DA 2022-08-24

ER

PT J

AU Ubaldi, L

Valenti, V

Borgese, RF

Collura, G

Fantacci, ME

Ferrera, G

Iacoviello, G

Abbate, BF

Laruina, F

Tripoli, A

Retico, A

Marrale, M

AF Ubaldi, L.

Valenti, V.

Borgese, R. F.

Collura, G.

Fantacci, M. E.

Ferrera, G.

Iacoviello, G.

Abbate, B. F.

Laruina, F.

Tripoli, A.

Retico, A.

Marrale, M.

TI Strategies to develop radiomics and machine learning models for lung

cancer stage and histology prediction using small data samples

SO PHYSICA MEDICA-EUROPEAN JOURNAL OF MEDICAL PHYSICS

LA English

DT Article

DE Radiomics; Machine learning; Cross validation; Non-small cell lung

cancer

ID STEREOTACTIC ABLATIVE RADIOTHERAPY; ARTIFICIAL-INTELLIGENCE;

RADIATION-THERAPY; BODY RADIOTHERAPY; MEDICAL IMAGES; SURVIVAL;

CLASSIFICATION; DIAGNOSIS; OUTCOMES; SBRT

AB Predictive models based on radiomics and machine-learning (ML) need large and annotated datasets for training, often difficult to collect. We designed an operative pipeline for model training to exploit data already available to the scientific community. The aim of this work was to explore the capability of radiomic features in predicting tumor histology and stage in patients with non-small cell lung cancer (NSCLC).

We analyzed the radiotherapy planning thoracic CT scans of a proprietary sample of 47 subjects (L-RT) and integrated this dataset with a publicly available set of 130 patients from the MAASTRO NSCLC collection (Lung1). We implemented intra- and inter-sample cross-validation strategies (CV) for evaluating the ML predictive model performances with not so large datasets.

We carried out two classification tasks: histology classification (3 classes) and overall stage classification (two classes: stage I and II). In the first task, the best performance was obtained by a Random Forest classifier, once the analysis has been restricted to stage I and II tumors of the Lung1 and L-RT merged dataset (AUC = 0.72 +/- 0.11). For the overall stage classification, the best results were obtained when training on Lung1 and testing of L-RT dataset (AUC = 0.72 +/- 0.04 for Random Forest and AUC = 0.84 +/- 0.03 for linear-kernel Support Vector Machine).

According to the classification task to be accomplished and to the heterogeneity of the available dataset(s), different CV strategies have to be explored and compared to make a robust assessment of the potential of a predictive model based on radiomics and ML.

C1 [Ubaldi, L.; Fantacci, M. E.; Laruina, F.] Univ Pisa, Phys Dept, Pisa, Italy.

[Ubaldi, L.; Fantacci, M. E.; Laruina, F.; Retico, A.] Natl Inst Nucl Phys INFN, Pisa Div, Largo Bruno Pontecorvo 3, I-56127 Pisa, Italy.

[Valenti, V.; Tripoli, A.] REM Radiat Therapy Ctr, I-95029 Catania, Italy.

[Borgese, R. F.; Collura, G.; Marrale, M.] Univ Palermo, Phys & Chem Dept Emilio Segre, Palermo, Italy.

[Borgese, R. F.; Collura, G.; Marrale, M.] Natl Inst Nucl Phys INFN, Catania Div, Catania, Italy.

[Iacoviello, G.; Abbate, B. F.] ARNAS Civ Hosp, Med Phys Dept, Palermo, Italy.

[Ferrera, G.] ARNAS Civ Hosp, Radiat Oncol, Palermo, Italy.

RP Retico, A (通讯作者)，Natl Inst Nucl Phys INFN, Pisa Div, Largo Bruno Pontecorvo 3, I-56127 Pisa, Italy.

RI Fantacci, Maria Evelina/ABD-4227-2020; ferrera, giuseppe/AAD-2649-2019;

MARRALE, MAURIZIO/I-9926-2014

OI Fantacci, Maria Evelina/0000-0003-2130-4372; MARRALE,

MAURIZIO/0000-0002-0091-3243; Valenti, Vito/0000-0002-2247-339X;

Laruina, Francesco/0000-0002-9401-9460; Collura,

Giorgio/0000-0003-0169-174X

FU INFN

FX This work has been carried out within the Artificial Intelligence in

Medicine (AIM) project funded by INFN (CSN5, 2019-2021) , https://

www.pi.infn.it/aim.

CR Aerts HJWL, 2014, NAT COMMUN, V5, DOI 10.1038/ncomms5006

Arcangeli S, 2015, BRIT J RADIOL, V88, DOI 10.1259/bjr.20140728

Astaraki M, 2021, PHYS MEDICA, V83, P146, DOI 10.1016/j.ejmp.2021.03.013

Aurelien, 2019, HANDS ON MACHINE LEA

Avanzo M, 2021, PHYS MEDICA, V83, P221, DOI 10.1016/j.ejmp.2021.04.010

Avanzo M, 2020, MED PHYS, V47, pE185, DOI 10.1002/mp.13678

Baine MJ, 2018, LUNG CANCER, V118, P20, DOI 10.1016/j.lungcan.2018.01.021

Balagurunathan Y, PHYS MED, V83, P72, DOI [10.1016/j, DOI 10.1016/J]

Baumann P, 2009, J CLIN ONCOL, V27, P3290, DOI 10.1200/JCO.2008.21.5681

Beckers R, 2021, PHYS MEDICA, V83, P1, DOI 10.1016/j.ejmp.2021.02.011

Biship C. M, 2006, PATTERN RECOGN, V4

Brawley OW, 2011, CA-CANCER J CLIN, V61, P67, DOI 10.3322/caac.20108

Castiglioni I, 2021, PHYS MEDICA, V83, P9, DOI 10.1016/j.ejmp.2021.02.006

Chang JY, 2014, INT J RADIAT ONCOL, V88, P1120, DOI 10.1016/j.ijrobp.2014.01.022

Chansky K, 2009, J THORAC ONCOL, V4, P792, DOI 10.1097/JTO.0b013e3181a7716e

Chi A, 2010, RADIOTHER ONCOL, V94, P1, DOI 10.1016/j.radonc.2009.12.008

Crino L, 2010, ANN ONCOL, V21, pv103, DOI 10.1093/annonc/mdq207

Cuccia F, 2020, J GERIATR ONCOL, V11, P475, DOI 10.1016/j.jgo.2019.05.002

Diaz O, 2021, PHYS MEDICA, V81, P141, DOI 10.1016/j.ejmp.2020.11.037

Edge SB, 2010, ANN SURG ONCOL, V17, P1471, DOI 10.1245/s10434-010-0985-4

Ferini G, 2021, IN VIVO, V35, P1379, DOI 10.21873/invivo.12390

Ferlay J, 2019, INT J CANCER, V144, P1941, DOI 10.1002/ijc.31937

Figlia V, 2018, RADIOL MED, V123, P406, DOI 10.1007/s11547-018-0858-7

Franks KN, 2015, CLIN ONCOL-UK, V27, P280, DOI 10.1016/j.clon.2015.01.006

Ganeshan B, 2010, CANCER IMAGING, V10, P137, DOI 10.1102/1470-7330.2010.0021

Gillies RJ, 2016, RADIOLOGY, V278, P563, DOI 10.1148/radiol.2015151169

Halabi S, 2014, J CLIN ONCOL, V32, P671, DOI 10.1200/JCO.2013.52.3696

Hofman V, 2014, B CANCER, V101, P958, DOI 10.1684/bdc.2014.2041

Hrnjica B, 2018, EMERGING RES OPPORTU, P310, DOI [DOI 10.4018/978-1-5225-6005-0, 10.4018/978-1-5225-6005-0]

Kikinis R., 2014, INTRAOPERATIVE IMAGI, P277, DOI DOI 10.1007/978-1-4614-7657-3_19

Kortesniemi M, 2018, PHYS MEDICA, V56, P90, DOI 10.1016/j.ejmp.2018.11.005

Kumar V, 2012, MAGN RESON IMAGING, V30, P1234, DOI 10.1016/j.mri.2012.06.010

Lambin P, 2012, EUR J CANCER, V48, P441, DOI 10.1016/j.ejca.2011.11.036

Louie AV, 2014, CHEST, V146, P1021, DOI 10.1378/chest.13-2924

Manco L, 2021, PHYS MEDICA, V83, P194, DOI 10.1016/j.ejmp.2021.03.026

Muren LP, 2013, RADIOTHER ONCOL, V109, P337, DOI 10.1016/j.radonc.2013.11.007

Nardone V, 2020, ONCOL LETT, V19, P1559, DOI 10.3892/ol.2019.11220

Onishi H, 2004, CANCER-AM CANCER SOC, V101, P1623, DOI 10.1002/cncr.20539

Ost D, 2008, AM J RESP CRIT CARE, V177, P516, DOI 10.1164/rccm.200706-815OC

Papadimitroulas P, 2021, PHYS MEDICA, V83, P108, DOI 10.1016/j.ejmp.2021.03.009

Patil R, 2016, TOMOGRAPHY, V2, P374, DOI 10.18383/j.tom.2016.00244

Pedregosa F, 2011, J MACH LEARN RES, V12, P2825

Pieper S, 2004, 2004 2ND IEEE INTERNATIONAL SYMPOSIUM ON BIOMEDICAL IMAGING: MACRO TO NANO, VOLS 1 and 2, P632

Raschka S., 2017, MACHINE LEARNING PYT

Rizzo Stefania, 2018, Eur Radiol Exp, V2, P36, DOI 10.1186/s41747-018-0068-z

Rusthoven KE, 2010, JAMA-J AM MED ASSOC, V303, P2354, DOI 10.1001/jama.2010.777

Scott WJ, 2007, CHEST, V132, p234S, DOI 10.1378/chest.07-1378

Senthi S, 2013, RADIOTHER ONCOL, V106, P276, DOI 10.1016/j.radonc.2013.01.004

Shiue K, 2018, J THORAC ONCOL, V13, P1549, DOI 10.1016/j.jtho.2018.06.007

Tran B, 2012, J CLIN ONCOL, V30, P647, DOI 10.1200/JCO.2011.39.2316

Traverso A, 2018, INT J RADIAT ONCOL, V102, P1143, DOI 10.1016/j.ijrobp.2018.05.053

Vadala RE, 2016, CLIN TRANSL ONCOL, V18, P1158, DOI 10.1007/s12094-016-1552-7

van Griethuysen JJM, 2017, CANCER RES, V77, pE104, DOI 10.1158/0008-5472.CAN-17-0339

Vansteenkiste J, 2014, ANN ONCOL, V25, P1462, DOI 10.1093/annonc/mdu089

Vellido A, 2020, NEURAL COMPUT APPL, V32, P18069, DOI 10.1007/s00521-019-04051-w

Woody NM, 2017, J THORAC ONCOL, V12, P510, DOI 10.1016/j.jtho.2016.11.002

Wu WM, 2016, FRONT ONCOL, V6, DOI 10.3389/fonc.2016.00071

Yip SSF, 2016, PHYS MED BIOL, V61, pR150, DOI 10.1088/0031-9155/61/13/R150

Zhang JX, 2013, LANCET ONCOL, V14, P1295, DOI 10.1016/S1470-2045(13)70491-1

NR 59

TC 3

Z9 3

U1 4

U2 8

PU ELSEVIER SCI LTD

PI OXFORD

PA THE BOULEVARD, LANGFORD LANE, KIDLINGTON, OXFORD OX5 1GB, OXON, ENGLAND

SN 1120-1797

EI 1724-191X

J9 PHYS MEDICA

JI Phys. Medica

PD OCT

PY 2021

VL 90

BP 13

EP 22

DI 10.1016/j.ejmp.2021.08.015

EA SEP 2021

PG 10

WC Radiology, Nuclear Medicine & Medical Imaging

WE Science Citation Index Expanded (SCI-EXPANDED)

SC Radiology, Nuclear Medicine & Medical Imaging

GA UY5ZN

UT WOS:000701601700003

PM 34521016

OA Green Published

DA 2022-08-24

ER

PT J

AU Tortora, M

Cordelli, E

Sicilia, R

Miele, M

Matteucci, P

Iannello, G

Ramella, S

Soda, P

AF Tortora, Matteo

Cordelli, Ermanno

Sicilia, Rosa

Miele, Marianna

Matteucci, Paolo

Iannello, Giulio

Ramella, Sara

Soda, Paolo

TI Deep Reinforcement Learning for Fractionated Radiotherapy in Non-Small

Cell Lung Carcinoma

SO ARTIFICIAL INTELLIGENCE IN MEDICINE

LA English

DT Article

DE Deep reinforcement learning; D3QN; Particle swarm optimization; NSCLC;

Radiation therapy; Tumour treatment optimization

ID MATHEMATICAL-MODELS; OPTIMIZATION; CANCER; TISSUE; TUMOR

AB Lung cancer is by far the leading cause of cancer death among both men and women. Radiation therapy is one of the main approaches to lung cancer treatment, and its planning is crucial for the therapy outcome. However, the current practice that uniformly delivers the dose does not take into account the patient-specific tumour features that may affect treatment success. Since radiation therapy is by its very nature a sequential procedure, Deep Reinforcement Learning (DRL) is a well-suited methodology to overcome this limitation. In this respect, in this work we present a DRL controller optimizing the daily dose fraction delivered to the patient on the basis of CT scans collected over time during the therapy, offering a personalized treatment not only for volume adaptation, as currently intended, but also for daily fractionation. Furthermore, this contribution introduces a virtual radiotherapy environment based on a set of ordinary differential equations modelling the tissue radiosensitivity by combining both the effect of the radiotherapy treatment and cell growth. Their parameters are estimated from CT scans routinely collected using the Particle Swarm Optimization algorithm. This permits the DRL to learn the optimal behaviour through an iterative trial and error process with the environment. We performed several experiments considering three rewards functions modelling treatment strategies with different tissue aggres-siveness and two exploration strategies for the exploration-exploitation dilemma. The results show that our DRL approach can adapt to radiation therapy treatment, optimizing its behaviour according to the different reward functions and outperforming the current clinical practice.

C1 [Tortora, Matteo; Cordelli, Ermanno; Sicilia, Rosa; Iannello, Giulio; Soda, Paolo] Univ Campus Biomed Rome, Dept Engn, Unit Comp Syst & Bioinformat, Via Alvaro del Portillo 21, I-00128 Rome, Italy.

[Miele, Marianna; Matteucci, Paolo; Ramella, Sara] Univ Campus Biomed Rome, Dept Med, Radiat Oncol, Via Alvaro del Portillo 21, I-00128 Rome, Italy.

RP Cordelli, E (通讯作者)，Univ Campus Biomed Rome, Dept Engn, Unit Comp Syst & Bioinformat, Via Alvaro del Portillo 21, I-00128 Rome, Italy.

EM m.tortora@unicampus.it; e.cordelli@unicampus.it; r.sicilia@unicampus.it;

m.miele@unicampus.it; p.matteucci@unicampus.it; g.iannello@unicampus.it;

s.ramella@unicampus.it; p.soda@unicampus.it

RI Sicilia, Rosa/AAC-6012-2022; RAMELLA, SARA/AAC-6523-2022

OI Sicilia, Rosa/0000-0002-2513-0827; RAMELLA, SARA/0000-0002-5782-7717;

Tortora, Matteo/0000-0002-3932-7380

CR Arulkumaran K, 2017, IEEE SIGNAL PROC MAG, V34, P26, DOI 10.1109/MSP.2017.2743240

Belfatto A, 2016, IEEE J BIOMED HEALTH, V20, P802, DOI 10.1109/JBHI.2015.2453437

Bentzen SM., 2009, BASIC CLIN RADIOBIOL, V4th, P120

Bentzen SM, 2012, RADIOTHER ONCOL, V105, P266, DOI 10.1016/j.radonc.2012.10.006

Benzekry S, 2014, PLOS COMPUT BIOL, V10, DOI 10.1371/journal.pcbi.1003800

Bibault JE, 2013, CANCER METAST REV, V32, P479, DOI 10.1007/s10555-013-9419-7

Bodgi L, 2016, J THEOR BIOL, V394, P93, DOI 10.1016/j.jtbi.2016.01.018

Boski M, 2017, 2017 10TH INTERNATIONAL WORKSHOP ON MULTIDIMENSIONAL (ND) SYSTEMS (NDS)

Bray F, 2018, CA-CANCER J CLIN, V68, P394, DOI 10.3322/caac.21492

Burnet Neil G, 2004, Cancer Imaging, V4, P153, DOI 10.1102/1470-7330.2004.0054

Busoniu L, 2008, IEEE T SYST MAN CY C, V38, P156, DOI 10.1109/TSMCC.2007.913919

Chapman JD, 2016, RADIOTHERAPY TREATME, V1st

Coronato A, 2022, IEEE T KNOWL DATA EN, V34, P3095, DOI 10.1109/TKDE.2020.3023553

Coronato A, 2020, ARTIF INTELL MED, V109, DOI 10.1016/j.artmed.2020.101964

Dong Y, 2019, BMC MED INF DECIS MA, V19, P19

Ertefaie A, 2016, STAT MED, V35, P2221, DOI 10.1002/sim.6859

Escandell-Montero P, 2014, ARTIF INTELL MED, V62, P47, DOI 10.1016/j.artmed.2014.07.004

Floreano D., 2008, BIOINSPIRED ARTIFICI

Fortunato M, INT C LEARN REPR

Fowler J. F., 2011, TECHNICAL BASIS RAD, P3

FOWLER JF, 1989, BRIT J RADIOL, V62, P679, DOI 10.1259/0007-1285-62-740-679

Gareth J., 2013, INTRO STAT LEARNING

Gerlee P, 2013, CANCER RES, V73, P2407, DOI 10.1158/0008-5472.CAN-12-4355

Gottesman O, 2019, NAT MED, V25, P16, DOI 10.1038/s41591-018-0310-5

Hasselt H. V., 2010, ADV NEURAL INFORM PR, V23, P2613

Hessel M, 2018, AAAI CONF ARTIF INTE, P3215

ISO I., 2007, MED DEVICES APPL RIS

Jalalimanesh A, 2017, J EXP THEOR ARTIF IN, V29, P1071, DOI 10.1080/0952813X.2017.1292319

Jalalimanesh A, 2017, MATH COMPUT SIMULAT, V133, P235, DOI 10.1016/j.matcom.2016.05.008

Jones B, 1999, ACTA ONCOL, V38, P883, DOI 10.1080/028418699432572

Kennedy J., 1995, 1995 IEEE International Conference on Neural Networks Proceedings (Cat. No.95CH35828), P1942, DOI 10.1109/ICNN.1995.488968

King DB, 2015, ACS SYM SER, V1214, P1

LeCun Y, 2015, NATURE, V521, P436, DOI DOI 10.1038/NATURE14539

Lei Ba J., 2016, ABS160706450 CORR

Liu Y, 2017, 2017 IEEE INTERNATIONAL CONFERENCE ON HEALTHCARE INFORMATICS (ICHI), P380, DOI 10.1109/ICHI.2017.45

Lou B, 2019, LANCET DIGIT HEALTH, V1, pE136, DOI 10.1016/S2589-7500(19)30058-5

Marcu LG, 2012, COMPUT MATH METHOD M, V2012, DOI 10.1155/2012/960256

McMahon SJ, 2019, CANCERS, V11, DOI 10.3390/cancers11020205

Mnih V., 2013, PLAYING ATARI DEEP R

Mnih V, 2015, NATURE, V518, P529, DOI 10.1038/nature14236

Molina JR, 2008, MAYO CLIN PROC, V83, P584, DOI 10.4065/83.5.584

Murphy H, 2016, BMC CANCER, V16, DOI 10.1186/s12885-016-2164-x

Ngo Phuong D., 2018, 2018 IEEE EMBS International Conference on Biomedical & Health Informatics (BHI), P333, DOI 10.1109/BHI.2018.8333436

NORTON L, 1988, CANCER RES, V48, P7067

Orth M, 2014, RADIAT ENVIRON BIOPH, V53, P1, DOI 10.1007/s00411-013-0497-2

Padmanabhan R, 2015, BIOMED SIGNAL PROCES, V22, P54, DOI 10.1016/j.bspc.2015.05.013

Peng X, 2018, AMIA ANN S P

Piot B, 2017, IEEE T NEUR NET LEAR, V28, P1814, DOI 10.1109/TNNLS.2016.2543000

Ramella S, 2018, PLOS ONE, V13, DOI 10.1371/journal.pone.0207455

Russell S.J., 2016, ARTIF INTELL, V3rd ed

Santiago A, 2016, RADIAT ONCOL, V11, DOI 10.1186/s13014-016-0643-5

Scheenstra AEH, 2014, INT J RADIAT ONCOL, V88, P224, DOI 10.1016/j.ijrobp.2013.10.015

Scheidegger S, 2011, Z MED PHYS, V21, P164, DOI 10.1016/j.zemedi.2010.11.001

Silver D, 2016, NATURE, V529, P484, DOI 10.1038/nature16961

Sutton R. S., 1988, Machine Learning, V3, P9, DOI 10.1023/A:1022633531479

Sutton RS, 2018, ADAPT COMPUT MACH LE, P1

Thames H D., 1987, FRACTIONATION RADIOT

Tseng HH, 2017, MED PHYS, V44, P6690, DOI 10.1002/mp.12625

US National Library of Medicine L. H. N. C. f. B. C., WHAT IS DIFF PREC ME

van Hasselt H, 2016, AAAI CONF ARTIF INTE, P2094

van Leeuwen CM, 2018, RADIAT ONCOL, V13, DOI 10.1186/s13014-018-1040-z

Vincent R., 2014, REINFORCEMENT LEARNI

Virtanen P, 2020, NAT METHODS, V17, P261, DOI 10.1038/s41592-019-0686-2

Wang ZY, 2016, PR MACH LEARN RES, V48

Winsor CP, 1932, P NATL ACAD SCI USA, V18, P1, DOI 10.1073/pnas.18.1.1

WITHERS H R, 1983, Radiotherapy and Oncology, V1, P187, DOI 10.1016/S0167-8140(83)80021-8

Yu C. M., ARXIV PREPRINT ARXIV

NR 67

TC 0

Z9 1

U1 3

U2 7

PU ELSEVIER

PI AMSTERDAM

PA RADARWEG 29, 1043 NX AMSTERDAM, NETHERLANDS

SN 0933-3657

EI 1873-2860

J9 ARTIF INTELL MED

JI Artif. Intell. Med.

PD SEP

PY 2021

VL 119

AR 102137

DI 10.1016/j.artmed.2021.102137

EA AUG 2021

PG 13

WC Computer Science, Artificial Intelligence; Engineering, Biomedical;

Medical Informatics

WE Science Citation Index Expanded (SCI-EXPANDED)

SC Computer Science; Engineering; Medical Informatics

GA UR8WM

UT WOS:000697022300003

PM 34531006

DA 2022-08-24

ER

PT J

AU Kakar, M

Mencattini, A

Salmeri, M

AF Kakar, Manish

Mencattini, Arianna

Salmeri, Marcello

TI Extracting Fuzzy Classification Rules from Texture Segmented HRCT Lung

Images

SO JOURNAL OF DIGITAL IMAGING

LA English

DT Article

DE NSCLC; IGRT; FIS; Rule-based classification

ID COMPUTER-AIDED DIAGNOSIS; PULMONARY NODULES; CT; RADIOTHERAPY; MOTION;

DELINEATION; VOLUME; SIZE

AB Automatic tools for detection and identification of lung and lesion from high-resolution CT (HRCT) are becoming increasingly important both for diagnosis and for delivering high-precision radiation therapy. However, development of robust and interpretable classifiers still presents a challenge especially in case of non-small cell lung carcinoma (NSCLC) patients. In this paper, we have attempted to devise such a classifier by extracting fuzzy rules from texture segmented regions from HRCT images of NSCLC patients. A fuzzy inference system (FIS) has been constructed starting from a feature extraction procedure applied on overlapping regions from the same organs and deriving simple if-then rules so that more linguistically interpretable decisions can be implemented. The proposed method has been tested on 138 regions extracted from CT scan images acquired from patients with lung cancer. Assuming two classes of tissues C1 (healthy tissues) and C2 (lesion) as negative and positive, respectively; preliminary results report an AUC = 0.98 for lesions and AUC = 0.93 for healthy tissue, with an optimal operating condition related to sensitivity = 0.96, and specificity = 0.98 for lesions and sensitivity 0.99, and specificity = 0.94 for healthy tissue. Finally, the following results have been obtained: false-negative rate (FNR) = 6 % (C1), FNR = 2 % (C2), false-positive rate (FPR) = 4 % (C1), FPR = 3 % (C2), true-positive rate (TPR) = 94 %, (C1) and TPR = 98 % (C2).

C1 [Kakar, Manish] Oslo Univ Hosp, Inst Canc Res, Dept Radiat Biol, Div Canc & Surg, N-0310 Oslo, Norway.

[Mencattini, Arianna; Salmeri, Marcello] Univ Roma Tor Vergata, Dept Elect Engn, Rome, Italy.

RP Mencattini, A (通讯作者)，Univ Roma Tor Vergata, Dept Elect Engn, Rome, Italy.

EM Manish.Kakar@rr-research.no; mencattini@ing.uniroma2.it;

salmeri@ing.uniroma2.it

RI Kakar, Manish/AAF-8066-2020; Mencattini, Arianna/K-7910-2015; Kakar,

Manish/N-6828-2019

OI Mencattini, Arianna/0000-0002-3753-0457;

CR Andersson ER, 2007, FUZZY ROUGH TECHNIQU

Armato SG, 2003, MED PHYS, V30, P1188, DOI 10.1118/1.1573210

Armato SG, 1999, RADIOGRAPHICS, V19, P1303, DOI 10.1148/radiographics.19.5.g99se181303

Arslan S, 2002, MED SCI MONITOR, V8, P493

Berbeco RI, 2005, PHYS MED BIOL, V50, P3655, DOI 10.1088/0031-9155/50/16/001

Chawla NV, 2002, J ARTIF INTELL RES, V16, P321, DOI 10.1613/jair.953

Colgan R, 2008, PHYS MED BIOL, V53, P5815, DOI 10.1088/0031-9155/53/20/017

Dewas S, 2011, RADIAT ONCOL, V6, DOI 10.1186/1748-717X-6-118

Ekberg L, 1998, RADIOTHER ONCOL, V48, P71, DOI 10.1016/S0167-8140(98)00046-2

Ferrero A., 2010, IEEE INSTR MEAS TECH

Geraghty PR, 2003, RADIOLOGY, V229, P475, DOI 10.1148/radiol.2291020499

Giraud P, 2002, RADIOTHER ONCOL, V62, P27, DOI 10.1016/S0167-8140(01)00444-3

Goo JM, 2011, KOREAN J RADIOL, V12, P145, DOI 10.3348/kjr.2011.12.2.145

GRAHAM MV, 1994, INT J RADIAT ONCOL, V29, P1105, DOI 10.1016/0360-3016(94)90407-3

Hamilton C S, 1992, Clin Oncol (R Coll Radiol), V4, P141, DOI 10.1016/S0936-6555(05)81075-1

Hoisak JDP, 2004, INT J RADIAT ONCOL, V60, P1298, DOI 10.1016/j.ijrobp.2004.07.681

Kakar M., 2009, IEEE C FUZZ SYST JEJ

Kakar M, 2009, COMPUT MED IMAG GRAP, V33, P72, DOI 10.1016/j.compmedimag.2008.10.009

Keall PJ, 2005, MED PHYS, V32, P942, DOI 10.1118/1.1879152

LEUNENS G, 1993, RADIOTHER ONCOL, V29, P169, DOI 10.1016/0167-8140(93)90243-2

Li Q, 2007, COMPUT MED IMAG GRAP, V31, P248, DOI 10.1016/j.compmedimag.2007.02.005

Mencattini A., 2011, IEEE INT WORKSH MED

Mencattini A, 2012, INT J COMPUT ASS RAD, V7, P573, DOI 10.1007/s11548-011-0659-0

PADLEY SPG, 1991, CLIN RADIOL, V44, P222, DOI 10.1016/S0009-9260(05)80183-7

Seiler PG, 2000, PHYS MED BIOL, V45, pN103, DOI 10.1088/0031-9155/45/9/402

Sharp GC, 2004, PHYS MED BIOL, V49, P5347, DOI 10.1088/0031-9155/49/23/011

Stevens CW, 2001, INT J RADIAT ONCOL, V51, P62, DOI 10.1016/S0360-3016(01)01621-2

TAIT DM, 1990, EUR J CANCER, V26, P750, DOI 10.1016/0277-5379(90)90135-G

URIE MM, 1991, INT J RADIAT ONCOL, V21, P91, DOI 10.1016/0360-3016(91)90170-9

Vorwerk H, 2009, RADIOTHER ONCOL, V91, P455, DOI 10.1016/j.radonc.2009.03.014

WATANABE H, 1994, IEEE T FUZZY SYST, V2, P267, DOI 10.1109/91.324806

Wiemker R, 2002, PROC SPIE, V4684, P677, DOI 10.1117/12.467210

Xing L, 2007, SEMIN RADIAT ONCOL, V17, P245, DOI 10.1016/j.semradonc.2007.07.004

ZADEH LA, 1965, INFORM CONTROL, V8, P338, DOI 10.1016/S0019-9958(65)90241-X

NR 34

TC 0

Z9 0

U1 0

U2 6

PU SPRINGER

PI NEW YORK

PA 233 SPRING ST, NEW YORK, NY 10013 USA

SN 0897-1889

EI 1618-727X

J9 J DIGIT IMAGING

JI J. Digit. Imaging

PD APR

PY 2013

VL 26

IS 2

BP 227

EP 238

DI 10.1007/s10278-012-9514-2

PG 12

WC Radiology, Nuclear Medicine & Medical Imaging

WE Science Citation Index Expanded (SCI-EXPANDED)

SC Radiology, Nuclear Medicine & Medical Imaging

GA 191SX

UT WOS:000322434000012

PM 22890442

OA Green Published

DA 2022-08-24

ER

PT J

AU Della Gala, G

Dirkx, MLP

Hoekstra, N

Fransen, D

Lanconelli, N

van de Pol, M

Heijmen, BJM

Petit, SF

AF Della Gala, Giuseppe

Dirkx, Maarten L. P.

Hoekstra, Nienke

Fransen, Dennie

Lanconelli, Nico

van de Pol, Marjan

Heijmen, Ben J. M.

Petit, Steven F.

TI Fully automated VMAT treatment planning for advanced-stage NSCLC

patients

SO STRAHLENTHERAPIE UND ONKOLOGIE

LA English

DT Article

DE Radiotherapy; intensity-modulated; Volumetric-modulated arc therapy;

Computer-assisted radiotherapy planning; Non-small cell lung carcinoma;

Organs at risk

ID MODULATED ARC THERAPY; PROSTATE-CANCER; LUNG-CANCER; OPTIMIZATION;

PLANS; NECK; HEAD; GENERATION

AB Purpose To develop a fully automated procedure for multicriterial volumetric modulated arc therapy (VMAT) treatment planning (autoVMAT) for stage III/IV non-small cell lung cancer (NSCLC) patients treated with curative intent.

Materials and methods After configuring the developed autoVMAT system for NSCLC, autoVMAT plans were compared with manually generated clinically delivered intensity-modulated radiotherapy (IMRT) plans for 41 patients. AutoVMAT plans were also compared to manually generated VMAT plans in the absence of time pressure. For 16 patients with reduced planning target volume (PTV) dose prescription in the clinical IMRT plan (to avoid violation of organs at risk tolerances), the potential for dose escalation with autoVMAT was explored.

Results Two physicians evaluated 35/41 autoVMAT plans (85%) as clinically acceptable. Compared to the manually generated IMRT plans, autoVMAT plans showed statistically significant improved PTV coverage (V-95% increased by 1.1% +/- 1.1%), higher dose conformity (R-50 reduced by 12.2% +/- 12.7%), and reduced mean lung, heart, and esophagus doses (reductions of 0.9 Gy +/- 1.0 Gy, 1.5 Gy +/- 1.8 Gy, 3.6 Gy +/- 2.8 Gy, respectively, all p < 0.001). To render the six remaining autoVMAT plans clinically acceptable, a dosimetrist needed less than 10 min hands-on time for fine-tuning. AutoVMAT plans were also considered equivalent or better than manually optimized VMAT plans. For 6/16 patients, autoVMAT allowed tumor dose escalation of 5-10 Gy.

Conclusion Clinically deliverable, high-quality autoVMAT plans can be generated fully automatically for the vast majority of advanced-stage NSCLC patients. For a subset of patients, autoVMAT allowed for tumor dose escalation.

C1 [Della Gala, Giuseppe; Dirkx, Maarten L. P.; Hoekstra, Nienke; Fransen, Dennie; van de Pol, Marjan; Heijmen, Ben J. M.; Petit, Steven F.] Erasmus MC, Dept Radiat Oncol, Inst Canc, NL-3008 AE Rotterdam, Netherlands.

[Della Gala, Giuseppe; Lanconelli, Nico] Univ Bologna, Scuola Sci, Alma Mater Studiorum, Bologna, Italy.

[Petit, Steven F.] Harvard Med Sch, Dept Radiat Oncol, Massachusetts Gen Hosp, Boston, MA USA.

RP Dirkx, MLP (通讯作者)，Erasmus MC, Dept Radiat Oncol, Inst Canc, NL-3008 AE Rotterdam, Netherlands.

EM m.dirkx@erasmusmc.nl

RI Hoekstra, Nienke/ABG-1917-2020

OI Hoekstra, Nienke/0000-0001-7355-6219; Della Gala,

Giuseppe/0000-0002-7143-6528

FU Dutch Cancer Society [KWF EMCR 2014-6667]

FX The department of Radiation Oncology of Erasmus MC Cancer Institute has

research collaborations with Elekta AB and Accuray. G. Della Gala,

M.L.P. Dirkx, N. Hoekstra, D. Fransen, N. Lanconelli, M. van de Pol, and

B.J.M. Heijmen declare that they have no competing interests. S.F. Petit

receives financial support from the Dutch Cancer Society (KWF EMCR

2014-6667).

CR Amit G, 2015, MED PHYS, V42, P770, DOI 10.1118/1.4905111

Bezjak A, 2012, Clin Oncol (R Coll Radiol), V24, P508, DOI 10.1016/j.clon.2012.05.007

Boylan C, 2014, J APPL CLIN MED PHYS, V15, P213, DOI 10.1120/jacmp.v15i1.4530

Breedveld S, 2012, MED PHYS, V39, P951, DOI 10.1118/1.3676689

Fiege J, 2011, MED PHYS, V38, P5217, DOI 10.1118/1.3615622

Fogliata A, 2014, RADIOTHER ONCOL, V113, P385, DOI 10.1016/j.radonc.2014.11.009

Hazell I, 2016, J APPL CLIN MED PHYS, V17, P272, DOI 10.1120/jacmp.v17i1.5901

Kamran SC, 2016, RADIOTHER ONCOL, V118, P515, DOI 10.1016/j.radonc.2015.12.028

Krayenbuehl J, 2015, RADIAT ONCOL, V10, DOI 10.1186/s13014-015-0533-2

Quan EM, 2012, INT J RADIAT ONCOL, V84, pE69, DOI 10.1016/j.ijrobp.2012.02.017

Sharfo AWM, 2015, RADIOTHER ONCOL, V114, P395, DOI 10.1016/j.radonc.2015.02.006

Sharpe MB, 2014, MED PHYS, V41, DOI 10.1118/1.4894496

Tol JP, 2016, MED PHYS, V43, P1818, DOI 10.1118/1.4944063

Voet PWJ, 2014, INT J RADIAT ONCOL, V88, P1175, DOI 10.1016/j.ijrobp.2013.12.046

Voet PWJ, 2013, INT J RADIAT ONCOL, V85, P866, DOI 10.1016/j.ijrobp.2012.04.015

Wu BB, 2012, INT J RADIAT ONCOL, V84, pE647, DOI 10.1016/j.ijrobp.2012.06.047

Zhang XD, 2011, PHYS MED BIOL, V56, P3873, DOI 10.1088/0031-9155/56/13/009

NR 17

TC 33

Z9 33

U1 1

U2 3

PU URBAN & VOGEL

PI MUNICH

PA NEUMARKTER STRASSE 43, D-81673 MUNICH, GERMANY

SN 0179-7158

EI 1439-099X

J9 STRAHLENTHER ONKOL

JI Strahlenther. Onkol.

PD MAY

PY 2017

VL 193

IS 5

BP 402

EP 409

DI 10.1007/s00066-017-1121-1

PG 8

WC Oncology; Radiology, Nuclear Medicine & Medical Imaging

WE Science Citation Index Expanded (SCI-EXPANDED)

SC Oncology; Radiology, Nuclear Medicine & Medical Imaging

GA ET2NM

UT WOS:000400110400006

PM 28314877

OA Green Published, hybrid

DA 2022-08-24

ER

PT J

AU Li, SL

Yang, N

Li, B

Zhou, ZG

Hao, HX

Folkert, MR

Iyengar, P

Westover, K

Choy, H

Timmerman, R

Jiang, S

Wang, J

AF Li, Shulong

Yang, Ning

Li, Bin

Zhou, Zhiguo

Hao, Hongxia

Folkert, Michael R.

Iyengar, Puneeth

Westover, Kenneth

Choy, Hak

Timmerman, Robert

Jiang, Steve

Wang, Jing

TI A pilot study using kernelled support tensor machine for distant failure

prediction in lung SBRT

SO MEDICAL IMAGE ANALYSIS

LA English

DT Article

DE Support tensor machine; NSCLC; SBRT; Radiomics; Medical imaging

ID STEREOTACTIC BODY RADIATION; CONVOLUTIONAL NEURAL-NETWORKS;

COMPUTER-AIDED DETECTION; FDG-PET; ALZHEIMERS-DISEASE; CANCER-TREATMENT;

TEXTURE ANALYSIS; RADIOMICS MODEL; IMAGE-ANALYSIS; FEATURES

AB We developed a kernelled support tensor machine (KSTM)-based model with tumor tensors derived from pre-treatment PET and CT imaging as input to predict distant failure in early stage non-small cell lung cancer (NSCLC) treated with stereotactic body radiation therapy (SBRT). The patient cohort included 110 early stage NSCLC patients treated with SBRT, 25 of whom experienced failure at distant sites. Three-dimensional tumor tensors were constructed and used as input for the KSTM-based classifier. A KSTM iterative algorithm with a convergent proof was developed to train the weight vectors for every mode of the tensor for the classifier. In contrast to conventional radiomics approaches that rely on handcrafted imaging features, the KSTM-based classifier uses 3D imaging as input, taking full advantage of the imaging information. The KSTM-based classifier preserves the intrinsic 3D geometry structure of the medical images and the correlation in the original images and trains the classification hyper-plane in an adaptive feature tensor space. The KSTM-based predictive algorithm was compared with three conventional machine learning models and three radiomics approaches. For PET and CT, the KSTM-based predictive method achieved the highest prediction results among the seven methods investigated in this study based on 10-fold cross validation and independent testing. (C) 2018 Elsevier B.V. All rights reserved.

C1 [Li, Shulong; Li, Bin] Southern Med Univ, Guangdong Prov Key Lab Med Image, Sch Biomed Engn, Proc, Guangzhou 510515, Guangdong, Peoples R China.

[Yang, Ning] Guangdong 2 Prov Peoples Hosp, Dept Med Imaging, Guangzhou 510317, Guangdong, Peoples R China.

[Zhou, Zhiguo; Folkert, Michael R.; Iyengar, Puneeth; Westover, Kenneth; Choy, Hak; Timmerman, Robert; Jiang, Steve; Wang, Jing] Univ Texas Southwestern Med Ctr Dallas, Dept Radiat Oncol, Dallas, TX 75235 USA.

[Hao, Hongxia] Xidian Univ, Sch Comp Sci & Technol, Xian 710071, Shaanxi, Peoples R China.

RP Wang, J (通讯作者)，Univ Texas Southwestern Med Ctr Dallas, Dept Radiat Oncol, Dallas, TX 75235 USA.

EM jing.wang@utsouthwestern.edu

RI Wang, Jing/N-7332-2019; Westover, Ken/AAZ-1795-2020; Hao,

Hongxia/AAO-7462-2020; Li, Shulong/AAK-9054-2020

OI Westover, Ken/0000-0003-3653-5923; Wang, Jing/0000-0002-8491-4146; Li,

Shulong/0000-0002-7466-4366

FU American Cancer Society [ACS-IRG-02-196]; National Institutes of Health

[5P30CA142543]; National Natural Science Foundation of China (NSFC)

[11771456]; NATIONAL CANCER INSTITUTE [P30CA142543] Funding Source: NIH

RePORTER

FX This work was partly supported by the American Cancer Society

(ACS-IRG-02-196), the National Institutes of Health (5P30CA142543) and

the National Natural Science Foundation of China (NSFC, 11771456). The

authors would like to thank Dr. Damiana Chiavolini for editing the

manuscript.

CR Abdi H, 2010, WIRES COMPUT STAT, V2, P433, DOI 10.1002/wics.101

Aerts H.J., 2014, NATURE COMMUN, V5

Ahmmed A., 2011, 2011 IEEE Symposium on Computers & Informatics (ISCI), P215, DOI 10.1109/ISCI.2011.5958914

[Anonymous], 2017, BRIEFINGS BIOINFORMA

Biswas S. K., 2016, ARXIV160907878

Chawla NV, 2002, J ARTIF INTELL RES, V16, P321, DOI 10.1613/jair.953

Chi A, 2010, RADIOTHER ONCOL, V94, P1, DOI 10.1016/j.radonc.2009.12.008

Chung A. G., 2015, ARXIV150900111

Church TR, 2013, NEW ENGL J MED, V368, P1980, DOI 10.1056/NEJMoa1209120

Ciompi F, 2015, MED IMAGE ANAL, V26, P195, DOI 10.1016/j.media.2015.08.001

Clarke K, 2012, RADIOTHER ONCOL, V104, P62, DOI 10.1016/j.radonc.2012.04.019

Cook GJR, 2014, CLIN TRANSL IMAGING, V2, P269, DOI 10.1007/s40336-014-0064-0

CORTES C, 1995, MACH LEARN, V20, P273, DOI 10.1023/A:1022627411411

Tao DC, 2007, KNOWL INF SYST, V13, P1, DOI 10.1007/s10115-006-0050-6

DAVIS LS, 1979, IEEE T PATTERN ANAL, V1, P251, DOI 10.1109/TPAMI.1979.4766921

De Lathauwer L., 1997, SIGNAL PROCESSING BA

Ettinger DS, 2010, J NATL COMPR CANC NE, V8, P740, DOI 10.6004/jnccn.2010.0056

Fletcher R. H., 2012, CLIN EPIDEMIOLOGY ES

Gillies RJ, 2016, RADIOLOGY, V278, P563, DOI 10.1148/radiol.2015151169

Ginsberg Robert J., 1995, Annals of Thoracic Surgery, V60, P615, DOI 10.1016/0003-4975(95)00537-U

Hao ZF, 2013, IEEE T IMAGE PROCESS, V22, P2911, DOI 10.1109/TIP.2013.2253485

Howlader N., SEER CANC STAT REV 1

Huang M, 2017, SCI REP, V7

Keogh E., 2017, ENCY MACHINE LEARNIN, P314, DOI DOI 10.1007/978-0-387-30164-8_192

Khamis H, 2017, MED IMAGE ANAL, V36, P15, DOI 10.1016/j.media.2016.10.007

Kohavi R, 1997, ARTIF INTELL, V97, P273, DOI 10.1016/S0004-3702(97)00043-X

Kolda TG, 2009, SIAM REV, V51, P455, DOI 10.1137/07070111X

Kononenko I, 1997, APPL INTELL, V7, P39, DOI 10.1023/A:1008280620621

Kooi T, 2017, MED IMAGE ANAL, V35, P303, DOI 10.1016/j.media.2016.07.007

Krizhevsky A, 2017, COMMUN ACM, V60, P84, DOI 10.1145/3065386

Kumar D, 2015, ARXIV150900117

Kumar V, 2012, MAGN RESON IMAGING, V30, P1234, DOI 10.1016/j.mri.2012.06.010

Lambin P, 2012, EUR J CANCER, V48, P441, DOI 10.1016/j.ejca.2011.11.036

Li SL, 2009, NONLINEAR ANAL-THEOR, V71, P5695, DOI 10.1016/j.na.2009.04.048

Lian CF, 2016, MED IMAGE ANAL, V32, P257, DOI 10.1016/j.media.2016.05.007

Liu HW, 2015, RADIOTHER ONCOL, V117, P71, DOI 10.1016/j.radonc.2015.08.027

Liu MX, 2017, MED IMAGE ANAL, V36, P123, DOI 10.1016/j.media.2016.11.002

Lovinfosse P, 2016, EUR J NUCL MED MOL I, V43, P1453, DOI 10.1007/s00259-016-3314-8

Madabhushi A, 2016, MED IMAGE ANAL, V33, P170, DOI 10.1016/j.media.2016.06.037

Magnin B, 2009, NEURORADIOLOGY, V51, P73, DOI 10.1007/s00234-008-0463-x

MARTINI N, 1995, J THORAC CARDIOV SUR, V109, P120, DOI 10.1016/S0022-5223(95)70427-2

Miller KD, 2016, CA-CANCER J CLIN, V66, P271, DOI 10.3322/caac.21349

Ming Liu, 2010, Proceedings of the 2010 Sixth International Conference on Intelligent Information Hiding and Multimedia Signal Processing (IIHMSP 2010), P462, DOI 10.1109/IIHMSP.2010.118

Namburete AIL, 2015, MED IMAGE ANAL, V21, P72, DOI 10.1016/j.media.2014.12.006

Nath SK, 2011, RADIOTHER ONCOL, V99, P12, DOI 10.1016/j.radonc.2011.02.006

Nikolaev A, 2016, INT J RADIAT ONCOL, V96, pE424, DOI 10.1016/j.ijrobp.2016.06.1695

OTSU N, 1979, IEEE T SYST MAN CYB, V9, P62, DOI 10.1109/TSMC.1979.4310076

Parmar C, 2015, SCI REP-UK, V5, DOI 10.1038/srep13087

RAUDYS SJ, 1991, IEEE T PATTERN ANAL, V13, P252, DOI 10.1109/34.75512

Roth HR, 2016, IEEE T MED IMAGING, V35, P1170, DOI 10.1109/TMI.2015.2482920

SATHYAMURTHY N, 1975, J CHEM PHYS, V63, P464, DOI 10.1063/1.431126

Senthi S, 2012, LANCET ONCOL, V13, P802, DOI 10.1016/S1470-2045(12)70242-5

Shashua A., 2001, P IEEE COMP SOC COMP, V1, P1

Shen Wei, 2015, Inf Process Med Imaging, V24, P588, DOI 10.1007/978-3-319-19992-4_46

Shin HC, 2016, IEEE T MED IMAGING, V35, P1285, DOI 10.1109/TMI.2016.2528162

Tajbakhsh N, 2016, IEEE T MED IMAGING, V35, P1299, DOI 10.1109/TMI.2016.2535302

Tan S, 2013, INT J RADIAT ONCOL, V85, P1375, DOI 10.1016/j.ijrobp.2012.10.017

Tao D., 2005, P 5 IEEE INT C DAT M, P8

Timmerman RD, 2014, INT J RADIAT ONCOL, V90, pS30, DOI 10.1016/j.ijrobp.2014.05.135

Timmerman R, 2010, JAMA-J AM MED ASSOC, V303, P1070, DOI 10.1001/jama.2010.261

Vallieres M, 2015, PHYS MED BIOL, V60, P5471, DOI 10.1088/0031-9155/60/14/5471

Vansteenkiste JF, 1999, J CLIN ONCOL, V17, P3201, DOI 10.1200/JCO.1999.17.10.3201

Wimmer G, 2016, MED IMAGE ANAL, V31, P16, DOI 10.1016/j.media.2016.02.001

Wu J, 2016, RADIOLOGY, V281, P270, DOI 10.1148/radiol.2016151829

Zhang LF, 2011, IEEE GEOSCI REMOTE S, V8, P374, DOI 10.1109/LGRS.2010.2077272

Zhou Z, 2017, ARXIV171001614

Zhou ZG, 2013, COMPUT BIOL MED, V43, P1462, DOI 10.1016/j.compbiomed.2013.07.023

Zhou ZG, 2017, PHYS MED BIOL, V62, P4460, DOI 10.1088/1361-6560/aa6ae5

Zhou ZG, 2016, RADIOTHER ONCOL, V119, P501, DOI 10.1016/j.radonc.2016.04.029

Zuluaga MA, 2015, MED IMAGE ANAL, V26, P185, DOI 10.1016/j.media.2015.09.001

NR 70

TC 10

Z9 10

U1 1

U2 22

PU ELSEVIER

PI AMSTERDAM

PA RADARWEG 29, 1043 NX AMSTERDAM, NETHERLANDS

SN 1361-8415

EI 1361-8423

J9 MED IMAGE ANAL

JI Med. Image Anal.

PD DEC

PY 2018

VL 50

BP 106

EP 116

DI 10.1016/j.media.2018.09.004

PG 11

WC Computer Science, Artificial Intelligence; Computer Science,

Interdisciplinary Applications; Engineering, Biomedical; Radiology,

Nuclear Medicine & Medical Imaging

WE Science Citation Index Expanded (SCI-EXPANDED)

SC Computer Science; Engineering; Radiology, Nuclear Medicine & Medical

Imaging

GA HA0KM

UT WOS:000449896900008

PM 30266009

OA Green Accepted

DA 2022-08-24

ER

PT J

AU Chen, XG

Sheikh, K

Nakajima, E

Lin, CT

Lee, J

Hu, C

Hales, RK

Forde, PM

Naidoo, J

Voong, KR

AF Chen, Xuguang

Sheikh, Khadija

Nakajima, Erica

Lin, Cheng Ting

Lee, Junghoon

Hu, Chen

Hales, Russell K.

Forde, Patrick M.

Naidoo, Jarushka

Khinh Ranh Voong

TI Radiation Versus Immune Checkpoint Inhibitor Associated Pneumonitis:

Distinct Radiologic Morphologies

SO ONCOLOGIST

LA English

DT Article

DE Immune checkpoint inhibitor; Immune-related adverse event;

Immune-related pneumonitis; Radiation pneumonitis; Non-small cell lung

carcinoma

ID CELL LUNG-CANCER; DEFINITIVE CHEMORADIATION; RADIOGRAPHIC PATTERNS;

RADIOMICS; THERAPY; CT

AB Background Patients with non-small cell lung cancer may develop pneumonitis after thoracic radiotherapy (RT) and immune checkpoint inhibitors (ICIs). We hypothesized that distinct morphologic features are associated with different pneumonitis etiologies. Materials and Methods We systematically compared computed tomography (CT) features of RT- versus ICI-pneumonitis. Clinical and imaging features were tested for association with pneumonitis severity. Lastly, we constructed an exploratory radiomics-based machine learning (ML) model to discern pneumonitis etiology. Results Between 2009 and 2019, 82 patients developed pneumonitis: 29 after thoracic RT, 23 after ICI, and 30 after RT + ICI. Fifty patients had grade 2 pneumonitis, 22 grade 3, and 7 grade 4. ICI-pneumonitis was more likely bilateral (65% vs. 28%; p = .01) and involved more lobes (66% vs. 45% involving at least three lobes) and was less likely to have sharp border (17% vs. 59%; p = .004) compared with RT-pneumonitis. Pneumonitis morphology after RT + ICI was heterogeneous, with 47% bilateral, 37% involving at least three lobes, and 40% sharp borders. Among all patients, risk factors for severe pneumonitis included poor performance status, smoking history, worse lung function, and bilateral and multifocal involvement on CT. An ML model based on seven radiomic features alone could distinguish ICI- from RT-pneumonitis with an area under the receiver-operating curve of 0.76 and identified the predominant etiology after RT + ICI concordant with multidisciplinary consensus. Conclusion RT- and ICI-pneumonitis exhibit distinct spatial features on CT. Bilateral and multifocal lung involvement is associated with severe pneumonitis. Integrating these morphologic features in the clinical management of patients who develop pneumonitis after RT and ICIs may improve treatment decision-making. Implications for Practice Patients with non-small cell lung cancer often receive thoracic radiation and immune checkpoint inhibitors (ICIs), both of which can cause pneumonitis. This study identified similarities and differences in pneumonitis morphology on computed tomography (CT) scans among pneumonitis due to radiotherapy (RT) alone, ICI alone, and the combination of both. Patients who have bilateral CT changes involving at least three lobes are more likely to have ICI-pneumonitis, whereas those with unilateral CT changes with sharp borders are more likely to have radiation pneumonitis. After RT and/or ICI, severe pneumonitis is associated with bilateral and multifocal CT changes. These results can help guide clinicians in triaging patients who develop pneumonitis after radiation and during ICI treatment.

C1 [Chen, Xuguang; Sheikh, Khadija; Lee, Junghoon; Hales, Russell K.; Khinh Ranh Voong] Johns Hopkins Univ, Sch Med, Dept Radiat Oncol & Mol Radiat Sci, Baltimore, MD USA.

[Lin, Cheng Ting] Johns Hopkins Univ, Dept Radiol & Radiol Sci, Baltimore, MD USA.

[Hu, Chen] Johns Hopkins Univ, Dept Biostat, Baltimore, MD 21205 USA.

[Nakajima, Erica; Hu, Chen; Forde, Patrick M.; Naidoo, Jarushka] Johns Hopkins Univ, Dept Oncol, Baltimore, MD USA.

RP Voong, KR (通讯作者)，Sidney Kimmel Canc Ctr, Thorac Ctr Excellence, Dept Radiat Oncol & Mol Radiat Sci, Johns Hopkins Bayview, 300 Mason Lord Dr, Baltimore, MD 21224 USA.

EM kvoong1@jhmi.edu

RI Forde, Patrick/AAX-2936-2021

OI Hu, Chen/0000-0003-4672-1981; Chen, Xuguang/0000-0001-8761-810X; Sheikh,

Khadija/0000-0002-1168-1783

FU Projekt DEAL

FX Open access funding enabled and organized by Projekt DEAL.; Open access

funding enabled and organized by Projekt DEAL.

CR Antonia SJ, 2018, NEW ENGL J MED, V379, P2342, DOI 10.1056/NEJMoa1809697

Antonia SJ, 2017, NEW ENGL J MED, V377, P1919, DOI 10.1056/NEJMoa1709937

Balaji A, 2021, J IMMUNOTHER CANCER, V9, DOI 10.1136/jitc-2020-001731

Barron F, 2020, FRONT ONCOL, V10, DOI 10.3389/fonc.2020.570233

Brzezianska E, 2006, MUTAT RES-FUND MOL M, V599, P26, DOI 10.1016/j.mrfmmm.2005.12.013

Castillo R, 2014, RADIAT ONCOL, V9, DOI 10.1186/1748-717X-9-74

Chaudhuri AA, 2016, RADIOTHER ONCOL, V119, P454, DOI 10.1016/j.radonc.2016.05.007

Chen S, 2019, CANCER IMAGING, V19, DOI 10.1186/s40644-019-0243-3

Choi YW, 2004, RADIOGRAPHICS, V24, P985, DOI 10.1148/rg.244035160

Colen RR, 2018, INVEST NEW DRUG, V36, P601, DOI 10.1007/s10637-017-0524-2

Cousin F, 2021, RADIOTHER ONCOL, V157, P47, DOI 10.1016/j.radonc.2021.01.001

Cunliffe A, 2015, INT J RADIAT ONCOL, V91, P1048, DOI 10.1016/j.ijrobp.2014.11.030

Darnell EP, 2020, CURR ONCOL REP, V22, DOI 10.1007/s11912-020-0897-9

Dolladille C, 2020, JAMA ONCOL, V6, P865, DOI 10.1001/jamaoncol.2020.0726

Ekert K, 2020, CANCERS, V12, DOI 10.3390/cancers12030761

Friedes C, 2020, CLIN LUNG CANCER, V21, pE622, DOI 10.1016/j.cllc.2020.05.013

Gomez DR, 2019, J CLIN ONCOL, V37, DOI 10.1200/JCO.19.00201

Gomez DR, 2016, LANCET ONCOL, V17, P1672, DOI 10.1016/S1470-2045(16)30532-0

Keffer S, 2020, ADV RADIAT ONCOL, V5, P238, DOI 10.1016/j.adro.2019.08.010

Ko EC, 2018, CLIN CANCER RES, V24, P5792, DOI 10.1158/1078-0432.CCR-17-3620

Kocher M, 2020, STRAHLENTHER ONKOL, V196, P856, DOI 10.1007/s00066-020-01626-8

Lambin P, 2017, NAT REV CLIN ONCOL, V14, P749, DOI 10.1038/nrclinonc.2017.141

Li MQ, 2019, BBA-REV CANCER, V1871, P323, DOI 10.1016/j.bbcan.2019.02.004

Lovinfosse P, 2018, EUR J NUCL MED MOL I, V45, P365, DOI 10.1007/s00259-017-3855-5

Luna JM, 2019, RADIOTHER ONCOL, V133, P106, DOI 10.1016/j.radonc.2019.01.003

Moran A, 2017, CLIN LUNG CANCER, V18, pE425, DOI 10.1016/j.cllc.2017.05.014

Naidoo J, 2017, J CLIN ONCOL, V35, P709, DOI 10.1200/JCO.2016.68.2005

Nishino M, 2016, CLIN CANCER RES, V22, P6051, DOI 10.1158/1078-0432.CCR-16-1320

Park H, 2020, EUR J RADIOL, V132, DOI 10.1016/j.ejrad.2020.109275

Park KJ, 2000, RADIOGRAPHICS, V20, P83, DOI 10.1148/radiographics.20.1.g00ja0483

Petit SF, 2011, INT J RADIAT ONCOL, V81, P698, DOI 10.1016/j.ijrobp.2010.06.016

Reuss JE, 2020, CURR ONCOL REP, V22, DOI 10.1007/s11912-020-00920-z

Robnett TJ, 2000, INT J RADIAT ONCOL, V48, P89, DOI 10.1016/S0360-3016(00)00648-9

Sheikh K, 2019, RADIAT ONCOL, V14, DOI 10.1186/s13014-019-1339-4

Suresh K, 2019, J CLIN INVEST, V129, P4305, DOI 10.1172/JCI128654

Thomas R, 2020, LUNG CANCER, V145, P132, DOI 10.1016/j.lungcan.2020.03.023

van Griethuysen JJM, 2017, CANCER RES, V77, pE104, DOI 10.1158/0008-5472.CAN-17-0339

Voong KR, 2020, LUNG CANCER, V150, P249, DOI 10.1016/j.lungcan.2020.08.022

Voong KR, 2019, CLIN LUNG CANCER, V20, pE470, DOI 10.1016/j.cllc.2019.02.018

Yu H, 2019, CLIN CANCER RES, V25, P4343, DOI 10.1158/1078-0432.CCR-18-1084

Yu HW, 2020, FRONT ONCOL, V10, DOI 10.3389/fonc.2020.00412

Yu H, 2009, IEEE T MED IMAGING, V28, P374, DOI 10.1109/TMI.2008.2004425

Yue JB, 2017, CLIN TRANSL RAD ONCO, V4, P1, DOI 10.1016/j.ctro.2017.04.001

NR 43

TC 5

Z9 5

U1 0

U2 4

PU WILEY

PI HOBOKEN

PA 111 RIVER ST, HOBOKEN 07030-5774, NJ USA

SN 1083-7159

EI 1549-490X

J9 ONCOLOGIST

JI Oncologist

PD OCT

PY 2021

VL 26

IS 10

BP E1822

EP E1832

DI 10.1002/onco.13900

EA AUG 2021

PG 11

WC Oncology

WE Science Citation Index Expanded (SCI-EXPANDED)

SC Oncology

GA WA7JB

UT WOS:000680951500001

PM 34251728

OA Green Published, hybrid

DA 2022-08-24

ER

PT J

AU Avanzo, M

Gagliardi, V

Stancanello, J

Blanck, O

Pirrone, G

El Naqa, I

Revelant, A

Sartor, G

AF Avanzo, Michele

Gagliardi, Vito

Stancanello, Joseph

Blanck, Oliver

Pirrone, Giovanni

El Naqa, Issam

Revelant, Alberto

Sartor, Giovanna

TI Combining computed tomography and biologically effective dose in

radiomics and deep learning improves prediction of tumor response to

robotic lung stereotactic body radiation therapy

SO MEDICAL PHYSICS

LA English

DT Article

DE lung cancer; machine learning; radiomics; radiotherapy; SBRT

ID CANCER; INTEROBSERVER; RADIOTHERAPY; VARIABILITY; OUTCOMES; RECIST; PET

AB Purpose The aim of this study is to improve the performance of machine learning (ML) models in predicting response of non-small cell lung cancer (NSCLC) to stereotactic body radiation therapy (SBRT) by integrating image features from pre-treatment computed tomography (CT) with features from the biologically effective dose (BED) distribution. Materials and methods Image features, consisting of crafted radiomic features or machine-learned features extracted using a convolutional neural network, were calculated from pre-treatment CT data and from dose distributions converted into BED for 80 NSCLC lesions over 76 patients treated with robotic guided SBRT. ML models using different combinations of features were trained to predict complete or partial response according to response criteria in solid tumors, including radiomics CT (Rad(CT)), radiomics CT and BED (Rad(CT,BED)), deep learning (DL) CT (DLCT), and DL CT and BED (DLCT,BED). Training of ML included feature selection by neighborhood component analysis followed by ensemble ML using robust boosting. A model was considered as acceptable when the sum of average sensitivity and specificity on test data in repeated cross validations was at least 1.5. Results Complete or partial response occurred in 58 out of 80 lesions. The best models to predict the tumor response were those using BED variables, achieving significantly better area under curve (AUC) and accuracy than those using only features from CT, including a Rad(CT,BED) model using three radiomic features from BED, which scored an accuracy of 0.799 (95% confidence intervals (0.75-0.85)) and AUC of 0.773 (0.688-0.846), and a DLCT,BED model also using three variables with an accuracy of 0.798 (0.649-0.829) and AUC of 0.812 (0.755-0.867). Conclusion According to our results, the inclusion of BED features improves the response prediction of ML models for lung cancer patients undergoing SBRT, regardless of the use of radiomic or DL features.

C1 [Avanzo, Michele; Gagliardi, Vito; Pirrone, Giovanni; Sartor, Giovanna] Ctr Riferimento Oncol Aviano CRO IRCCS, Med Phys Dept, Via F Gallini 2, I-33081 Aviano, PN, Italy.

[Stancanello, Joseph] Elekta SA, Boulogne, France.

[Blanck, Oliver] Univ Med Ctr Schleswig Holstein, Dept Radiat Oncol, Kiel, Germany.

[El Naqa, Issam] Moffitt Univ, Dept Machine Learning, Tampa, FL USA.

[Revelant, Alberto] Ctr Riferimento Oncol Aviano CRO IRCCS, Radiat Oncol Dept, Aviano, PN, Italy.

RP Avanzo, M (通讯作者)，Ctr Riferimento Oncol Aviano CRO IRCCS, Med Phys Dept, Via F Gallini 2, I-33081 Aviano, PN, Italy.

EM mavanzo@cro.it

RI Avanzo, Michele/C-8529-2009; Revelant, Alberto/AAC-4972-2022

OI Avanzo, Michele/0000-0003-1711-4242; Revelant,

Alberto/0000-0002-4505-9650; Blanck, Oliver/0000-0003-1391-1308

FU "5x1000 per la Ricerca Sanitaria" of Centro di Riferimento Oncologico

diAviano (CRO) IRCCS [J32F16001240001]; National Institute of Health

(NIH) [R37-CA222215, R01-CA233487, R41CA243722]; NIBIB

[75N92020D00018/75N92020F0001]

FX The present researchwas in part supported by "5x1000 per la Ricerca

Sanitaria" of Centro di Riferimento Oncologico diAviano (CRO) IRCCS,

Grant Number J32F16001240001 and grants fromNational Institute of Health

(NIH): R37-CA222215 and R01-CA233487,R41CA243722 and NIBIB contract

75N92020D00018/75N92020F0001

CR Avanzo M, 2020, MED PHYS, V47, pE185, DOI 10.1002/mp.13678

Avanzo M, 2020, FRONT ONCOL, V10, DOI 10.3389/fonc.2020.00490

Avanzo M, 2020, STRAHLENTHER ONKOL, V196, P879, DOI 10.1007/s00066-020-01625-9

Avanzo M, 2019, MED PHYS, V46, P1447, DOI 10.1002/mp.13379

Avanzo M, 2017, PHYS MEDICA, V38, P122, DOI 10.1016/j.ejmp.2017.05.071

Baek S, 2019, SCI REP-UK, V9, DOI 10.1038/s41598-019-53461-2

Baumann R, 2018, FRONT ONCOL, V8, DOI 10.3389/fonc.2018.00171

Bousabarah K, 2019, STRAHLENTHER ONKOL, V195, P830, DOI 10.1007/s00066-019-01452-7

Bridle J. S., 1990, Neurocomputing, Algorithms, Architectures and Applications. Proceedings of the NATO Advanced Research Workshop, P227

Ciompi F, 2017, SCI REP-UK, V7, DOI 10.1038/srep46479

Coroller TP, 2016, RADIOTHER ONCOL, V119, P480, DOI 10.1016/j.radonc.2016.04.004

Dissaux G, 2020, J NUCL MED, V61, P814, DOI 10.2967/jnumed.119.228106

Donahue Jeff, 2013, CORR

Dou TH, 2018, PLOS ONE, V13, DOI 10.1371/journal.pone.0206108

El Naqa I, 2018, MED PHYS, V45, pE834, DOI 10.1002/mp.12811

Costa MGF, 2019, BMC MED IMAGING, V19, DOI 10.1186/s12880-019-0389-2

Ferreira JR, 2021, CLIN IMAG, V74, P27, DOI 10.1016/j.clinimag.2020.12.017

Freund Y, 2009, ARXIV09052138

Fried DV, 2016, RADIOLOGY, V278, P214, DOI 10.1148/radiol.2015142920

Garau N, 2020, MED PHYS, V47, P4125, DOI 10.1002/mp.14308

Greengrass E., 2005, 5 IEEE INT C DAT MIN

Grills IS, 2012, J THORAC ONCOL, V7, P1382, DOI 10.1097/JTO.0b013e318260e00d

Guckenberger M, 2014, STRAHLENTHER ONKOL, V190, P26, DOI 10.1007/s00066-013-0450-y

Hawkins SH, 2014, IEEE ACCESS, V2, P1418, DOI 10.1109/ACCESS.2014.2373335

He KM, 2016, PROC CVPR IEEE, P770, DOI 10.1109/CVPR.2016.90

Hosny A, 2018, PLOS MED, V15, DOI 10.1371/journal.pmed.1002711

Hubbard L, 2019, EUR RADIOL EXP, V3, DOI 10.1186/s41747-019-0093-6

Huynh E, 2016, RADIOTHER ONCOL, V120, P258, DOI 10.1016/j.radonc.2016.05.024

Ioffe S, 2015, PR MACH LEARN RES, V37, P448

Kim H, 2021, KNOWL-BASED SYST, V218, DOI 10.1016/j.knosys.2021.106855

Klement RJ, 2020, INT J RADIAT ONCOL, V107, P579, DOI 10.1016/j.ijrobp.2020.03.005

Lafata K, 2018, PHYS MED BIOL, V63, DOI 10.1088/1361-6560/aae56a

Lafata KJ, 2019, PHYS MED BIOL, V64, DOI 10.1088/1361-6560/aaf5a5

Lao JW, 2017, SCI REP-UK, V7, DOI 10.1038/s41598-017-10649-8

Li ZY, 2014, 2014 IEEE INTERNATIONAL SYMPOSIUM ON HAPTIC, AUDIO AND VISUAL ENVIRONMENTS AND GAMES (HAVE)

Liang B, 2019, FRONT ONCOL, V9, DOI 10.3389/fonc.2019.00269

Lundervold AS, 2019, Z MED PHYS, V29, P102, DOI 10.1016/j.zemedi.2018.11.002

Ma Y, 2012, ENSEMBLE MACHINE LEARNING: METHODS AND APPLICATIONS, P1, DOI 10.1007/978-1-4419-9326-7

Mattonen SA, 2016, BRIT J RADIOL, V89, DOI 10.1259/bjr.20160113

Mazzola R, 2019, STRAHLENTHER ONKOL, V195, P719, DOI 10.1007/s00066-018-01419-0

McErlean Aoife, 2013, Radiology, V269, P451, DOI 10.1148/radiol.13122665

Moran A, 2017, CLIN LUNG CANCER, V18, pE425, DOI 10.1016/j.cllc.2017.05.014

Moreno AC, 2020, J THORAC ONCOL, V15, P101, DOI 10.1016/j.jtho.2019.08.2505

Muenzel D, 2012, RADIOL ONCOL, V46, P8, DOI 10.2478/v10019-012-0009-z

Muhlbaier M, 2005, LECT NOTES COMPUT SC, V3541, P326

Ohri N, 2018, PRACT RADIAT ONCOL, V8, pE33, DOI 10.1016/j.prro.2017.10.002

Ohri N, 2012, INT J RADIAT ONCOL, V84, pE379, DOI 10.1016/j.ijrobp.2012.04.040

Oikonomou A, 2018, SCI REP-UK, V8, DOI 10.1038/s41598-018-22357-y

Orlhac F, 2017, J NUCL MED, V58, P387, DOI 10.2967/jnumed.116.181859

Pan YX, 2018, J APPL CLIN MED PHYS, V19, P142, DOI 10.1002/acm2.12314

Parekh V, 2016, EXPERT REV PRECIS ME, V1, P207, DOI 10.1080/23808993.2016.1164013

Parekh VS, 2019, EXPERT REV PRECIS ME, V4, P59, DOI 10.1080/23808993.2019.1585805

Parmar C, 2018, CLIN CANCER RES, V24, P3492, DOI 10.1158/1078-0432.CCR-18-0385

Power Michael, 2013, Evid Based Med, V18, P5, DOI 10.1136/eb-2012-100645

Sankar V., 2019, ARXIV190104641

Shiue K, 2018, J THORAC ONCOL, V13, P1549, DOI 10.1016/j.jtho.2018.06.007

Suzuki C, 2010, ACTA ONCOL, V49, P509, DOI 10.3109/02841861003705794

Temming S, 2018, STRAHLENTHER ONKOL, V194, P91, DOI 10.1007/s00066-017-1194-x

Varma S, 2006, BMC BIOINFORMATICS, V7, DOI 10.1186/1471-2105-7-91

Wang KD, 2021, IEEE T CYBERNETICS, V51, P1556, DOI 10.1109/TCYB.2019.2957101

Welch ML, 2020, PHYS MEDICA, V70, P145, DOI 10.1016/j.ejmp.2020.01.027

Xu YW, 2019, CLIN CANCER RES, V25, P3266, DOI 10.1158/1078-0432.CCR-18-2495

Yamashita R., 2018, INSIGHTS IMAGING, V9, P611, DOI [10.1007/s13244-018-0639-9, DOI 10.1007/s13244-018-0639-9]

Yosinski J., 2015, INT C MACH LEARN WOR

Zhang Lifei, 2015, Med Phys, V42, P1341, DOI 10.1118/1.4908210

Zhang YC, 2017, SCI REP-UK, V7, DOI 10.1038/srep46349

Zwanenburg A, 2016, ARXIV, DOI DOI 10.1148/RADIOL.2020191145

NR 67

TC 7

Z9 7

U1 3

U2 8

PU WILEY

PI HOBOKEN

PA 111 RIVER ST, HOBOKEN 07030-5774, NJ USA

SN 0094-2405

EI 2473-4209

J9 MED PHYS

JI Med. Phys.

PD OCT

PY 2021

VL 48

IS 10

BP 6257

EP 6269

DI 10.1002/mp.15178

EA SEP 2021

PG 13

WC Radiology, Nuclear Medicine & Medical Imaging

WE Science Citation Index Expanded (SCI-EXPANDED)

SC Radiology, Nuclear Medicine & Medical Imaging

GA WR0CC

UT WOS:000693372100001

PM 34415574

DA 2022-08-24

ER

PT J

AU Sun, WB

Niraula, D

El Naqa, I

Ten Haken, RK

Dinov, I

Cuneo, K

Jin, JDY

AF Sun, Wenbo

Niraula, Dipesh

El Naqa, Issam

Ten Haken, Randall K.

Dinov, Ivo

Cuneo, Kyle

Jin, Judy (Jionghua)

TI Precision radiotherapy via information integration of expert human

knowledge and AI recommendation to optimize clinical decision making

SO COMPUTER METHODS AND PROGRAMS IN BIOMEDICINE

LA English

DT Article

DE Precision medicine; Decision making; Artificial intelligence; Computer

model calibration; Gaussian process modeling

ID INDUCIBLE PROTEIN-10; LUNG-CANCER; RADIATION; STATISTICS

AB In the precision medicine era, there is a growing need for precision radiotherapy where the planned radiation dose needs to be optimally determined by considering a myriad of patient-specific information in order to ensure treatment efficacy. Existing artificial-intelligence (AI) methods can recommend radiation dose prescriptions within the scope of this available information. However, treating physicians may not fully entrust the AI's recommended prescriptions due to known limitations or at instances when the AI recommendation may go beyond physicians' current knowledge. This paper lays out a systematic method to integrate expert human knowledge with AI recommendations for optimizing clinical decision making. Towards this goal, Gaussian process (GP) models are integrated with deep neural networks (DNNs) to quantify the uncertainty of the treatment outcomes given by physicians and AI recommendations, respectively, which are further used as a guideline to educate clinical physicians and improve AI models performance. The proposed method is demonstrated in a comprehensive dataset where patient-specific information and treatment outcomes are prospectively collected during radiotherapy of 67 non-small cell lung cancer (NSCLC) patients and are retrospectively analyzed.(c) 2022 Elsevier B.V. All rights reserved.

C1 [Sun, Wenbo; Jin, Judy (Jionghua)] Univ Michigan, Dept Ind & Operat Engn, Ann Arbor, MI 48109 USA.

[Niraula, Dipesh; El Naqa, Issam] H Lee Moffitt Canc Ctr & Res Inst, Dept Machine Learning, Tampa, FL USA.

[Ten Haken, Randall K.; Cuneo, Kyle] Univ Michigan, Dept Radiat Oncol, Ann Arbor, MI USA.

[Dinov, Ivo] Univ Michigan, Dept Computat Med & Bioinformat, Ann Arbor, MI USA.

RP Sun, WB (通讯作者)，Univ Michigan, Dept Ind & Operat Engn, Ann Arbor, MI 48109 USA.

EM sunwbgt@umich.edu; Dipesh.Niraula@moffitt.org; Issam.ElNaqa@moffitt.org;

rth@med.umich.edu; kcuneo@umich.edu; jhjin@umich.edu

RI El Naqa, Issam/T-3066-2019

OI El Naqa, Issam/0000-0001-6023-1132; Niraula, Dipesh/0000-0002-2245-8536

FU NIH [R01-CA233487]

FX The research is partly supported by NIH grant R01-CA233487. The authors

would like to thank Dr. Yi Luo for valuable discussions

CR ANGIOLILLO AL, 1995, J EXP MED, V182, P155, DOI 10.1084/jem.182.1.155

Ashton JR, 2018, THERANOSTICS, V8, P1782, DOI 10.7150/thno.22621

Benedict SH, 2016, INT J RADIAT ONCOL, V95, P873, DOI 10.1016/j.ijrobp.2016.03.006

Bengio Y., 2016, DEEP LEARNING

Carroll Gabriel., 2021, GEN FRAMEWORK ROBUST

Chakraborty B., 2013, STAT METHODS DYNAMIC

Chang JS, 2008, INT J CANCER, V123, P2095, DOI 10.1002/ijc.23801

Cressie N, 2003, STAT SCI, V18, P436, DOI 10.1214/ss/1081443228

Dufour JH, 2002, J IMMUNOL, V168, P3195, DOI 10.4049/jimmunol.168.7.3195

El Naqa I., 2018, GUIDE OUTCOME MODELI

El Naqa I, 2018, JCO CLIN CANCER INFO, V2, DOI 10.1200/CCI.18.00002

Goodfellow I., 2014, ADV NEURAL INFORM PR, P2672

Hildebrandt MAT, 2010, PLOS ONE, V5, DOI 10.1371/journal.pone.0012402

IMDRF SaMD Working Group, 2015, SOFTWARE MEDICAL DEV

IMDRF SaMD Working Group, 2013, SOFTW MED DEV SAMD K

IMDRF Software as a Medical Device (SaMD) Working Group, 2014, SOFTW MED DEV POSS F

Kennedy MC, 2001, J R STAT SOC B, V63, P425, DOI 10.1111/1467-9868.00294

Kiyohara Chikako, 2007, Int J Med Sci, V4, P59

Kong FM, 2017, JAMA ONCOL, V3, P1358, DOI 10.1001/jamaoncol.2017.0982

Laffey JG, 2002, ANESTHESIOLOGY, V97, P215

Luo Y, 2018, MED PHYS, V45, P3980, DOI 10.1002/mp.13029

Luo Y, 2017, RADIOTHER ONCOL, V123, P85, DOI 10.1016/j.radonc.2017.02.004

LUSTER AD, 1985, NATURE, V315, P672, DOI 10.1038/315672a0

Mahasittiwat P, 2013, J RADIOL ONCOL, V2, P191, DOI 10.1007/s13566-013-0091-x

Moodie EEM, 2014, STAT BIOSCI, V6, P223, DOI 10.1007/s12561-013-9103-z

Niraula D, 2021, SCI REP-UK, V11, DOI 10.1038/s41598-021-02910-y

Pearl J, 2009, STAT SURV, V3, P96, DOI 10.1214/09-SS057

Qian M, 2011, ANN STAT, V39, P1180, DOI 10.1214/10-AOS864

Ramirez MF, 2013, ANESTHESIOLOGY, V3, P133, DOI DOI 10.4236/0JANES.2013.33031

Rasmussen CE, 2004, LECT NOTES ARTIF INT, V3176, P63, DOI 10.1007/978-3-540-28650-9_4

Rich AS, 2019, NAT MACH INTELL, V1, P174, DOI 10.1038/s42256-019-0038-z

Schaue D, 2012, RADIAT RES, V178, P505, DOI 10.1667/RR3031.1

Sutton RS, 2018, ADAPT COMPUT MACH LE, P1

TIERNEY L, 1986, J AM STAT ASSOC, V81, P82, DOI 10.2307/2287970

Tseng HH, 2018, FRONT ONCOL, V8, DOI 10.3389/fonc.2018.00266

Tseng HH, 2017, MED PHYS, V44, P6690, DOI 10.1002/mp.12625

U.S. Department of Health and Human Services Food and Drug Administration Center for Devices and Radiological Health, 2017, SOFTW MED DEV SAMD C

USFDA, 2019, PROPOSED REGULATORY

Vallieres M. C., 2018, THESIS MCGILL U LIB

van Hasselt H, 2016, AAAI CONF ARTIF INTE, P2094

Zhao YQ, 2012, J AM STAT ASSOC, V107, P1106, DOI 10.1080/01621459.2012.695674

NR 41

TC 0

Z9 0

U1 0

U2 0

PU ELSEVIER IRELAND LTD

PI CLARE

PA ELSEVIER HOUSE, BROOKVALE PLAZA, EAST PARK SHANNON, CO, CLARE, 00000,

IRELAND

SN 0169-2607

EI 1872-7565

J9 COMPUT METH PROG BIO

JI Comput. Meth. Programs Biomed.

PD JUN

PY 2022

VL 221

AR 106927

DI 10.1016/j.cmpb.2022.106927

PG 10

WC Computer Science, Interdisciplinary Applications; Computer Science,

Theory & Methods; Engineering, Biomedical; Medical Informatics

WE Science Citation Index Expanded (SCI-EXPANDED)

SC Computer Science; Engineering; Medical Informatics

GA 2M5WJ

UT WOS:000817769500001

PM 35675722

OA Green Submitted

DA 2022-08-24

ER

PT J

AU Kerhet, A

Small, C

Quon, H

Riauka, T

Schrader, L

Greiner, R

Yee, D

McEwan, A

Roa, W

AF Kerhet, A.

Small, C.

Quon, H.

Riauka, T.

Schrader, L.

Greiner, R.

Yee, D.

McEwan, A.

Roa, W.

TI Application of machine learning methodology for PET-based definition of

lung cancer

SO CURRENT ONCOLOGY

LA English

DT Article

DE Positron-emission tomography; PET; radiation treatment; lung cancer;

gross tumour volume; GTV; artificial intelligence; machine learning;

support vector machine; SVM

ID TARGET VOLUME DEFINITION; F-18-FDG PET; THRESHOLD SEGMENTATION; IMAGE

SEGMENTATION; FDG-PET; RADIOTHERAPY; DELINEATION; CT

AB We applied a learning methodology framework to assist in the threshold-based segmentation of non-small-cell lung cancer (NSCLC) tumours in positron-emission tomography-computed tomography (PET-CT) imaging for use in radiotherapy planning. Gated and standard free-breathing studies of two patients were independently analysed (four studies in total). Each study had a PET-CT and a treatment-planning CT image. The reference gross tumour volume (GTV) was identified by two experienced radiation oncologists who also determined reference standardized uptake value (SUV) thresholds that most closely approximated the GTV contour on each slice. A set of uptake distribution-related attributes was calculated for each pet slice. A machine learning algorithm was trained on a subset of the pet slices to cope with slice-to-slice variation in the optimal SUV threshold: that is, to predict the most appropriate SUV threshold from the calculated attributes for each slice. The algorithm's performance was evaluated using the remainder of the pet slices. A high degree of geometric similarity was achieved between the areas outlined by the predicted and the reference SUV thresholds (Jac-card index exceeding 0.82). No significant difference was found between the gated and the free-breathing results in the same patient. In this preliminary work, we demonstrated the potential applicability of a machine learning methodology as an auxiliary tool for radiation treatment planning in NSCLC.

C1 [Kerhet, A.; Riauka, T.; McEwan, A.] Univ Alberta, Dept Oncol, Edmonton, AB T6G 1Z2, Canada.

[Small, C.; Quon, H.; Yee, D.; Roa, W.] Cross Canc Inst, Dept Radiat Oncol, Edmonton, AB T6G 1Z2, Canada.

[Riauka, T.] Cross Canc Inst, Dept Med Phys, Edmonton, AB T6G 1Z2, Canada.

[Schrader, L.; McEwan, A.] Cross Canc Inst, Dept Oncol Imaging, Edmonton, AB T6G 1Z2, Canada.

[Greiner, R.] Univ Alberta, Dept Comp Sci, Edmonton, AB T6G 1Z2, Canada.

[Greiner, R.] Alberta Ingenu Ctr Machine Learning, Edmonton, AB, Canada.

RP Kerhet, A (通讯作者)，Univ Alberta, Dept Oncol, 11560 Univ Ave, Edmonton, AB T6G 1Z2, Canada.

EM kerhet@ualberta.ca

RI Greiner, Russell/AAQ-4502-2020

OI Greiner, Russell/0000-0001-8327-934X

FU Alberta Cancer Board; Alberta Cancer Foundation; Natural Sciences and

Engineering Research Council of Canada; Alberta Ingenuity Centre for

Machine Learning

FX This project was made possible by a grant from the Alberta Cancer Board

and the Alberta Cancer Foundation. Russell Greiner was partially funded

by the Natural Sciences and Engineering Research Council of Canada and

the Alberta Ingenuity Centre for Machine Learning.

CR Bailey D.L., 2005, POSITRON EMISSION TO

Black QC, 2004, INT J RADIAT ONCOL, V60, P1272, DOI 10.1016/j.ijrobp.2004.06.254

Canadian Cancer Society/National Cancer Institute of Canada. Canadian Cancer Statistics, 2008, CAN CANC STAT 2008

Daisne JF, 2003, RADIOTHER ONCOL, V69, P247, DOI 10.1016/S0167-8140(03)00270-6

Drever L, 2007, J APPL CLIN MED PHYS, V8, P93, DOI 10.1120/jacmp.v8i2.2367

Drever L, 2007, MED PHYS, V34, P1253, DOI 10.1118/1.2712043

Drever L, 2006, MED PHYS, V33, P1583, DOI 10.1118/1.2198308

El Naqa I, 2007, MED PHYS, V34, P4738, DOI 10.1118/1.2799886

Faria SL, 2008, INT J RADIAT ONCOL, V70, P1035, DOI 10.1016/j.ijrobp.2007.07.2379

Geets X, 2007, EUR J NUCL MED MOL I, V34, P1427, DOI 10.1007/s00259-006-0363-4

Greco C, 2007, LUNG CANCER, V57, P125, DOI 10.1016/j.lungcan.2007.03.020

Gregoire V, 2007, J NUCL MED, V48, p68S

Nestle U, 2005, J NUCL MED, V46, P1342

Nestle U, 2007, EUR J NUCL MED MOL I, V34, P453, DOI 10.1007/s00259-006-0252-x

Nestle U, 2006, RADIOTHER ONCOL, V81, P209, DOI 10.1016/j.radonc.2006.09.011

Parkin DM, 2005, CA-CANCER J CLIN, V55, P74, DOI 10.3322/canjclin.55.2.74

PISANI P, 1993, INT J CANCER, V55, P891, DOI 10.1002/ijc.2910550604

Rembielak A, 2008, ONKOLOGIE, V31, P57, DOI [10.1159/000112207, 10.1159/0000112207]

Ries L, 2002, SEER CANC STAT REV 1

Smola AJ, 2004, STAT COMPUT, V14, P199, DOI 10.1023/B:STCO.0000035301.49549.88

Valk P.E, 2006, POSITRON EMISSION TO

van Baardwijk A, 2006, CANCER TREAT REV, V32, P245, DOI 10.1016/j.ctrv.2006.02.002

Vapnik V., 1999, NATURE STAT LEARNING

WIELER HJ, 2000, PET CLIN ONCOLOGY

Yu HM, 2009, EUR J RADIOL, V72, P104, DOI 10.1016/j.ejrad.2008.06.015

NR 25

TC 14

Z9 14

U1 2

U2 13

PU MULTIMED INC

PI TORONTO

PA 66 MARTIN ST, TORONTO, ON L9T 2R2, CANADA

SN 1198-0052

J9 CURR ONCOL

JI Curr. Oncol.

PY 2010

VL 17

IS 1

BP 41

EP 47

PG 7

WC Oncology

WE Science Citation Index Expanded (SCI-EXPANDED)

SC Oncology

GA V27XB

UT WOS:000208644800003

PM 20179802

OA gold, Green Submitted, Green Published

DA 2022-08-24

ER

PT J

AU Zhang, FL

Wang, QS

Li, HP

AF Zhang, Fuli

Wang, Qiusheng

Li, Haipeng

TI Automatic Segmentation of the Gross Target Volume in Non-Small Cell Lung

Cancer Using a Modified Version of ResNet

SO TECHNOLOGY IN CANCER RESEARCH & TREATMENT

LA English

DT Article

DE deep learning; automatic segmentation; gross target volume; non-small

cell lung cancer; residual convolutional block; convolutional neural

network

ID RADIOTHERAPY; TUMORS; RISK; CT

AB Radiotherapy plays an important role in the treatment of non-small cell lung cancer. Accurate segmentation of the gross target volume is very important for successful radiotherapy delivery. Deep learning techniques can obtain fast and accurate segmentation, which is independent of experts' experience and saves time compared with manual delineation. In this paper, we introduce a modified version of ResNet and apply it to segment the gross target volume in computed tomography images of patients with non-small cell lung cancer. Normalization was applied to reduce the differences among images and data augmentation techniques were employed to further enrich the data of the training set. Two different residual convolutional blocks were used to efficiently extract the deep features of the computed tomography images, and the features from all levels of the ResNet were merged into a single output. This simple design achieved a fusion of deep semantic features and shallow appearance features to generate dense pixel outputs. The test loss tended to be stable after 50 training epochs, and the segmentation took 21 ms per computed tomography image. The average evaluation metrics were: Dice similarity coefficient, 0.73; Jaccard similarity coefficient, 0.68; true positive rate, 0.71; and false positive rate, 0.0012. Those results were better than those of U-Net, which was used as a benchmark. The modified ResNet directly extracted multi-scale context features from original input images. Thus, the proposed automatic segmentation method can quickly segment the gross target volume in non-small cell lung cancer cases and be applied to improve consistency in contouring.

C1 [Zhang, Fuli] Chinese Peoples Liberat Army Gen Hosp, Dept Radiat Oncol, Med Ctr 7, Beijing 100700, Peoples R China.

[Wang, Qiusheng; Li, Haipeng] Beihang Univ, Sch Automat Sci & Elect Engn, Beijing, Peoples R China.

RP Zhang, FL (通讯作者)，Chinese Peoples Liberat Army Gen Hosp, Dept Radiat Oncol, Med Ctr 7, Beijing 100700, Peoples R China.

EM radiozfli@163.com

OI Zhang, FL/0000-0002-7213-9031

FU Beijing Municipal Science and Technology Commission

FX The study was supported by Beijing Municipal Science and Technology

Commission (No.Z181100001718011). The author(s) received no financial

support for the research, authorship, and/or publication of this

article.

CR Barkati M, 2016, J MED IMAG RADIAT ON, V60, P255, DOI 10.1111/1754-9485.12416

Bauer S, 2011, LECT NOTES COMPUT SC, V6893, P354, DOI 10.1007/978-3-642-23626-6_44

Bi N, 2019, FRONT ONCOL, V9, DOI 10.3389/fonc.2019.01192

Bottou Leon, 2012, Neural Networks: Tricks of the Trade. Second Edition: LNCS 7700, P421, DOI 10.1007/978-3-642-35289-8_25

Ferlay J, 2015, INT J CANCER, V136, pE359, DOI 10.1002/ijc.29210

Iqbal S, 2018, MICROSC RES TECHNIQ, V81, P419, DOI 10.1002/jemt.22994

Janardhanaprabhu S, 2019, J MED SYST, V43, DOI 10.1007/s10916-019-1366-6

Jian JM, 2018, AUSTRALAS PHYS ENG S, V41, P393, DOI 10.1007/s13246-018-0636-9

Jiang J, 2018, LECT NOTES COMPUT SC, V11071, P777, DOI 10.1007/978-3-030-00934-2_86

Jiang J, 2019, IEEE T MED IMAGING, V38, P134, DOI 10.1109/TMI.2018.2857800

Kaiming He, 2016, 2016 IEEE Conference on Computer Vision and Pattern Recognition (CVPR), P770, DOI 10.1109/CVPR.2016.90

Kingma D, 2014, ARXIV

Kirillov A, 2019, PROC CVPR IEEE, P6392, DOI 10.1109/CVPR.2019.00656

Krizhevsky A, 2017, COMMUN ACM, V60, P84, DOI 10.1145/3065386

Lin L, 2019, RADIOLOGY, V291, P677, DOI 10.1148/radiol.2019182012

Lin Tsung-Yi, 2020, IEEE Trans Pattern Anal Mach Intell, V42, P318, DOI [10.1109/TPAMI.2018.2858826, 10.1109/ICCV.2017.324]

Lin TY, 2017, PROC CVPR IEEE, P936, DOI 10.1109/CVPR.2017.106

Liu HH, 2007, INT J RADIAT ONCOL, V68, P531, DOI 10.1016/j.ijrobp.2006.12.066

Liu Y, 2017, PLOS ONE, V12, DOI 10.1371/journal.pone.0185844

Long J, 2015, PROC CVPR IEEE, P3431, DOI 10.1109/CVPR.2015.7298965

Men K, 2018, PHYS MED BIOL, V63, DOI 10.1088/1361-6560/aada6c

Men K, 2018, PHYS MEDICA, V50, P13, DOI 10.1016/j.ejmp.2018.05.006

Men K, 2017, MED PHYS, V44, P6377, DOI 10.1002/mp.12602

Nakai H, 2020, ACAD RADIOL, V27, P563, DOI 10.1016/j.acra.2019.05.016

Razzak MI, 2019, IEEE J BIOMED HEALTH, V23, P1911, DOI 10.1109/JBHI.2018.2874033

Ronneberger O, 2015, LECT NOTES COMPUT SC, V9351, P234, DOI 10.1007/978-3-319-24574-4_28

Siegel RL., 2020, CA-CANCER J CLIN, V70, P7, DOI [DOI 10.3322/caac.21551, 10.3322/caac.21590]

Sudre CH, 2017, LECT NOTES COMPUT SC, V10553, P240, DOI 10.1007/978-3-319-67558-9_28

Tang W, 2019, CHINESE J CANCER RES, V31, P316, DOI 10.21147/j.issn.1000-9604.2019.02.06

Thillaikkarasi R, 2019, J MED SYST, V43, DOI 10.1007/s10916-019-1223-7

Trebeschi S, 2017, SCI REP-UK, V7, DOI 10.1038/s41598-017-05728-9

van Mourik AM, 2010, RADIOTHER ONCOL, V94, P286, DOI 10.1016/j.radonc.2010.01.009

Wang C, 2019, RADIOTHER ONCOL, V131, P101, DOI 10.1016/j.radonc.2018.10.037

Yu F., 2016, ICLR

Zhao XM, 2019, PHYS MED BIOL, V64, DOI 10.1088/1361-6560/aaf44b

Zhong ZS, 2019, MED PHYS, V46, P619, DOI 10.1002/mp.13331

NR 36

TC 8

Z9 8

U1 7

U2 17

PU SAGE PUBLICATIONS INC

PI THOUSAND OAKS

PA 2455 TELLER RD, THOUSAND OAKS, CA 91320 USA

SN 1533-0346

EI 1533-0338

J9 TECHNOL CANCER RES T

JI Technol. Cancer Res. Treat.

PD AUG 14

PY 2020

VL 19

AR 1533033820947484

DI 10.1177/1533033820947484

PG 9

WC Oncology

WE Science Citation Index Expanded (SCI-EXPANDED)

SC Oncology

GA NF5PF

UT WOS:000563348100001

OA gold, Green Published

DA 2022-08-24

ER

PT J

AU Luna, JM

Chao, HH

Diffenderfer, ES

Valdes, G

Chinniah, C

Ma, G

Cengel, KA

Solberg, TD

Berman, AT

Simone, CB

AF Luna, Jose Marcio

Chao, Hann-Hsiang

Diffenderfer, Eric S.

Valdes, Gilmer

Chinniah, Chidambaram

Ma, Grace

Cengel, Keith A.

Solberg, Timothy D.

Berman, Abigail T.

Simone, Charles B., II

TI Predicting radiation pneumonitis in locally advanced stage II-III

non-small cell lung cancer using machine learning

SO RADIOTHERAPY AND ONCOLOGY

LA English

DT Article

DE Radiation pneumonitis; Non-small cell lung cancer; Machine learning;

Random forest; RUSBoost; CART; Support vector machines; Logistic

regression

ID VOLUME HISTOGRAM PARAMETERS; PULMONARY TOXICITY; THERAPY; RADIOTHERAPY;

OUTCOMES

AB Background and purpose: Radiation pneumonitis (RP) is a radiotherapy dose-limiting toxicity for locally advanced non-small cell lung cancer (LA-NSCLC). Prior studies have proposed relevant dosimetric constraints to limit this toxicity. Using machine learning algorithms, we performed analyses of contributing factors in the development of RP to uncover previously unidentified criteria and elucidate the relative importance of individual factors.

Materials and methods: We evaluated 32 clinical features per patient in a cohort of 203 stage II-III LANSCLC patients treated with definitive chemoradiation to a median dose of 66.6 Gy in 1.8 Gy daily fractions at our institution from 2008 to 2016. Of this cohort, 17.7% of patients developed grade >= 2 RP. Univariate analysis was performed using trained decision stumps to individually analyze statistically significant predictors of RP and perform feature selection. Applying Random Forest, we performed multivariate analysis to assess the combined performance of important predictors of RP.

Results: On univariate analysis, lung V20, lung mean, lung V10 and lung V5 were found to be significant RP predictors with the greatest balance of specificity and sensitivity. On multivariate analysis, Random Forest (AUC = 0.66, p = 0.0005) identified esophagus max (20.5%), lung V20 (16.4%), lung mean (15.7%) and pack-year (14.9%) as the most common primary differentiators of RP.

Conclusions: We highlight Random Forest as an accurate machine learning method to identify known and new predictors of symptomatic RP. Furthermore, this analysis confirms the importance of lung V20, lung mean and pack-year as predictors of RP while also introducing esophagus max as an important RP predictor. (C) 2019 Elsevier B.V. All rights reserved.

C1 [Luna, Jose Marcio; Chao, Hann-Hsiang; Diffenderfer, Eric S.; Ma, Grace; Cengel, Keith A.; Berman, Abigail T.] Univ Penn, Dept Radiat Oncol, 3400 Civ Ctr Blvd,TRC 8-130, Philadelphia, PA 19104 USA.

[Valdes, Gilmer; Solberg, Timothy D.] Univ Calif San Francisco, Dept Radiat Oncol, San Francisco, CA USA.

[Chinniah, Chidambaram] Albany Med Coll, Albany, NY 12208 USA.

[Simone, Charles B., II] Univ Maryland, Sch Med, Dept Radiat Oncol, Baltimore, MD 21201 USA.

RP Luna, JM (通讯作者)，Univ Penn, Dept Radiat Oncol, 3400 Civ Ctr Blvd,TRC 8-130, Philadelphia, PA 19104 USA.

EM Jose.Luna@uphs.upenn.edu

RI Luna, Jose Marcio/ABG-1296-2020

OI Simone, Charles/0000-0002-0867-3694; Luna, Jose/0000-0002-5513-022X; ,

Timothy/0000-0001-8829-7774

FU Abramson Cancer Center of the University of Pennsylvania

FX This work was partially supported by the Abramson Cancer Center of the

University of Pennsylvania through award granted by the Emerson

Collective.

CR [Anonymous], [No title captured]

Barriger RB, 2010, INT J RADIAT ONCOL, V78, P1381, DOI 10.1016/j.ijrobp.2009.09.030

Bentzen SM, 2010, INT J RADIAT ONCOL, V76, pS3, DOI 10.1016/j.ijrobp.2009.09.040

Bledsoe TJ, 2017, CLIN CHEST MED, V38, P201, DOI 10.1016/j.ccm.2016.12.004

Bradley JD, 2007, INT J RADIAT ONCOL, V69, P985, DOI 10.1016/j.ijrobp.2007.04.077

Collins GS, 2015, CIRCULATION, V131, P211, DOI [10.1161/CIRCULATIONAHA.114.014508, 10.7326/M14-0697, 10.1016/j.jclinepi.2014.11.010, 10.1186/s12916-014-0241-z, 10.1136/bmj.g7594, 10.1002/bjs.9736]

CORTES C, 1995, MACH LEARN, V20, P273, DOI 10.1023/A:1022627411411

COX DR, 1958, J R STAT SOC B, V20, P215

Dang J, 2014, LUNG CANCER, V86, P329, DOI 10.1016/j.lungcan.2014.10.005

Dang J, 2014, RADIAT ONCOL, V9, DOI 10.1186/1748-717X-9-172

Dang J, 2013, ACTA ONCOL, V52, P1175, DOI 10.3109/0284186X.2012.747696

Darcy AM, 2016, JAMA-J AM MED ASSOC, V315, P551, DOI 10.1001/jama.2015.18421

El Naqa I, 2009, PHYS MED BIOL, V54, pS9, DOI 10.1088/0031-9155/54/18/S02

Friedman J.H., 1984, CLASSIFICATION REGRE, P368, DOI DOI 10.1201/9781315139470-8

Graham MV, 1999, INT J RADIAT ONCOL, V45, P323, DOI 10.1016/S0360-3016(99)00183-2

Graves PR, 2010, SEMIN RADIAT ONCOL, V20, P201, DOI 10.1016/j.semradonc.2010.01.010

Hernando ML, 2001, INT J RADIAT ONCOL, V51, P650, DOI 10.1016/S0360-3016(01)01685-6

Kang J, 2015, INT J RADIAT ONCOL, V93, P1127, DOI 10.1016/j.ijrobp.2015.07.2286

Kim M, 2011, RADIAT ONCOL J, V29, P181, DOI 10.3857/roj.2011.29.3.181

Kocak Z, 2007, INT J RADIAT ONCOL, V67, P178, DOI 10.1016/j.ijrobp.2006.09.031

Kolda TG, 2003, SIAM REV, V45, P385, DOI 10.1137/S0036144502428893

Lipton Z, 2016, ICML WORKSH HUM INT, P1

Luna J. M., 2017, NIPS, P1

Luo Y, 2017, RADIOTHER ONCOL, V123, P85, DOI 10.1016/j.radonc.2017.02.004

Marks LB, 2010, INT J RADIAT ONCOL, V76, pS70, DOI 10.1016/j.ijrobp.2009.06.091

Marks LB, 1997, INT J RADIAT ONCOL, V39, P563, DOI 10.1016/S0360-3016(97)00343-X

MathWorks, 2015, MATLAB, V118

MCDONALD S, 1995, INT J RADIAT ONCOL, V31, P1187, DOI 10.1016/0360-3016(94)00429-O

Palma DA, 2013, INT J RADIAT ONCOL, V85, P444, DOI 10.1016/j.ijrobp.2012.04.043

Parashar B, 2011, AM J CLIN ONCOL-CANC, V34, P160, DOI 10.1097/COC.0b013e3181d6b40f

Park YH, 2013, RADIAT ONCOL J, V31, P34, DOI 10.3857/roj.2013.31.1.34

Ramella S, 2010, INT J RADIAT ONCOL, V76, P110, DOI 10.1016/j.ijrobp.2009.01.036

Rancati T, 2003, RADIOTHER ONCOL, V67, P275, DOI 10.1016/S0167-8140(03)00119-1

Rodrigues G, 2004, RADIOTHER ONCOL, V71, P127, DOI 10.1016/j.radonc.2004.02.015

Seiffert C, 2010, IEEE T SYST MAN CY A, V40, P185, DOI 10.1109/TSMCA.2009.2029559

Simone CB, 2017, SEMIN RADIAT ONCOL, V27, P370, DOI 10.1016/j.semradonc.2017.04.009

Svetnik V, 2003, J CHEM INF COMP SCI, V43, P1947, DOI 10.1021/ci034160g

Valdes G, 2016, SCI REP-UK, V6, DOI 10.1038/srep37854

Valdes G, 2016, PHYS MED BIOL, V61, P6105, DOI 10.1088/0031-9155/61/16/6105

Wang DQ, 2013, CLIN TRANSL ONCOL, V15, P364, DOI 10.1007/s12094-012-0931-y

NR 40

TC 32

Z9 34

U1 0

U2 9

PU ELSEVIER IRELAND LTD

PI CLARE

PA ELSEVIER HOUSE, BROOKVALE PLAZA, EAST PARK SHANNON, CO, CLARE, 00000,

IRELAND

SN 0167-8140

EI 1879-0887

J9 RADIOTHER ONCOL

JI Radiother. Oncol.

PD APR

PY 2019

VL 133

BP 106

EP 112

DI 10.1016/j.radonc.2019.01.003

PG 7

WC Oncology; Radiology, Nuclear Medicine & Medical Imaging

WE Science Citation Index Expanded (SCI-EXPANDED)

SC Oncology; Radiology, Nuclear Medicine & Medical Imaging

GA HQ9QK

UT WOS:000462762200016

PM 30935565

DA 2022-08-24

ER

PT J

AU Zhang, T

Yang, Y

Wang, JB

Men, K

Wang, X

Deng, L

Bi, N

AF Zhang, Tao

Yang, Yin

Wang, Jingbo

Men, Kuo

Wang, Xin

Deng, Lei

Bi, Nan

TI Comparison between atlas and convolutional neural network based

automatic segmentation of multiple organs at risk in non-small cell lung

cancer

SO MEDICINE

LA English

DT Article

DE automatic segmentation based on atlas; automatic segmentation based on

convolutional neural network; non-small cell lung cancer; organs at

risk; postoperative radiation therapy

ID CLINICAL TARGET VOLUME; RADIATION-THERAPY; STAGE-II; RADIOTHERAPY;

IMAGES; HEAD; CT

AB Delineation of organs at risk (OARs) is important but time consuming for radiotherapy planning. Automatic segmentation of OARs based on convolutional neural network (CNN) has been established for lung cancer patients at our institution. The aim of this study is to compare automatic segmentation based on CNN (AS-CNN) with automatic segmentation based on atlas (AS-Atlas) in terms of the efficiency and accuracy of OARs contouring. The OARs, including the lungs, esophagus, heart, liver, and spinal cord, of 19 non-small cell lung cancer patients were delineated using three methods: AS-CNN, AS-Atlas in the Pinnacle(3)-software, and manual delineation (MD) by a senior radiation oncologist. MD was used as the ground-truth reference, and the segmentation efficiency was evaluated by the time spent per patient. The accuracy was evaluated using the Mean surface distance (MSD) and Dice similarity coefficient (DSC). The paired t-test or Wilcoxon signed-rank test was used to compare these indexes between the 2 automatic segmentation models. In the 19 testing cases, both AS-CNN and AS-Atlas saved substantial time compared with MD. AS-CNN was more efficient than AS-Atlas (1.6 min vs 2.4 min,P < .001). In terms of the accuracy, AS-CNN performed well in the esophagus, with a DSC of 73.2%. AS-CNN was better than AS-Atlas in segmenting the left lung (DSC: 94.8% vs 93.2%,P = .01; MSD: 1.10 cm vs 1.73 cm,P < .001) and heart (DSC: 89.3% vs 85.8%,P = .05; MSD: 1.65 cm vs 3.66 cm,P < .001). Furthermore, AS-CNN exhibited superior performance in segmenting the liver (DSC: 93.7% vs 93.6%,P = .81; MSD: 2.03 cm VS 2.11 cm,P = .66). The results obtained from AS-CNN and AS-Atlas were similar in segmenting the right lung. However, the performance of AS-CNN in the spinal cord was inferior to that of AS-Atlas (DSC: 82.1% vs 86.8%,P = .01; MSD: 0.87 cm vs 0.66 cm,P = .01). Our study demonstrated that AS-CNN significantly reduced the contouring time and outperformed AS-Atlas in most cases. AS-CNN can potentially be used for OARs segmentation in patients with pathological N2 (pN2) non-small cell lung cancer.

C1 [Zhang, Tao; Yang, Yin; Wang, Jingbo; Men, Kuo; Wang, Xin; Deng, Lei; Bi, Nan] Chinese Acad Med Sci & Peking Union Med Coll, Canc Hosp, Natl Clin Res Ctr Canc, Dept Radiat Oncol,Natl Canc Ctr, 17 PanjiayuanNanli, Beijing 100021, Peoples R China.

RP Bi, N (通讯作者)，Chinese Acad Med Sci & Peking Union Med Coll, Canc Hosp, Natl Clin Res Ctr Canc, Dept Radiat Oncol,Natl Canc Ctr, 17 PanjiayuanNanli, Beijing 100021, Peoples R China.

EM binan_email@163.com

FU CAMS Innovation Fund for Medical Sciences [2017-I2M-1-009]; Beijing

Municipal Science AMP; Technology Commission [Z181100001918002]

FX This study was supported by grants of CAMS Innovation Fund for Medical

Sciences (No. 2017-I2M-1-009) and the Beijing Municipal Science &

Technology Commission (No. Z181100001918002)

CR Ayyalusamy A, 2019, RADIAT ONCOL J, V37, P134, DOI 10.3857/roj.2019.00038

Bi N, 2019, FRONT ONCOL, V9, DOI 10.3389/fonc.2019.01192

Bray F, 2018, CA-CANCER J CLIN, V68, P394, DOI 10.3322/caac.21492

Chang JY, 2017, JAMA ONCOL, V3, DOI 10.1001/jamaoncol.2017.2032

Delpon G, 2016, FRONT ONCOL, V6, DOI 10.3389/fonc.2016.00178

Douillard JY, 2008, INT J RADIAT ONCOL, V72, P695, DOI 10.1016/j.ijrobp.2008.01.044

Ibragimov B, 2017, PHYS MED BIOL, V62, P8943, DOI 10.1088/1361-6560/aa9262

Ibragimov B, 2017, MED PHYS, V44, P547, DOI 10.1002/mp.12045

Kilburn JM, 2016, PRACT RADIAT ONCOL, V6, pE73, DOI 10.1016/j.prro.2015.10.004

Lally BE, 2006, J CLIN ONCOL, V24, P2998, DOI 10.1200/JCO.2005.04.6110

Lee H, 2019, FRONT ONCOL, V9, DOI 10.3389/fonc.2019.00239

Li XA, 2009, INT J RADIAT ONCOL, V73, P944, DOI 10.1016/j.ijrobp.2008.10.034

Liao ZXX, 2010, INT J RADIAT ONCOL, V76, P775, DOI 10.1016/j.ijrobp.2009.02.032

Men K, 2018, PHYS MEDICA, V50, P13, DOI 10.1016/j.ejmp.2018.05.006

Men K, 2017, FRONT ONCOL, V7, DOI 10.3389/fonc.2017.00315

Men K, 2017, MED PHYS, V44, P6377, DOI 10.1002/mp.12602

National Comprehensive Cancer Network, 2020, NON SMALL CELL LUNG

Schreibmann E, 2014, J APPL CLIN MED PHYS, V15, P4468, DOI DOI 10.1120/JACMP.V15I4.4468

Wang SW, 2017, RADIAT ONCOL, V12, DOI 10.1186/s13014-017-0946-1

Wardman K, 2016, J APPL CLIN MED PHYS, V17, P146, DOI 10.1120/jacmp.v17i4.6051

Wennstig AK, 2017, RADIOTHER ONCOL, V122, P72, DOI 10.1016/j.radonc.2016.11.007

Wittenstein O, 2019, STRAHLENTHER ONKOL, V195, P1094, DOI 10.1007/s00066-019-01463-4

Xu YJ, 2017, SCI REP-UK, V7, DOI 10.1038/s41598-017-14629-w

Zhu JH, 2019, ACTA ONCOL, V58, P257, DOI 10.1080/0284186X.2018.1529421

ZIJDENBOS AP, 1994, IEEE T MED IMAGING, V13, P716, DOI 10.1109/42.363096

NR 25

TC 8

Z9 9

U1 3

U2 9

PU LIPPINCOTT WILLIAMS & WILKINS

PI PHILADELPHIA

PA TWO COMMERCE SQ, 2001 MARKET ST, PHILADELPHIA, PA 19103 USA

SN 0025-7974

EI 1536-5964

J9 MEDICINE

JI Medicine (Baltimore)

PD AUG 21

PY 2020

VL 99

IS 34

AR e21800

DI 10.1097/MD.0000000000021800

PG 6

WC Medicine, General & Internal

WE Science Citation Index Expanded (SCI-EXPANDED)

SC General & Internal Medicine

GA OC9DS

UT WOS:000579455900070

PM 32846816

OA gold, Green Published

DA 2022-08-24

ER

PT J

AU Luna, JM

Chao, HH

Shinohara, RT

Ungar, LH

Cengel, KA

Pryma, DA

Chinniah, C

Berman, AT

Katz, SI

Kontos, D

Simone, CB

Diffenderfer, ES

AF Luna, Jose Marcio

Chao, Hann-Hsiang

Shinohara, Russel T.

Ungar, Lyle H.

Cengel, Keith A.

Pryma, Daniel A.

Chinniah, Chidambaram

Berman, Abigail T.

Katz, Sharyn, I

Kontos, Despina

Simone, Charles B., II

Diffenderfer, Eric S.

TI Machine learning highlights the deficiency of conventional dosimetric

constraints for prevention of high-grade radiation esophagitis in

non-small cell lung cancer treated with chemoradiation

SO CLINICAL AND TRANSLATIONAL RADIATION ONCOLOGY

LA English

DT Article

DE Radiation esophagitis; Machine learning; Non-small cell lung cancer;

Chemoradiation; Radiation-induced toxicity; Intensity-modulated

radiation therapy; Proton beam therapy

ID CONCURRENT CHEMORADIATION; PREDICTORS; THERAPY; RADIOTHERAPY; TOXICITY;

CHEMOTHERAPY; CARCINOMA; MODEL; TIME

AB Background and Purpose: Radiation esophagitis is a clinically important toxicity seen with treatment for locally-advanced non-small cell lung cancer. There is considerable disagreement among prior studies in identifying predictors of radiation esophagitis. We apply machine learning algorithms to identify factors contributing to the development of radiation esophagitis to uncover previously unidentified criteria and more robust dosimetric factors.

Materials and Methods: We used machine learning approaches to identify predictors of grade >= 3 radiation esophagitis in a cohort of 202 consecutive locally-advanced non-small cell lung cancer patients treated with definitive chemoradiation from 2008 to 2016. We evaluated 35 clinical features per patient grouped into risk factors, comorbidities, imaging, stage, histology, radiotherapy, chemotherapy and dosimetry. Univariate and multivariate analyses were performed using a panel of 11 machine learning algorithms combined with predictive power assessments.

Results: All patients were treated to a median dose of 66.6 Gy at 1.8 Gy per fraction using photon (89.6%) and proton (10.4%) beam therapy, most often with concurrent chemotherapy (86.6%). 11.4% of patients developed grade >= 3 radiation esophagitis. On univariate analysis, no individual feature was found to predict radiation esophagitis (AUC range 0.45-0.55, p >= 0.07). In multivariate analysis, all machine learning algorithms exhibited poor predictive performance (AUC range 0.46-0.56, p >= 0.07).

Conclusions: Contemporary machine learning algorithms applied to our modern, relatively large institutional cohort could not identify any reliable predictors of grade >= 3 radiation esophagitis. Additional patients are needed, and novel patient-specific and treatment characteristics should be investigated to develop clinically meaningful methods to mitigate this survival altering toxicity. (C) 2020 The Author(s). Published by Elsevier B.V. on behalf of European Society for Radiotherapy and Oncology.

C1 [Luna, Jose Marcio; Cengel, Keith A.; Berman, Abigail T.; Diffenderfer, Eric S.] Univ Penn, Perelman Ctr Adv Med, Dept Radiat Oncol, 3400 Civ Ctr Blvd, Philadelphia, PA 19104 USA.

[Chao, Hann-Hsiang] Hunter Holmes McGuire Vet Affairs Med Ctr, Dept Radiat Oncol, 1201 Broad Rock Blvd, Richmond, VA 23249 USA.

[Shinohara, Russel T.] Univ Penn, Dept Biostat & Epidemiol, 423 Guardian Dr, Philadelphia, PA 19104 USA.

[Ungar, Lyle H.] Univ Penn, Dept Comp & Informat Sci, 3330 Walnut St, Philadelphia, PA 19104 USA.

[Pryma, Daniel A.; Katz, Sharyn, I; Kontos, Despina] Univ Penn, Dept Radiol, 3400 Spruce St, Philadelphia, PA 19104 USA.

[Chinniah, Chidambaram] Albany Med Coll, 43 New Scotland Ave, Albany, NY 12208 USA.

[Simone, Charles B., II] New York Proton Ctr, Dept Radiat Oncol, 225 East 126th St, New York, NY 10035 USA.

RP Luna, JM (通讯作者)，Bldg 421,SCTR 8-130,3400 Civ Ctr Blvd, Philadelphia, PA 19104 USA.

EM jose.luna@pennmedicine.upenn.edu

RI Katz, Sharyn/AAK-1408-2020; Luna, Jose Marcio/ABG-1296-2020

OI Katz, Sharyn/0000-0001-9816-2291; Luna, Jose/0000-0002-5513-022X

FU Emerson Collective Research Fund

FX This work was partially supported by an award granted by the Emerson

Collective Research Fund. Special thanks to Grace Ma for her assistance

extracting clinical information for the cohort.

CR Ahn SJ, 2005, INT J RADIAT ONCOL, V61, P335, DOI 10.1016/j.ijrobp.2004.06.014

Altman DG, 2014, CLIN CHEM, V60, P580, DOI 10.1373/clinchem.2013.220335

[Anonymous], [No title captured]

Auperin A, 2010, J CLIN ONCOL, V28, P2181, DOI 10.1200/JCO.2009.26.2543

Bahn E, 2020, RADIOTHER ONCOL, V144, P148, DOI 10.1016/j.radonc.2019.11.018

Belderbos J, 2005, RADIOTHER ONCOL, V75, P157, DOI 10.1016/j.radonc.2005.03.021

Bradley J, 2004, SEMIN RADIAT ONCOL, V14, P280, DOI 10.1016/j.semradonc.2004.06.003

Bradley J, 2004, INT J RADIAT ONCOL, V58, P1106, DOI 10.1016/j.ijrobp.2003.09.080

Bradley JD, 2015, LANCET ONCOL, V16, P187, DOI 10.1016/S1470-2045(14)71207-0

Chajon E, 2015, BRIT J RADIOL, V88, DOI 10.1259/bjr.20150311

Chao HH, 2018, J APPL CLIN MED PHYS, V19, P539, DOI 10.1002/acm2.12415

Chapet O, 2005, RADIOTHER ONCOL, V77, P176, DOI 10.1016/j.radonc.2005.10.001

Chawla NV, 2002, J ARTIF INTELL RES, V16, P321, DOI 10.1613/jair.953

Collins GS, 2015, CIRCULATION, V131, P211, DOI [10.1161/CIRCULATIONAHA.114.014508, 10.7326/M14-0697, 10.1016/j.jclinepi.2014.11.010, 10.1186/s12916-014-0241-z, 10.1136/bmj.g7594, 10.1002/bjs.9736]

CORTES C, 1995, MACH LEARN, V20, P273, DOI 10.1023/A:1022627411411

COX DR, 1958, J R STAT SOC B, V20, P215

Curran WJ, 2011, J NATL CANCER I, V103, P1452, DOI 10.1093/jnci/djr325

Duda R.O., 2006, PATTERN CLASSIFICATI

Folch-Fortuny A, 2015, CHEMOMETR INTELL LAB, V146, P77, DOI 10.1016/j.chemolab.2015.05.006

Giaddui T, 2016, RADIAT ONCOL, V11, DOI 10.1186/s13014-016-0640-8

Gomez DR, 2012, INT J RADIAT ONCOL, V84, P1010, DOI 10.1016/j.ijrobp.2012.01.071

Hawkins Peter G, 2018, Transl Oncol, V11, P102, DOI 10.1016/j.tranon.2017.11.005

Huang EX, 2017, ADV RADIAT ONCOL, V2, P37, DOI 10.1016/j.adro.2016.11.003

Luna JM, 2019, RADIOTHER ONCOL, V133, P106, DOI 10.1016/j.radonc.2019.01.003

Machtay M, 2005, INT J RADIAT ONCOL, V63, P667, DOI 10.1016/j.ijrobp.2005.03.037

Maguire PD, 1999, INT J RADIAT ONCOL, V45, P97, DOI 10.1016/S0360-3016(99)00163-7

Manapov F, 2013, RADIAT ONCOL, V8, DOI 10.1186/1748-717X-8-122

Mehmood Q, 2016, J THORAC ONCOL, V11, P213, DOI 10.1016/j.jtho.2015.10.006

Movsas B, 2016, JAMA ONCOL, V2, P359, DOI 10.1001/jamaoncol.2015.3969

Palma DA, 2013, INT J RADIAT ONCOL, V87, P690, DOI 10.1016/j.ijrobp.2013.07.029

Patel AB, 2004, INT J RADIAT ONCOL, V60, P1106, DOI 10.1016/j.ijrobp.2004.04.051

PRAAGMAN J, 1985, EUR J OPER RES, V19, P144, DOI 10.1016/0377-2217(85)90321-2

Rose J, 2009, RADIOTHER ONCOL, V91, P282, DOI 10.1016/j.radonc.2008.09.010

Rwigema JCM, 2017, CANCER-AM CANCER SOC, V123, P4244, DOI 10.1002/cncr.30870

Seiffert C, 2010, IEEE T SYST MAN CY A, V40, P185, DOI 10.1109/TSMCA.2009.2029559

Simone CB, 2017, SEMIN RADIAT ONCOL, V27, P370, DOI 10.1016/j.semradonc.2017.04.009

Singh AK, 2003, INT J RADIAT ONCOL, V55, P337, DOI 10.1016/S0360-3016(02)03937-8

Svetnik V, 2003, J CHEM INF COMP SCI, V43, P1947, DOI 10.1021/ci034160g

Thor M, 2019, RADIOTHER ONCOL, V138, P45, DOI 10.1016/j.radonc.2019.05.011

Wada K, 2019, ANTICANCER RES, V39, P491, DOI 10.21873/anticanres.13139

Werner-Wasik M, 2000, INT J RADIAT ONCOL, V48, P689, DOI 10.1016/S0360-3016(00)00699-4

Werner-Wasik M, 2005, SEMIN ONCOL, V32, pS60, DOI 10.1053/j.seminoncol.2005.03.011

Werner-Wasik M, 2011, CLIN LUNG CANCER, V12, P245, DOI 10.1016/j.cllc.2011.03.026

NR 43

TC 4

Z9 5

U1 1

U2 4

PU ELSEVIER IRELAND LTD

PI CLARE

PA ELSEVIER HOUSE, BROOKVALE PLAZA, EAST PARK SHANNON, CO, CLARE, 00000,

IRELAND

EI 2405-6308

J9 CLIN TRANSL RAD ONCO

JI Clin. Transl. Radiat. Oncol.

PD MAY

PY 2020

VL 22

BP 69

EP 75

DI 10.1016/j.ctro.2020.03.007

PG 7

WC Oncology; Radiology, Nuclear Medicine & Medical Imaging

WE Science Citation Index Expanded (SCI-EXPANDED)

SC Oncology; Radiology, Nuclear Medicine & Medical Imaging

GA LL9BE

UT WOS:000531846900011

PM 32274426

OA gold, Green Published

DA 2022-08-24

ER

PT J

AU Bi, N

Wang, JB

Zhang, T

Chen, XY

Xia, WL

Miao, JJ

Xu, KP

Wu, LF

Fan, QR

Wang, LH

Li, YX

Zhou, ZM

Dai, JR

AF Bi, Nan

Wang, Jingbo

Zhang, Tao

Chen, Xinyuan

Xia, Wenlong

Miao, Junjie

Xu, Kunpeng

Wu, Linfang

Fan, Quanrong

Wang, Luhua

Li, Yexiong

Zhou, Zongmei

Dai, Jianrong

TI Deep Learning Improved Clinical Target Volume Contouring Quality and

Efficiency for Postoperative Radiation Therapy in Non-small Cell Lung

Cancer

SO FRONTIERS IN ONCOLOGY

LA English

DT Article

DE non-small cell lung cancer; postoperative radiotherapy; clinical target

volume; deep learning; automatic contour

ID LYMPH-NODE STATIONS; BIG DATA; RADIOTHERAPY; ONCOLOGY; SEGMENTATION;

ATLAS; DEFINITION; OUTCOMES; CT; DELINEATION

AB Purpose: To investigate whether a deep learning-assisted contour (DLAC) could provide greater accuracy, inter-observer consistency, and efficiency compared with a manual contour (MC) of the clinical target volume (CTV) for non-small cell lung cancer (NSCLC) receiving postoperative radiotherapy (PORT). Materials and Methods: A deep dilated residual network was used to achieve the effective automatic contour of the CTV. Eleven junior physicians contoured CTVs on 19 patients by using both MC and DLAC methods independently. Compared with the ground truth, the accuracy of the contour was evaluated by using the Dice coefficient and mean distance to agreement (MDTA). The coefficient of variation (CV) and standard distance deviation (SDD) were rendered to measure the inter-observer variability or consistency. The time consumed for each of the two contouring methods was also compared. Results: A total of 418 CTV sets were generated. DLAC improved contour accuracy when compared with MC and was associated with a larger Dice coefficient (mean +/- SD: 0.75 +/- 0.06 vs. 0.72 +/- 0.07, p < 0.001) and smaller MDTA (mean +/- SD: 2.97 +/- 0.91 mm vs. 3.07 +/- 0.98 mm, p < 0.001). The DLAC was also associated with decreased inter-observer variability, with a smaller CV (mean +/- SD: 0.129 +/- 0.040 vs. 0.183 +/- 0.043, p < 0.001) and SDD (mean +/- SD: 0.47 +/- 0.22 mm vs. 0.72 +/- 0.41 mm, p < 0.001). In addition, a value of 35% of time saving was provided by the DLAC (median: 14.81 min vs. 9.59 min, p < 0.001). Conclusions: Compared with MC, the DLAC is a promising strategy to obtain superior accuracy, consistency, and efficiency for the PORT-CTV in NSCLC.

C1 [Bi, Nan; Wang, Jingbo; Zhang, Tao; Chen, Xinyuan; Xia, Wenlong; Miao, Junjie; Xu, Kunpeng; Wu, Linfang; Fan, Quanrong; Wang, Luhua; Li, Yexiong; Zhou, Zongmei; Dai, Jianrong] Chinese Acad Med Sci & Peking Union Med Coll, Dept Radiat Oncol, Natl Canc Ctr, Natl Clin Res Ctr Canc,Canc Hosp, Beijing, Peoples R China.

[Wang, Luhua] Chinese Acad Med Sci & Peking Union Med Coll, Canc Hosp, Natl Canc Ctr, Natl Clin Res Ctr Canc, Beijing, Peoples R China.

[Wang, Luhua] Chinese Acad Med Sci & Peking Union Med Coll, Shenzhen Hosp, Beijing, Peoples R China.

RP Zhou, ZM; Dai, JR (通讯作者)，Chinese Acad Med Sci & Peking Union Med Coll, Dept Radiat Oncol, Natl Canc Ctr, Natl Clin Res Ctr Canc,Canc Hosp, Beijing, Peoples R China.

EM zhouzongmei2013@163.com; dai_jianrong@cicams.ac.cn

FU CAMS Initiative for Innovative Medicine (CAMS-I2M) [2017-I2M-1-005,

2016-I2M-1-001]; National Natural Science Foundation of China

[11875320]; Non-profit Central Research Institute Fund of Chinese

Academy of Medical Sciences [2018PT32011]

FX This work was supported by the CAMS Initiative for Innovative Medicine

(CAMS-I2M, Grant Nos: 2017-I2M-1-005, 2016-I2M-1-001), the National

Natural Science Foundation of China (Grant No: 11875320), and the

Non-profit Central Research Institute Fund of Chinese Academy of Medical

Sciences (Grant No: 2018PT32011).

CR Bibault JE, 2016, CANCER LETT, V382, P110, DOI 10.1016/j.canlet.2016.05.033

Billiet C, 2016, CANCER TREAT REV, V51, P10, DOI 10.1016/j.ctrv.2016.10.001

Chapet O, 2005, INT J RADIAT ONCOL, V63, P170, DOI 10.1016/j.ijrobp.2004.12.060

Comelli A, 2019, ARTIF INTELL MED, V94, P67, DOI 10.1016/j.artmed.2019.01.002

Corso CD, 2015, J THORAC ONCOL, V10, P148, DOI 10.1097/JTO.0000000000000406

Cui YF, 2015, PRACT RADIAT ONCOL, V5, pE67, DOI 10.1016/j.prro.2014.05.005

Eaton BR, 2016, JNCI-J NATL CANCER I, V108, DOI 10.1093/jnci/djw034

Feng X, 2019, MED PHYS, V46, P2169, DOI 10.1002/mp.13466

Giri MG, 2016, MED PHYS, V43, P2491, DOI 10.1118/1.4947123

Herskovic A, 2017, J THORAC ONCOL, V12, P302, DOI 10.1016/j.jtho.2016.09.135

Hillner BE, 2000, J CLIN ONCOL, V18, P2327, DOI 10.1200/JCO.2000.18.11.2327

Ibragimov B, 2018, MED PHYS, V45, P4763, DOI 10.1002/mp.13122

Itazawa T, 2017, J RADIAT RES, V58, P86, DOI 10.1093/jrr/rrw076

Jia YQ, 2014, PROCEEDINGS OF THE 2014 ACM CONFERENCE ON MULTIMEDIA (MM'14), P675, DOI 10.1145/2647868.2654889

Jiang J, 2018, LECT NOTES COMPUT SC, V11071, P777, DOI 10.1007/978-3-030-00934-2_86

Lin L, 2019, RADIOLOGY, V291, P677, DOI 10.1148/radiol.2019182012

Liu C, 2019, INT J RADIAT ONCOL, V104, P924, DOI 10.1016/j.ijrobp.2019.03.017

Louie AV, 2010, RADIOTHER ONCOL, V95, P166, DOI 10.1016/j.radonc.2009.12.028

Lustberg T, 2018, RADIOTHER ONCOL, V126, P312, DOI 10.1016/j.radonc.2017.11.012

Men K, 2018, PHYS MED BIOL, V63, DOI 10.1088/1361-6560/aada6c

Men K, 2018, PHYS MEDICA, V50, P13, DOI 10.1016/j.ejmp.2018.05.006

Men K, 2017, MED PHYS, V44, P6377, DOI 10.1002/mp.12602

Mikell JL, 2015, J THORAC ONCOL, V10, P462, DOI 10.1097/JTO.0000000000000411

Mikell JK, 2018, EJNMMI PHYS, V5, DOI 10.1186/s40658-018-0230-y

Nouranian S, 2015, IEEE T MED IMAGING, V34, P950, DOI 10.1109/TMI.2014.2371823

O'Sullivan D., 2010, GEOGRAPHICAL INFORM

Ohri N, 2013, JNCI-J NATL CANCER I, V105, P387, DOI 10.1093/jnci/djt001

Pallavaram S, 2015, NEUROSURGERY, V76, P756, DOI 10.1227/NEU.0000000000000714

Piert M, 2018, EJNMMI RES, V8, DOI 10.1186/s13550-018-0377-5

Robinson CG, 2015, J CLIN ONCOL, V33, P870, DOI 10.1200/JCO.2014.58.5380

Rusch VW, 2009, J THORAC ONCOL, V4, P568, DOI 10.1097/JTO.0b013e3181a0d82e

Segedin B, 2016, RADIOL ONCOL, V50, P254, DOI 10.1515/raon-2016-0023

Spoelstra FOB, 2010, INT J RADIAT ONCOL, V76, P1106, DOI 10.1016/j.ijrobp.2009.02.072

Taha AA, 2015, BMC MED IMAGING, V15, DOI 10.1186/s12880-015-0068-x

Urban D, 2013, J THORAC ONCOL, V8, P940, DOI 10.1097/JTO.0b013e318292c53e

Van Baardwijk A, 2007, INT J RADIAT ONCOL, V68, P771, DOI 10.1016/j.ijrobp.2006.12.067

Van de Steene J, 2002, RADIOTHER ONCOL, V62, P37, DOI 10.1016/S0167-8140(01)00453-4

Vinod SK, 2016, RADIOTHER ONCOL, V121, P169, DOI 10.1016/j.radonc.2016.09.009

Wang EH, 2015, J THORAC ONCOL, V10, P937, DOI 10.1097/JTO.0000000000000519

Wang LH, 2017, SEMIN RADIAT ONCOL, V27, P164, DOI 10.1016/j.semradonc.2016.11.008

Wisnivesky JP, 2012, CANCER-AM CANCER SOC, V118, P4478, DOI 10.1002/cncr.26585

Yang JZ, 2018, MED PHYS, V45, P4568, DOI 10.1002/mp.13141

Zhuang MZ, 2016, MED PHYS, V43, P4483, DOI 10.1118/1.4954844

NR 43

TC 14

Z9 15

U1 2

U2 18

PU FRONTIERS MEDIA SA

PI LAUSANNE

PA AVENUE DU TRIBUNAL FEDERAL 34, LAUSANNE, CH-1015, SWITZERLAND

SN 2234-943X

J9 FRONT ONCOL

JI Front. Oncol.

PD NOV 13

PY 2019

VL 9

AR 1192

DI 10.3389/fonc.2019.01192

PG 8

WC Oncology

WE Science Citation Index Expanded (SCI-EXPANDED)

SC Oncology

GA JR2RQ

UT WOS:000499479100001

PM 31799181

OA Green Published, gold

DA 2022-08-24

ER

PT J

AU Valdes, G

Solberg, TD

Heskel, M

Ungar, L

Simone, CB

AF Valdes, Gilmer

Solberg, Timothy D.

Heskel, Marina

Ungar, Lyle

Simone, Charles B., II

TI Using machine learning to predict radiation pneumonitis in patients with

stage I non-small cell lung cancer treated with stereotactic body

radiation therapy

SO PHYSICS IN MEDICINE AND BIOLOGY

LA English

DT Article

DE radiation pneumonitis; stereotactic body radiation therapy (SBRT);

non-small cell lung cancer; machine learning; Decision Trees; RUSBoost;

Random Forests

ID DOSE-VOLUME HISTOGRAM; INDUCED PULMONARY TOXICITY; RADIOTHERAPY

OUTCOMES; RISK

AB To develop a patient-specific 'big data' clinical decision tool to predict pneumonitis in stage I non-small cell lung cancer (NSCLC) patients after stereotactic body radiation therapy (SBRT).

61 features were recorded for 201 consecutive patients with stage I NSCLC treated with SBRT, in whom 8 (4.0%) developed radiation pneumonitis. Pneumonitis thresholds were found for each feature individually using decision stumps. The performance of three different algorithms (Decision Trees, Random Forests, RUSBoost) was evaluated. Learning curves were developed and the training error analyzed and compared to the testing error in order to evaluate the factors needed to obtain a cross-validated error smaller than 0.1. These included the addition of new features, increasing the complexity of the algorithm and enlarging the sample size and number of events.

In the univariate analysis, the most important feature selected was the diffusion capacity of the lung for carbon monoxide (DLCO adj%). On multivariate analysis, the three most important features selected were the dose to 15 cc of the heart, dose to 4 cc of the trachea or bronchus, and race. Higher accuracy could be achieved if the RUSBoost algorithm was used with regularization. To predict radiation pneumonitis within an error smaller than 10%, we estimate that a sample size of 800 patients is required.

Clinically relevant thresholds that put patients at risk of developing radiation pneumonitis were determined in a cohort of 201 stage I NSCLC patients treated with SBRT. The consistency of these thresholds can provide radiation oncologists with an estimate of their reliability and may inform treatment planning and patient counseling. The accuracy of the classification is limited by the number of patients in the study and not by the features gathered or the complexity of the algorithm.

C1 [Valdes, Gilmer; Solberg, Timothy D.; Heskel, Marina; Simone, Charles B., II] Univ Penn, Dept Radiat Oncol, Perelman Ctr Adv Med, Philadelphia, PA 19104 USA.

[Ungar, Lyle] Univ Penn, Dept Comp & Informat Sci, 200 S 33Rd St, Philadelphia, PA 19104 USA.

RP Valdes, G (通讯作者)，Univ Penn, Dept Radiat Oncol, Perelman Ctr Adv Med, Philadelphia, PA 19104 USA.

EM gilmer.valdes@ucsf.edu

OI Simone, Charles/0000-0002-0867-3694; , Timothy/0000-0001-8829-7774

FU NATIONAL HUMAN GENOME RESEARCH INSTITUTE [T32HG000046] Funding Source:

NIH RePORTER

CR Arias M, 2014, PHARMACOECONOMICS, V32, P1141, DOI 10.1007/s40273-014-0195-1

BREIMAN L, 2001, MACH LEARN, V0045

Breiman L., 2004, 670 UC BERK

Chang DT, 2006, INT J RADIAT ONCOL, V65, P125, DOI 10.1016/j.ijrobp.2005.09.047

Chen S, 2007, MED PHYS, V34, P3808, DOI 10.1118/1.2776669

Cunliffe A, 2015, INT J RADIAT ONCOL, V91, P1048, DOI 10.1016/j.ijrobp.2014.11.030

Dang J, 2014, LUNG CANCER, V86, P329, DOI 10.1016/j.lungcan.2014.10.005

El Naqa I, 2006, INT J RADIAT ONCOL, V64, P1275, DOI 10.1016/j.ijrobp.2005.11.022

El Naqa I, 2009, PHYS MED BIOL, V54, pS9, DOI 10.1088/0031-9155/54/18/S02

Graham MV, 1999, INT J RADIAT ONCOL, V45, P323, DOI 10.1016/S0360-3016(99)00183-2

Hastie T., 2009, SPRINGER SERIES STAT, V2nd ed., DOI [10.1007/978-0-387-84858-7, DOI 10.1007/978-0-387-21606-5]

Hernando ML, 2001, INT J RADIAT ONCOL, V51, P650, DOI 10.1016/S0360-3016(01)01685-6

Hope AJ, 2006, INT J RADIAT ONCOL, V65, P112, DOI 10.1016/j.ijrobp.2005.11.046

Kang J, 2015, INT J RADIAT ONCOL, V93, P1127, DOI 10.1016/j.ijrobp.2015.07.2286

Kim M, 2011, RADIAT ONCOL J, V29, P181, DOI 10.3857/roj.2011.29.3.181

Klement RJ, 2014, INT J RADIAT ONCOL, V88, P732, DOI 10.1016/j.ijrobp.2013.11.216

Kwa SLS, 1998, INT J RADIAT ONCOL, V42, P1, DOI 10.1016/S0360-3016(98)00196-5

Lambin P, 2013, RADIOTHER ONCOL, V109, P159, DOI 10.1016/j.radonc.2013.07.007

Lee S, 2015, MED PHYS, V42, P2421, DOI 10.1118/1.4915284

Lind PA, 2006, INT J RADIAT ONCOL, V64, P765, DOI 10.1016/j.ijrobp.2005.08.011

MARTEL MK, 1994, INT J RADIAT ONCOL, V28, P575, DOI 10.1016/0360-3016(94)90181-3

Moiseenko V, 2003, RADIOTHER ONCOL, V67, P265, DOI 10.1016/S0167-8140(03)00003-3

Parkin DM, 2005, CA-CANCER J CLIN, V55, P74, DOI 10.3322/canjclin.55.2.74

Pudil J N P, 1994, PATTERN RECOGNIT LET, V15, P6

Rancati T, 2003, RADIOTHER ONCOL, V67, P275, DOI 10.1016/S0167-8140(03)00119-1

Rijsbergen C. J. V., 1979, INFORM RETRIEVAL

Robnett TJ, 2000, INT J RADIAT ONCOL, V48, P89, DOI 10.1016/S0360-3016(00)00648-9

Saeys Y, 2007, BIOINFORMATICS, V23, P2507, DOI 10.1093/bioinformatics/btm344

Seiffert C, 2010, IEEE T SYST MAN CY A, V40, P185, DOI 10.1109/TSMCA.2009.2029559

Simone CB, 2015, ANN TRANSL MED, V3, DOI 10.3978/j.issn.2305-5839.2015.07.26

Simone CB, 2013, CHEST, V143, P1784, DOI 10.1378/chest.12-2580

Theuws JCM, 1998, RADIOTHER ONCOL, V48, P33, DOI 10.1016/S0167-8140(98)00019-X

Theuws JCM, 1998, RADIOTHER ONCOL, V49, P233, DOI 10.1016/S0167-8140(98)00117-0

Tsujino K, 2006, INT J RADIAT ONCOL, V64, P1100, DOI 10.1016/j.ijrobp.2005.09.025

NR 34

TC 58

Z9 60

U1 2

U2 22

PU IOP Publishing Ltd

PI BRISTOL

PA TEMPLE CIRCUS, TEMPLE WAY, BRISTOL BS1 6BE, ENGLAND

SN 0031-9155

EI 1361-6560

J9 PHYS MED BIOL

JI Phys. Med. Biol.

PD AUG 21

PY 2016

VL 61

IS 16

BP 6105

EP 6120

DI 10.1088/0031-9155/61/16/6105

PG 16

WC Engineering, Biomedical; Radiology, Nuclear Medicine & Medical Imaging

WE Science Citation Index Expanded (SCI-EXPANDED)

SC Engineering; Radiology, Nuclear Medicine & Medical Imaging

GA DX2NZ

UT WOS:000384208600018

PM 27461154

OA Green Accepted

DA 2022-08-24

ER

PT J

AU Hu, SL

Luo, M

Li, YL

AF Hu, Shuli

Luo, Man

Li, Yaling

TI Machine Learning for the Prediction of Lymph Nodes Micrometastasis in

Patients with Non-Small Cell Lung Cancer: A Comparative Analysis of Two

Practical Prediction Models for Gross Target Volume Delineation

SO CANCER MANAGEMENT AND RESEARCH

LA English

DT Article

DE non-small cell lung cancer; lymph nodes micrometastasis; prediction

model; random forest; gross target volume; machine learning

ID POSITRON-EMISSION-TOMOGRAPHY; RADIATION-THERAPY; METASTASIS;

CLASSIFICATION; ADENOCARCINOMA; RADIOTHERAPY; PROGNOSIS; SURGERY; SIZE;

TNM

AB Purpose: The lymph node gross target volume (GTV) delineation in patients with non-small cell lung cancer (NSCLC) is crucial for prognosis. This study aimed to develop a predictive model that can be used to differentiate between lymph nodes micrometastasis (LNM) and non-lymph nodes micrometastasis (non-LNM).

Patients and Methods: A retrospective study involving 1524 patients diagnosed with NSCLC was collected in the First Hospital of Wuhan between January 1, 2017, and April 1, 2020. Duplicated and useless variables were excluded, and 16 candidate variables were selected for further analysis. The random forest (RF) algorithm and generalized linear (GL) algorithm were used to screen out the variables that greatly affected the LNM prediction, respectively. The area under the curve (AUC) was compared between the RF model and GL model.

Results: The RF model revealed that the variables, including pathology, degree of differentiation, maximum short diameter of lymph node, tumor diameter, pulmonary membrane invasion, clustered lymph nodes, and T stage, were more significant for LNM prediction. Multifactorial logistic regression analysis for the GL model indicated that vascular invasion, tumor diameter, degree of differentiation, pulmonary membrane invasion, and maximum standard uptake value (SUVmax) were positively associated with LNM. The AUC for the RF model and GL model was 0.83 (95% CI: 0.75 to 0.90) and 0.64 (95% CI: 0.60 to 0.70), respectively.

Conclusion: We successfully established an accurate and optimized RF model that could be used to predict LNM in patients with NSCLC. This model can be used to evaluate the risk of an individual patient experiencing LNM and therefore facilitate the choice of treatment.

C1 [Hu, Shuli; Li, Yaling] Wuhan 1 Hosp, Dept Intens Care Unit, 215 Zhongshan Rd, Wuhan 430022, Hubei, Peoples R China.

[Luo, Man] Wuhan 1 Hosp, Dept Oncol, Wuhan 430022, Peoples R China.

RP Li, YL (通讯作者)，Wuhan 1 Hosp, Dept Intens Care Unit, 215 Zhongshan Rd, Wuhan 430022, Hubei, Peoples R China.

EM 2861696710@qq.com

FU Special Fund for Clinical Research of Wu Jieping Medical Foundation

[320.6750.18463]

FX This research was supported by the Special Fund for Clinical Research of

Wu Jieping Medical Foundation (No.320.6750.18463).

CR Amin MB, 2017, CA-CANCER J CLIN, V67, P93, DOI 10.3322/caac.21388

Armstrong RA, 2014, OPHTHAL PHYSL OPT, V34, P502, DOI 10.1111/opo.12131

Bade BC, 2020, CLIN CHEST MED, V41, P1, DOI 10.1016/j.ccm.2019.10.001

Bille A, 2009, EUR J CARDIO-THORAC, V36, P440, DOI 10.1016/j.ejcts.2009.04.003

Brooks ED, 2020, J THORAC ONCOL, V15, P176, DOI 10.1016/j.jtho.2019.10.016

Chen KZ, 2013, ANN THORAC SURG, V96, P1761, DOI 10.1016/j.athoracsur.2013.06.038

Cortes J, 2020, CA-CANCER J CLIN, V70, P105, DOI 10.3322/caac.21597

De Leyn P, 2014, EUR J CARDIO-THORAC, V45, P787, DOI 10.1093/ejcts/ezu028

de Vries FEE, 2014, EJSO-EUR J SURG ONC, V40, P1777, DOI 10.1016/j.ejso.2014.08.483

Dong M, 2017, J MED IMAG RADIAT ON, V61, P652, DOI 10.1111/1754-9485.12599

Dreiseitl S, 2002, J BIOMED INFORM, V35, P352, DOI 10.1016/S1532-0464(03)00034-0

Edge SB, 2010, ANN SURG ONCOL, V17, P1471, DOI 10.1245/s10434-010-0985-4

Ettinger DS, 2015, J NATL COMPR CANC NE, V13, P515, DOI 10.6004/jnccn.2015.0071

Feng SH, 2019, DIAGN INTERV RADIOL, V25, P270, DOI 10.5152/dir.2019.18458

Gorai A, 2015, EUR J CARDIO-THORAC, V47, P653, DOI 10.1093/ejcts/ezu244

Graham ANJ, 1999, J THORAC CARDIOV SUR, V117, P246, DOI 10.1016/S0022-5223(99)70419-8

Haruki T, 2017, ANN THORAC CARDIOVAS, V23, P181, DOI 10.5761/atcs.oa.16-00309

Herbst RS, 2018, NATURE, V553, P446, DOI 10.1038/nature25183

Hetth FJF, 2008, CHEST, V133, P887, DOI 10.1378/chest.07-2535

Ito M, 2014, LUNG CANCER, V85, P270, DOI 10.1016/j.lungcan.2014.05.014

Kaseda K, 2016, WORLD J SURG, V40, P2976, DOI 10.1007/s00268-016-3652-5

Kelsey CR, 2009, CANCER-AM CANCER SOC, V115, P5218, DOI 10.1002/cncr.24625

Kristensen E, 2017, BEHAV RES METHODS, V49, P2255, DOI 10.3758/s13428-017-0856-z

Lee NK, 2013, CLIN LUNG CANCER, V14, P399, DOI 10.1016/j.cllc.2012.11.002

Moon Y, 2014, WORLD J SURG ONCOL, V12, DOI 10.1186/1477-7819-12-388

Mountain CF, 1997, CHEST, V111, P1718, DOI 10.1378/chest.111.6.1718

Murgu SD, 2015, CHEST, V147, P1401, DOI 10.1378/chest.14-1355

Nagata Y, 2015, INT J RADIAT ONCOL, V93, P989, DOI 10.1016/j.ijrobp.2015.07.2278

Nambu A, 2010, EUR J RADIOL, V73, P510, DOI 10.1016/j.ejrad.2009.01.021

Nomori H, 2004, J THORAC CARDIOV SUR, V127, P1087, DOI 10.1016/j.jtcvs.2003.08.010

Qu YM, 2015, PHARM STAT, V14, P56, DOI 10.1002/pst.1658

Rusch VW, 2009, J THORAC ONCOL, V4, P568, DOI 10.1097/JTO.0b013e3181a0d82e

Scott M, 2014, J SMALL ANIM PRACT, V55, P527, DOI 10.1111/jsap.12260

Shirai K, 2019, FRONT ONCOL, V9, DOI 10.3389/fonc.2019.00731

Song L, 2013, BMC BIOINFORMATICS, V14, DOI 10.1186/1471-2105-14-5

Strobl C, 2007, BMC BIOINFORMATICS, V8, DOI 10.1186/1471-2105-8-25

VANDENBREKEL MWM, 1990, RADIOLOGY, V177, P379, DOI 10.1148/radiology.177.2.2217772

Vorwerk H, 2009, RADIOTHER ONCOL, V91, P455, DOI 10.1016/j.radonc.2009.03.014

Wu S, 2020, J AM MED INFORM ASSN, V27, P457, DOI 10.1093/jamia/ocz200

Yanagawa N, 2014, ANN THORAC SURG, V98, P453, DOI 10.1016/j.athoracsur.2014.04.108

Yu Y, 2018, THORAC CANCER, V9, P516, DOI 10.1111/1759-7714.12598

Zhao F, 2019, BMC CANCER, V19, DOI 10.1186/s12885-019-5632-2

NR 42

TC 1

Z9 1

U1 1

U2 2

PU DOVE MEDICAL PRESS LTD

PI ALBANY

PA PO BOX 300-008, ALBANY, AUCKLAND 0752, NEW ZEALAND

SN 1179-1322

J9 CANCER MANAG RES

JI Cancer Manag. Res.

PY 2021

VL 13

BP 4811

EP 4820

DI 10.2147/CMAR.S313941

PG 10

WC Oncology

WE Science Citation Index Expanded (SCI-EXPANDED)

SC Oncology

GA ST0SH

UT WOS:000662160700001

PM 34168500

OA Green Published

DA 2022-08-24

ER

PT J

AU Zhang, YC

Oikonomou, A

Wong, A

Haider, MA

Khalvati, F

AF Zhang, Yucheng

Oikonomou, Anastasia

Wong, Alexander

Haider, Masoom A.

Khalvati, Farzad

TI Radiomics-based Prognosis Analysis for Non-Small Cell Lung Cancer

SO SCIENTIFIC REPORTS

LA English

DT Article

ID CT TEXTURE ANALYSIS; FEATURE-SELECTION; CLASSIFICATION; PREDICTION;

SMOTE

AB Radiomics characterizes tumor phenotypes by extracting large numbers of quantitative features from radiological images. Radiomic features have been shown to provide prognostic value in predicting clinical outcomes in several studies. However, several challenges including feature redundancy, unbalanced data, and small sample sizes have led to relatively low predictive accuracy. In this study, we explore different strategies for overcoming these challenges and improving predictive performance of radiomics-based prognosis for non-small cell lung cancer (NSCLC). CT images of 112 patients (mean age 75 years) with NSCLC who underwent stereotactic body radiotherapy were used to predict recurrence, death, and recurrence-free survival using a comprehensive radiomics analysis. Different feature selection and predictive modeling techniques were used to determine the optimal configuration of prognosis analysis. To address feature redundancy, comprehensive analysis indicated that Random Forest models and Principal Component Analysis were optimum predictive modeling and feature selection methods, respectively, for achieving high prognosis performance. To address unbalanced data, Synthetic Minority Over-sampling technique was found to significantly increase predictive accuracy. A full analysis of variance showed that data endpoints, feature selection techniques, and classifiers were significant factors in affecting predictive accuracy, suggesting that these factors must be investigated when building radiomics-based predictive models for cancer prognosis.

C1 [Zhang, Yucheng; Oikonomou, Anastasia; Haider, Masoom A.; Khalvati, Farzad] Univ Toronto, Sunnybrook Res Inst, Dept Med Imaging, Toronto, ON, Canada.

[Wong, Alexander] Univ Waterloo, Dept Syst Design Engn, Waterloo, ON, Canada.

RP Khalvati, F (通讯作者)，Univ Toronto, Sunnybrook Res Inst, Dept Med Imaging, Toronto, ON, Canada.

EM farzad.khalvati@sri.utoronto.ca

RI Haider, Masoom/Q-1315-2017

FU Sunnybrook Research Summer Student Award Program; Ontario Institute for

Cancer Research (OICR)

FX This research has been supported by Sunnybrook Research Summer Student

Award Program and Ontario Institute for Cancer Research (OICR).

CR Aerts HJWL, 2014, NAT COMMUN, V5, DOI 10.1038/ncomms5006

Al-Shahib Ali, 2005, Appl Bioinformatics, V4, P195, DOI 10.2165/00822942-200504030-00004

Allemani C, 2015, LANCET, V385, P977, DOI 10.1016/S0140-6736(14)62038-9

Bermingham ML, 2015, SCI REP-UK, V5, DOI 10.1038/srep10312

Blagus R, 2013, BMC BIOINFORMATICS, V14, DOI 10.1186/1471-2105-14-106

BREIMAN L, 2001, MACH LEARN, V0045

Cameron A, 2016, IEEE T BIO-MED ENG, V63, P1145, DOI 10.1109/TBME.2015.2485779

Chawla NV, 2002, J ARTIF INTELL RES, V16, P321, DOI 10.1613/jair.953

Dy JG, 2004, J MACH LEARN RES, V5, P845

Fawcett T, 2006, PATTERN RECOGN LETT, V27, P861, DOI 10.1016/j.patrec.2005.10.010

Figueroa RL, 2012, BMC MED INFORM DECIS, V12, DOI 10.1186/1472-6947-12-8

Fort G, 2005, BIOINFORMATICS, V21, P1104, DOI 10.1093/bioinformatics/bti114

Ganeshan B, 2012, EUR RADIOL, V22, P796, DOI 10.1007/s00330-011-2319-8

Ganeshan B, 2010, CANCER IMAGING, V10, P137, DOI 10.1102/1470-7330.2010.0021

Gillies RJ, 2016, RADIOLOGY, V278, P563, DOI 10.1148/radiol.2015151169

Haider MA, 2017, CANCER IMAGING, V17, DOI 10.1186/s40644-017-0106-8

Hearst MA, 1998, IEEE INTELL SYST APP, V13, P18, DOI 10.1109/5254.708428

Hira Z. M., 2015, REV FEATURE SELECTIO, V2015

Huang LC, 2009, J TRANSL MED, V7, DOI 10.1186/1479-5876-7-81

Huang YQ, 2016, RADIOLOGY, V281, P947, DOI 10.1148/radiol.2016152234

Jia Wu, 2016, RADIOLOGY, V281

Khalvati F, 2015, BMC MED IMAGING, V15, DOI 10.1186/s12880-015-0069-9

Kotsiantis S B, 2007, INFORMATICA, V31, P249, DOI DOI 10.31449/INF.V31I3.148

Kotsiantis S. B., 2007, MACHINE LEARNING REV, P159, DOI [10.1007/s10462-007-9052-3, DOI 10.1007/S10462-007-9052-3]

Krizhevsky A., 2012, ADV NEURAL INFORM PR, V25, DOI DOI 10.1145/3065386

Kuhn M, 2008, J STAT SOFTW, V28, P1, DOI 10.18637/jss.v028.i05

Kumar V, 2012, MAGN RESON IMAGING, V30, P1234, DOI 10.1016/j.mri.2012.06.010

Lambin P, 2012, EUR J CANCER, V48, P441, DOI 10.1016/j.ejca.2011.11.036

Larran P., 2007, REV FEATURE SELECTIO, V23, P2507

Monti S, 2003, MACH LEARN, V52, P91, DOI 10.1023/A:1023949509487

Parekh V, 2016, EXPERT REV PRECIS ME, V1, P207, DOI 10.1080/23808993.2016.1164013

Parmar C, 2015, SCI REP-UK, V5, DOI 10.1038/srep13087

Parmar C, 2015, SCI REP-UK, V5, DOI 10.1038/srep11044

Parmar C, 2014, PLOS ONE, V9, DOI 10.1371/journal.pone.0102107

Provost F., 2000, P AAAI 2000 WORKSH, DOI 10.1.1.33.507

Pyka T, 2015, RADIAT ONCOL, V10, DOI 10.1186/s13014-015-0407-7

Zhang J., 2016, J COMPUT VIS IMAGING, V2

NR 37

TC 140

Z9 148

U1 8

U2 37

PU NATURE PORTFOLIO

PI BERLIN

PA HEIDELBERGER PLATZ 3, BERLIN, 14197, GERMANY

SN 2045-2322

J9 SCI REP-UK

JI Sci Rep

PD APR 18

PY 2017

VL 7

AR 46349

DI 10.1038/srep46349

PG 8

WC Multidisciplinary Sciences

WE Science Citation Index Expanded (SCI-EXPANDED)

SC Science & Technology - Other Topics

GA ES5VY

UT WOS:000399615400001

PM 28418006

OA Green Published, gold

DA 2022-08-24

ER

PT J

AU Kodama, T

Arimura, H

Shirakawa, Y

Ninomiya, K

Yoshitake, T

Shioyama, Y

AF Kodama, Takumi

Arimura, Hidetaka

Shirakawa, Yuko

Ninomiya, Kenta

Yoshitake, Tadamasa

Shioyama, Yoshiyuki

TI Relapse predictability of topological signature on pretreatment planning

CT images of stage I non-small cell lung cancer patients before

treatment with stereotactic ablative radiotherapy

SO THORACIC CANCER

LA English

DT Article

DE non-small cell lung cancer (NSCLC); radiotherapy; relapse; topology

ID BODY RADIATION-THERAPY; PREDICTION; RADIOMICS; FEATURES; SURGERY;

PROGNOSIS

AB Background This study aimed to explore the predictability of topological signatures linked to the locoregional relapse (LRR) and distant metastasis (DM) on pretreatment planning computed tomography images of stage I non-small cell lung cancer (NSCLC) patients before treatment with stereotactic ablative radiotherapy (SABR). Methods We divided 125 primary stage I NSCLC patients (LRR: 34, DM: 22) into training (n = 60) and test datasets (n = 65), and the training dataset was augmented to 260 cases using a synthetic minority oversampling technique. The relapse predictabilities of the conventional wavelet-based features (WF), topology-based features [BF, Betti number (BN) map features; iBF, inverted BN map features], and their combined features (BWF, iBWF) were compared. The patients were stratified into high-risk and low-risk groups using the medians of the radiomics scores in the training dataset. Results For the LRR in the test, the iBF, iBWF, and WF showed statistically significant differences (p < 0.05), and the highest nLPC was obtained for the iBF. For the DM in the test, the iBWF showed a significant difference and the highest nLPC. Conclusion The iBF indicated the potential of improving the LRR and DM prediction of stage I NSCLC patients prior to undergoing SABR.

C1 [Kodama, Takumi] Kyushu Univ, Grad Sch Med Sci, Dept Hlth Sci, Div Med Quantum Sci, Fukuoka, Japan.

[Arimura, Hidetaka] Kyushu Univ, Fac Med Sci, Dept Hlth Sci, Div Med Quantum Sci, Fukuoka, Japan.

[Shirakawa, Yuko] Natl Hosp Org Kyushu Canc Ctr, Fukuoka, Japan.

[Ninomiya, Kenta] Sanford Burnham Prebys Med Discovery Inst, La Jolla, CA USA.

[Yoshitake, Tadamasa] Kyushu Univ, Grad Sch Med Sci, Dept Clin Radiol, Fukuoka, Japan.

[Shioyama, Yoshiyuki] SAGA HIMAT Fdn, Ion Beam Therapy Ctr, Tosu, Saga, Japan.

RP Arimura, H (通讯作者)，Kyushu Univ, Dept Hlth Sci, Fac Med Sci, Higashi Ku, 3-1-1 Maidashi, Fukuoka, Japan.

EM arimura.hidetaka.616@m.kyushu-u.ac.jp

OI Kodama, Takumi/0000-0002-8134-0837

FU JSPS KAKENHI [JP20K08084]

FX JSPS KAKENHI, Grant/Award Number: JP20K08084

CR Ackerson BG, 2018, LUNG CANCER, V125, P185, DOI 10.1016/j.lungcan.2018.09.020

[Anonymous], 2018, HMISC V4 1 1

[Anonymous], GLMNETV

[Anonymous], 2020, SURVIVAL V32 7

Balari S Lorenzo G Gonzalez G., 2013, COMPUTATIONAL PHENOT, DOI [10.1093/acprof:oso/9780199665464.001.0001, DOI 10.1093/ACPROF:OSO/9780199665464.001.0001]

Bray F, 2018, CA-CANCER J CLIN, V68, P394, DOI 10.3322/caac.21492

Chawla NV, 2002, J ARTIF INTELL RES, V16, P321, DOI 10.1613/jair.953

Collins LG, 2007, AM FAM PHYSICIAN, V75, P56

Ettinger DS, 2017, J NATL COMPR CANC NE, V15, P504, DOI 10.6004/jnccn.2017.0050

HARRELL FE, 1982, JAMA-J AM MED ASSOC, V247, P2543, DOI 10.1001/jama.247.18.2543

HERMAN GT, 1992, IEEE COMPUT GRAPH, V12, P69, DOI 10.1109/38.135915

Kadoya N, 2020, MED PHYS, V47, P2197, DOI 10.1002/mp.14104

Kakino R, 2020, MED PHYS, V47, P4634, DOI 10.1002/mp.14380

Kolodziejski LS, 2003, NEOPLASMA, V50, P66

Ma LF, 2016, THORAC CANCER, V7, P442, DOI 10.1111/1759-7714.12352

Mackin D, 2018, SCI REP-UK, V8, DOI 10.1038/s41598-018-20713-6

Mattonen SA, 2016, INT J RADIAT ONCOL, V94, P1121, DOI 10.1016/j.ijrobp.2015.12.369

Ninomiya K, 2021, PLOS ONE, V16, DOI 10.1371/journal.pone.0244354

Ninomiya K, 2020, PHYS MEDICA, V69, P90, DOI 10.1016/j.ejmp.2019.11.026

Onishi H, 2011, INT J RADIAT ONCOL, V81, P1352, DOI 10.1016/j.ijrobp.2009.07.1751

Santos MK, 2014, EUR J RADIOL, V83, P1275, DOI 10.1016/j.ejrad.2014.04.019

Schmid M, 2016, EXPERT SYST APPL, V63, P450, DOI 10.1016/j.eswa.2016.07.018

Seo YS, 2019, THORAC CANCER, V10, P1489, DOI 10.1111/1759-7714.13103

Soufi M, 2018, MED PHYS, V45, P5116, DOI 10.1002/mp.13202

Tandberg DJ, 2018, CANCER-AM CANCER SOC, V124, P667, DOI 10.1002/cncr.31196

Thawani R, 2018, LUNG CANCER, V115, P34, DOI 10.1016/j.lungcan.2017.10.015

Timmerman RD, 2018, JAMA ONCOL, V4, P1263, DOI 10.1001/jamaoncol.2018.1251

Vallieres, 2015, RADIOMICS MATLAB PRO

Vallieres M, 2015, PHYS MED BIOL, V60, P5471, DOI 10.1088/0031-9155/60/14/5471

Wang SD, 2018, SCI REP-UK, V8, DOI 10.1038/s41598-018-27707-4

Wu J, 2016, RADIOLOGY, V281, P270, DOI 10.1148/radiol.2016151829

Yang Y, 2021, FRONT ONCOL, V11, DOI 10.3389/fonc.2021.746785

Zheng XP, 2014, INT J RADIAT ONCOL, V90, P603, DOI 10.1016/j.ijrobp.2014.05.055

NR 33

TC 0

Z9 0

U1 1

U2 1

PU WILEY

PI HOBOKEN

PA 111 RIVER ST, HOBOKEN 07030-5774, NJ USA

SN 1759-7706

EI 1759-7714

J9 THORAC CANCER

JI Thorac. Cancer

PD AUG

PY 2022

VL 13

IS 15

BP 2117

EP 2126

DI 10.1111/1759-7714.14483

EA JUN 2022

PG 10

WC Oncology; Respiratory System

WE Science Citation Index Expanded (SCI-EXPANDED)

SC Oncology; Respiratory System

GA 3N0QZ

UT WOS:000811891700001

PM 35711108

OA Green Published

DA 2022-08-24

ER

PT J

AU Yoo, J

Lee, J

Cheon, M

Woo, SK

Ahn, MJ

Pyo, HR

Choi, YS

Han, JH

Choi, JY

AF Yoo, Jang

Lee, Jaeho

Cheon, Miju

Woo, Sang-Keun

Ahn, Myung-Ju

Pyo, Hong Ryull

Choi, Yong Soo

Han, Joung Ho

Choi, Joon Young

TI Predictive Value of F-18-FDG PET/CT Using Machine Learning for

Pathological Response to Neoadjuvant Concurrent Chemoradiotherapy in

Patients with Stage III Non-Small Cell Lung Cancer

SO CANCERS

LA English

DT Article

DE non-small cell lung cancer; neoadjuvant concurrent chemoradiotherapy;

F-18-FDG PET; CT; machine learning; random forest; pathologic complete

response

ID THERAPY; CHEMOTHERAPY; SURGERY; RADIOTHERAPY; CRITERIA; PERCIST; VOLUME;

TUMORS

AB Simple Summary The pathological complete response (pCR) after neoadjuvant chemoradiotherapy (CCRT) is an independent prognostic factor for progression-free and overall survival in non-small cell lung cancer (NSCLC). F-18-FDG PET/CT has been performed for initial staging work-up, treatment response, and follow-up in patients with NSCLC. Machine learning (ML) as an empirical data science has become relevant to nuclear medicine. We investigated the predictive performance of F-18-FDG PET/CT using an ML model to assess the treatment response to neoadjuvant CCRT in patients with stage III NSCLC, and compared the performance of the ML model predictions to predictions from conventional PET parameters and from physicians. The predictions from the ML model using radiomic features of F-18-FDG PET/CT provided better accuracy than predictions from conventional PET parameters and from physicians for the neoadjuvant CCRT response of stage III non-small cell lung cancer. We investigated predictions from F-18-FDG PET/CT using machine learning (ML) to assess the neoadjuvant CCRT response of patients with stage III non-small cell lung cancer (NSCLC) and compared them with predictions from conventional PET parameters and from physicians. A retrospective study was conducted of 430 patients. They underwent F-18-FDG PET/CT before initial treatment and after neoadjuvant CCRT followed by curative surgery. We analyzed texture features from segmented tumors and reviewed the pathologic response. The ML model employed a random forest and was used to classify the binary outcome of the pathological complete response (pCR). The predictive accuracy of the ML model for the pCR was 93.4%. The accuracy of predicting pCR using the conventional PET parameters was up to 70.9%, and the accuracy of the physicians' assessment was 80.5%. The accuracy of the prediction from the ML model was significantly higher than those derived from conventional PET parameters and provided by physicians (p < 0.05). The ML model is useful for predicting pCR after neoadjuvant CCRT, which showed a higher predictive accuracy than those achieved from conventional PET parameters and from physicians.

C1 [Yoo, Jang; Cheon, Miju] Vet Hlth Serv Med Ctr, Dept Nucl Med, Seoul 05368, South Korea.

[Lee, Jaeho] Seoul Natl Univ, Dept Prevent Med, Coll Med, Seoul 03080, South Korea.

[Woo, Sang-Keun] Korea Inst Radiol & Med Sci KIRAMS, Korea Canc Ctr Hosp, Dept Nucl Med, Seoul 01812, South Korea.

[Ahn, Myung-Ju] Sungkyunkwan Univ, Samsung Med Ctr, Dept Med, Div Hematol Oncol,Sch Med, Seoul 06351, South Korea.

[Pyo, Hong Ryull] Sungkyunkwan Univ, Samsung Med Ctr, Dept Radiat Oncol, Sch Med, Seoul 06351, South Korea.

[Choi, Yong Soo] Sungkyunkwan Univ, Samsung Med Ctr, Dept Thorac & Cardiovasc Surg, Sch Med, Seoul 06351, South Korea.

[Han, Joung Ho] Sungkyunkwan Univ, Samsung Med Ctr, Dept Pathol, Sch Med, Seoul 06351, South Korea.

[Choi, Joon Young] Sungkyunkwan Univ, Samsung Med Ctr, Dept Nucl Med, Sch Med, Seoul 06351, South Korea.

RP Choi, JY (通讯作者)，Sungkyunkwan Univ, Samsung Med Ctr, Dept Nucl Med, Sch Med, Seoul 06351, South Korea.

EM jang8214.yoo@gmail.com; hoyajh21@gmail.com; diva1813@naver.com;

skwoo@kirams.re.kr; silk.ahn@samsung.com; hr.pyo@samsung.com;

ysooyah.choi@samsung.com; joungho.han@samsung.com; jynm.choi@samsung.com

OI Yoo, Jang/0000-0003-4664-4904; CHEON, MIJU/0000-0001-7469-7769; Woo,

Sang-Keun/0000-0002-6728-8876; Choi, Joon Young/0000-0003-1060-0096

FU National Research Foundation of Korea (NRF) - Korea government (Ministry

of Science and ICT) [NRF-2020M2D9A1094072]; Future Medicine 20*30

Project of the Samsung Medical Center [SMO1220071]; VHS Medical Center

Research Grant [VHSMC 22001]

FX This work was supported by the National Research Foundation of Korea

(NRF) grant funded by the Korea government (Ministry of Science and ICT)

(No. NRF-2020M2D9A1094072), Future Medicine 20*30 Project of the Samsung

Medical Center (#SMO1220071), and VHS Medical Center Research Grant (No.

VHSMC 22001).

CR Antunovic L, 2019, EUR J NUCL MED MOL I, V46, P1468, DOI 10.1007/s00259-019-04313-8

Arbour KC, 2019, JAMA-J AM MED ASSOC, V322, P764, DOI 10.1001/jama.2019.11058

Cerfolio RJ, 2004, ANN THORAC SURG, V78, P1903, DOI 10.1016/j.athoracsur.2004.06.102

Cottrell TR, 2018, ANN ONCOL, V29, P1853, DOI 10.1093/annonc/mdy218

Cremonesi M, 2017, EUR J NUCL MED MOL I, V44, P1915, DOI 10.1007/s00259-017-3762-9

D'Angelillo RM, 2009, J THORAC ONCOL, V4, P1517, DOI 10.1097/JTO.0b013e3181b9e860

De Ruysscher D, 2009, RADIOTHER ONCOL, V91, P415, DOI 10.1016/j.radonc.2009.01.004

DeSantis CE, 2014, CA-CANCER J CLIN, V64, P252, DOI 10.3322/caac.21235

Eun NL, 2020, RADIOLOGY, V294, P31, DOI 10.1148/radiol.2019182718

Ha S, 2017, SCI REP-UK, V7, DOI 10.1038/s41598-017-01524-7

Hoffmann B, 2019, METHODS MOL BIOL, V1878, P263, DOI 10.1007/978-1-4939-8868-6_16

Huang CM, 2020, SCI REP-UK, V10, DOI 10.1038/s41598-020-69345-9

Hyun OJ, 2016, RADIOLOGY, V280, P576, DOI 10.1148/radiol.2016142043

Hyun SH, 2015, AM J ROENTGENOL, V205, P623, DOI 10.2214/AJR.14.13847

Iravani A, 2019, EUR J NUCL MED MOL I, V46, P1869, DOI 10.1007/s00259-019-04388-3

Kim AW, 2011, ANN THORAC SURG, V92, P233, DOI 10.1016/j.athoracsur.2011.03.001

Kim HK, 2016, LUNG CANCER, V96, P56, DOI 10.1016/j.lungcan.2016.03.016

Li PL, 2020, EUR J NUCL MED MOL I, V47, P1116, DOI 10.1007/s00259-020-04684-3

Lo Gullo R, 2020, BREAST, V49, P115, DOI 10.1016/j.breast.2019.11.009

Meti N, 2021, JCO CLIN CANCER INFO, V5, P66, DOI 10.1200/CCI.20.00078

Mouillet G, 2012, J THORAC ONCOL, V7, P841, DOI 10.1097/JTO.0b013e31824c7d92

Pottgen C, 2013, EUR J CANCER, V49, P2107, DOI 10.1016/j.ejca.2013.02.030

Pottgen C, 2006, CLIN CANCER RES, V12, P97, DOI 10.1158/1078-0432.CCR-05-0510

Rami-Porta R, 2009, ANN THORAC CARDIOVAS, V15, P4

Roengvoraphoj O, 2018, STRAHLENTHER ONKOL, V194, P107, DOI 10.1007/s00066-017-1229-3

Schreiner W, 2018, J THORAC DIS, V10, P2795, DOI 10.21037/jtd.2018.05.68

Shen WC, 2020, ANN TRANSL MED, V8, DOI 10.21037/atm.2020.01.107

Shin S, 2020, J THORAC DIS, V12, P2602, DOI 10.21037/jtd.2020.03.23

Sollini M, 2017, SCI REP-UK, V7, DOI 10.1038/s41598-017-00426-y

Stupp R, 2009, LANCET ONCOL, V10, P785, DOI 10.1016/S1470-2045(09)70172-X

Szyszko TA, 2016, LUNG CANCER, V94, P7, DOI 10.1016/j.lungcan.2016.01.010

Tahmassebi A, 2019, INVEST RADIOL, V54, P110, DOI 10.1097/RLI.0000000000000518

Tanahashi M, 2020, J THORAC DIS, V12, P2644, DOI 10.21037/jtd.2020.03.17

Wahl RL, 2009, J NUCL MED, V50, p122S, DOI 10.2967/jnumed.108.057307

Yakar Melek, 2021, Technol Cancer Res Treat, V20, p15330338211016373, DOI 10.1177/15330338211016373

Yoo J, 2021, EUR RADIOL, V31, P4184, DOI 10.1007/s00330-020-07523-z

NR 36

TC 0

Z9 0

U1 3

U2 3

PU MDPI

PI BASEL

PA ST ALBAN-ANLAGE 66, CH-4052 BASEL, SWITZERLAND

EI 2072-6694

J9 CANCERS

JI Cancers

PD APR

PY 2022

VL 14

IS 8

AR 1987

DI 10.3390/cancers14081987

PG 12

WC Oncology

WE Science Citation Index Expanded (SCI-EXPANDED)

SC Oncology

GA 0T1UU

UT WOS:000786759800001

PM 35454899

OA gold, Green Published

DA 2022-08-24

ER

PT J

AU Blanc-Durand, P

Campedel, L

Mule, S

Jegou, S

Luciani, A

Pigneur, F

Itti, E

AF Blanc-Durand, Paul

Campedel, Luca

Mule, Sebastien

Jegou, Simon

Luciani, Alain

Pigneur, Frederic

Itti, Emmanuel

TI Prognostic value of anthropometric measures extracted from whole-body CT

using deep learning in patients with non-small-cell lung cancer

SO EUROPEAN RADIOLOGY

LA English

DT Article

DE Tomography; X-ray computed; Machine learning; Lung cancer; Adiposity

ID VISCERAL ADIPOSE-TISSUE; NETWORK; VOLUME

AB Introduction The aim of the study was to extract anthropometric measures from CT by deep learning and to evaluate their prognostic value in patients with non-small-cell lung cancer (NSCLC). Methods A convolutional neural network was trained to perform automatic segmentation of subcutaneous adipose tissue (SAT), visceral adipose tissue (VAT), and muscular body mass (MBM) from low-dose CT images in 189 patients with NSCLC who underwent pretherapy PET/CT. After a fivefold cross-validation in a subset of 35 patients, anthropometric measures extracted by deep learning were normalized to the body surface area (BSA) to control the various patient morphologies. VAT/SAT ratio and clinical parameters were included in a Cox proportional-hazards model for progression-free survival (PFS) and overall survival (OS). Results Inference time for a whole volume was about 3 s. Mean Dice similarity coefficients in the validation set were 0.95, 0.93, and 0.91 for SAT, VAT, and MBM, respectively. For PFS prediction, T-stage, N-stage, chemotherapy, radiation therapy, and VAT/SAT ratio were associated with disease progression on univariate analysis. On multivariate analysis, only N-stage (HR = 1.7 [1.2-2.4]; p = 0.006), radiation therapy (HR = 2.4 [1.0-5.4]; p = 0.04), and VAT/SAT ratio (HR = 10.0 [2.7-37.9]; p < 0.001) remained significant prognosticators. For OS, male gender, smoking status, N-stage, a lower SAT/BSA ratio, and a higher VAT/SAT ratio were associated with mortality on univariate analysis. On multivariate analysis, male gender (HR = 2.8 [1.2-6.7]; p = 0.02), N-stage (HR = 2.1 [1.5-2.9]; p < 0.001), and the VAT/SAT ratio (HR = 7.9 [1.7-37.1]; p < 0.001) remained significant prognosticators. Conclusion The BSA-normalized VAT/SAT ratio is an independent predictor of both PFS and OS in NSCLC patients.

C1 [Blanc-Durand, Paul; Itti, Emmanuel] Henri Mondor Hosp, AP HP, Dept Nucl Med, F-94010 Creteil, France.

[Blanc-Durand, Paul; Itti, Emmanuel] U PEC, Team 8, INSERM IMRB, F-94000 Creteil, France.

[Blanc-Durand, Paul; Mule, Sebastien; Luciani, Alain; Itti, Emmanuel] U PEC, F-94000 Creteil, France.

[Campedel, Luca] Grp Hosp Pitie Salpetriere C Foix, AP HP, Dept Oncol, F-75013 Paris, France.

[Mule, Sebastien; Luciani, Alain; Pigneur, Frederic] Henri Mondor Hosp, AP HP, Dept Radiol, F-94010 Creteil, France.

[Jegou, Simon] Owkin, F-75010 Paris, France.

RP Blanc-Durand, P (通讯作者)，Henri Mondor Hosp, AP HP, Dept Nucl Med, F-94010 Creteil, France.; Blanc-Durand, P (通讯作者)，U PEC, Team 8, INSERM IMRB, F-94000 Creteil, France.; Blanc-Durand, P (通讯作者)，U PEC, F-94000 Creteil, France.

EM paul.blancdurand@aphp.fr

RI Mulé, Sébastien/ABI-6590-2020

OI Itti, Emmanuel/0000-0003-1578-4058; Mule, Sebastien/0000-0002-6896-6149

CR Bakr S, 2018, SCI DATA, V5, DOI 10.1038/sdata.2018.202

Belharbi S, 2017, COMPUT BIOL MED, V87, P95, DOI 10.1016/j.compbiomed.2017.05.018

Bridge CP, 2018, LECT NOTES COMPUT SC, V11041, P204, DOI 10.1007/978-3-030-01201-4_22

Brown JC, 2018, EUR J NUTR, V57, P191, DOI 10.1007/s00394-016-1308-8

Buvat I, 2017, J NUCL MED, V58

C Nioche, 2016, MEADECINE NUCLEAAIRE, V40, P208, DOI DOI 10.1016/j.mednuc.2016.03.107

Chang PJ, 2019, RADIOLOGY, V290, P680, DOI 10.1148/radiol.2018182557

Cicek Ozgun, 2016, Medical Image Computing and Computer-Assisted Intervention - MICCAI 2016. 19th International Conference. Proceedings: LNCS 9901, P424, DOI 10.1007/978-3-319-46723-8_49

Clark K, 2013, J DIGIT IMAGING, V26, P1045, DOI 10.1007/s10278-013-9622-7

Decazes P, 2016, J NUCL MED, V57, P753, DOI 10.2967/jnumed.115.164913

Du Bois D, 1989, Nutrition, V5, P303

DUBOIS D, 1989, NUTRITION, V5, P303

Fidon L, 2018, LECT NOTES COMPUT SC, V10670, P64, DOI 10.1007/978-3-319-75238-9_6

Gibson E, 2018, COMPUT METH PROG BIO, V158, P113, DOI 10.1016/j.cmpb.2018.01.025

Hilmi M, 2019, PHARMACOL THERAPEUT, V196, P135, DOI 10.1016/j.pharmthera.2018.12.003

Hochhegger B, 2015, J BRAS PNEUMOL, V41, P264, DOI 10.1590/S1806-37132015000004479

Hopkins JJ, 2017, EXPERT REV CLIN PHAR, V10, P947, DOI 10.1080/17512433.2017.1347503

Hunter JD, 2007, COMPUT SCI ENG, V9, P90, DOI 10.1109/MCSE.2007.55

Jegou S, 2017, IEEE COMPUT SOC CONF, P1175, DOI 10.1109/CVPRW.2017.156

Klopp AH, 2012, CLIN CANCER RES, V18, P771, DOI 10.1158/1078-0432.CCR-11-1916

Lee H, 2017, J DIGIT IMAGING, V30, P487, DOI 10.1007/s10278-017-9988-z

Lee JW, 2018, CLIN IMAG, V50, P308, DOI 10.1016/j.clinimag.2018.05.006

Lewiner T., 2003, Journal of Graphics Tools, V8, P1, DOI 10.1080/10867651.2003.10487582

Litjens G, 2017, MED IMAGE ANAL, V42, P60, DOI 10.1016/j.media.2017.07.005

Mendez J, 1960, METABOLISM

Mensink SD, 2011, PROC SPIE, V7963, DOI 10.1117/12.878017

Nattenmuller J, 2017, PLOS ONE, V12, DOI 10.1371/journal.pone.0169136

Popinat G, 2019, ONCOIMMUNOLOGY, V8, DOI 10.1080/2162402X.2019.1580128

Schaudinn A, 2015, NMR BIOMED, V28, P583, DOI 10.1002/nbm.3286

Shachar SS, 2016, EUR J CANCER, V57, P58, DOI 10.1016/j.ejca.2015.12.030

Villa C, 2017, CLIN PHYSIOL FUNCT I, V37, P183, DOI 10.1111/cpf.12284

Wang YZ, 2017, COMPUT METH PROG BIO, V144, P97, DOI 10.1016/j.cmpb.2017.03.017

Weston AD, 2019, RADIOLOGY, V290, P669, DOI 10.1148/radiol.2018181432

NR 33

TC 9

Z9 10

U1 1

U2 8

PU SPRINGER

PI NEW YORK

PA ONE NEW YORK PLAZA, SUITE 4600, NEW YORK, NY, UNITED STATES

SN 0938-7994

EI 1432-1084

J9 EUR RADIOL

JI Eur. Radiol.

PD JUN

PY 2020

VL 30

IS 6

BP 3528

EP 3537

DI 10.1007/s00330-019-06630-w

EA FEB 2020

PG 10

WC Radiology, Nuclear Medicine & Medical Imaging

WE Science Citation Index Expanded (SCI-EXPANDED)

SC Radiology, Nuclear Medicine & Medical Imaging

GA LR8BV

UT WOS:000516098100002

PM 32055950

DA 2022-08-24

ER

PT J

AU Grgic, A

Nestle, U

Schaefer-Schuler, A

Kremp, S

Ballek, E

Fleckenstein, J

Rube, C

Kirsch, CM

Hellwig, D

AF Grgic, Aleksandar

Nestle, Ursula

Schaefer-Schuler, Andrea

Kremp, Stephanie

Ballek, Elena

Fleckenstein, Jochen

Ruebe, Christian

Kirsch, Carl-Martin

Hellwig, Dirk

TI Nonrigid Versus Rigid Registration of Thoracic F-18-FDG PET and CT in

Patients with Lung Cancer: An Intraindividual Comparison of Different

Breathing Maneuvers

SO JOURNAL OF NUCLEAR MEDICINE

LA English

DT Article

DE non-small cell lung carcinoma (NSCLC); spiral computed tomography;

positron emission tomography; image registration; computer-assisted

image analysis

ID WHOLE-BODY PET; FDG-PET; CO-REGISTRATION; 3-DIMENSIONAL REGISTRATION;

EMISSION-TOMOGRAPHY; IMAGE REGISTRATION; RESPIRATORY MOTION; CLINICAL

ONCOLOGY; RADIOTHERAPY; DELINEATION

AB In lung cancer, F-18-FDG PET, CT, and F-18-FDG PET/CT are used for noninvasive staging and therapy planning. Even with improved image registration techniques-especially in the modern hybrid PET/CT scanners-inaccuracies in the fusion process may occur, leading to errors in image interpretation. The aim of this study was to investigate by an intraindividual analysis whether, in comparison with a rigid algorithm, a nonrigid registration algorithm improves the quality of fusion between F-18-FDG PET and CT. Methods: Sixteen patients with histologically proven non-small cell lung cancer underwent a thoracic F-18-FDG PET acquisition in radiotherapy treatment position and 3 CT acquisitions (expiration, inspiration, and mid breath-hold) on the same day. All scans were registered with rigid and nonrigid procedures, resulting in 6 fused datasets: rigid inspiration, rigid expiration, rigid mid breath-hold, nonrigid inspiration, nonrigid expiration, and nonrigid mid breath-hold. The quality of alignment was assessed by 3 experienced readers at 8 anatomic landmarks: lung apices, aortic arch, heart, spine, sternum, carina, diaphragm, and tumor using an alignment score ranging from 1 (no alignment) to 5 (exact alignment). Results: Nonrigid PET/CT showed better alignment than rigid PET/CT (3.5 +/- 0.7 vs. 3.3 +/- 0.7, P < 0.001). Regarding the breathing maneuver, no difference between nonrigid mid breath-hold and rigid mid breath-hold was observed. In contrast, the alignment quality significantly improved from rigid expiration to nonrigid expiration (3.4 +/- 0.7 vs. 3.6 +/- 0.7, P < 0.001) and from rigid inspiration to nonrigid inspiration (3.1 +/- 0.7 vs. 3.3 +/- 0.7, P < 0.001). With regard to individual landmarks, an improvement in fusion quality through the use of nonrigid registration was obvious at the lung apices, carina, and aortic arch. Conclusion: The alignment quality of thoracic F-18-FDG PET/CT exhibits a marked dependence on the breathing maneuver performed during the CT acquisition, as demonstrated in an intraindividual comparison. Nonrigid registration is a significant improvement over rigid registration if the CT is performed during full inspiration or full expiration. The best fusion results are obtained with the CT performed at mid breath-hold using rigid registration, without an improvement using nonrigid algorithms.

C1 [Grgic, Aleksandar; Nestle, Ursula; Schaefer-Schuler, Andrea; Ballek, Elena; Kirsch, Carl-Martin; Hellwig, Dirk] Univ Saarland, Dept Nucl Med, Med Ctr, D-66421 Homburg, Germany.

[Nestle, Ursula] Univ Hosp Freiburg, Dept Radiooncol, Freiburg, Germany.

[Kremp, Stephanie; Fleckenstein, Jochen; Ruebe, Christian] Univ Saarland, Dept Radiooncol, Med Ctr, D-66421 Homburg, Germany.

RP Grgic, A (通讯作者)，Univ Saarland, Dept Nucl Med, Med Ctr, Kirrbergerstr 1,Gebaude 50, D-66421 Homburg, Germany.

EM aleksandar.grgic@uks.eu

RI Hellwig, Dirk/O-8617-2019; Nestle, Ursula/ABG-2339-2021; Hellwig,

Dirk/A-4128-2008

OI Hellwig, Dirk/0000-0002-3056-0143;

CR Aquino SL, 2003, J COMPUT ASSIST TOMO, V27, P479, DOI 10.1097/00004728-200307000-00004

Beyer T, 2000, J NUCL MED, V41, P1369

Beyer T, 2003, EUR J NUCL MED MOL I, V30, P588, DOI 10.1007/s00259-002-1097-6

Bilfinger Thomas V, 2003, Respir Care Clin N Am, V9, P141, DOI 10.1016/S1078-5337(02)00086-2

Bridges RL, 2009, J NUCL MED, V50, P835, DOI 10.2967/jnumed.108.055574

Fitton I, 2008, INT J RADIAT ONCOL, V70, P1403, DOI 10.1016/j.ijrobp.2007.08.063

Fleiss J. L, 1981, STAT METHODS RATES P

Gilman MD, 2007, J COMPUT ASSIST TOMO, V31, P395, DOI 10.1097/01.rct.0000237817.18678.9c

Gilman MD, 2006, AM J ROENTGENOL, V187, P1357, DOI 10.2214/AJR.05.1427

Goerres GW, 2002, EUR J NUCL MED MOL I, V29, P351, DOI 10.1007/s00259-001-0710-4

Gould KL, 2007, J NUCL MED, V48, P1112, DOI 10.2967/jnumed.107.039792

Grgic A, 2009, INT J RADIAT ONCOL, V73, P103, DOI 10.1016/j.ijrobp.2008.03.063

Halpern BS, 2005, CHEST, V128, P2289, DOI 10.1016/S0012-3692(15)52634-2

Hellwig D, 2009, NUKLEARMED-NUCL MED, V48, P59, DOI 10.3413/nukmed-0217

Ireland RH, 2007, INT J RADIAT ONCOL, V68, P952, DOI 10.1016/j.ijrobp.2007.02.017

Jemal A, 2008, JNCI-J NATL CANCER I, V100, P1672, DOI 10.1093/jnci/djn389

Krishnasetty V, 2005, RADIOLOGY, V237, P635, DOI 10.1148/radiol.2372041719

Lamare F, 2007, PHYS MED BIOL, V52, P121, DOI 10.1088/0031-9155/52/1/009

Moreno A, 2008, COMPUT AIDED SURG, V13, P281, DOI 10.3109/10929080802431980

Nehmeh SA, 2002, J NUCL MED, V43, P876

PELIZZARI CA, 1989, J COMPUT ASSIST TOMO, V13, P20, DOI 10.1097/00004728-198901000-00004

Pietrzyk U, 2005, NUKLEARMED-NUCL MED, V44, pS13

Schaefer A, 2008, EUR J NUCL MED MOL I, V35, P1989, DOI 10.1007/s00259-008-0875-1

Shekhar R, 2005, J NUCL MED, V46, P1488

Slomka PJ, 2003, J NUCL MED, V44, P1156

Ukena D, 2004, LUNG CANCER, V45, pS75, DOI 10.1016/j.lungcan.2004.07.989

Weigert M, 2008, Z MED PHYS, V18, P59, DOI 10.1016/j.zemedi.2007.07.004

West J, 1997, J COMPUT ASSIST TOMO, V21, P554, DOI 10.1097/00004728-199707000-00007

Wolz G, 2007, NUKLEARMED-NUCL MED, V46, P43, DOI 10.1055/s-0037-1616625

Wolz G, 2007, INT J COMPUT ASS RAD, V2, P183, DOI 10.1007/s11548-007-0128-y

NR 30

TC 17

Z9 17

U1 0

U2 2

PU SOC NUCLEAR MEDICINE INC

PI RESTON

PA 1850 SAMUEL MORSE DR, RESTON, VA 20190-5316 USA

SN 0161-5505

J9 J NUCL MED

JI J. Nucl. Med.

PD DEC

PY 2009

VL 50

IS 12

BP 1921

EP 1926

DI 10.2967/jnumed.109.065649

PG 6

WC Radiology, Nuclear Medicine & Medical Imaging

WE Science Citation Index Expanded (SCI-EXPANDED)

SC Radiology, Nuclear Medicine & Medical Imaging

GA 529YM

UT WOS:000272555300006

PM 19910420

OA Bronze

DA 2022-08-24

ER

PT J

AU Hosny, A

Parmar, C

Coroller, TP

Grossmann, P

Zeleznik, R

Kumar, A

Bussink, J

Gillies, RJ

Mak, RH

Aerts, HJWL

AF Hosny, Ahmed

Parmar, Chintan

Coroller, Thibaud P.

Grossmann, Patrick

Zeleznik, Roman

Kumar, Avnish

Bussink, Johan

Gillies, Robert J.

Mak, Raymond H.

Aerts, Hugo J. W. L.

TI Deep learning for lung cancer prognostication: A retrospective

multi-cohort radiomics study

SO PLOS MEDICINE

LA English

DT Article

ID CONVOLUTIONAL NEURAL-NETWORKS; PHENOTYPE FEATURES; TUMOR MEASUREMENTS;

SURVIVAL; BIOMARKERS; LOCATION; CLASSIFICATION; VARIABILITY; EDITION;

PACKAGE

AB Background

Non-small-cell lung cancer (NSCLC) patients often demonstrate varying clinical courses and outcomes, even within the same tumor stage. This study explores deep learning applications in medical imaging allowing for the automated quantification of radiographic characteristics and potentially improving patient stratification.

Methods and findings

We performed an integrative analysis on 7 independent datasets across 5 institutions totaling 1,194 NSCLC patients (age median = 68.3 years [range 32.5-93.3], survival median = 1.7 years [range 0.0-11.7]). Using external validation in computed tomography (CT) data, we identified prognostic signatures using a 3D convolutional neural network (CNN) for patients treated with radiotherapy (n = 771, age median = 68.0 years [range 32.5-93.3], survival median = 1.3 years [range 0.0-11.7]). We then employed a transfer learning approach to achieve the same for surgery patients (n = 391, age median = 69.1 years [range 37.2-88.0], survival median = 3.1 years [range 0.0-8.8]). We found that the CNN predictions were significantly associated with 2-year overall survival from the start of respective treatment for radiotherapy (area under the receiver operating characteristic curve [AUC] = 0.70 [95% CI 0.63-0.78], p < 0.001) and surgery (AUC = 0.71 [95% CI 0.60-0.82], p < 0.001) patients. The CNN was also able to significantly stratify patients into low and high mortality risk groups in both the radiotherapy (p < 0.001) and surgery (p = 0.03) datasets. Additionally, the CNN was found to significantly outperform random forest models built on clinical parameters-including age, sex, and tumor node metastasis stage-as well as demonstrate high robustness against test-retest (intraclass correlation coefficient = 0.91) and inter-reader (Spearman's rank-order correlation = 0.88) variations. To gain a better understanding of the characteristics captured by the CNN, we identified regions with the most contribution towards predictions and highlighted the importance of tumor-surrounding tissue in patient stratification. We also present preliminary findings on the biological basis of the captured phenotypes as being linked to cell cycle and transcriptional processes. Limitations include the retrospective nature of this study as well as the opaque black box nature of deep learning networks.

Conclusions

Our results provide evidence that deep learning networks may be used for mortality risk stratification based on standard-of-care CT images from NSCLC patients. This evidence motivates future research into better deciphering the clinical and biological basis of deep learning networks as well as validation in prospective data.

C1 [Hosny, Ahmed; Parmar, Chintan; Coroller, Thibaud P.; Grossmann, Patrick; Zeleznik, Roman; Kumar, Avnish; Aerts, Hugo J. W. L.] Harvard Med Sch, Brigham & Womens Hosp, Dana Farber Canc Inst, Dept Radiat Oncol, Boston, MA 02115 USA.

[Bussink, Johan] Radboud Univ Nijmegen, Med Ctr, Dept Radiat Oncol, Nijmegen, Netherlands.

[Gillies, Robert J.] H Lee Moffitt Canc Ctr & Res Inst, Dept Canc Physiol, Tampa, FL USA.

[Mak, Raymond H.; Aerts, Hugo J. W. L.] Harvard Med Sch, Brigham & Womens Hosp, Dept Radiol, Boston, MA 02115 USA.

RP Aerts, HJWL (通讯作者)，Harvard Med Sch, Brigham & Womens Hosp, Dana Farber Canc Inst, Dept Radiat Oncol, Boston, MA 02115 USA.; Aerts, HJWL (通讯作者)，Harvard Med Sch, Brigham & Womens Hosp, Dept Radiol, Boston, MA 02115 USA.

EM Hugo_Aerts@dfci.harvard.edu

RI parmar, chintan/J-2977-2019; Aerts, Hugo/ABF-2821-2020; Bussink,

Jan/N-3584-2014

OI parmar, chintan/0000-0002-2140-814X; Aerts, Hugo/0000-0002-2122-2003;

Gillies, Robert/0000-0002-8888-7747; Kumar, Avnish/0000-0001-5882-748X;

Mak, Raymond/0000-0002-8754-0565; Bussink, Johan/0000-0002-5751-4796;

Coroller, Thibaud/0000-0001-7662-8724

FU National Institute of Health [NIH-USA U24CA194354, NIH-USA U01CA190234];

NATIONAL CANCER INSTITUTE [U24CA194354, U01CA190234] Funding Source: NIH

RePORTER

FX Authors acknowledge financial support from the National Institute of

Health (NIH-USA U24CA194354, and NIH-USA U01CA190234);

https://grants.nih.gov/funding/index.htm.The funders had no role in

study design, data collection and analysis, decision to publish, or

preparation of the manuscript.

CR Abadi Martin, 2016, arXiv

Aerts HJWL, 2014, NAT COMMUN, V5, DOI 10.1038/ncomms5006

Ahrendt SA, 2003, J NATL CANCER I, V95, P961, DOI 10.1093/jnci/95.13.961

ALBERTI W, 1995, BRIT MED J, V311, P899

American Cancer Society, 2017, CANC FACTS FIG 2016

Amin MB, 2017, CA-CANCER J CLIN, V67, P93, DOI 10.3322/caac.21388

Arriagada R, 2010, J CLIN ONCOL, V28, P35, DOI 10.1200/JCO.2009.23.2272

Bai HX, 2016, BRIT J RADIOL, V89, DOI 10.1259/bjr.20151030

BENJAMINI Y, 1995, J R STAT SOC B, V57, P289, DOI 10.1111/j.2517-6161.1995.tb02031.x

Burotto M, 2014, J THORAC ONCOL, V9, P1609, DOI 10.1097/JTO.0000000000000302

Burrell RA, 2013, NATURE, V501, P338, DOI 10.1038/nature12625

Carneiro G, 2017, I S BIOMED IMAGING, P130, DOI 10.1109/ISBI.2017.7950485

Cistaro A, 2013, RADIOL ONCOL, V47, P219, DOI 10.2478/raon-2013-0023

Coroller TP, 2016, RADIOTHER ONCOL, V119, P480, DOI 10.1016/j.radonc.2016.04.004

Coroller TP, 2015, RADIOTHER ONCOL, V114, P345, DOI 10.1016/j.radonc.2015.02.015

Cruz-Roa A, 2017, SCI REP-UK, V7, DOI 10.1038/srep46450

De Jay N, 2013, BIOINFORMATICS, V29, P2365, DOI 10.1093/bioinformatics/btt383

Egeblad M, 2010, DEV CELL, V18, P884, DOI 10.1016/j.devcel.2010.05.012

El-Hachem N, 2016, ENVIRON HEALTH PERSP, V124, P313, DOI 10.1289/ehp.1409157

ESR, 2011, INSIGHTS IMAGING, V2, P621, DOI 10.1007/s13244-011-0125-0

Esteva A, 2017, NATURE, V542, P115, DOI 10.1038/nature21056

Finlayson S. G., 2018, ARXIV PREPRINT ARXIV

Forsberg D, 2017, J DIGIT IMAGING, V30, P406, DOI 10.1007/s10278-017-9945-x

Ganeshan B, 2013, RADIOLOGY, V266, P326, DOI 10.1148/radiol.12112428

Ganeshan B, 2010, CANCER IMAGING, V10, P137, DOI 10.1102/1470-7330.2010.0021

Ghafoorian M, 2017, SCI REP-UK, V7, DOI 10.1038/s41598-017-05300-5

Ghafoorian M, 2017, NEUROIMAGE-CLIN, V14, P391, DOI 10.1016/j.nicl.2017.01.033

Gospodarowicz MK, 2004, CANCER, V100, P1, DOI 10.1002/cncr.11898

Grossmann P, 2017, ELIFE, V6, DOI 10.7554/eLife.23421

Grossmann P, 2016, BMC CANCER, V16, DOI 10.1186/s12885-016-2659-5

Gulshan V, 2016, JAMA-J AM MED ASSOC, V316, P2402, DOI 10.1001/jama.2016.17216

Hammernik K., 2017, BILDVERARBEITUNG MED, V2017, P92, DOI DOI 10.1007/978-3-662-54345-0_25

Hoang T, 2005, J CLIN ONCOL, V23, P175, DOI 10.1200/JCO.2005.04.177

Hosny A, 2018, NAT REV CANCER, V18, P500, DOI 10.1038/s41568-018-0016-5

Huynh E, 2016, RADIOTHER ONCOL, V120, P258, DOI 10.1016/j.radonc.2016.05.024

Ioffe S., 2015, P INT C MACH LEARN L, V37, P448, DOI 10.5555/3045118.3045167

Irizarry RA, 2003, BIOSTATISTICS, V4, P249, DOI 10.1093/biostatistics/4.2.249

Kim H, 2017, PLOS ONE, V12, DOI 10.1371/journal.pone.0187500

Kingma D, 2014, ARXIV

Kooi T, 2017, MED IMAGE ANAL, V35, P303, DOI 10.1016/j.media.2016.07.007

Kotikalapudi R, 2018, KERAS VIS

Krizhevsky A., 2012, ADV NEURAL INFORM PR, V25, DOI DOI 10.1145/3065386

Kuhn M, 2008, J STAT SOFTW, V28, P1, DOI 10.18637/jss.v028.i05

Lambin P, 2017, NAT REV CLIN ONCOL, V14, P749, DOI 10.1038/nrclinonc.2017.141

Lambin P, 2012, EUR J CANCER, V48, P441, DOI 10.1016/j.ejca.2011.11.036

Lao JW, 2017, SCI REP-UK, V7, DOI 10.1038/s41598-017-10649-8

Liberzon A, 2011, BIOINFORMATICS, V27, P1739, DOI 10.1093/bioinformatics/btr260

Litjens G, 2017, MED IMAGE ANAL, V42, P60, DOI 10.1016/j.media.2017.07.005

Long J, 2015, PROC CVPR IEEE, P3431, DOI 10.1109/CVPR.2015.7298965

Lundstrom CF, 2017, RADIOLOGY, V285, P12, DOI 10.1148/radiol.2017170062

Maas A.L., 2013, P ICML, V30, P3

Miao S, 2016, IEEE T MED IMAGING, V35, P1352, DOI 10.1109/TMI.2016.2521800

Milletari F, 2016, INT CONF 3D VISION, P565, DOI 10.1109/3DV.2016.79

Mirsadraee S, 2012, WORLD J RADIOL, V4, P128, DOI 10.4329/wjr.v4.i4.128

Molina JR, 2008, MAYO CLIN PROC, V83, P584, DOI 10.4065/83.5.584

Ng A. Y, 2004, PROC 21 INT C MACH L, P78, DOI DOI 10.1145/1015330.1015435

Ngiam J., P 28 INT C MACH LEAR, P689, DOI DOI 10.5555/3104482.3104569

Oberije C, 2014, RADIOTHER ONCOL, V112, P37, DOI 10.1016/j.radonc.2014.04.012

OECD iLibrary, 2018, HLTH EQ COMP TOM CT

OECD iLibrary, 2018, HLTH CAR US COMP TOM

Oikonomou A, 2018, SCI REP-UK, V8, DOI 10.1038/s41598-018-22357-y

Oxnard GR, 2011, J CLIN ONCOL, V29, P3114, DOI 10.1200/JCO.2010.33.7071

Pan SJ, 2010, IEEE T KNOWL DATA EN, V22, P1345, DOI 10.1109/TKDE.2009.191

Pan YH, 2015, IEEE ENG MED BIO, P699, DOI 10.1109/EMBC.2015.7318458

Parkin DM, 2005, CA-CANCER J CLIN, V55, P74, DOI 10.3322/canjclin.55.2.74

Parmar C, 2015, SCI REP-UK, V5, DOI 10.1038/srep13087

Paul R, 2016, TOMOGRAPHY, V2, P388, DOI 10.18383/j.tom.2016.00211

Pepek JM, 2011, J THORAC ONCOL, V6, P757, DOI 10.1097/JTO.0b013e31821038c0

Prechelt L, 1998, LECT NOTES COMPUT SC, V1524, P55

Schroder MS, 2011, BIOINFORMATICS, V27, P3206, DOI 10.1093/bioinformatics/btr511

Sculier JP, 2008, J THORAC ONCOL, V3, P457, DOI 10.1097/JTO.0b013e31816de2b8

Selvaraju RR, 2020, INT J COMPUT VISION, V128, P336, DOI 10.1007/s11263-019-01228-7

Shien K, 2017, J THORAC DIS, V9, pE489, DOI 10.21037/jtd.2017.03.183

Shwartz-Ziv Ravid, 2017, ARXIV170300810

Srivastava N, 2014, J MACH LEARN RES, V15, P1929

Subramanian A, 2005, P NATL ACAD SCI USA, V102, P15545, DOI 10.1073/pnas.0506580102

Thakur MK, 2016, SEMIN RESP CRIT CARE, V37, P760, DOI 10.1055/s-0036-1592337

Uramoto H, 2014, TRANSL LUNG CANCER R, V3, P242, DOI 10.3978/j.issn.2218-6751.2013.12.05

van Griethuysen JJM, 2017, CANCER RES, V77, pE104, DOI 10.1158/0008-5472.CAN-17-0339

Wang XQ, 2017, SCI REP-UK, V7, DOI 10.1038/s41598-017-04963-4

Wu CF, 2015, MEDICINE, V94, DOI 10.1097/MD.0000000000001337

Yang X, 2017, NEUROIMAGE, V158, P378, DOI 10.1016/j.neuroimage.2017.07.008

Yuan X, 2017, ARXIV171207107

Zappa C, 2016, TRANSL LUNG CANCER R, V5, P288, DOI 10.21037/tlcr.2016.06.07

Zhang B, 2017, CANCER LETT, V403, P21, DOI 10.1016/j.canlet.2017.06.004

Zhang J, 2012, J CLIN ONCOL, V30, DOI 10.1200/jco.2012.30.30_suppl.81

Zhang QS, 2018, FRONT INFORM TECH EL, V19, P27, DOI 10.1631/FITEE.1700808

Zhao BS, 2009, RADIOLOGY, V252, P263, DOI 10.1148/radiol.2522081593

Zheng YF, 2015, LECT NOTES COMPUT SC, V9349, P565, DOI 10.1007/978-3-319-24553-9_69

NR 89

TC 212

Z9 222

U1 21

U2 70

PU PUBLIC LIBRARY SCIENCE

PI SAN FRANCISCO

PA 1160 BATTERY STREET, STE 100, SAN FRANCISCO, CA 94111 USA

SN 1549-1277

EI 1549-1676

J9 PLOS MED

JI PLos Med.

PD NOV

PY 2018

VL 15

IS 11

AR e1002711

DI 10.1371/journal.pmed.1002711

PG 25

WC Medicine, General & Internal

WE Science Citation Index Expanded (SCI-EXPANDED)

SC General & Internal Medicine

GA HC5FB

UT WOS:000451827800025

PM 30500819

OA gold, Green Published, Green Submitted

DA 2022-08-24

ER

PT J

AU Huang, ZH

Hu, C

Chi, CX

Jiang, Z

Tong, YX

Zhao, CL

AF Huang, Zhangheng

Hu, Chuan

Chi, Changxing

Jiang, Zhe

Tong, Yuexin

Zhao, Chengliang

TI An Artificial Intelligence Model for Predicting 1-Year Survival of Bone

Metastases in Non-Small-Cell Lung Cancer Patients Based on XGBoost

Algorithm

SO BIOMED RESEARCH INTERNATIONAL

LA English

DT Article

ID PROGNOSTIC-FACTORS; MULTICENTER

AB Non-small-cell lung cancer (NSCLC) patients often develop bone metastases (BM), and the overall survival for these patients is usually perishing. However, a model with high accuracy for predicting the survival of NSCLC with BM is still lacking. Here, we aimed to establish a model based on artificial intelligence for predicting the 1-year survival rate of NSCLC with BM by using extreme gradient boosting (XGBoost), a large-scale machine learning algorithm. We selected NSCLC patients with BM between 2010 and 2015 from the Surveillance, Epidemiology, and End Results database. In total, 5973 cases were enrolled and divided into the training (n = 4183) and validation (n = 1790) sets. XGBoost, random forest, support vector machine, and logistic algorithms were used to generate predictive models. Receiver operating characteristic curves were used to evaluate and compare the predictive performance of each model. The parameters including tumor size, age, race, sex, primary site, histological subtype, grade, laterality, T stage, N stage, surgery, radiotherapy, chemotherapy, distant metastases to other sites (lung, brain, and liver), and marital status were selected to construct all predictive models. The XGBoost model had a better performance in both training and validation sets as compared with other models in terms of accuracy. Our data suggested that the XGBoost model is the most precise and personalized tool for predicting the 1-year survival rate for NSCLC patients with BM. This model can help the clinicians to design more rational and effective therapeutic strategies.

C1 [Huang, Zhangheng; Hu, Chuan; Tong, Yuexin; Zhao, Chengliang] Chengde Med Univ, Affiliated Hosp, Dept Spine Surg, Chengde, Hebei, Peoples R China.

[Hu, Chuan] Qingdao Univ, Dept Orthoped, Affiliated Hosp, Qingdao, Shandong, Peoples R China.

[Chi, Changxing] Kunming Med Univ, Dept Radiotherapy, Affiliated Hosp 3, Kunming, Yunnan, Peoples R China.

[Jiang, Zhe] Jilin Univ, Sch Publ Hlth, Changchun, Jilin, Peoples R China.

RP Zhao, CL (通讯作者)，Chengde Med Univ, Affiliated Hosp, Dept Spine Surg, Chengde, Hebei, Peoples R China.

EM 38221965@qq.com

CR Altorki NK, 2019, NAT REV CANCER, V19, P9, DOI 10.1038/s41568-018-0081-9

Asamura H, 2008, J THORAC ONCOL, V3, P46, DOI 10.1097/JTO.0b013e31815e8577

Balachandran VP, 2015, LANCET ONCOL, V16, pE173, DOI 10.1016/S1470-2045(14)71116-7

Cappuzzo F, 2010, LANCET ONCOL, V11, P521, DOI 10.1016/S1470-2045(10)70112-1

Chansky K, 2009, J THORAC ONCOL, V4, P792, DOI 10.1097/JTO.0b013e3181a7716e

Chen LL, 2009, BRIT J CANCER, V101, P749, DOI 10.1038/sj.bjc.6605214

Cho BC, 2019, ANTICANCER RES, V39, P1403, DOI 10.21873/anticanres.13255

Choi SW, 2019, HEALTHC INFORM RES, V25, P305, DOI 10.4258/hir.2019.25.4.305

Decroisette C, 2011, J THORAC ONCOL, V6, P576, DOI 10.1097/JTO.0b013e318206a1e3

Deng JQ, 2018, CANCER MANAG RES, V10, P6143, DOI 10.2147/CMAR.S183878

Esposito M, 2014, PHARMACOL THERAPEUT, V141, P222, DOI 10.1016/j.pharmthera.2013.10.006

Fukui T, 2015, GEN THORAC CARDIOVAS, V63, P507, DOI 10.1007/s11748-015-0564-5

Hu CA, 2020, BMJ OPEN, V10, DOI 10.1136/bmjopen-2019-033898

Kazem MA, 2017, SURG-J R COLL SURG E, V15, P93, DOI 10.1016/j.surge.2016.06.002

Lababede O, 2018, ONCOLOGIST, V23, P844, DOI 10.1634/theoncologist.2017-0659

Langley RR, 2011, INT J CANCER, V128, P2527, DOI 10.1002/ijc.26031

LeVasseur N, 2016, CANCER TREAT REV, V50, P183, DOI 10.1016/j.ctrv.2016.09.013

Li H, 2019, EUR J CARDIO-THORAC, V55, P1121, DOI 10.1093/ejcts/ezy439

Liu WM, 2018, CLIN EXP METASTAS, V35, P753, DOI 10.1007/s10585-018-9943-5

Morgensztern D, 2010, J THORAC ONCOL, V5, P29, DOI 10.1097/JTO.0b013e3181c5920c

Ogunleye A, 2020, IEEE ACM T COMPUT BI, V17, P2131, DOI 10.1109/TCBB.2019.2911071

Pruksakorn D, 2018, J BONE ONCOL, V10, P1, DOI 10.1016/j.jbo.2017.10.001

Riihimaki M, 2014, LUNG CANCER, V86, P78, DOI 10.1016/j.lungcan.2014.07.020

Sathiakumar Nalini, 2013, Lung India, V30, P20, DOI 10.4103/0970-2113.106127

Sculier JP, 2008, J THORAC ONCOL, V3, P457, DOI 10.1097/JTO.0b013e31816de2b8

Song Q, 2019, J CANCER RES CLIN, V145, P737, DOI 10.1007/s00432-018-02826-7

Sugiura H, 2008, CLIN ORTHOP RELAT R, V466, P729, DOI 10.1007/s11999-007-0051-0

Tamayo D, 2016, ASTROPHYS J LETT, V832, DOI 10.3847/2041-8205/832/2/L22

Thatcher N, 2005, LANCET, V366, P1527, DOI 10.1016/S0140-6736(05)67625-8

Torlay L, 2017, Brain Inform, V4, P159, DOI 10.1007/s40708-017-0065-7

Wang B, 2019, J BONE ONCOL, V17, DOI 10.1016/j.jbo.2019.100251

Wang Y, 2019, FUTURE ONCOL, V15, P3395, DOI 10.2217/fon-2019-0007

Zhang L, 2017, MED SCI MONITOR, V23, DOI 10.12659/MSM.902971

Zheng XQ, 2019, TRANSL LUNG CANCER R, V8, P367, DOI 10.21037/tlcr.2019.08.16

NR 34

TC 9

Z9 9

U1 1

U2 6

PU HINDAWI LTD

PI LONDON

PA ADAM HOUSE, 3RD FLR, 1 FITZROY SQ, LONDON, W1T 5HF, ENGLAND

SN 2314-6133

EI 2314-6141

J9 BIOMED RES INT

JI Biomed Res. Int.

PD JUN 28

PY 2020

VL 2020

AR 3462363

DI 10.1155/2020/3462363

PG 13

WC Biotechnology & Applied Microbiology; Medicine, Research & Experimental

WE Science Citation Index Expanded (SCI-EXPANDED)

SC Biotechnology & Applied Microbiology; Research & Experimental Medicine

GA MQ3MC

UT WOS:000552799900003

PM 32685470

OA Green Published, gold

DA 2022-08-24

ER

PT J

AU Li, HM

Galperin-Aizenberg, M

Pryma, D

Simone, CB

Fan, Y

AF Li, Hongming

Galperin-Aizenberg, Maya

Pryma, Daniel

Simone, Charles B., II

Fan, Yong

TI Unsupervised machine learning of radiomic features for predicting

treatment response and overall survival of early stage non-small cell

lung cancer patients treated with stereotactic body radiation therapy

SO RADIOTHERAPY AND ONCOLOGY

LA English

DT Article

DE Unsupervised machine learning; Radiomics; Non-small cell lung cancer;

Stereotactic body radiation therapy

ID FDG-PET; QUANTITATIVE IMAGE; BIOMARKERS; SIGNATURE; EVENTS

AB Background and purpose: To predict treatment response and survival of NSCLC patients receiving stereotactic body radiation therapy (SBRT), we develop an unsupervised machine learning method for stratifying patients and extracting meta-features simultaneously based on imaging data.

Material and methods: This study was performed based on an F-18-FDG-PET dataset of 100 consecutive patients who were treated with SBRT for early stage NSCLC. Each patient's tumor was characterized by 722 radiomic features. An unsupervised two-way clustering method was used to identify groups of patients and radiomic features simultaneously. The groups of patients were compared in terms of survival and freedom from nodal failure. Meta-features were computed for building survival models to predict survival and free of nodal failure.

Results: Differences were found between 2 groups of patients when the patients were clustered into 3 groups in terms of both survival (p = 0.003) and freedom from nodal failure (p = 0.038). Average concordance measures for predicting survival and nodal failure were 0.640 +/- 0.029 and 0.664 +/- 0.063 respectively, better than those obtained by prediction models built upon clinical variables (p < 0.04).

Conclusions: The evaluation results demonstrate that our method allows us to stratify patients and predict survival and freedom from nodal failure with better performance than current alternative methods. (C) 2018 Elsevier B.V. All rights reserved.

C1 [Li, Hongming; Galperin-Aizenberg, Maya; Pryma, Daniel; Fan, Yong] Univ Penn, Dept Radiol, Perelman Sch Med, Philadelphia, PA 19104 USA.

[Simone, Charles B., II] Univ Maryland, Sch Med, Maryland Proton Treatment Ctr, Baltimore, MD 21201 USA.

RP Fan, Y (通讯作者)，Richards Bldg,7th Floor,RM D703, Philadelphia, PA 19104 USA.

EM yong.fan@ieee.org

RI Fan, Yong/O-4412-2014

OI Fan, Yong/0000-0001-9869-4685; Simone, Charles/0000-0002-0867-3694

FU National Institutes of Health [CA223358, CA189523, EB022573, DK114786,

DA039215, DA039002]; Precision Lung Radiotherapy Grant of the University

of Pennsylvania; NATIONAL CANCER INSTITUTE [R21CA223358, U24CA189523]

Funding Source: NIH RePORTER; NATIONAL INSTITUTE OF BIOMEDICAL IMAGING

AND BIOENGINEERING [R01EB022573] Funding Source: NIH RePORTER; NATIONAL

INSTITUTE OF DIABETES AND DIGESTIVE AND KIDNEY DISEASES [P50DK114786]

Funding Source: NIH RePORTER; NATIONAL INSTITUTE ON DRUG ABUSE

[R01DA039215, U54DA039002] Funding Source: NIH RePORTER

FX This work was supported in part by National Institutes of Health grants

[grant numbers CA223358, CA189523, EB022573, DK114786, DA039215, and

DA039002] and Precision Lung Radiotherapy Grant of the University of

Pennsylvania.

CR Austin PC, 2017, J CLIN EPIDEMIOL, V83, P75, DOI 10.1016/j.jclinepi.2016.11.017

Chen BJ, 2017, RADIAT ONCOL, V12, DOI 10.1186/s13014-017-0885-x

Cistaro A, 2013, RADIOL ONCOL, V47, P219, DOI 10.2478/raon-2013-0023

Constanzo J, 2017, TRANSL LUNG CANCER R, V6, P635, DOI 10.21037/tlcr.2017.09.07

Coroller TP, 2016, RADIOTHER ONCOL, V119, P480, DOI 10.1016/j.radonc.2016.04.004

Coroller TP, 2015, RADIOTHER ONCOL, V114, P345, DOI 10.1016/j.radonc.2015.02.015

Davatzikos C, 2018, J MED IMAGING, V5, DOI 10.1117/1.JMI.5.1.011018

Demsar J, 2006, J MACH LEARN RES, V7, P1

Desseroit MC, 2016, EUR J NUCL MED MOL I, V43, P1477, DOI 10.1007/s00259-016-3325-5

Emaminejad N, 2016, IEEE T BIO-MED ENG, V63, P1034, DOI 10.1109/TBME.2015.2477688

Fu LP, 2012, PLOS ONE, V7, DOI 10.1371/journal.pone.0050914

Gillies RJ, 2016, RADIOLOGY, V278, P563, DOI 10.1148/radiol.2015151169

Grady L, 2006, IEEE T PATTERN ANAL, V28, P1768, DOI 10.1109/TPAMI.2006.233

Grossmann P, 2017, ELIFE, V6, DOI 10.7554/eLife.23421

Hatt M, 2017, EUR J NUCL MED MOL I, V44, P151, DOI 10.1007/s00259-016-3427-0

Hawkins SH, 2014, IEEE ACCESS, V2, P1418, DOI 10.1109/ACCESS.2014.2373335

Hotelling H, 1933, J EDUC PSYCHOL, V24, P417, DOI 10.1037/h0071325

Huang YQ, 2016, RADIOLOGY, V281, P947, DOI 10.1148/radiol.2016152234

Huynh E, 2016, RADIOTHER ONCOL, V120, P258, DOI 10.1016/j.radonc.2016.05.024

KAPLAN EL, 1958, J AM STAT ASSOC, V53, P457, DOI 10.2307/2281868

Kumar V, 2012, MAGN RESON IMAGING, V30, P1234, DOI 10.1016/j.mri.2012.06.010

Lambin P, 2012, EUR J CANCER, V48, P441, DOI 10.1016/j.ejca.2011.11.036

Lee G, 2017, EUR J RADIOL, V86, P297, DOI 10.1016/j.ejrad.2016.09.005

Leijenaar RTH, 2015, SCI REP-UK, V5, DOI 10.1038/srep11075

Li H, 2017, INT J RADIAT ONCOL, V99, pS34, DOI 10.1016/j.ijrobp.2017.06.092

Li H, 2011, SEGMENTATION BRAIN T, P606

Li HM, 2012, 2012 9TH IEEE INTERNATIONAL SYMPOSIUM ON BIOMEDICAL IMAGING (ISBI), P1715, DOI 10.1109/ISBI.2012.6235910

Li Q, 2017, RADIAT ONCOL, V12, DOI 10.1186/s13014-017-0892-y

Li Q, 2017, MED PHYS, V44, P4341, DOI 10.1002/mp.12309

Lian CF, 2016, MED IMAGE ANAL, V32, P257, DOI 10.1016/j.media.2016.05.007

Liu Y, 2016, CLIN LUNG CANCER, V17, P441, DOI 10.1016/j.cllc.2016.02.001

Lovinfosse P, 2016, EUR J NUCL MED MOL I, V43, P1453, DOI 10.1007/s00259-016-3314-8

MANTEL NATHAN, 1966, CANCERCHEMOTHERAP REP, V50, P163

Ogundimu EO, 2016, J CLIN EPIDEMIOL, V76, P175, DOI 10.1016/j.jclinepi.2016.02.031

Park H, 2006, PROC 12 ACM SIGKDD I, P126

Peng H., 2016, P 25 INT JOINT C ART, P1918

Peng HY, 2017, AAAI CONF ARTIF INTE, P2471

Peng HY, 2017, INFORM SCIENCES, V418, P652, DOI 10.1016/j.ins.2017.08.036

Scrivener M, 2016, TRANSL CANCER RES, V5, P398, DOI 10.21037/tcr.2016.06.18

Vaidya M, 2012, RADIOTHER ONCOL, V102, P239, DOI 10.1016/j.radonc.2011.10.014

Vallieres M, 2015, PHYS MED BIOL, V60, P5471, DOI 10.1088/0031-9155/60/14/5471

Vallieres M, 2018, J NUCL MED, V59, P189, DOI 10.2967/jnumed.117.200501

van Griethuysen JJM, 2017, CANCER RES, V77, pE104, DOI 10.1158/0008-5472.CAN-17-0339

Wu J, 2017, CLIN CANCER RES, V23, P3334, DOI 10.1158/1078-0432.CCR-16-2415

Wu J, 2016, RADIOLOGY, V281, P270, DOI 10.1148/radiol.2016151829

Yu W, 2017, INT J RAD ONCOL BIOL

Zwanenburg A, 2016, RADOLOGY, DOI DOI 10.1148/RADIOL.2020191145

NR 47

TC 43

Z9 48

U1 0

U2 10

PU ELSEVIER IRELAND LTD

PI CLARE

PA ELSEVIER HOUSE, BROOKVALE PLAZA, EAST PARK SHANNON, CO, CLARE, 00000,

IRELAND

SN 0167-8140

EI 1879-0887

J9 RADIOTHER ONCOL

JI Radiother. Oncol.

PD NOV

PY 2018

VL 129

IS 2

SI SI

BP 218

EP 226

DI 10.1016/j.radonc.2018.06.025

PG 9

WC Oncology; Radiology, Nuclear Medicine & Medical Imaging

WE Science Citation Index Expanded (SCI-EXPANDED)

SC Oncology; Radiology, Nuclear Medicine & Medical Imaging

GA HD1LK

UT WOS:000452271400004

PM 30473058

OA Green Accepted

DA 2022-08-24

ER

PT J

AU Hao, HX

Zhou, ZG

Li, SL

Maquilan, G

Folkert, MR

Iyengar, P

Westover, KD

Albuquerque, K

Liu, F

Choy, H

Timmerman, R

Yang, L

Wang, J

AF Hao, Hongxia

Zhou, Zhiguo

Li, Shulong

Maquilan, Genevieve

Folkert, Michael R.

Iyengar, Puneeth

Westover, Kenneth D.

Albuquerque, Kevin

Liu, Fang

Choy, Hak

Timmerman, Robert

Yang, Lin

Wang, Jing

TI Shell feature: a new radiomics descriptor for predicting distant failure

after radiotherapy in non-small cell lung cancer and cervix cancer

SO PHYSICS IN MEDICINE AND BIOLOGY

LA English

DT Article

DE NSCLC; cervix cancer; radiomics; distant failure; shell

ID GYNECOLOGIC-ONCOLOGY-GROUP; RADIATION-THERAPY; METASTASIS; CARCINOMA;

CLASSIFICATION; INVASION; OUTCOMES; CHEMOTHERAPY; HYDROXYUREA; CISPLATIN

AB Distant failure is the main cause of human cancer-related mortalities. To develop a model for predicting distant failure in non-small cell lung cancer (NSCLC) and cervix cancer (CC) patients, a shell feature, consisting of outer voxels around the tumor boundary, was constructed using pre-treatment positron emission tomography (PET) images from 48 NSCLC patients received stereotactic body radiation therapy and 52 CC patients underwent external beam radiation therapy and concurrent chemotherapy followed with high-dose-rate intracavitary brachytherapy. The hypothesis behind this feature is that non-invasive and invasive tumors may have different morphologic patterns in the tumor periphery, in turn reflecting the differences in radiological presentations in the PET images. The utility of the shell was evaluated by the support vector machine classifier in comparison with intensity, geometry, gray level co-occurrence matrix-based texture, neighborhood gray tone difference matrix-based texture, and a combination of these four features. The results were assessed in terms of accuracy, sensitivity, specificity, and AUC. Collectively, the shell feature showed better predictive performance than all the other features for distant failure prediction in both NSCLC and CC cohorts.

C1 [Hao, Hongxia; Liu, Fang] Xidian Univ, Sch Comp Sci & Technol, Xian 710071, Shaanxi, Peoples R China.

[Hao, Hongxia; Liu, Fang] Xidian Univ, Ministr Educ, Key Lab Intelligent Percept & Image Understandin, Xian 710071, Shaanxi, Peoples R China.

[Zhou, Zhiguo; Maquilan, Genevieve; Folkert, Michael R.; Iyengar, Puneeth; Westover, Kenneth D.; Albuquerque, Kevin; Choy, Hak; Timmerman, Robert; Wang, Jing] Univ Texas Southwestern Med Ctr Dallas, Dept Radiat Oncol, Dallas, TX 75235 USA.

[Li, Shulong] Southern Med Univ, Sch Biomed Engn, Guangzhou 510515, Guangdong, Peoples R China.

[Yang, Lin] Chinese Acad Med Sci, Canc Hosp, Natl Canc Ctr, Dept Pathol, Beijing 100021, Peoples R China.

RP Wang, J (通讯作者)，Univ Texas Southwestern Med Ctr Dallas, Dept Radiat Oncol, Dallas, TX 75235 USA.

EM Jing.Wang@utsouthwestern.edu

RI Yang, Lin/J-6807-2019; Li, Shulong/AAK-9054-2020; Wang,

Jing/N-7332-2019; Westover, Ken/AAZ-1795-2020; Hao,

Hongxia/AAO-7462-2020

OI Yang, Lin/0000-0002-7594-3770; Westover, Ken/0000-0003-3653-5923; Wang,

Jing/0000-0002-8491-4146

FU American Cancer Society [ACS-IRG-02-196]; US National Institutes of

Health [5P30CA142543]; NATIONAL CANCER INSTITUTE [P30CA142543] Funding

Source: NIH RePORTER; NATIONAL INSTITUTE OF BIOMEDICAL IMAGING AND

BIOENGINEERING [R01EB020366] Funding Source: NIH RePORTER

FX This work was supported in part by the American Cancer Society

(ACS-IRG-02-196) and US National Institutes of Health (5P30CA142543).

The authors would like to thank Dr Damiana Chiavolini for providing

helpful suggestions and editing the manuscript.

CR Almangush A, 2014, HEAD NECK-J SCI SPEC, V36, P811, DOI 10.1002/hed.23380

Braumann UD, 2005, IEEE T MED IMAGING, V24, P1286, DOI 10.1109/TMI.2005.855437

Chaffer CL, 2011, SCIENCE, V331, P1559, DOI 10.1126/science.1203543

Chang C.-C., 2011, ACM T INTEL SYST TEC, V2, DOI DOI 10.1145/1961189.1961199

Chawla NV, 2002, J ARTIF INTELL RES, V16, P321, DOI 10.1613/jair.953

Chetty IJ, 2013, RADIOTHER ONCOL, V109, P498, DOI 10.1016/j.radonc.2013.10.012

Cook GJR, 2014, CLIN TRANSL IMAGING, V2, P269, DOI 10.1007/s40336-014-0064-0

Dan GA, 2016, J CLIN MED, V5, P51

Gu S., 2014, ADV NEURAL INFORM PR, P793

Huang BX, 2016, PLOS ONE, V11, DOI 10.1371/journal.pone.0166311

Huang MY, 2017, SCI REP-UK, V7, DOI 10.1038/srep39880

Kadota K, 2014, J THORAC ONCOL, V9, P1126, DOI 10.1097/JTO.0000000000000253

Khamis H, 2017, MED IMAGE ANAL, V36, P15, DOI 10.1016/j.media.2016.10.007

Kidd EA, 2010, INT J RADIAT ONCOL, V77, P1085, DOI 10.1016/j.ijrobp.2009.06.041

KOELZER VH, 2014, FRONT ONCOL, V4

Koelzer VH, 2016, HUM PATHOL, V47, P4, DOI 10.1016/j.humpath.2015.08.007

Lennon FE, 2015, NAT REV CLIN ONCOL, V12, P664, DOI 10.1038/nrclinonc.2015.108

Lin C.-J., 2003, TECH REP

Lu SH, 2017, J THORAC ONCOL, V12, P223, DOI 10.1016/j.jtho.2016.09.129

Lugli A, 2017, MODERN PATHOL, V30, P1299, DOI 10.1038/modpathol.2017.46

Mehlen P, 2006, NAT REV CANCER, V6, P449, DOI 10.1038/nrc1886

Meijering EHW, 2001, MED IMAGE ANAL, V5, P111, DOI 10.1016/S1361-8415(00)00040-2

Mezheyeuski A, 2016, SCI REP-UK, V6, DOI 10.1038/srep36149

Nogami Y, 2014, ANTICANCER RES, V34, P585

Onozato ML, 2013, AM J SURG PATHOL, V37, P287, DOI 10.1097/PAS.0b013e31826885fb

Plaks V, 2013, SCIENCE, V341, P1186, DOI 10.1126/science.1235226

Quail DF, 2013, NAT MED, V19, P1423, DOI 10.1038/nm.3394

Robertson-Tessi M, 2015, CANCER RES, V75, P1567, DOI 10.1158/0008-5472.CAN-14-1428

Rose PG, 2007, J CLIN ONCOL, V25, P2804, DOI 10.1200/JCO.2006.09.4532

Saha PK, 2015, IEEE T MED IMAGING, V34, P1940, DOI 10.1109/TMI.2015.2417112

Schmid MP, 2014, GYNECOL ONCOL, V133, P256, DOI 10.1016/j.ygyno.2014.02.004

Suykens JAK, 1999, NEURAL PROCESS LETT, V9, P293, DOI 10.1023/A:1018628609742

Taira T, 2012, LUNG CANCER, V76, P423, DOI 10.1016/j.lungcan.2011.11.010

Timmerman R, 2010, JAMA-J AM MED ASSOC, V303, P1070, DOI 10.1001/jama.2010.261

Travis WD, 2015, J THORAC ONCOL, V10, P1243, DOI 10.1097/JTO.0000000000000630

Valastyan S, 2011, CELL, V147, P275, DOI 10.1016/j.cell.2011.09.024

van Baardwijk A, 2008, RADIOTHER ONCOL, V87, P55, DOI 10.1016/j.radonc.2008.02.002

Whitney CW, 1999, J CLIN ONCOL, V17, P1339, DOI 10.1200/JCO.1999.17.5.1339

Wood SL, 2014, CANCER TREAT REV, V40, P558, DOI 10.1016/j.ctrv.2013.10.001

Wu J, 2016, RADIOLOGY, V281, P270, DOI 10.1148/radiol.2016151829

Yamaguchi Y, 2010, J THORAC ONCOL, V5, P1361, DOI 10.1097/JTO.0b013e3181eaf2f3

Zhou ZG, 2013, COMPUT BIOL MED, V43, P1462, DOI 10.1016/j.compbiomed.2013.07.023

Zhou ZG, 2017, PHYS MED BIOL, V62, P4460, DOI 10.1088/1361-6560/aa6ae5

Zuluaga MA, 2015, MED IMAGE ANAL, V26, P185, DOI 10.1016/j.media.2015.09.001

NR 44

TC 36

Z9 38

U1 0

U2 20

PU IOP PUBLISHING LTD

PI BRISTOL

PA TEMPLE CIRCUS, TEMPLE WAY, BRISTOL BS1 6BE, ENGLAND

SN 0031-9155

EI 1361-6560

J9 PHYS MED BIOL

JI Phys. Med. Biol.

PD MAY

PY 2018

VL 63

IS 9

AR 095007

DI 10.1088/1361-6560/aabb5e

PG 17

WC Engineering, Biomedical; Radiology, Nuclear Medicine & Medical Imaging

WE Science Citation Index Expanded (SCI-EXPANDED)

SC Engineering; Radiology, Nuclear Medicine & Medical Imaging

GA GE7PL

UT WOS:000431425200002

PM 29616661

OA Green Submitted, Green Accepted

DA 2022-08-24

ER

PT J

AU Leijenaar, RTH

Carvalho, S

Velazquez, ER

Van Elmpt, WJC

Parmar, C

Hoekstra, OS

Hoekstra, CJ

Boellaard, R

Dekker, ALAJ

Gillies, RJ

Aerts, HJWL

Lambin, P

AF Leijenaar, Ralph T. H.

Carvalho, Sara

Velazquez, Emmanuel Rios

Van Elmpt, Wouter J. C.

Parmar, Chintan

Hoekstra, Otto S.

Hoekstra, Corneline J.

Boellaard, Ronald

Dekker, Andre L. A. J.

Gillies, Robert J.

Aerts, Hugo J. W. L.

Lambin, Philippe

TI Stability of FDG-PET Radiomics features: An integrated analysis of

test-retest and inter-observer variability

SO ACTA ONCOLOGICA

LA English

DT Article

ID CELL LUNG-CANCER; STANDARDIZED UPTAKE VALUE; RESPONSE ASSESSMENT;

TEXTURAL FEATURES; F-18-FDG PET; RADIOTHERAPY; IMAGES; TUMOR;

REPEATABILITY; CT

AB Purpose. Besides basic measurements as maximum standardized uptake value (SUV)(max) or SUVmean derived from 18F-FDG positron emission tomography (PET) scans, more advanced quantitative imaging features (i.e. "Radiomics" features) are increasingly investigated for treatment monitoring, outcome prediction, or as potential biomarkers. With these prospected applications of Radiomics features, it is a requisite that they provide robust and reliable measurements. The aim of our study was therefore to perform an integrated stability analysis of a large number of PET-derived features in non-small cell lung carcinoma (NSCLC), based on both a test-retest and an inter-observer setup. Methods. Eleven NSCLC patients were included in the test-retest cohort. Patients underwent repeated PET imaging within a one day interval, before any treatment was delivered. Lesions were delineated by applying a threshold of 50% of the maximum uptake value within the tumor. Twenty-three NSCLC patients were included in the inter-observer cohort. Patients underwent a diagnostic whole body PET-computed tomography (CT). Lesions were manually delineated based on fused PET-CT, using a standardized clinical delineation protocol. Delineation was performed independently by five observers, blinded to each other. Fifteen first order statistics, 39 descriptors of intensity volume histograms, eight geometric features and 44 textural features were extracted. For every feature, test-retest and inter-observer stability was assessed with the intra-class correlation coefficient (ICC) and the coefficient of variability, normalized to mean and range. Similarity between test-retest and inter-observer stability rankings of features was assessed with Spearman's rank correlation coefficient. Results. Results showed that the majority of assessed features had both a high test-retest (71%) and inter-observer (91%) stability in terms of their ICC. Overall, features more stable in repeated PET imaging were also found to be more robust against inter-observer variability. Conclusion. Results suggest that further research of quantitative imaging features is warranted with respect to more advanced applications of PET imaging as being used for treatment monitoring, outcome prediction or imaging biomarkers.

C1 [Leijenaar, Ralph T. H.; Carvalho, Sara; Velazquez, Emmanuel Rios; Van Elmpt, Wouter J. C.; Parmar, Chintan; Dekker, Andre L. A. J.; Aerts, Hugo J. W. L.; Lambin, Philippe] MUMC, Dept Radiat Oncol MAASTRO, GROW Sch Oncol & Dev Biol, Maastricht, Netherlands.

[Hoekstra, Otto S.; Boellaard, Ronald] Vrije Univ Amsterdam Med Ctr, Dept Radiol & Nucl Med, Amsterdam, Netherlands.

[Hoekstra, Corneline J.] Jeroen Bosch Med Ctr, Dept Nucl Med, Shertogenbosch, Netherlands.

[Gillies, Robert J.] Univ S Florida, Coll Med, H Lee Moffitt Canc Ctr & Res Inst, Dept Canc Imaging & Metab, Tampa, FL 33612 USA.

[Aerts, Hugo J. W. L.] Harvard Univ, Brigham & Womens Hosp, Dana Farber Canc Inst, Dept Radiat Oncol,Med Sch, Boston, MA 02115 USA.

[Aerts, Hugo J. W. L.] Harvard Univ, Brigham & Womens Hosp, Dana Farber Canc Inst, Dept Radiol,Med Sch, Boston, MA 02115 USA.

RP Leijenaar, RTH (通讯作者)，MAASTRO Clin, Dr Tanslaan 12, NL-6229 ET Maastricht, Netherlands.

EM ralph.leijenaar@maastro.nl

RI Dekker, Andre/AAE-4830-2019; parmar, chintan/J-2977-2019; Aerts,

Hugo/ABF-2821-2020; Aerts, Hugo/P-6350-2015

OI Dekker, Andre/0000-0002-0422-7996; parmar, chintan/0000-0002-2140-814X;

Aerts, Hugo/0000-0002-2122-2003; Aerts, Hugo/0000-0002-2122-2003;

Boellaard, Ronald/0000-0002-0313-5686; Gillies,

Robert/0000-0002-8888-7747; Lambin, Philippe/0000-0001-7961-0191

FU QuIC-ConCePT project; EFPI A companies; Innovative Medicine Initiative

Joint Undertaking (IMI JU) [115151]; National Institute of Health

[NIH-USA U01 CA 143062-01]; CTMM framework (AIRFORCE project) [030-103];

EU; euroCAT; Kankeronderzoekfonds Limburg from the Health Foundation

Limburg; Dutch Cancer Society [KWF UM 2011-5020, KWF UM 2009-4454];

NATIONAL CANCER INSTITUTE [U01CA143062] Funding Source: NIH RePORTER

FX Authors acknowledge financial support from the QuIC-ConCePT project,

which is partly funded by EFPI A companies and the Innovative Medicine

Initiative Joint Undertaking (IMI JU) under Grant Agreement No. 115151.

Authors also acknowledge financial support from the National Institute

of Health (NIH-USA U01 CA 143062-01, Radiomics of NSCLC), the CTMM

framework (AIRFORCE project, grant 030-103), EU 6th and 7th framework

program (EUROXY, METOXIA, EURECA, ART-FORCE), euroCAT (IVA

Interreg-www.eurocat.info), Kankeronderzoekfonds Limburg from the Health

Foundation Limburg and the Dutch Cancer Society (KWF UM 2011-5020, KWF

UM 2009-4454).

CR Bland JM, 2007, J BIOPHARM STAT, V17, P571, DOI 10.1080/10543400701329422

Buckler AJ, 2011, RADIOLOGY, V259, P875, DOI 10.1148/radiol.10100800

Cheebsumon P, 2012, EJNMMI RES, V2, DOI 10.1186/2191-219X-2-56

de Langen AJ, 2012, J NUCL MED, V53, P701, DOI 10.2967/jnumed.111.095299

De Ruysscher D, 2012, LUNG CANCER, V75, P141, DOI 10.1016/j.lungcan.2011.07.018

Deasy JO, 2003, MED PHYS, V30, P979, DOI 10.1118/1.1568978

El Naqa I, 2009, PATTERN RECOGN, V42, P1162, DOI 10.1016/j.patcog.2008.08.011

Frings V, 2010, J NUCL MED, V51, P1870, DOI 10.2967/jnumed.110.077255

Galavis PE, 2010, ACTA ONCOL, V49, P1012, DOI 10.3109/0284186X.2010.498437

Galloway M., 1975, COMPUT VISION GRAPH, V4, P172, DOI [10.1016/S0146-664X(75)80008-6, DOI 10.1016/S0146-664X(75)80008-6]

HARALICK RM, 1973, IEEE T SYST MAN CYB, VSMC3, P610, DOI 10.1109/TSMC.1973.4309314

Kumar V, 2012, MAGN RESON IMAGING, V30, P1234, DOI 10.1016/j.mri.2012.06.010

Lambin P, 2013, NAT REV CLIN ONCOL, V10, P27, DOI 10.1038/nrclinonc.2012.196

Lambin P, 2012, EUR J CANCER, V48, P441, DOI 10.1016/j.ejca.2011.11.036

Lin P, 2011, RADIOTHER ONCOL, V101, P284, DOI 10.1016/j.radonc.2011.06.030

SHROUT PE, 1979, PSYCHOL BULL, V86, P420, DOI 10.1037/0033-2909.86.2.420

Takeda A, 2011, RADIOTHER ONCOL, V101, P291, DOI 10.1016/j.radonc.2011.08.008

Thie JA, 2004, J NUCL MED, V45, P1431

Tixier F, 2012, J NUCL MED, V53, P693, DOI 10.2967/jnumed.111.099127

Tixier F, 2011, J NUCL MED, V52, P369, DOI 10.2967/jnumed.110.082404

Vaidya M, 2012, RADIOTHER ONCOL, V102, P239, DOI 10.1016/j.radonc.2011.10.014

Van Baardwijk A, 2007, INT J RADIAT ONCOL, V68, P771, DOI 10.1016/j.ijrobp.2006.12.067

Van Elmpt W, 2011, Q J NUCL MED MOL IM, V55, P648

van Elmpt W, 2012, J NUCL MED, V53, P1514, DOI 10.2967/jnumed.111.102566

Velazquez ER, 2010, ACTA ONCOL, V49, P1033, DOI 10.3109/0284186X.2010.498441

NR 25

TC 287

Z9 297

U1 1

U2 71

PU INFORMA HEALTHCARE

PI LONDON

PA TELEPHONE HOUSE, 69-77 PAUL STREET, LONDON EC2A 4LQ, ENGLAND

SN 0284-186X

J9 ACTA ONCOL

JI Acta Oncol.

PD OCT

PY 2013

VL 52

IS 7

BP 1391

EP 1397

DI 10.3109/0284186X.2013.812798

PG 7

WC Oncology

WE Science Citation Index Expanded (SCI-EXPANDED)

SC Oncology

GA 223AU

UT WOS:000324776100020

PM 24047337

OA Green Accepted, Green Published, Bronze

DA 2022-08-24

ER

PT J

AU Grgic, A

Ballek, E

Fleckenstein, J

Moca, N

Kremp, S

Schaefer, A

Kuhnigk, JM

Rube, C

Kirsch, CM

Hellwig, D

AF Grgic, Aleksandar

Ballek, Elena

Fleckenstein, Jochen

Moca, Norbert

Kremp, Stephanie

Schaefer, Andrea

Kuhnigk, Jan-Martin

Ruebe, Christian

Kirsch, Carl-Martin

Hellwig, Dirk

TI Impact of rigid and nonrigid registration on the determination of

F-18-FDG PET-based tumour volume and standardized uptake value in

patients with lung cancer

SO EUROPEAN JOURNAL OF NUCLEAR MEDICINE AND MOLECULAR IMAGING

LA English

DT Article

DE Non-small cell lung carcinoma (NSCLC); Spiral computed tomography;

Positron emission tomography; Image registration; Image analysis;

Computer-assisted

ID RESPIRATORY MOTION; IMAGE REGISTRATION; CO-REGISTRATION; FDG-PET; CT;

RADIOTHERAPY; THORAX; DELINEATION; TOMOGRAPHY

AB Assessment of the metabolically active tumour tissue by FDG PET is evolving for use in the diagnosis of non-small-cell lung cancer (NSCLC), in the planning of radiotherapy, and in follow-up and response evaluation. For exact evaluation accurate registration of PET and CT data is required. The registration process is usually based on rigid algorithms; however, nonrigid algorithms are increasingly being used. The influence of the registration method on FDG PET-based standardized uptake value (SUVmax) and metabolic tumour volume (MTV) definition has not yet been evaluated. We compared intra- and interindividual differences in SUV and MTV between rigid- and nonrigid-registered PET and CT acquired during different breathing manoeuvres.

The study group comprised 28 radiotherapy candidates with histologically proven NSCLC who underwent FDG PET acquisition and three CT acquisitions (expiration - EXP, inspiration - INS, mid-breath-hold - MID). All scans were registered with both a rigid (R) and a nonrigid (NR) procedure resulting in six fused datasets: R-INS, R-EXP, R-MID, NR-INS, NR-EXP and NR-MID. For the delineation of MTVs a contrast-oriented contouring algorithm developed in-house was used. To accelerate the delineation a semiautomatic software prototype was utilized.

Tumour mean SUVmax did not differ for R and NR registration (R 17.5 +/- 7, NR 17.4 +/- 7; p=0.2). The mean MTV was higher by 3 +/- 12 ml (p=0.02) in the NR group than in the R group, as was the mean tumour diameter (by 0.1 +/- 0.2 cm; p < 0.01). With respect to the three different breathing manoeuvres, there were no differences in MTV in the R group (p > 0.7). In intraindividual comparison there were no significant differences in MTVs concerning the registration pairs R-EXP (68 +/- 88 ml) vs. NR-EXP (69 +/- 85 ml) und R-MID (68 +/- 86 ml) vs. NR-MID (69 +/- 83 ml) (both p > 0.4). However, the MTVs were larger after NR registration during inspiration (R-INS 68 +/- 82 vs. NR-INS 78 +/- 93 ml; p=0.02).

The use of nonrigid algorithms may lead to a change in MTV, whose extent is influenced by the breathing manoeuvre on CT. Nonrigid registration methods cannot be recommended for the definition of MTV if the CT scan is performed during inspiration. The choice of registration algorithm has no significant impact on SUVmax.

C1 [Grgic, Aleksandar; Ballek, Elena; Moca, Norbert; Schaefer, Andrea; Kirsch, Carl-Martin; Hellwig, Dirk] Univ Saarland, Med Ctr, Dept Nucl Med, D-66421 Homburg, Germany.

[Fleckenstein, Jochen; Kremp, Stephanie; Ruebe, Christian] Univ Saarland, Med Ctr, Dept Radiooncol, D-66421 Homburg, Germany.

[Kuhnigk, Jan-Martin] MEVIS, Bremen, Germany.

RP Grgic, A (通讯作者)，Univ Saarland, Med Ctr, Dept Nucl Med, Geb 50, D-66421 Homburg, Germany.

EM aleksandar.grgic@uks.eu

RI Hellwig, Dirk/O-8617-2019; Hellwig, Dirk/A-4128-2008

OI Hellwig, Dirk/0000-0002-3056-0143; Kuhnigk,

Jan-Martin/0000-0001-9255-2993

CR Boellaard R, 2009, J NUCL MED, V50, p11S, DOI 10.2967/jnumed.108.057182

Czernin J, 2007, J NUCL MED, V48, p2S

Daou D, 2008, EUR J NUCL MED MOL I, V35, P1961, DOI 10.1007/s00259-008-0931-x

DICE LR, 1945, ECOLOGY, V26, P297, DOI 10.2307/1932409

Erdi YE, 2004, J NUCL MED, V45, P1287

Facey K, 2007, HEALTH TECHNOL ASSES, V11, P1

Fitton I, 2008, INT J RADIAT ONCOL, V70, P1403, DOI 10.1016/j.ijrobp.2007.08.063

Gietema HA, 2007, RADIOLOGY, V245, P888, DOI 10.1148/radiol.2452061054

Gilman MD, 2007, J COMPUT ASSIST TOMO, V31, P395, DOI 10.1097/01.rct.0000237817.18678.9c

Gilman MD, 2006, AM J ROENTGENOL, V187, P1357, DOI 10.2214/AJR.05.1427

Goerres GW, 2002, EUR J NUCL MED MOL I, V29, P351, DOI 10.1007/s00259-001-0710-4

Grgic A, 2009, INT J RADIAT ONCOL, V73, P103, DOI 10.1016/j.ijrobp.2008.03.063

Grgic A, 2010, EUR J NUCL MED MOL I, V37, P1087, DOI 10.1007/s00259-010-1387-3

Grgic A, 2009, J NUCL MED, V50, P1921, DOI 10.2967/jnumed.109.065649

Hellwig D, 2009, NUKLEARMED-NUCL MED, V48, P59, DOI 10.3413/nukmed-0217

Hellwig D, 2009, NUKLEARMEDIZIN, V48

Hicks RJ, 2009, J NUCL MED, V50, p31S, DOI 10.2967/jnumed.108.057216

Ireland RH, 2007, INT J RADIAT ONCOL, V68, P952, DOI 10.1016/j.ijrobp.2007.02.017

Krishnasetty V, 2005, RADIOLOGY, V237, P635, DOI 10.1148/radiol.2372041719

Kuhnigk JM, 2005, RADIOGRAPHICS, V25, P525, DOI 10.1148/rg.252045070

Liu C, 2009, PHYS MED BIOL, V54, P7345, DOI 10.1088/0031-9155/54/24/007

Liu HH, 2007, INT J RADIAT ONCOL, V68, P531, DOI 10.1016/j.ijrobp.2006.12.066

Lucignani G, 2009, EUR J NUCL MED MOL I, V36, P1520, DOI 10.1007/s00259-009-1214-x

Moreno A, 2008, COMPUT AIDED SURG, V13, P281, DOI 10.3109/10929080802431980

Nehmeh SA, 2004, MED PHYS, V31, P3179, DOI 10.1118/1.1809778

Nehmeh SA, 2008, SEMIN NUCL MED, V38, P167, DOI 10.1053/j.semnuclmed.2008.01.002

Nestle U, 2007, EUR J NUCL MED MOL I, V34, P453, DOI 10.1007/s00259-006-0252-x

Pietrzyk U, 2005, NUKLEARMED-NUCL MED, V44, pS13

Schaefer A, 2008, EUR J NUCL MED MOL I, V35, P1989, DOI 10.1007/s00259-008-0875-1

Shankar LK, 2006, J NUCL MED, V47, P1059

Slomka PJ, 2009, EUR J NUCL MED MOL I, V36, P44, DOI 10.1007/s00259-008-0941-8

Slomka PJ, 2003, J NUCL MED, V44, P1156

Tylski P, 2010, J NUCL MED, V51, P268, DOI 10.2967/jnumed.109.066241

Wahl RL, 2009, J NUCL MED, V50, p122S, DOI 10.2967/jnumed.108.057307

Weber WA, 2009, J NUCL MED, V50, p1S, DOI 10.2967/jnumed.108.057174

NR 35

TC 5

Z9 5

U1 0

U2 2

PU SPRINGER

PI NEW YORK

PA 233 SPRING ST, NEW YORK, NY 10013 USA

SN 1619-7070

EI 1619-7089

J9 EUR J NUCL MED MOL I

JI Eur. J. Nucl. Med. Mol. Imaging

PD MAY

PY 2011

VL 38

IS 5

BP 856

EP 864

DI 10.1007/s00259-010-1719-3

PG 9

WC Radiology, Nuclear Medicine & Medical Imaging

WE Science Citation Index Expanded (SCI-EXPANDED)

SC Radiology, Nuclear Medicine & Medical Imaging

GA 745DH

UT WOS:000289144800008

PM 21258929

DA 2022-08-24

ER

PT J

AU Zhang, J

Jin, JB

Ai, Y

Zhu, KC

Xiao, CJ

Xie, CY

Jin, XC

AF Zhang, Ji

Jin, Juebin

Ai, Yao

Zhu, Kecheng

Xiao, Chengjian

Xie, Congying

Jin, Xiance

TI Computer Tomography Radiomics-Based Nomogram in the Survival Prediction

for Brain Metastases From Non-Small Cell Lung Cancer Underwent Whole

Brain Radiotherapy

SO FRONTIERS IN ONCOLOGY

LA English

DT Article

DE brain metastasis; non-small cell lung cancer; whole brain radiotherapy;

overall survival; radiomics; nomogram

ID BIOMARKERS

AB Prognostic parameters and models were believed to be helpful in improving the treatment outcome for patients with brain metastasis (BM). The purpose of this study was to investigate the feasibility of computer tomography (CT) radiomics based nomogram to predict the survival of patients with BM from non-small cell lung cancer (NSCLC) treated with whole brain radiotherapy (WBRT). A total of 195 patients with BM from NSCLC who underwent WBRT from January 2012 to December 2016 were retrospectively reviewed. Radiomics features were extracted and selected from pretherapeutic CT images with least absolute shrinkage and selection operator (LASSO) regression. A nomogram was developed and evaluated by integrating radiomics features and clinical factors to predict the survival of individual patient. Five radiomics features were screened out from 105 radiomics features according to the LASSO Cox regression. According to the optimal cutoff value of radiomics score (Rad-score), patients were stratified into low-risk (Rad-score <= -0.14) and high-risk (Rad-score > -0.14) groups. Multivariable analysis indicated that sex, karnofsky performance score (KPS) and Rad-score were independent predictors for overall survival (OS). The concordance index (C-index) of the nomogram in the training cohort and validation cohort was 0.726 and 0.660, respectively. An area under curve (AUC) of 0.786 and 0.788 was achieved for the short-term and long-term survival prediction, respectively. In conclusion, the nomogram based on radiomics features from CT images and clinical factors was feasible to predict the OS of BM patients from NSCLC who underwent WBRT.

C1 [Zhang, Ji; Jin, Juebin; Ai, Yao; Zhu, Kecheng; Xiao, Chengjian; Xie, Congying; Jin, Xiance] Wenzhou Med Univ, Affiliated Hosp 1, Dept Radiotherapy Ctr, Wenzhou, Peoples R China.

[Xie, Congying] Wenzhou Med Univ, Affiliated Hosp 2, Dept Radiat & Med Oncol, Wenzhou, Peoples R China.

RP Xie, CY; Jin, XC (通讯作者)，Wenzhou Med Univ, Affiliated Hosp 1, Dept Radiotherapy Ctr, Wenzhou, Peoples R China.; Xie, CY (通讯作者)，Wenzhou Med Univ, Affiliated Hosp 2, Dept Radiat & Med Oncol, Wenzhou, Peoples R China.

EM wzxiecongying@163.com; jinxc1979@hotmail.com

FU Wenzhou Municipal Science and Technology Bureau [2018ZY016, H20180003];

National Natural Science Foundation of China [11675122]

FX This work was partially funded by the Wenzhou Municipal Science and

Technology Bureau (Nos. 2018ZY016 and H20180003) and National Natural

Science Foundation of China under Grant No. 11675122.

CR Barnholtz-Sloan JS, 2012, NEURO-ONCOLOGY, V14, P910, DOI 10.1093/neuonc/nos087

Brown PD, 2020, J CLIN ONCOL, V38, P1019, DOI 10.1200/JCO.19.02767

Cyll K, 2017, BRIT J CANCER, V117, P367, DOI 10.1038/bjc.2017.171

Della Seta M, 2019, ACTA RADIOL, V60, P1496, DOI 10.1177/0284185119831692

Diamandis EP, 2012, BMC MED, V10, DOI 10.1186/1741-7015-10-87

Gui CC, 2019, J NEURO-ONCOL, V144, P351, DOI 10.1007/s11060-019-03235-7

Huang CY, 2020, J NEURO-ONCOL, V146, P439, DOI 10.1007/s11060-019-03343-4

Huang YQ, 2016, RADIOLOGY, V281, P947, DOI 10.1148/radiol.2016152234

Jamal-Hanjani M, 2015, CLIN CANCER RES, V21, P1258, DOI 10.1158/1078-0432.CCR-14-1429

Jenkinson MD, 2011, EUR J CANCER, V47, P649, DOI 10.1016/j.ejca.2010.11.033

Joshi R, 2016, CLIN NEUROL NEUROSUR, V147, P30, DOI 10.1016/j.clineuro.2016.05.001

Karami E, 2019, SCI REP-UK, V9, DOI 10.1038/s41598-019-56185-5

Kniep HC, 2019, RADIOLOGY, V290, P479, DOI 10.1148/radiol.2018180946

Lagerwaard FJ, 1999, INT J RADIAT ONCOL, V43, P795, DOI 10.1016/S0360-3016(98)00442-8

Lee JM, 2010, ANN ONCOL, V21, P205, DOI 10.1093/annonc/mdq375

Liubota R., 2017, Experimental Oncology, V39, P75

Mulvenna P, 2016, LANCET, V388, P2004, DOI 10.1016/S0140-6736(16)30825-X

Nieder C, 2009, RADIAT ONCOL, V4, DOI 10.1186/1748-717X-4-10

Oh Y, 2009, CANCER, V115, P2930, DOI 10.1002/cncr.24333

Ostrom QT, 2015, NEURO-ONCOLOGY, V17, P1, DOI [10.1093/neuonc/nov189, 10.1093/neuonc/noaa200]

Park Y, 2015, J NEURO-ONCOL, V125, P377, DOI 10.1007/s11060-015-1926-7

Pietrantonio F, 2015, RADIOTHER ONCOL, V117, P315, DOI 10.1016/j.radonc.2015.08.023

Rades D, 2013, STRAHLENTHER ONKOL, V189, P996, DOI 10.1007/s00066-013-0442-y

Rades D, 2017, INT J GYNECOL CANCER, V27, P597, DOI 10.1097/IGC.0000000000000899

Schuette W, 2004, LUNG CANCER, V45, pS253, DOI 10.1016/j.lungcan.2004.07.967

Sehmisch L, 2017, ANTICANCER RES, V37, P249, DOI 10.21873/anticanres.11314

Silvestri GA, 2007, CHEST, V132, p178S, DOI 10.1378/chest.07-1360

SORENSEN JB, 1988, J CLIN ONCOL, V6, P1474, DOI 10.1200/JCO.1988.6.9.1474

Sperduto PW, 2008, INT J RADIAT ONCOL, V70, P510, DOI 10.1016/j.ijrobp.2007.06.074

Sperduto PW, 2010, INT J RADIAT ONCOL, V77, P655, DOI 10.1016/j.ijrobp.2009.08.025

Szopa W, 2017, BIOMED RES INT, V2017, DOI 10.1155/2017/8013575

Taimur Sadaf, 2003, Curr Oncol Rep, V5, P342, DOI 10.1007/s11912-003-0077-8

Tsao M, 2012, CANCER-AM CANCER SOC, V118, P2486, DOI 10.1002/cncr.26515

Yokoi K, 1999, CHEST, V115, P714, DOI 10.1378/chest.115.3.714

Zindler JD, 2017, RADIOTHER ONCOL, V123, P189, DOI 10.1016/j.radonc.2017.02.006

NR 35

TC 2

Z9 2

U1 1

U2 4

PU FRONTIERS MEDIA SA

PI LAUSANNE

PA AVENUE DU TRIBUNAL FEDERAL 34, LAUSANNE, CH-1015, SWITZERLAND

SN 2234-943X

J9 FRONT ONCOL

JI Front. Oncol.

PD FEB 11

PY 2021

VL 10

AR 610691

DI 10.3389/fonc.2020.610691

PG 9

WC Oncology

WE Science Citation Index Expanded (SCI-EXPANDED)

SC Oncology

GA QL8UI

UT WOS:000621355600001

PM 33643912

OA Green Published, gold

DA 2022-08-24

ER

PT J

AU Fave, X

Zhang, LF

Yang, JZ

Mackin, D

Balter, P

Gomez, D

Followill, D

Jones, AK

Stingo, F

Liao, ZX

Mohan, R

Court, L

AF Fave, Xenia

Zhang, Lifei

Yang, Jinzhong

Mackin, Dennis

Balter, Peter

Gomez, Daniel

Followill, David

Jones, Aaron Kyle

Stingo, Francesco

Liao, Zhongxing

Mohan, Radhe

Court, Laurence

TI Delta-radiomics features for the prediction of patient outcomes in

non-small cell lung cancer

SO SCIENTIFIC REPORTS

LA English

DT Article

ID CT TEXTURE ANALYSIS; RESPONSE EVALUATION CRITERIA; COMPUTED-TOMOGRAPHY;

TUMOR HETEROGENEITY; SURVIVAL; RECIST; REPRODUCIBILITY; VALIDATION;

THERAPY; IMPACT

AB Radiomics is the use of quantitative imaging features extracted from medical images to characterize tumor pathology or heterogeneity. Features measured at pretreatment have successfully predicted patient outcomes in numerous cancer sites. This project was designed to determine whether radiomics features measured from non-small cell lung cancer (NSCLC) change during therapy and whether those features (delta-radiomics features) can improve prognostic models. Features were calculated from pretreatment and weekly intra-treatment computed tomography images for 107 patients with stage III NSCLC. Pretreatment images were used to determine feature-specific image preprocessing. Linear mixed-effects models were used to identify features that changed significantly with dose-fraction. Multivariate models were built for overall survival, distant metastases, and local recurrence using only clinical factors, clinical factors and pretreatment radiomics features, and clinical factors, pretreatment radiomics features, and delta-radiomics features. All of the radiomics features changed significantly during radiation therapy. For overall survival and distant metastases, pretreatment compactness improved the c-index. For local recurrence, pretreatment imaging features were not prognostic, while texture-strength measured at the end of treatment significantly stratified high-and low-risk patients. These results suggest radiomics features change due to radiation therapy and their values at the end of treatment may be indicators of tumor response.

C1 [Fave, Xenia; Zhang, Lifei; Yang, Jinzhong; Mackin, Dennis; Balter, Peter; Followill, David; Mohan, Radhe; Court, Laurence] Univ Texas MD Anderson Canc Ctr, Dept Radiat Phys, 1515 Holcombe Blvd, Houston, TX 77030 USA.

[Fave, Xenia; Court, Laurence] Univ Texas Houston, Grad Sch Biomed Sci Houston, 6767 Bertner Ave, Houston, TX 77030 USA.

[Gomez, Daniel; Liao, Zhongxing] Univ Texas MD Anderson Canc Ctr, Dept Radiat Oncol, 1515 Holcombe Blvd, Houston, TX 77030 USA.

[Jones, Aaron Kyle] Univ Texas MD Anderson Canc Ctr, Dept Imaging Phys, 1515 Holcombe Blvd, Houston, TX 77030 USA.

[Stingo, Francesco] Univ Florence, Dipartimento Stat Informat Applicaz G Parenti, Viale Morgagni 59, I-50134 Florence, Italy.

RP Fave, X (通讯作者)，Univ Texas MD Anderson Canc Ctr, Dept Radiat Phys, 1515 Holcombe Blvd, Houston, TX 77030 USA.; Fave, X (通讯作者)，Univ Texas Houston, Grad Sch Biomed Sci Houston, 6767 Bertner Ave, Houston, TX 77030 USA.

EM xjfave@mdanderson.org

RI Mackin, Dennis/Y-1503-2019; Stingo, Francesco/N-6514-2019

OI Stingo, Francesco/0000-0001-9150-8552; Court,

Laurence/0000-0002-3241-6145; Ray, Xenia/0000-0003-0150-0843; Yang,

Jinzhong/0000-0002-9254-4501

FU U.S.National Institutes of Health [5U19CA021239]; Cancer Prevention and

Research Institute of Texas [RP110562-P2]; NATIONAL CANCER INSTITUTE

[U19CA021239] Funding Source: NIH RePORTER

FX This project was funded in part by grant 5U19CA021239 from the U.S.

National Institutes of Health and by grant RP110562-P2 from the Cancer

Prevention and Research Institute of Texas. The authors would also like

to acknowledge Kathryn Hale for help with manuscript preparation.

CR Aerts HJWL, 2014, NAT COMMUN, V5, DOI 10.1038/ncomms5006

AMADASUN M, 1989, IEEE T SYST MAN CYB, V19, P1264, DOI 10.1109/21.44046

ANDERSON JR, 1983, J CLIN ONCOL, V1, P710, DOI 10.1200/JCO.1983.1.11.710

Balagurunathan Y, 2014, TRANSL ONCOL, V7, P72, DOI 10.1593/tlo.13844

Basu S, 2011, IEEE SYS MAN CYBERN, P1306, DOI 10.1109/ICSMC.2011.6083840

Bates D, 2015, J STAT SOFTW, V67, P1, DOI 10.18637/jss.v067.i01

BENJAMINI Y, 1995, J R STAT SOC B, V57, P289, DOI 10.1111/j.2517-6161.1995.tb02031.x

Carvalho S, 2016, RADIOTHER ONCOL, V118, pS20, DOI DOI 10.1016/S0167-8140(16)30042-1

Chalkidou A, 2015, PLOS ONE, V10, DOI 10.1371/journal.pone.0124165

Chao KSC, 2007, INT J RADIAT ONCOL, V68, P1512, DOI 10.1016/j.ijrobp.2007.04.037

Coroller TP, 2016, RADIOTHER ONCOL, V119, P480, DOI 10.1016/j.radonc.2016.04.004

Coroller TP, 2015, RADIOTHER ONCOL, V114, P345, DOI 10.1016/j.radonc.2015.02.015

Cunliffe A, 2015, INT J RADIAT ONCOL, V91, P1048, DOI 10.1016/j.ijrobp.2014.11.030

Dafni U, 2011, CIRC-CARDIOVASC QUAL, V4, P363, DOI 10.1161/CIRCOUTCOMES.110.957951

Eisenhauer EA, 2009, EUR J CANCER, V45, P228, DOI 10.1016/j.ejca.2008.10.026

Fave X, 2016, TRANSL CANCER RES, V5, P349, DOI 10.21037/tcr.2016.07.11

Fave X, 2015, COMPUT MED IMAG GRAP, V44, P54, DOI 10.1016/j.compmedimag.2015.04.006

Fried DV, 2014, INT J RADIAT ONCOL, V90, P834, DOI 10.1016/j.ijrobp.2014.07.020

Fushiki T, 2011, STAT COMPUT, V21, P137, DOI 10.1007/s11222-009-9153-8

Galloway M., 1975, COMPUT VISION GRAPH, V4, P172, DOI [10.1016/S0146-664X(75)80008-6, DOI 10.1016/S0146-664X(75)80008-6]

Ganeshan B, 2012, EUR RADIOL, V22, P796, DOI 10.1007/s00330-011-2319-8

Ganeshan B, 2010, CANCER IMAGING, V10, P137, DOI 10.1102/1470-7330.2010.0021

Gevaert O, 2012, RADIOLOGY, V264, P387, DOI 10.1148/radiol.12111607

Goh V, 2011, RADIOLOGY, V261, P165, DOI 10.1148/radiol.11110264

HARALICK RM, 1979, P IEEE, V67, P786, DOI 10.1109/PROC.1979.11328

HARALICK RM, 1973, IEEE T SYST MAN CYB, VSMC3, P610, DOI 10.1109/TSMC.1973.4309314

HARRELL FE, 1982, JAMA-J AM MED ASSOC, V247, P2543, DOI 10.1001/jama.247.18.2543

HILSENBECK SG, 1992, BREAST CANCER RES TR, V22, P197, DOI 10.1007/BF01840833

Hilsenbeck SG, 1996, STAT MED, V15, P103, DOI 10.1002/(SICI)1097-0258(19960115)15:1<103::AID-SIM156>3.0.CO;2-Y

Jaffe CC, 2006, J CLIN ONCOL, V24, P3245, DOI 10.1200/JCO.2006.06.5599

Kumar V, 2012, MAGN RESON IMAGING, V30, P1234, DOI 10.1016/j.mri.2012.06.010

Lambin P, 2012, EUR J CANCER, V48, P441, DOI 10.1016/j.ejca.2011.11.036

Liu HH, 2007, INT J RADIAT ONCOL, V68, P531, DOI 10.1016/j.ijrobp.2006.12.066

Mackin D, 2015, INVEST RADIOL, V50, P757, DOI 10.1097/RLI.0000000000000180

Miles KA, 2016, CANCER IMAGING, V16, DOI 10.1186/s40644-016-0065-5

Molina JR, 2008, MAYO CLIN PROC, V83, P584, DOI 10.4065/83.5.584

Nishino M, 2010, AM J ROENTGENOL, V195, pW221, DOI 10.2214/AJR.09.3928

Parmar C, 2015, SCI REP-UK, V5, DOI 10.1038/srep11044

R Core Team, 2019, R LANG ENV STAT COMP

Rao SX, 2016, UNITED EUR GASTROENT, V4, P257, DOI 10.1177/2050640615601603

Seppenwoolde Y, 2002, INT J RADIAT ONCOL, V53, P822, DOI 10.1016/S0360-3016(02)02803-1

Simon RM, 2011, BRIEF BIOINFORM, V12, P203, DOI 10.1093/bib/bbr001

The University of Texas MD Anderson Cancer Center, IM GUID AD CONF PHOT

Therneau T., 2015, SURVIVAL PACKAGE SUR

Tian F, 2015, ABDOM IMAGING, V40, P1705, DOI 10.1007/s00261-014-0318-3

Venables WN, 2002, MODERN APPL STAT S, DOI DOI 10.1007/978-0-387-21706-2

Wang H, 2005, INT J RADIAT ONCOL, V61, P725, DOI 10.1016/j.ijrobp.2004.07.677

Wang H, 2010, EUR J RADIOL, V74, P124, DOI 10.1016/j.ejrad.2009.01.024

Weiss GJ, 2014, PLOS ONE, V9, DOI 10.1371/journal.pone.0100244

Wickham H, 2009, USE R, P1, DOI 10.1007/978-0-387-98141-3_1

Win T, 2013, CLIN CANCER RES, V19, P3591, DOI 10.1158/1078-0432.CCR-12-1307

Zhang Lifei, 2015, Med Phys, V42, P1341, DOI 10.1118/1.4908210

NR 52

TC 166

Z9 180

U1 2

U2 25

PU NATURE RESEARCH

PI BERLIN

PA HEIDELBERGER PLATZ 3, BERLIN, 14197, GERMANY

SN 2045-2322

J9 SCI REP-UK

JI Sci Rep

PD APR 3

PY 2017

VL 7

AR 588

DI 10.1038/s41598-017-00665-z

PG 11

WC Multidisciplinary Sciences

WE Science Citation Index Expanded (SCI-EXPANDED)

SC Science & Technology - Other Topics

GA EQ5PV

UT WOS:000398136000001

PM 28373718

OA gold, Green Published

DA 2022-08-24

ER

PT J

AU Jiang, YQ

Gao, Q

Chen, H

Shi, XX

Wu, JB

Chen, Y

Zhang, Y

Pang, HW

Lin, S

AF Jiang, Yi-Qing

Gao, Qin

Chen, Han

Shi, Xiang-Xiang

Wu, Jing-Bo

Chen, Yue

Zhang, Yan

Pang, Hao-Wen

Lin, Sheng

TI Positron Emission Tomography-Based Short-Term Efficacy Evaluation and

Prediction in Patients With Non-Small Cell Lung Cancer Treated With

Hypo-Fractionated Radiotherapy

SO FRONTIERS IN ONCOLOGY

LA English

DT Article

DE positron emission tomography; radiomics; non-small-cell lung cancer;

computed tomography; hypo-fractionated radiotherapy

ID STEREOTACTIC BODY RADIOTHERAPY; RESPONSE EVALUATION; METABOLIC BIOPSY;

FDG-PET; RADIOMICS; LESIONS; TOOL

AB Background

Positron emission tomography is known to provide more accurate estimates than computed tomography when staging non-small cell lung cancer. The aims of this prospective study were to contrast the short-term efficacy of the two imaging methods while evaluating the effects of hypo-fractionated radiotherapy in non-small cell lung cancer, and to establish a short-term efficacy prediction model based on the radiomics features of positron emission tomography.

Methods

This nonrandomized-controlled trial was conducted from March 2015 to June 2019. Thirty-one lesions of 30 patients underwent the delineation of the regions of interest on positron emission tomography and computed tomography 1 month before, and 3 months after hypo-fractionated radiotherapy. Each patient was evaluated for the differences in local objective response rate between the two images. The Kaplan Meier method was used to analyze the local objective response and subsequent survival duration of the two imaging methods. The 3D Slicer was used to extract the radiomics features based on positron emission tomography. Least absolute shrinkage and selection operator regression was used to eliminate redundant features, and logistic regression analysis was used to develop the curative-effect-predicting model, which was displayed through a radiomics nomogram. Receiver operating characteristic curve and decision curve were used to evaluate the accuracy and clinical usefulness of the prediction model.

Results

Positron emission tomography-based local objective response rate was significantly higher than that based on computed tomography [70.97% (22/31) and 12.90% (4/31), respectively (p<0.001)]. The mean survival time of responders and non-responders assessed by positron emission tomography was 28.6 months vs. 11.4 months (p=0.29), whereas that assessed by computed tomography was 24.5 months vs. 26 months (p=0.66), respectively. Three radiomics features were screened to establish a personalized prediction nomogram with high area under curve (0.94, 95% CI 0.85-0.99, p<0.001). The decision curve showed a high clinical value of the radiomics nomogram.

Conclusions

We recommend positron emission tomography for evaluating the short-term efficacy of hypo-fractionated radiotherapy in non-small cell lung cancer, and that the radiomics nomogram could be an important technique for the prediction of short-term efficacy, which might enable an improved and precise treatment.

Registration number/URL

ChiCTR1900027768/http://www.chictr.org.cn/showprojen.aspx?proj=46057

C1 [Jiang, Yi-Qing; Gao, Qin; Chen, Han; Shi, Xiang-Xiang; Wu, Jing-Bo; Pang, Hao-Wen; Lin, Sheng] Southwest Med Univ, Affiliated Hosp, Dept Oncol, Luzhou, Peoples R China.

[Chen, Yue; Zhang, Yan; Lin, Sheng] Southwest Med Univ, Nucl Med & Mol Imaging Key Lab Sichuan Prov, Affiliated Hosp, Luzhou, Peoples R China.

RP Pang, HW; Lin, S (通讯作者)，Southwest Med Univ, Affiliated Hosp, Dept Oncol, Luzhou, Peoples R China.; Lin, S (通讯作者)，Southwest Med Univ, Nucl Med & Mol Imaging Key Lab Sichuan Prov, Affiliated Hosp, Luzhou, Peoples R China.

EM haowenpang@foxmail.com; lslinsheng@163.com

FU National Natural Science Foundation of China [81201682]; Scientific

Research Foundation of the Luzhou Science and Technology Bureau

[2016LZXNYD-J05]; Southwest Medical University Foundation [201617]

FX This work was supported by the grants from the National Natural Science

Foundation of China (no. 81201682), the Scientific Research Foundation

of the Luzhou Science and Technology Bureau (no. 2016LZXNYD-J05), and

the Southwest Medical University Foundation (no. 201617).

CR Ahn HK, 2019, CLIN RADIOL, V74, P467, DOI 10.1016/j.crad.2019.02.008

Antunovic L, 2019, EUR J NUCL MED MOL I, V46, P1468, DOI 10.1007/s00259-019-04313-8

Babyak MA, 2004, PSYCHOSOM MED, V66, P411, DOI 10.1097/01.psy.0000127692.23278.a9

Baek S, 2019, SCI REP-UK, V9, DOI 10.1038/s41598-019-53461-2

Balachandran VP, 2015, LANCET ONCOL, V16, pE173, DOI 10.1016/S1470-2045(14)71116-7

Beggs AD, 2002, EUR J NUCL MED MOL I, V29, P542, DOI 10.1007/s00259-001-0736-7

Birim O, 2006, EJSO-EUR J SURG ONC, V32, P12, DOI 10.1016/j.ejso.2005.10.001

Boellaard R, 2010, EUR J NUCL MED MOL I, V37, P181, DOI 10.1007/s00259-009-1297-4

Bradley J, 2012, INT J RADIAT ONCOL, V82, P435, DOI 10.1016/j.ijrobp.2010.09.033

Bray F, 2018, CA-CANCER J CLIN, V68, P394, DOI 10.3322/caac.21492

Chalkidou A, 2015, PLOS ONE, V10, DOI 10.1371/journal.pone.0124165

Depeursinge A, 2020, ARXIV200605470

Detterbeck FC, 2017, CHEST, V151, P193, DOI 10.1016/j.chest.2016.10.010

Dunlap NE, 2012, INT J RADIAT ONCOL, V84, P1071, DOI 10.1016/j.ijrobp.2012.01.088

Eisenhauer EA, 2009, EUR J CANCER, V45, P228, DOI 10.1016/j.ejca.2008.10.026

Ettinger DS, 2014, J NATL COMPR CANC NE, V12, P1738, DOI 10.6004/jnccn.2014.0176

Fedorov A, 2012, MAGN RESON IMAGING, V30, P1323, DOI 10.1016/j.mri.2012.05.001

Fischer BM, 2006, LUNG CANCER, V54, P41, DOI 10.1016/j.lungcan.2006.06.012

Flechsig P, 2017, MOL IMAGING BIOL, V19, P315, DOI 10.1007/s11307-016-0996-z

Giannini V, 2019, EUR J NUCL MED MOL I, V46, P878, DOI 10.1007/s00259-018-4250-6

Gill AB, 2020, CANCERS, V12, DOI 10.3390/cancers12123493

Hain SF, 2001, EUR J NUCL MED, V28, P1336, DOI 10.1007/s002590100563

Kumar V, 2012, MAGN RESON IMAGING, V30, P1234, DOI 10.1016/j.mri.2012.06.010

Lambin P, 2012, EUR J CANCER, V48, P441, DOI 10.1016/j.ejca.2011.11.036

Linda A, 2011, EUR J RADIOL, V79, P147, DOI 10.1016/j.ejrad.2009.10.029

Mac Manus MP, 2003, J CLIN ONCOL, V21, P1285, DOI 10.1200/JCO.2003.07.054

Ohri N, 2016, J NUCL MED, V57, P842, DOI 10.2967/jnumed.115.166934

Ouyang W, 2019, CANCER MED-US, V8, P4605, DOI 10.1002/cam4.2366

Pastis NJ, 2014, CHEST, V146, P406, DOI 10.1378/chest.13-2281

Peng H, 2019, CLIN CANCER RES, V25, P4271, DOI 10.1158/1078-0432.CCR-18-3065

Rajendran JG, 2006, CLIN CANCER RES, V12, P5435, DOI 10.1158/1078-0432.CCR-05-1773

Rundo L, 2017, COMPUT METH PROG BIO, V144, P77, DOI 10.1016/j.cmpb.2017.03.011

Takeda A, 2013, LUNG CANCER, V79, P248, DOI 10.1016/j.lungcan.2012.11.008

Tixier F, 2011, J NUCL MED, V52, P369, DOI 10.2967/jnumed.110.082404

Vesselle H, 2000, CLIN CANCER RES, V6, P3837

Xiang L, 2015, INT J RADIAT ONCOL, V92, P1027, DOI 10.1016/j.ijrobp.2015.04.019

Young H, 1999, EUR J CANCER, V35, P1773, DOI 10.1016/S0959-8049(99)00229-4

Yu W, 2018, INT J RADIAT ONCOL, V102, P1090, DOI 10.1016/j.ijrobp.2017.10.046

Yuan M, 2017, EUR RADIOL, V27, P4857, DOI 10.1007/s00330-017-4855-3

Zwanenburg A., 2016, IMAGE BIOMARKER STAN

Zwanenburg A, 2020, RADIOLOGY, V295, P328, DOI 10.1148/radiol.2020191145

NR 41

TC 1

Z9 1

U1 4

U2 7

PU FRONTIERS MEDIA SA

PI LAUSANNE

PA AVENUE DU TRIBUNAL FEDERAL 34, LAUSANNE, CH-1015, SWITZERLAND

SN 2234-943X

J9 FRONT ONCOL

JI Front. Oncol.

PD FEB 25

PY 2021

VL 11

AR 590836

DI 10.3389/fonc.2021.590836

PG 10

WC Oncology

WE Science Citation Index Expanded (SCI-EXPANDED)

SC Oncology

GA QU5VI

UT WOS:000627349000001

PM 33718144

OA Green Published, gold

DA 2022-08-24

ER

PT J

AU Kawahara, D

Imano, N

Nishioka, R

Ogawa, K

Kimura, T

Nakashima, T

Iwamoto, H

Fujitaka, K

Hattori, N

Nagata, Y

AF Kawahara, Daisuke

Imano, Nobuki

Nishioka, Riku

Ogawa, Kouta

Kimura, Tomoki

Nakashima, Taku

Iwamoto, Hiroshi

Fujitaka, Kazunori

Hattori, Noboru

Nagata, Yasushi

TI Prediction of radiation pneumonitis after definitive radiotherapy for

locally advanced non-small cell lung cancer using multi-region radiomics

analysis

SO SCIENTIFIC REPORTS

LA English

DT Article

ID THERAPY; SELECTION

AB To predict grade >= 2 radiation pneumonitis (RP) in patients with locally advanced non-small cell lung cancer (NSCLC) using multi-region radiomics analysis. Data from 77 patients with NSCLC who underwent definitive radiotherapy between 2008 and 2018 were analyzed. Radiomic feature extraction from the whole lung (whole-lung radiomics analysis) and imaging- and dosimetric-based segmentation (multi-region radiomics analysis) were performed. Patients with RP grade >= 2 or < 2 were classified. Predictors were selected with least absolute shrinkage and selection operator logistic regression and the model was built with neural network classifiers. A total of 49,383 radiomics features per patient image were extracted from the radiotherapy planning computed tomography. We identified 4 features and 13 radiomics features in the whole-lung and multi-region radiomics analysis for classification, respectively. The accuracy and area under the curve (AUC) without the synthetic minority over-sampling technique (SMOTE) were 60.8%, and 0.62 for whole-lung and 80.1%, and 0.84 for multi-region radiomics analysis. These were improved 1.7% for whole-lung and 2.1% for multi-region radiomics analysis with the SMOTE. The developed multi-region radiomics analysis can help predict grade >= 2 RP. The radiomics features in the median- and high-dose regions, and the local intensity roughness and variation were important factors in predicting grade >= 2 RP.

C1 [Kawahara, Daisuke; Imano, Nobuki; Nagata, Yasushi] Hiroshima Univ, Grad Sch Biomed Hlth Sci, Dept Radiat Oncol, 1-3-2 Kagamiyama, Hiroshima 7348551, Japan.

[Nishioka, Riku] Hiroshima Univ, Grad Sch Biomed & Hlth Sci, Med & Dent Sci Course, Hiroshima, Japan.

[Ogawa, Kouta] Hiroshima Univ, Sch Med, Hiroshima, Japan.

[Kimura, Tomoki] Kochi Univ, Kochi Med Sch, Dept Radiol, Div Radiat Oncol, Kochi, Japan.

[Nakashima, Taku; Iwamoto, Hiroshi; Fujitaka, Kazunori; Hattori, Noboru] Hiroshima Univ, Grad Sch Biomed Hlth Sci, Dept Mol & Internal Med, Hiroshima, Japan.

[Nagata, Yasushi] Hiroshima High Precis Radiotherapy Canc Ctr, Hiroshima, Japan.

RP Kawahara, D (通讯作者)，Hiroshima Univ, Grad Sch Biomed Hlth Sci, Dept Radiat Oncol, 1-3-2 Kagamiyama, Hiroshima 7348551, Japan.

EM daika99@hiroshima-u.ac.jp

RI Nakashima, Taku/D-1517-2011

OI Nakashima, Taku/0000-0002-0035-674X

CR Allen AM, 2006, INT J RADIAT ONCOL, V65, P640, DOI 10.1016/j.ijrobp.2006.03.012

Antonia SJ, 2018, NEW ENGL J MED, V379, P2342, DOI 10.1056/NEJMoa1809697

Blagus R, 2013, BMC BIOINFORMATICS, V14, DOI 10.1186/1471-2105-14-106

Chawla NV, 2002, J ARTIF INTELL RES, V16, P321, DOI 10.1613/jair.953

Dang J, 2013, ACTA ONCOL, V52, P1175, DOI 10.3109/0284186X.2012.747696

Gierada DS, 2000, CHEST, V117, P991, DOI 10.1378/chest.117.4.991

Hao HX, 2018, PHYS MED BIOL, V63, DOI 10.1088/1361-6560/aabb5e

Imano N, 2019, J THORAC ONCOL, V14, pS635, DOI 10.1016/j.jtho.2019.08.1338

Jin HK, 2009, RADIOTHER ONCOL, V91, P427, DOI 10.1016/j.radonc.2008.09.009

Kickingereder P, 2016, RADIOLOGY, V281, P907, DOI 10.1148/radiol.2016161382

Krafft SP, 2018, MED PHYS, V45, P5317, DOI 10.1002/mp.13150

Lambin P, 2012, EUR J CANCER, V48, P441, DOI 10.1016/j.ejca.2011.11.036

Liang B, 2019, FRONT ONCOL, V9, DOI 10.3389/fonc.2019.00269

Marks LB, 2010, INT J RADIAT ONCOL, V76, pS70, DOI 10.1016/j.ijrobp.2009.06.091

National Cancer Institute, COMMON TERMINOLOGY C

Nie P, 2020, CANCER IMAGING, V20, DOI 10.1186/s40644-020-00297-z

Park YW, 2019, EUR RADIOL, V29, P4068, DOI 10.1007/s00330-018-5830-3

Rice DC, 2007, INT J RADIAT ONCOL, V69, P350, DOI 10.1016/j.ijrobp.2007.03.011

Shi SM, 2017, TECHNOL CANCER RES T, V16, P316, DOI 10.1177/1533034616661665

Simone CB, 2017, SEMIN RADIAT ONCOL, V27, P370, DOI 10.1016/j.semradonc.2017.04.009

Sura S, 2008, RADIOTHER ONCOL, V87, P17, DOI 10.1016/j.radonc.2008.02.005

Tibshirani R, 2011, J R STAT SOC B, V73, P273, DOI 10.1111/j.1467-9868.2011.00771.x

Tucker SL, 2008, INT J RADIAT ONCOL, V72, P568, DOI 10.1016/j.ijrobp.2008.04.053

van Griethuysen JJM, 2017, CANCER RES, V77, pE104, DOI 10.1158/0008-5472.CAN-17-0339

Xie CY, 2019, EBIOMEDICINE, V44, P289, DOI 10.1016/j.ebiom.2019.05.023

Yao B, 2016, NIGER J CLIN PRACT, V19, P25, DOI 10.4103/1119-3077.173709

Yom SS, 2007, INT J RADIAT ONCOL, V68, P94, DOI 10.1016/j.ijrobp.2006.12.031

Zhang JX, 2013, LANCET ONCOL, V14, P1295, DOI 10.1016/S1470-2045(13)70491-1

NR 28

TC 1

Z9 1

U1 1

U2 5

PU NATURE PORTFOLIO

PI BERLIN

PA HEIDELBERGER PLATZ 3, BERLIN, 14197, GERMANY

SN 2045-2322

J9 SCI REP-UK

JI Sci Rep

PD AUG 10

PY 2021

VL 11

IS 1

AR 16232

DI 10.1038/s41598-021-95643-x

PG 9

WC Multidisciplinary Sciences

WE Science Citation Index Expanded (SCI-EXPANDED)

SC Science & Technology - Other Topics

GA TY6PJ

UT WOS:000683904100015

PM 34376721

OA Green Submitted, gold, Green Published

DA 2022-08-24

ER

PT J

AU van Timmeren, JE

Leijenaar, RTH

van Elmpt, W

Reymen, B

Oberije, C

Monshouwer, R

Bussink, J

Brink, C

Hansen, O

Lambin, P

AF van Timmeren, Janna E.

Leijenaar, Ralph T. H.

van Elmpt, Wouter

Reymen, Bart

Oberije, Cary

Monshouwer, Rene

Bussink, Johan

Brink, Carsten

Hansen, Olfred

Lambin, Philippe

TI Survival prediction of non-small cell lung cancer patients using

radiomics analyses of cone-beam CT images

SO RADIOTHERAPY AND ONCOLOGY

LA English

DT Article

DE Radiomics; Computed tomography; Cone-beam CT; Non-small cell lung

cancer; Survival prediction

ID COMPUTED-TOMOGRAPHY; RADIATION-THERAPY; PROGNOSTIC VALUE; TUMOR

PHENOTYPE; FEATURES; REPRODUCIBILITY; TEXTURE; CARCINOMA; RADIOTHERAPY;

VALIDATION

AB Background and purpose: In this study we investigated the interchangeability of planning CT and cone beam CT (CBCT) extracted radiomic features. Furthermore, a previously described CT based prognostic radiomic signature for non-small cell lung cancer (NSCLC) patients using CBCT based features was validated.

Material and methods: One training dataset of 132 and two validation datasets of 62 and 94 stage I-IV NSCLC patients were included. Interchangeability was assessed by performing a linear regression on CT and CBCT extracted features. A two-step correction was applied prior to model validation of a previously published radiomic signature.

Results: 13.3% (149 out of 1119) of the radiomic features, including all features of the previously published radiomic signature, showed an R-2 above 0.85 between intermodal imaging techniques. For the radiomic signature, Kaplan-Meier curves were significantly different between groups with high and low prognostic value for both modalities. Harrell's concordance index was 0.69 for CT and 0.66 for CBCT models for dataset 1.

Conclusions: The results show that a subset of radiomic features extracted from CT and CBCT images are interchangeable using simple linear regression. Moreover, a previously developed radiomics signature has prognostic value for overall survival in three CBCT cohorts, showing the potential of CBCT radiomics to be used as prognostic imaging biomarker. (C) 2017 The Authors. Published by Elsevier Ireland Ltd.

C1 [van Timmeren, Janna E.; Leijenaar, Ralph T. H.; van Elmpt, Wouter; Reymen, Bart; Oberije, Cary; Lambin, Philippe] Maastricht Univ, Med Ctr, GROW Sch Oncol & Dev Biol, Dept Radiat Oncol MAASTRO, Maastricht, Netherlands.

[Monshouwer, Rene; Bussink, Johan] Radboud Univ Nijmegen, Med Ctr, Dept Radiat Oncol, Nijmegen, Netherlands.

[Brink, Carsten; Hansen, Olfred] Univ Southern Denmark, Inst Clin Res, Odense, Denmark.

[Brink, Carsten; Hansen, Olfred] Odense Univ Hosp, Lab Radiat Phys, Odense, Denmark.

[Hansen, Olfred] Odense Univ Hosp, Dept Oncol, Odense, Denmark.

RP van Timmeren, JE (通讯作者)，Maastricht Univ, Med Ctr, GROW Sch Oncol & Dev Biol, MAASTRO Clin,Dept Radiat Oncol, Dr Tanslaan 12, NL-6229 ET Maastricht, Netherlands.

EM janita.vantimmeren@maastro.nl

RI Monshouwer, R./L-4527-2015; Bussink, Jan/N-3584-2014; Oberije,

Cary/ABA-6178-2020; van Timmeren, Janita/AAL-4456-2020; Hansen,

Olfred/D-1432-2012

OI Oberije, Cary/0000-0003-0749-5117; van Timmeren,

Janita/0000-0002-8166-6853; Hansen, Olfred/0000-0003-0396-1424; Bussink,

Johan/0000-0002-5751-4796; Brink, Carsten/0000-0003-3906-1962; Lambin,

Philippe/0000-0001-7961-0191

FU ERC [694812 - Hypoximmuno]; QuIC-ConCePT project; EFPI A companies

[115151]; Innovative Medicine Initiative Joint Undertaking (IMI JU)

[115151]; Dutch Technology Foundation STW [10696 DuCAT, P14-19 Radiomics

STRaTegy]; Technology Programme of the Ministry of Economic Affairs; EU

7th framework program (ARTFORCE) [257144]; EU 7th framework program

(REQUITE) [601826]; SME Phase 2 (EU) [673780 - RAIL]; European Program

H2020-2015-17 (BD2Decide) [PHC30-689715]; European Program H2020-2015-17

(ImmunoSABR) [733008]; Interreg V-A Euregio Meuse-Rhine

("Eura-diomics"); Kankeronderzoekfonds Limburg from Health Foundation

Limburg; Alpe d'HuZes-KWF (DESIGN); EUROSTARS (DART); Dutch Cancer

Society

FX This work was supported by the ERC advanced grant (ERC-ADG-2015, no

694812 - Hypoximmuno) and the QuIC-ConCePT project, which is partly

funded by EFPI A companies and the Innovative Medicine Initiative Joint

Undertaking (IMI JU) under Grant Agreement No. 115151. This research is

also supported by the Dutch Technology Foundation STW (grant no 10696

DuCAT & no P14-19 Radiomics STRaTegy), which is the applied science

division of NWO, and the Technology Programme of the Ministry of

Economic Affairs. Authors also acknowledge financial support from the EU

7th framework program (ARTFORCE - no 257144, REQUITE - no 601826), SME

Phase 2 (EU proposal 673780 - RAIL), the European Program H2020-2015-17

(BD2Decide - PHC30-689715 and ImmunoSABR - no 733008), Interreg V-A

Euregio Meuse-Rhine ("Eura-diomics"), Kankeronderzoekfonds Limburg from

the Health Foundation Limburg, Alpe d'HuZes-KWF (DESIGN), EUROSTARS

(DART) and the Dutch Cancer Society. Authors thank the contribution of

Anisha Gogineni for editing.

CR Aerts HJWL, 2016, SCI REP-UK, V6, DOI 10.1038/srep33860

Aerts HJWL, 2014, NAT COMMUN, V5, DOI 10.1038/ncomms5006

Antunes J, 2016, TRANSL ONCOL, V9, P155, DOI 10.1016/j.tranon.2016.01.008

Balagurunathan Y, 2014, J DIGIT IMAGING, V27, P805, DOI 10.1007/s10278-014-9716-x

Balagurunathan Y, 2014, TRANSL ONCOL, V7, P72, DOI 10.1593/tlo.13844

Bernchou U, 2015, RADIOTHER ONCOL, V117, P17, DOI 10.1016/j.radonc.2015.07.021

Bertelsen A, 2011, RADIOTHER ONCOL, V100, P351, DOI 10.1016/j.radonc.2011.08.012

Brink C, 2014, INT J RADIAT ONCOL, V89, P916, DOI 10.1016/j.ijrobp.2014.03.038

Cardiac CT, 2016, IMAGING DIAGNOSIS CA

Carvalho S, 2013, ACTA ONCOL, V52, P1398, DOI 10.3109/0284186X.2013.812795

Coroller TP, 2015, RADIOTHER ONCOL, V114, P345, DOI 10.1016/j.radonc.2015.02.015

Cunliffe A, 2015, INT J RADIAT ONCOL, V91, P1048, DOI 10.1016/j.ijrobp.2014.11.030

Fave X, 2015, MED PHYS, V42, P6784, DOI 10.1118/1.4934826

Ferlay J, 2015, INT J CANCER, V136, pE359, DOI 10.1002/ijc.29210

Fried DV, 2014, INT J RADIAT ONCOL, V90, P834, DOI 10.1016/j.ijrobp.2014.07.020

Ganeshan B, 2013, RADIOLOGY, V266, P326, DOI 10.1148/radiol.12112428

Ganeshan B, 2012, EUR RADIOL, V22, P796, DOI 10.1007/s00330-011-2319-8

Gillies RJ, 2016, RADIOLOGY, V278, P563, DOI 10.1148/radiol.2015151169

Jaffray DA, 2002, INT J RADIAT ONCOL, V53, P1337, DOI 10.1016/S0360-3016(02)02884-5

Kumar V, 2012, MAGN RESON IMAGING, V30, P1234, DOI 10.1016/j.mri.2012.06.010

Lambin P, 2016, ADV DRUG DELIV REV

Lambin P, 2013, RADIOTHER ONCOL, V109, P159, DOI 10.1016/j.radonc.2013.07.007

Lambin P, 2013, NAT REV CLIN ONCOL, V10, P27, DOI 10.1038/nrclinonc.2012.196

Lambin P, 2012, EUR J CANCER, V48, P441, DOI 10.1016/j.ejca.2011.11.036

Lee S, 2015, J APPL CLIN MED PHYS, V16, P195, DOI 10.1120/jacmp.v16i6.5620

Leijenaar RTH, 2015, ACTA ONCOL, V54, P1423, DOI 10.3109/0284186X.2015.1061214

Mackin D, 2015, INVEST RADIOL, V50, P757, DOI 10.1097/RLI.0000000000000180

Moteabbed M, 2015, MED PHYS, V42, P196, DOI 10.1118/1.4903292

Oberije C, 2015, INT J RADIAT ONCOL, V92, P935, DOI 10.1016/j.ijrobp.2015.02.048

Oberije C, 2014, RADIOTHER ONCOL, V112, P37, DOI 10.1016/j.radonc.2014.04.012

Royston P., 2013, BMC MED RES METHODOL, V13, p1

Torre LA, 2015, CA-CANCER J CLIN, V65, P87, DOI 10.3322/caac.21262

van Timmeren JE, 2017, DATA SURVIVAL PREDIC

Veiga C, 2014, MED PHYS, V41, DOI 10.1118/1.4864240

Zhao BS, 2016, SCI REP-UK, V6, DOI 10.1038/srep23428

NR 35

TC 99

Z9 99

U1 0

U2 12

PU ELSEVIER IRELAND LTD

PI CLARE

PA ELSEVIER HOUSE, BROOKVALE PLAZA, EAST PARK SHANNON, CO, CLARE, 00000,

IRELAND

SN 0167-8140

EI 1879-0887

J9 RADIOTHER ONCOL

JI Radiother. Oncol.

PD JUN

PY 2017

VL 123

IS 3

BP 363

EP 369

DI 10.1016/j.radonc.2017.04.016

PG 7

WC Oncology; Radiology, Nuclear Medicine & Medical Imaging

WE Science Citation Index Expanded (SCI-EXPANDED)

SC Oncology; Radiology, Nuclear Medicine & Medical Imaging

GA EZ9IL

UT WOS:000405043400004

PM 28506693

OA hybrid, Green Published

DA 2022-08-24

ER

PT J

AU Chang, RS

Qi, SL

Yue, Y

Zhang, XY

Song, JD

Qian, W

AF Chang, Runsheng

Qi, Shouliang

Yue, Yong

Zhang, Xiaoye

Song, Jiangdian

Qian, Wei

TI Predictive Radiomic Models for the Chemotherapy Response in

Non-Small-Cell Lung Cancer based on Computerized-Tomography Images

SO FRONTIERS IN ONCOLOGY

LA English

DT Article

DE lung cancer; radiomics; CT images; chemotherapy response; machine

learning

ID PHASE-III; RADIATION-THERAPY; HETEROGENEITY; CISPLATIN; SURVIVAL;

INFORMATION; TRIAL

AB The heterogeneity and complexity of non-small cell lung cancer (NSCLC) tumors mean that NSCLC patients at the same stage can have different chemotherapy prognoses. Accurate predictive models could recognize NSCLC patients likely to respond to chemotherapy so that they can be given personalized and effective treatment. We propose to identify predictive imaging biomarkers from pre-treatment CT images and construct a radiomic model that can predict the chemotherapy response in NSCLC. This single-center cohort study included 280 NSCLC patients who received first-line chemotherapy treatment. Non-contrast CT images were taken before and after the chemotherapy, and clinical information were collected. Based on the Response Evaluation Criteria in Solid Tumors and clinical criteria, the responses were classified into two categories: response (n = 145) and progression (n = 135), then all data were divided into two cohorts: training cohort (224 patients) and independent test cohort (56 patients). In total, 1629 features characterizing the tumor phenotype were extracted from a cube containing the tumor lesion cropped from the pre-chemotherapy CT images. After dimensionality reduction, predictive models of the chemotherapy response of NSCLC with different feature selection methods and different machine-learning classifiers (support vector machine, random forest, and logistic regression) were constructed. For the independent test cohort, the predictive model based on a random-forest classifier with 20 radiomic features achieved the best performance, with an accuracy of 85.7% and an area under the receiver operating characteristic curve of 0.941 (95% confidence interval, 0.898-0.982). Of the 20 selected features, four were first-order statistics of image intensity and the others were texture features. For nine features, there were significant differences between the response and progression groups (p < 0.001). In the response group, three features, indicating heterogeneity, were overrepresented and one feature indicating homogeneity was underrepresented. The proposed radiomic model with pre-chemotherapy CT features can predict the chemotherapy response of patients with non-small cell lung cancer. This radiomic model can help to stratify patients with NSCLC, thereby offering the prospect of better treatment.

C1 [Chang, Runsheng; Qi, Shouliang; Song, Jiangdian] Northeastern Univ, Coll Med & Biol Informat Engn, Shenyang, Peoples R China.

[Qi, Shouliang] Northeastern Univ, Key Lab Intelligent Comp Med Image, Minist Educ, Shenyang, Peoples R China.

[Yue, Yong] China Med Univ, Dept Radiol, Shengjing Hosp, Shenyang, Peoples R China.

[Zhang, Xiaoye] China Med Univ, Dept Oncol, Shengjing Hosp, Shenyang, Peoples R China.

[Qian, Wei] Univ Texas El Paso, Dept Elect & Comp Engn, El Paso, TX 79968 USA.

RP Qi, SL (通讯作者)，Northeastern Univ, Coll Med & Biol Informat Engn, Shenyang, Peoples R China.; Qi, SL (通讯作者)，Northeastern Univ, Key Lab Intelligent Comp Med Image, Minist Educ, Shenyang, Peoples R China.

EM qisl@bmie.neu.edu.cn

FU National Natural Science Foundation of China [82072008, 81671773,

61672146]; Fundamental Research Funds for the Central Universities

[N2124006-3]

FX This study was supported by the National Natural Science Foundation of

China (Grant number: 82072008, 81671773, 61672146) and the Fundamental

Research Funds for the Central Universities (Grant number: N2124006-3).

CR Aberle DR, 2011, NEW ENGL J MED, V365, P395, DOI 10.1056/NEJMoa1102873

Algohary A, 2020, CANCERS, V12, DOI 10.3390/cancers12082200

Avanzo M, 2017, PHYS MEDICA, V38, P122, DOI 10.1016/j.ejmp.2017.05.071

Bashir U, 2016, AM J ROENTGENOL, V207, P534, DOI 10.2214/AJR.15.15864

Braman NM, 2017, BREAST CANCER RES, V19, DOI 10.1186/s13058-017-0846-1

Bray F, 2018, CA-CANCER J CLIN, V68, P394, DOI 10.3322/caac.21492

Chen AT, 2020, I S BIOMED IMAGING, P678, DOI 10.1109/ISBI45749.2020.9098561

Chetan MR, 2021, EUR RADIOL, V31, P1049, DOI 10.1007/s00330-020-07141-9

Choy H, 2015, LUNG CANCER, V87, P232, DOI 10.1016/j.lungcan.2014.12.003

Deyiaene M, 2019, IEEE ENG MED BIO, P2580, DOI 10.1109/EMBC.2019.8856582

Dong D, 2020, ANN ONCOL, V31, P912, DOI 10.1016/j.annonc.2020.04.003

Dong D, 2019, ANN ONCOL, V30, P431, DOI 10.1093/annonc/mdz001

Ettinger DS, 2013, J NATL COMPR CANC NE, V11, P645, DOI 10.6004/jnccn.2013.0084

Fedorov A, 2012, MAGN RESON IMAGING, V30, P1323, DOI 10.1016/j.mri.2012.05.001

Gatsonis CA, 2011, RADIOLOGY, V258, P243, DOI 10.1148/radiol.10091808

Gillies RJ, 2016, RADIOLOGY, V278, P563, DOI 10.1148/radiol.2015151169

Grossmann P, 2017, ELIFE, V6, DOI 10.7554/eLife.23421

Hosny A, 2018, PLOS MED, V15, DOI 10.1371/journal.pmed.1002711

Hosny A, 2018, NAT REV CANCER, V18, P500, DOI 10.1038/s41568-018-0016-5

Jamal-Hanjani M, 2017, NEW ENGL J MED, V376, P2109, DOI 10.1056/NEJMoa1616288

Junttila MR, 2013, NATURE, V501, P346, DOI 10.1038/nature12626

Khorrami M, 2019, RADIOL-ARTIF INTELL, V1, DOI 10.1148/ryai.2019180012

Lambin P, 2017, NAT REV CLIN ONCOL, V14, P749, DOI 10.1038/nrclinonc.2017.141

Lambin P, 2012, EUR J CANCER, V48, P441, DOI 10.1016/j.ejca.2011.11.036

Lee G, 2017, EUR J RADIOL, V86, P297, DOI 10.1016/j.ejrad.2016.09.005

Lou B, 2019, LANCET DIGIT HEALTH, V1, pE136, DOI 10.1016/S2589-7500(19)30058-5

O'Connor JPB, 2017, SEMIN CELL DEV BIOL, V64, P48, DOI 10.1016/j.semcdb.2016.10.001

O'Connor JPB, 2015, CLIN CANCER RES, V21, P249, DOI 10.1158/1078-0432.CCR-14-0990

Parmar C, 2015, SCI REP-UK, V5, DOI 10.1038/srep13087

Paul R, 2016, TOMOGRAPHY, V2, P388, DOI 10.18383/j.tom.2016.00211

Paz-Ares LG, 2013, J CLIN ONCOL, V31, P2895, DOI 10.1200/JCO.2012.47.1102

Peng HC, 2005, IEEE T PATTERN ANAL, V27, P1226, DOI 10.1109/TPAMI.2005.159

Rigatti Steven J, 2017, J Insur Med, V47, P31, DOI 10.17849/insm-47-01-31-39.1

Scagliotti GV, 2008, J CLIN ONCOL, V26, P3543, DOI 10.1200/JCO.2007.15.0375

Schiller JH, 2002, NEW ENGL J MED, V346, P92, DOI 10.1056/NEJMoa011954

Schwartz LH, 2016, EUR J CANCER, V62, P132, DOI 10.1016/j.ejca.2016.03.081

Seki S, 2020, MAGN RESON MED SCI, V19, P29, DOI 10.2463/mrms.mp.2018-0158

Senan S, 2016, J CLIN ONCOL, V34, P953, DOI 10.1200/JCO.2015.64.8824

Siegel RL, 2022, CA-CANCER J CLIN, V72, P7, DOI [10.3322/caac.21332, 10.3322/caac.21708, 10.3322/caac.21551]

Song JD, 2018, CLIN CANCER RES, V24, P3583, DOI 10.1158/1078-0432.CCR-17-2507

Torre LA, 2016, ADV EXP MED BIOL, V893, P1, DOI 10.1007/978-3-319-24223-1_1

van Griethuysen JJM, 2017, CANCER RES, V77, pE104, DOI 10.1158/0008-5472.CAN-17-0339

Velazquez ER, 2017, CANCER RES, V77, P3922, DOI 10.1158/0008-5472.CAN-17-0122

Wang X, 2020, PHYS MED BIOL, V65, DOI 10.1088/1361-6560/ab6e51

Win T, 2013, CLIN CANCER RES, V19, P3591, DOI 10.1158/1078-0432.CCR-12-1307

Xu YW, 2019, CLIN CANCER RES, V25, P3266, DOI 10.1158/1078-0432.CCR-18-2495

Zhao XZ, 2018, INT J COMPUT ASS RAD, V13, P585, DOI 10.1007/s11548-017-1696-0

Zhao Zhujiang, 2010, J Biomed Biotechnol, V2010, P737535, DOI 10.1155/2010/737535

Zhou QF, 2016, KNOWL-BASED SYST, V95, P1, DOI 10.1016/j.knosys.2015.11.010

Zhou QF, 2014, MECH SYST SIGNAL PR, V46, P82, DOI 10.1016/j.ymssp.2013.12.013

NR 50

TC 5

Z9 5

U1 2

U2 3

PU FRONTIERS MEDIA SA

PI LAUSANNE

PA AVENUE DU TRIBUNAL FEDERAL 34, LAUSANNE, CH-1015, SWITZERLAND

SN 2234-943X

J9 FRONT ONCOL

JI Front. Oncol.

PD JUL 7

PY 2021

VL 11

AR 646190

DI 10.3389/fonc.2021.646190

PG 13

WC Oncology

WE Science Citation Index Expanded (SCI-EXPANDED)

SC Oncology

GA TR5FI

UT WOS:000678989400001

PM 34307127

OA gold, Green Published

DA 2022-08-24

ER

PT J

AU Zarinshenas, R

Ladbury, C

McGee, H

Raz, D

Erhunmwunsee, L

Pathak, R

Glaser, S

Salgia, R

Williams, T

Amini, A

AF Zarinshenas, Reza

Ladbury, Colton

McGee, Heather

Raz, Dan

Erhunmwunsee, Loretta

Pathak, Ranjan

Glaser, Scott

Salgia, Ravi

Williams, Terence

Amini, Arya

TI Machine learning to refine prognostic and predictive nodal burden

thresholds for post-operative radiotherapy in completely resected stage

III-N2 non-small cell lung cancer

SO RADIOTHERAPY AND ONCOLOGY

LA English

DT Article

DE Machine learning; Big data; NCDB; PORT; NSCLC

ID LYMPH-NODES; ADJUVANT CHEMOTHERAPY; RADIATION-THERAPY; SURVIVAL;

ASSOCIATION; NUMBER; IMPACT

AB Background: The role of post-operative radiotherapy (PORT) for completely resected N2 non-small-cell lung cancer (NSCLC) is controversial in light of recent randomized data. We sought to utilize machine learning to identify a subset of patients who may still benefit from PORT based on extent of nodal involvement. Materials/Methods: Patients with completely resected N2 NSCLC were identified in the National Cancer Database. We trained a machine-learning based model of overall survival (OS). SHapley Additive exPlanation (SHAP) values were used to identify prognostic and predictive thresholds of number of positive lymph nodes (LNs) involved and lymph node ratio (LNR). Cox proportional hazards regression was used for confirmatory analysis. Results: A total of 16,789 patients with completely resected N2 NSCLC were identified. Using the SHAP values, we identified thresholds of 3+ positive LNs and a LNR of 0.34+. On multivariate analysis, PORT was not significantly associated with OS (p = 0.111). However, on subset analysis of patients with 3+ positive LNs, PORT improved OS (HR: 0.91; 95% CI: 0.86-0.97; p = 0.002). On a separate subset analysis in patients with a LNR of 0.34+, PORT improved OS (HR: 0.90; 95% CI: 0.85-0.96; p = 0.001). Patients with 3+ positive lymph nodes had a 5-year OS of 38% with PORT compared to 31% without PORT. Patient with positive lymph node ratio 0.34+ had a 5-year OS of 38% with PORT compared to 29% without PORT. Conclusions: Patients with a high lymph node burden or lymph node ratio may present a subpopulation of patients who could benefit from PORT. To our knowledge, this is the first study to use machine learning algorithms to address this question with a large national dataset. These findings address an important question in the field of thoracic oncology and warrant further investigation in prospective studies.(c) 2022 Elsevier B.V. All rights reserved. Radiotherapy and Oncology 173 (2022) 10-18

C1 [Zarinshenas, Reza; Ladbury, Colton; McGee, Heather; Glaser, Scott; Williams, Terence; Amini, Arya] City Hope Natl Med Ctr, Dept Radiat Oncol, Ft Lauderdale, FL USA.

[Raz, Dan; Erhunmwunsee, Loretta] City Hope Natl Med Ctr, Dept Surg, Ft Lauderdale, FL USA.

[Erhunmwunsee, Loretta] City Hope Natl Med Ctr, Dept Populat Sci, Ft Lauderdale, FL USA.

[Pathak, Ranjan; Salgia, Ravi] City Hope Natl Med Ctr, Dept Med Oncol, Duarte, CA USA.

[Amini, Arya] City Hope Natl Med Ctr Radiat Oncol, 1500 E Duarte Rd, Duarte, CA 91010 USA.

RP Amini, A (通讯作者)，City Hope Natl Med Ctr Radiat Oncol, 1500 E Duarte Rd, Duarte, CA 91010 USA.

EM aamini@coh.org

CR Bilimoria KY, 2008, ANN SURG ONCOL, V15, P683, DOI 10.1245/s10434-007-9747-3

Billiet C, 2014, RADIOTHER ONCOL, V110, P3, DOI 10.1016/j.radonc.2013.08.011

Burdett S, 1998, LANCET, V352, P257

Corso CD, 2015, J THORAC ONCOL, V10, P148, DOI 10.1097/JTO.0000000000000406

Dai HH, 2011, ONCOLOGIST, V16, P641, DOI 10.1634/theoncologist.2010-0343

Douillard JY, 2008, INT J RADIAT ONCOL, V72, P695, DOI 10.1016/j.ijrobp.2008.01.044

Fukui T, 2006, J THORAC ONCOL, V1, P120, DOI 10.1097/01243894-200602000-00004

HARRELL FE, 1984, STAT MED, V3, P143, DOI 10.1002/sim.4780030207

HARRELL FE, 1982, JAMA-J AM MED ASSOC, V247, P2543, DOI 10.1001/jama.247.18.2543

Harrell FE, 1996, STAT MED, V15, P361, DOI 10.1002/(SICI)1097-0258(19960229)15:4<361::AID-SIM168>3.0.CO;2-4

Hui ZG, 2021, JAMA ONCOL, V7, P1178, DOI 10.1001/jamaoncol.2021.1910

Hui ZG, 2015, THORAC CANCER, V6, P346, DOI 10.1111/1759-7714.12186

Lally BE, 2006, J CLIN ONCOL, V24, P2998, DOI 10.1200/JCO.2005.04.6110

Le Pechoux C, 2021, LANCET ONCOL

Lee JG, 2008, ANN THORAC SURG, V85, P211, DOI 10.1016/j.athoracsur.2007.08.020

Li R, 2020, JCO CLIN CANCER INFO, V4, P637, DOI 10.1200/CCI.20.00002

Lundberg SM, 2018, NAT BIOMED ENG, V2, P749, DOI 10.1038/s41551-018-0304-0

Matsuguma Haruhisa, 2008, Interact Cardiovasc Thorac Surg, V7, P573, DOI 10.1510/icvts.2007.174342

Mikell JL, 2015, J THORAC ONCOL, V10, P462, DOI 10.1097/JTO.0000000000000411

Moncada-Torres A, 2021, SCI REP-UK, V11, DOI 10.1038/s41598-021-86327-7

Patel SH, 2014, LUNG CANCER, V84, P156, DOI 10.1016/j.lungcan.2014.02.016

Robinson CG, 2015, J CLIN ONCOL, V33, P870, DOI 10.1200/JCO.2014.58.5380

Sakib N, 2018, NUCL MED COMMUN, V39, P51, DOI 10.1097/MNM.0000000000000764

Shinde A, 2019, LUNG CANCER, V133, P136, DOI 10.1016/j.lungcan.2019.05.020

Suzuki K, 1999, J THORAC CARDIOV SUR, V118, P145, DOI 10.1016/S0022-5223(99)70153-4

Urban D, 2013, J THORAC ONCOL, V8, P940, DOI 10.1097/JTO.0b013e318292c53e

Wei SH, 2011, J THORAC ONCOL, V6, P310, DOI 10.1097/JTO.0b013e3181ff9b45

Xu YJ, 2018, ONCOL LETT, V15, P2641, DOI 10.3892/ol.2017.7601

NR 28

TC 0

Z9 0

U1 1

U2 1

PU ELSEVIER IRELAND LTD

PI CLARE

PA ELSEVIER HOUSE, BROOKVALE PLAZA, EAST PARK SHANNON, CO, CLARE, 00000,

IRELAND

SN 0167-8140

EI 1879-0887

J9 RADIOTHER ONCOL

JI Radiother. Oncol.

PD AUG

PY 2022

VL 173

BP 10

EP 18

DI 10.1016/j.radonc.2022.05.019

PG 9

WC Oncology; Radiology, Nuclear Medicine & Medical Imaging

WE Science Citation Index Expanded (SCI-EXPANDED)

SC Oncology; Radiology, Nuclear Medicine & Medical Imaging

GA 1Z2XE

UT WOS:000808692500002

PM 35618098

DA 2022-08-24

ER

PT J

AU Krarup, MMK

Nygard, L

Vogelius, IR

Andersen, FL

Cook, G

Goh, V

Fischer, BM

AF Krarup, Marie Manon Krebs

Nygard, Lotte

Vogelius, Ivan Richter

Andersen, Flemming Littrup

Cook, Gary

Goh, Vicky

Fischer, Barbara Malene

TI Heterogeneity in tumours: Validating the use of radiomic features on

F-18-FDG PET/CT scans of lung cancer patients as a prognostic tool

SO RADIOTHERAPY AND ONCOLOGY

LA English

DT Article

DE Positron Emission Tomography Computed; Tomography; Carcinoma, Non Small

Cell Lung; Prognosis; Texture features; Heterogeneity; Radiomics

ID TEXTURAL FEATURES; FDG-PET; ESOPHAGEAL CANCER; IMAGES; RECONSTRUCTION;

ASPHERICITY; IMPACT; VOLUME; QUANTIFICATION; PREDICTION

AB Aim: The aim was to validate promising radiomic features (RFs)(1) on F-18-flourodeoxyglucose positron emission tomography/computed tomography-scans (F-18-FDG PET/CT) of non-small cell lung cancer (NSCLC) patients undergoing definitive chemo-radiotherapy.

Methods: F-18-FDG PET/CT scans performed for radiotherapy (RT) planning were retrieved. Auto-segmentation with visual adaption was used to define the primary tumour on PET images. Six preselected prognostic and reproducible PET texture -and shape-features were calculated using texture respectively shape analysis. The correlation between these RFs and metabolic active tumour volume (MTV)(3), gross tumour volume (GTV)(4) and maximum and mean of standardized uptake value (SUV)(5) was tested with a Spearman's Rank test. The prognostic value of RFs was tested in a univariate cox regression analysis and a multivariate cox regression analysis with GTV, clinical stage and histology. P-value <= 0.05 were considered significant.

Results: Image analysis was performed for 233 patients: 145 males and 88 females, mean age of 65.7 and clinical stage II-IV. Mean GTV was 129.87 cm(3) (SD 130.30 cm(3)). Texture and shape-features correlated more strongly to MTV and GTV compared to SUV-measurements. Four RFs predicted PFS in the univariate analysis. No RFs predicted PFS in the multivariate analysis, whereas GTV and clinical stage predicted PFS (p = 0.001 and p = 0.008 respectively).

Conclusion: The pre-selected RFs were insignificant in predicting PFS in combination with GTV, clinical stage and histology. These results might be due to variations in technical parameters. However, it is relevant to question whether RFs are stable enough to provide clinically useful information. (C) 2019 Elsevier B.V. All rights reserved.

C1 [Krarup, Marie Manon Krebs; Andersen, Flemming Littrup; Fischer, Barbara Malene] Rigshosp, Dept Clin Physiol Nucl Med & PET, Copenhagen, Denmark.

[Nygard, Lotte; Vogelius, Ivan Richter] Rigshosp, Dept Oncol, Copenhagen, Denmark.

[Vogelius, Ivan Richter] Univ Copenhagen, Fac Hlth & Med Sci, Copenhagen, Denmark.

[Cook, Gary; Goh, Vicky; Fischer, Barbara Malene] Kings Coll London, St Thomas Hosp, Sch Biomed Engn & Imaging Sci, PET Ctr, Westminster Bridge Rd, London SE1 7EH, England.

RP Krarup, MMK (通讯作者)，Copenhagen Univ Hosp, Rigshosp, Dept Clin Physiol Nucl Med & PET, Copenhagen, Denmark.

EM marie.manon.krebs.krarup.01@regionh.dk; lotte.nygaard@regionh.dk;

ivan.richter.vogelius@regionh.dk; flemming.andersen@regionh.dk;

gary.cook@kcl.ac.uk; vicky.goh@kcl.ac.uk; malene.fischer@kcl.ac.uk

OI Krarup, Marie Manon Krebs/0000-0001-5670-3280; Vogelius, Ivan

Richter/0000-0002-8877-1218; Goh, Vicky/0000-0002-2321-8091; Andersen,

Flemming Littrup/0000-0003-2821-1849; Cook, Gary/0000-0002-8732-8134;

Fischer, Barbara Malene/0000-0002-6065-3375

FU Danish Capital Region; Scandinavian Society of Clinical Physiology and

Nuclear Medicine (SSCPNM)

FX The study was supported with funds from the Danish Capital Region,

(administered by the Department of Clinical Medicine, University of

Copenhagen) earmarked for research with an international focus. The

Scandinavian Society of Clinical Physiology and Nuclear Medicine

(SSCPNM) supported us with a travel grand. The sponsors had no

involvement in the study or in the decision to submit the article for

publication.

CR Aerts HJWL, 2014, NAT COMMUN, V5, DOI 10.1038/ncomms5006

[Anonymous], 2014, REV MAN REVMAN

[Anonymous], [No title captured]

[Anonymous], 2018, TEXTURE USER GUIDE L

Apostolova I, 2016, EUR J NUCL MED MOL I, V43, P2360, DOI 10.1007/s00259-016-3452-z

Apostolova I, 2014, BMC CANCER, V14, DOI 10.1186/1471-2407-14-896

Apostolova I, 2014, EUR RADIOL, V24, P2077, DOI 10.1007/s00330-014-3269-8

Bailly C, 2016, PLOS ONE, V11, DOI 10.1371/journal.pone.0159984

Beukinga RJ, 2017, J NUCL MED, V58, P723, DOI 10.2967/jnumed.116.180299

Brooks FJ, 2014, J NUCL MED, V55, P37, DOI 10.2967/jnumed.112.116715

Chalkidou A, 2015, PLOS ONE, V10, DOI 10.1371/journal.pone.0124165

Choi ER, 2016, ONCOTARGET, V7, P67302, DOI 10.18632/oncotarget.11693

Cook GJR, 2018, INT J RADIAT ONCOL, V102, P1083, DOI 10.1016/j.ijrobp.2017.12.268

Cook GJR, 2013, J NUCL MED, V54, P19, DOI 10.2967/jnumed.112.107375

Davnall F, 2012, INSIGHTS IMAGING, V3, P573, DOI 10.1007/s13244-012-0196-6

Desseroit MC, 2016, EUR J NUCL MED MOL I, V43, P1477, DOI 10.1007/s00259-016-3325-5

Dong XZ, 2015, J MED IMAG RADIAT ON, V59, P338, DOI 10.1111/1754-9485.12289

Doumou G, 2015, EUR RADIOL, V25, P2805, DOI 10.1007/s00330-015-3681-8

Fried DV, 2016, RADIOLOGY, V278, P214, DOI 10.1148/radiol.2015142920

Galavis PE, 2010, ACTA ONCOL, V49, P1012, DOI 10.3109/0284186X.2010.498437

Galloway M., 1975, COMPUT VISION GRAPH, V4, P172, DOI [10.1016/S0146-664X(75)80008-6, DOI 10.1016/S0146-664X(75)80008-6]

Ganeshan B, 2013, RADIOLOGY, V266, P326, DOI 10.1148/radiol.12112428

Han S, 2018, ANN NUCL MED, V32, P602, DOI 10.1007/s12149-018-1281-9

HARALICK RM, 1973, IEEE T SYST MAN CYB, VSMC3, P610, DOI 10.1109/TSMC.1973.4309314

Hatt M, 2018, EUR J NUCL MED MOL I, V45, P630, DOI 10.1007/s00259-017-3865-3

Hatt M, 2017, EUR J NUCL MED MOL I, V44, P151, DOI 10.1007/s00259-016-3427-0

Hatt M, 2015, J NUCL MED, V56, P38, DOI 10.2967/jnumed.114.144055

Hatt M, 2013, EUR J NUCL MED MOL I, V40, P1662, DOI 10.1007/s00259-013-2486-8

Hatt M, 2011, EUR J NUCL MED MOL I, V38, P663, DOI 10.1007/s00259-010-1688-6

Hofheinz F, 2015, EUR J NUCL MED MOL I, V42, P429, DOI 10.1007/s00259-014-2953-x

Lambin P, 2012, EUR J CANCER, V48, P441, DOI 10.1016/j.ejca.2011.11.036

Larue RTHM, 2017, BRIT J RADIOL, V90, DOI 10.1259/bjr.20160665

Lasnon C, 2016, EUR J NUCL MED MOL I, V43, P2324, DOI 10.1007/s00259-016-3441-2

Leijenaar RTH, 2013, ACTA ONCOL, V52, P1391, DOI 10.3109/0284186X.2013.812798

Lemarignier C, 2017, EUR J NUCL MED MOL I, V44, P1145, DOI 10.1007/s00259-017-3641-4

Lovinfosse P, 2016, EUR J NUCL MED MOL I, V43, P1453, DOI 10.1007/s00259-016-3314-8

Nakajo M, 2017, EUR J NUCL MED MOL I, V44, P206, DOI 10.1007/s00259-016-3506-2

Nioche C, 2018, CANCER RES, V78, P4786, DOI 10.1158/0008-5472.CAN-18-0125

Nyflot MJ, 2015, J MED IMAGING, V2, DOI 10.1117/1.JMI.2.4.041002

Nygard L, 2016, RADIOTHER ONCOL, V118, P460, DOI 10.1016/j.radonc.2016.01.009

Orlhac F, 2018, J NUCL MED, V59, P1321, DOI 10.2967/jnumed.117.199935

Orlhac F, 2015, PLOS ONE, V10, DOI 10.1371/journal.pone.0145063

Orlhac F, 2014, J NUCL MED, V55, P414, DOI 10.2967/jnumed.113.129858

Parmar C, 2015, SCI REP-UK, V5, DOI 10.1038/srep11044

Pyka T, 2015, RADIAT ONCOL, V10, DOI 10.1186/s13014-015-0407-7

Reuze S, 2018, INT J RADIAT ONCOL, V102, P1117, DOI 10.1016/j.ijrobp.2018.05.022

Reuze S, 2017, ONCOTARGET, V8, P43169, DOI 10.18632/oncotarget.17856

Sollini M, 2017, SCI REP-UK, V7, DOI 10.1038/s41598-017-00426-y

TAYLOR R, 1990, J DIAGN MED SONOG, V6, P35, DOI 10.1177/875647939000600106

Tixier F, 2014, J NUCL MED, V55, P1235, DOI 10.2967/jnumed.113.133389

Tixier F, 2012, J NUCL MED, V53, P693, DOI 10.2967/jnumed.111.099127

van Velden FHP, 2016, MOL IMAGING BIOL, V18, P788, DOI 10.1007/s11307-016-0940-2

Yan JH, 2015, J NUCL MED, V56, P1667, DOI 10.2967/jnumed.115.156927

NR 53

TC 22

Z9 23

U1 2

U2 8

PU ELSEVIER IRELAND LTD

PI CLARE

PA ELSEVIER HOUSE, BROOKVALE PLAZA, EAST PARK SHANNON, CO, CLARE, 00000,

IRELAND

SN 0167-8140

EI 1879-0887

J9 RADIOTHER ONCOL

JI Radiother. Oncol.

PD MAR

PY 2020

VL 144

BP 72

EP 78

DI 10.1016/j.radonc.2019.10.012

PG 7

WC Oncology; Radiology, Nuclear Medicine & Medical Imaging

WE Science Citation Index Expanded (SCI-EXPANDED)

SC Oncology; Radiology, Nuclear Medicine & Medical Imaging

GA KU2GK

UT WOS:000519526500011

PM 31733491

DA 2022-08-24

ER

PT J

AU Yang, H

Wang, L

Shao, GL

Dong, BQ

Wang, F

Wei, YG

Li, P

Chen, HY

Chen, WJ

Zheng, Y

He, YW

Zhao, YK

Du, XH

Sun, XJ

Wang, Z

Wang, YZ

Zhou, X

Lai, XJ

Feng, W

Shen, LM

Qiu, GQ

Ji, YL

Chen, JX

Jiang, YH

Liu, JS

Zeng, J

Wang, CC

Zhao, Q

Yang, X

Hu, X

Ma, HL

Chen, QX

Chen, M

Jiang, HT

Xu, YJ

AF Yang, Hong

Wang, Lin

Shao, Guoliang

Dong, Baiqiang

Wang, Fang

Wei, Yuguo

Li, Pu

Chen, Haiyan

Chen, Wujie

Zheng, Yao

He, Yiwei

Zhao, Yankun

Du, Xianghui

Sun, Xiaojiang

Wang, Zhun

Wang, Yuezhen

Zhou, Xia

Lai, Xiaojing

Feng, Wei

Shen, Liming

Qiu, Guoqing

Ji, Yongling

Chen, Jianxiang

Jiang, Youhua

Liu, Jinshi

Zeng, Jian

Wang, Changchun

Zhao, Qiang

Yang, Xun

Hu, Xiao

Ma, Honglian

Chen, Qixun

Chen, Ming

Jiang, Haitao

Xu, Yujin

TI A combined predictive model based on radiomics features and clinical

factors for disease progression in early-stage non-small cell lung

cancer treated with stereotactic ablative radiotherapy

SO FRONTIERS IN ONCOLOGY

LA English

DT Article

DE non-small cell lung cancer; stereotactic ablative radiotherapy;

progression; radiomics; predictive model

ID BODY RADIATION-THERAPY; PHASE-II TRIAL; LOCAL RECURRENCE; OUTCOMES;

SURVIVAL; SURGERY; SABR; SBRT

AB PurposeTo accurately assess disease progression after Stereotactic Ablative Radiotherapy (SABR) of early-stage Non-Small Cell Lung Cancer (NSCLC), a combined predictive model based on pre-treatment CT radiomics features and clinical factors was established. MethodsThis study retrospectively analyzed the data of 96 patients with early-stage NSCLC treated with SABR. Clinical factors included general information (e.g. gender, age, KPS, Charlson score, lung function, smoking status), pre-treatment lesion status (e.g. diameter, location, pathological type, T stage), radiation parameters (biological effective dose, BED), the type of peritumoral radiation-induced lung injury (RILI). Independent risk factors were screened by logistic regression analysis. Radiomics features were extracted from pre-treatment CT. The minimum Redundancy Maximum Relevance (mRMR) and the Least Absolute Shrinkage and Selection Operator (LASSO) were adopted for the dimensionality reduction and feature selection. According to the weight coefficient of the features, the Radscore was calculated, and the radiomics model was constructed. Multiple logistic regression analysis was applied to establish the combined model based on radiomics features and clinical factors. Receiver Operating Characteristic (ROC) curve, DeLong test, Hosmer-Lemeshow test, and Decision Curve Analysis (DCA) were used to evaluate the model's diagnostic efficiency and clinical practicability. ResultsWith the median follow-up of 59.1 months, 29 patients developed progression and 67 remained good controlled within two years. Among the clinical factors, the type of peritumoral RILI was the only independent risk factor for progression (P< 0.05). Eleven features were selected from 1781 features to construct a radiomics model. For predicting disease progression after SABR, the Area Under the Curve (AUC) of training and validation cohorts in the radiomics model was 0.88 (95%CI 0.80-0.96) and 0.80 (95%CI 0.62-0.98), and AUC of training and validation cohorts in the combined model were 0.88 (95%CI 0.81-0.96) and 0.81 (95%CI 0.62-0.99). Both the radiomics and the combined models have good prediction efficiency in the training and validation cohorts. Still, DeLong test shows that there is no difference between them. ConclusionsCompared with the clinical model, the radiomics model and the combined model can better predict the disease progression of early-stage NSCLC after SABR, which might contribute to individualized follow-up plans and treatment strategies.

C1 [Yang, Hong; Shao, Guoliang; Wang, Fang; Chen, Haiyan; Chen, Wujie; Zheng, Yao; He, Yiwei; Zhao, Yankun; Jiang, Haitao] Univ Chinese Acad Sci, Canc Hosp, Zhejiang Canc Hosp, Inst Basic Med & Canc IBMC,Dept Radiol,Chinese Aca, Hangzhou, Peoples R China.

[Wang, Lin] Shaoxing Univ, Sch Med, Shaoxing, Peoples R China.

[Dong, Baiqiang; Chen, Ming] Sun Yat sen Univ, Collaborat Innovat Ctr Canc Med, Canc Ctr, Dept Radiat Oncol,tate Key Lab Oncol South China,, Guangzhou, Peoples R China.

[Wei, Yuguo] Precis Hlth Inst, Gen Elect GE Healthcare, Hangzhou, Peoples R China.

[Li, Pu] Univ Chinese Acad Sci, Canc Hosp, Zhejiang Canc Hosp, Inst Basic Med & Canc IBMC,Dept Radiat Phys,Chines, Hangzhou, Peoples R China.

[Du, Xianghui; Sun, Xiaojiang; Wang, Zhun; Wang, Yuezhen; Zhou, Xia; Lai, Xiaojing; Feng, Wei; Shen, Liming; Qiu, Guoqing; Ji, Yongling; Chen, Jianxiang; Hu, Xiao; Ma, Honglian; Xu, Yujin] Univ Chinese Acad Sci, Canc Hosp, Zhejiang Canc Hosp, Inst Basic Med & Canc IBMC,Chinese Acad Sci,Dept, Hangzhou, Peoples R China.

[Jiang, Youhua; Liu, Jinshi; Zeng, Jian; Wang, Changchun; Zhao, Qiang; Yang, Xun; Chen, Qixun] Univ Chinese Acad Sci, Canc Hosp, Zhejiang Canc Hosp, Inst Basic Med & Canc IBMC,Chinese Acad Sci,Dept T, Hangzhou, Peoples R China.

RP Jiang, HT (通讯作者)，Univ Chinese Acad Sci, Canc Hosp, Zhejiang Canc Hosp, Inst Basic Med & Canc IBMC,Dept Radiol,Chinese Aca, Hangzhou, Peoples R China.; Xu, YJ (通讯作者)，Univ Chinese Acad Sci, Canc Hosp, Zhejiang Canc Hosp, Inst Basic Med & Canc IBMC,Chinese Acad Sci,Dept, Hangzhou, Peoples R China.

EM jianght@zjcc.org.cn; xuyj@zjcc.org.cn

FU Medical and Health Research Project of Zhejiang Province; Beijing Xisike

Clinical Oncology Research Foundation; Beijing Science and Technology

Innovation Medical Development Foundation; [2020KY486]; [2020KY079];

[Y-2019AZMS-0061]; [KC2021-JX-0186-63]

FX Funding This study was supported by grants from Medical and Health

Research Project of Zhejiang Province (Grant Number: 2020KY486 ;

2020KY079); Beijing Xisike Clinical Oncology Research Foundation

(Y-2019AZMS-0061); and Beijing Science and Technology Innovation Medical

Development Foundation (KC2021-JX-0186-63).

CR Abel S, 2019, LUNG CANCER, V128, P127, DOI 10.1016/j.lungcan.2018.12.022

Ardakani AA, 2022, COMPUT METH PROG BIO, V215, DOI 10.1016/j.cmpb.2021.106609

Avanzo M, 2017, PHYS MEDICA, V38, P122, DOI 10.1016/j.ejmp.2017.05.071

Baumann P, 2009, J CLIN ONCOL, V27, P3290, DOI 10.1200/JCO.2008.21.5681

Bhatt AD, 2015, AM J CLIN ONCOL-CANC, V38, P41, DOI 10.1097/COC.0b013e318287bd7f

Chang JY, 2012, RADIAT ONCOL, V7, DOI 10.1186/1748-717X-7-152

Chicas-Sett R, 2019, INT J MOL SCI, V20, DOI 10.3390/ijms20092173

Dahele M, 2011, J THORAC ONCOL, V6, P1221, DOI 10.1097/JTO.0b013e318219aac5

Grills IS, 2010, J CLIN ONCOL, V28, P928, DOI 10.1200/JCO.2009.25.0928

Grills IS, 2012, J THORAC ONCOL, V7, P1382, DOI 10.1097/JTO.0b013e318260e00d

Halpenny D, 2015, CLIN IMAG, V39, P254, DOI 10.1016/j.clinimag.2014.12.005

Huang K, 2012, RADIOTHER ONCOL, V102, P335, DOI 10.1016/j.radonc.2011.12.018

Kadoya N, 2020, MED PHYS, V47, P2197, DOI 10.1002/mp.14104

Kang JJ, 2020, INT J RADIAT ONCOL, V106, P90, DOI 10.1016/j.ijrobp.2019.09.037

Kato S, 2010, JPN J RADIOL, V28, P259, DOI 10.1007/s11604-009-0415-3

Klement RJ, 2014, INT J RADIAT ONCOL, V88, P732, DOI 10.1016/j.ijrobp.2013.11.216

Lafata KJ, 2019, PHYS MED BIOL, V64, DOI 10.1088/1361-6560/aaf5a5

Lambin P, 2017, NAT REV CLIN ONCOL, V14, P749, DOI 10.1038/nrclinonc.2017.141

Li Q, 2017, MED PHYS, V44, P4341, DOI 10.1002/mp.12309

Limkin EJ, 2017, ANN ONCOL, V28, P1191, DOI 10.1093/annonc/mdx034

Liu J, 2019, MED PHYS, V46, P3091, DOI 10.1002/mp.13551

Luo LM, 2022, FRONT ONCOL, V11, DOI 10.3389/fonc.2021.819047

Ma LF, 2016, THORAC CANCER, V7, P442, DOI 10.1111/1759-7714.12352

Onishi H, 2004, CANCER-AM CANCER SOC, V101, P1623, DOI 10.1002/cncr.20539

Palma DA, 2011, INT J RADIAT ONCOL, V80, P506, DOI 10.1016/j.ijrobp.2010.02.032

Rossi G, 2021, CANCER RES, V81, P724, DOI 10.1158/0008-5472.CAN-20-0999

Scott WJ, 2007, CHEST, V132, p234S, DOI 10.1378/chest.07-1378

Senthi S, 2012, LANCET ONCOL, V13, P802, DOI 10.1016/S1470-2045(12)70242-5

Sung H, 2021, CA-CANCER J CLIN, V71, P209, DOI 10.3322/caac.21660

Timmerman RD, 2014, INT J RADIAT ONCOL, V90, pS30, DOI 10.1016/j.ijrobp.2014.05.135

van Griethuysen JJM, 2017, CANCER RES, V77, pE104, DOI 10.1158/0008-5472.CAN-17-0339

van Timmeren JE, 2019, RADIOTHER ONCOL, V136, P78, DOI 10.1016/j.radonc.2019.03.032

Yang Y, 2021, FRONT ONCOL, V11, DOI 10.3389/fonc.2021.746785

Zheng XP, 2014, INT J RADIAT ONCOL, V90, P603, DOI 10.1016/j.ijrobp.2014.05.055

NR 34

TC 0

Z9 0

U1 0

U2 0

PU FRONTIERS MEDIA SA

PI LAUSANNE

PA AVENUE DU TRIBUNAL FEDERAL 34, LAUSANNE, CH-1015, SWITZERLAND

SN 2234-943X

J9 FRONT ONCOL

JI Front. Oncol.

PD AUG 2

PY 2022

VL 12

AR 967360

DI 10.3389/fonc.2022.967360

PG 13

WC Oncology

WE Science Citation Index Expanded (SCI-EXPANDED)

SC Oncology

GA 3U6AW

UT WOS:000841052800001

PM 35982975

OA Green Accepted, gold

DA 2022-08-24

ER

PT J

AU Moran, A

Wang, YC

Dyer, BA

Yip, SSF

Daly, ME

Yamamoto, T

AF Moran, Angel

Wang, Yichuan

Dyer, Brandon A.

Yip, Stephen S. F.

Daly, Megan E.

Yamamoto, Tokihiro

TI Prognostic Value of Computed Tomography and/or F-18-Fluorodeoxyglucose

Positron Emission Tomography Radiomics Features in Locally Advanced

Non-small Cell Lung Cancer

SO CLINICAL LUNG CANCER

LA English

DT Article

DE Carcinoma; Chemoradiotherapy; Imaging; Survival analysis; Texture

features

ID STANDARDIZED UPTAKE VALUES; FDG-PET; TEXTURAL FEATURES; TUMOR

CHARACTERISTICS; F-18-FDG PET; RADIOTHERAPY; HETEROGENEITY; PREDICTION;

RECONSTRUCTION; ACQUISITION

AB We compared the prognostic value of computed tomography (CT) and F-18-fluorodeoxyglucose positron emission tomography (PET) radiomics features for patients with locally advanced non-small cell lung cancer treated with chemoradiotherapy. This 39-patient study demonstrated that adding PET radiomics features to conventional factors significantly improved the prognostic value versus conventional factors alone; adding CT radiomics features did not improve accuracy.

Introduction: We investigated whether adding computed tomography (CT) and/or F-18-fluorodeoxyglucose (F-18-FDG) PET radiomics features to conventional prognostic factors (CPFs) improves prognostic value in locally advanced nonsmall cell lung cancer (NSCLC). Materials and Methods: We retrospectively identified 39 cases with stage III NSCLC who received chemoradiotherapy and underwent planning CT and staging F-18-FDG PET scans. Seven CPFs were recorded. Feature selection was performed on 48 CT and 49 PET extracted radiomics features. A penalized multivariate Cox proportional hazards model was used to generate models for overall survival based on CPFs alone, CPFs with CT features, CPFs with PET features, and CPFs with CT and PET features. Linear predictors generated and categorized into 2 risk groups for which Kaplan-Meier survival cur ves were calculated. A log-rank test was performed to quantify the discrimination between the groups and calculated the Harrell's C-index to quantify the discriminatory power. A likelihood ratio test was performed to determine whether adding CT and/or PET features to CPFs improved model performance. Results: All 4 models significantly discriminated between the 2 risk groups. The discriminatory power was significantly increased when CPFs were combined with PET features (C-index 0.82; likelihood ratio test P<.01) or with both CT and PET features (0.83; P<.01) compared with CPFs alone (0.68). There was no significant improvement when CPFs were combined with CT features (0.68). Conclusion: Adding PET radiomics features to CPFs yielded a significant improvement in the prognostic value in locally advanced NSCLC; adding CT features did not. (C) 2021 Elsevier Inc. All rights reserved.

C1 [Moran, Angel; Daly, Megan E.; Yamamoto, Tokihiro] Univ Calif Davis, Sch Med, Dept Radiat Oncol, 4501 X St, Sacramento, CA 95817 USA.

[Wang, Yichuan] Univ Calif Davis, Dept Stat, Davis, CA 95616 USA.

[Dyer, Brandon A.] Univ Washington, Sch Med, Dept Radiat Oncol, Seattle, WA USA.

[Yip, Stephen S. F.] AIQ Solut Inc, Madison, WI USA.

RP Yamamoto, T (通讯作者)，Univ Calif Davis, Sch Med, Dept Radiat Oncol, 4501 X St, Sacramento, CA 95817 USA.

EM toyamamoto@ucdavis.edu

FU Radiological Society of North America (RSNA) Research Medical Student

Grant; National Institutes of Health (NIH)/National Cancer Institute

(NCI) [K12 CA138464]; NIH/National Center for Advancing Translational

Sciences [UL1 TR001860]

FX This study was supported in part by the Radiological Society of North

America (RSNA) Research Medical Student Grant (A.M.), National

Institutes of Health (NIH)/National Cancer Institute (NCI) grant K12

CA138464 (M.E.D), and NIH/National Center for Advancing Translational

Sciences grant UL1 TR001860.

CR Aerts HJWL, 2014, NAT COMMUN, V5, DOI 10.1038/ncomms5006

Auperin A, 2010, J CLIN ONCOL, V28, P2181, DOI 10.1200/JCO.2009.26.2543

Berghmans T, 2008, J THORAC ONCOL, V3, P6, DOI 10.1097/JTO.0b013e31815e6d6b

Berghmans Thierry, 2011, Ther Adv Med Oncol, V3, P127, DOI 10.1177/1758834011401951

Boellaard R, 2009, J NUCL MED, V50, p11S, DOI 10.2967/jnumed.108.057182

Bogowicz M, 2017, ACTA ONCOL, V56, P1531, DOI 10.1080/0284186X.2017.1346382

Bradley JD, 2015, LANCET ONCOL, V16, P187, DOI 10.1016/S1470-2045(14)71207-0

Brooks FJ, 2013, RADIAT ONCOL, V8, DOI 10.1186/1748-717X-8-294

Brooks FJ, 2011, RADIAT ONCOL, V6, DOI 10.1186/1748-717X-6-69

Chicklore S, 2013, EUR J NUCL MED MOL I, V40, P133, DOI 10.1007/s00259-012-2247-0

Collins GS, 2015, CIRCULATION, V131, P211, DOI [10.1161/CIRCULATIONAHA.114.014508, 10.7326/M14-0697, 10.1016/j.jclinepi.2014.11.010, 10.1186/s12916-014-0241-z, 10.1136/bmj.g7594, 10.1002/bjs.9736]

Cook GJR, 2013, J NUCL MED, V54, P19, DOI 10.2967/jnumed.112.107375

Dong XZ, 2016, PLOS ONE, V11, DOI 10.1371/journal.pone.0157836

Fang YHD, 2014, BIOMED RES INT, V2014, DOI 10.1155/2014/248505

Fried DV, 2016, RADIOLOGY, V278, P214, DOI 10.1148/radiol.2015142920

Fried DV, 2014, INT J RADIAT ONCOL, V90, P834, DOI 10.1016/j.ijrobp.2014.07.020

Galavis PE, 2010, ACTA ONCOL, V49, P1012, DOI 10.3109/0284186X.2010.498437

Ganeshan B, 2012, EUR RADIOL, V22, P796, DOI 10.1007/s00330-011-2319-8

Gould MK, 2003, ANN INTERN MED, V139, P879, DOI 10.7326/0003-4819-139-11-200311180-00013

Groheux D, 2015, EUR J NUCL MED MOL I, V42, P1682, DOI 10.1007/s00259-015-3110-x

Grove O, 2015, PLOS ONE, V10, DOI 10.1371/journal.pone.0118261

Hatt M, 2017, EUR J NUCL MED MOL I, V44, P151, DOI 10.1007/s00259-016-3427-0

Jeraj R, 2015, J NUCL MED, V56, P1752, DOI 10.2967/jnumed.114.141424

Kumar V, 2012, MAGN RESON IMAGING, V30, P1234, DOI 10.1016/j.mri.2012.06.010

Lambin P, 2012, EUR J CANCER, V48, P441, DOI 10.1016/j.ejca.2011.11.036

LECHEVALIER T, 1991, JNCI-J NATL CANCER I, V83, P417, DOI 10.1093/jnci/83.6.417

Lemarignier C, 2017, EUR J NUCL MED MOL I, V44, P1145, DOI 10.1007/s00259-017-3641-4

LOWE VJ, 1995, J NUCL MED, V36, P883

O'Rourke N, 2010, COCHRANE DB SYST REV, DOI 10.1002/14651858.CD002140.pub3

PEREZ CA, 1987, CANCER, V59, P1874, DOI 10.1002/1097-0142(19870601)59:11<1874::AID-CNCR2820591106>3.0.CO;2-Z

Siegel RL., 2020, CA-CANCER J CLIN, V70, P7, DOI [DOI 10.3322/caac.21551, 10.3322/caac.21590]

Silvestri GA, 2007, CHEST, V132, p178S, DOI 10.1378/chest.07-1360

Vaidya M, 2012, RADIOTHER ONCOL, V102, P239, DOI 10.1016/j.radonc.2011.10.014

Vallieres M, 2015, PHYS MED BIOL, V60, P5471, DOI 10.1088/0031-9155/60/14/5471

Wahl RL, 2009, J NUCL MED, V50, p122S, DOI 10.2967/jnumed.108.057307

Westerterp M, 2007, EUR J NUCL MED MOL I, V34, P392, DOI 10.1007/s00259-006-0224-1

Yip S, 2014, PLOS ONE, V9, DOI 10.1371/journal.pone.0115510

Yip SSF, 2016, PHYS MED BIOL, V61, pR150, DOI 10.1088/0031-9155/61/13/R150

ZASADNY KR, 1993, RADIOLOGY, V189, P847, DOI 10.1148/radiology.189.3.8234714

NR 39

TC 1

Z9 1

U1 0

U2 0

PU CIG MEDIA GROUP, LP

PI DALLAS

PA 3500 MAPLE AVENUE, STE 750, DALLAS, TX 75219-3931 USA

SN 1525-7304

EI 1938-0690

J9 CLIN LUNG CANCER

JI Clin. Lung Cancer

PD SEP

PY 2021

VL 22

IS 5

BP 461

EP 468

DI 10.1016/j.cllc.2021.03.015

EA SEP 2021

PG 8

WC Oncology

WE Science Citation Index Expanded (SCI-EXPANDED)

SC Oncology

GA XN1VD

UT WOS:000729299500010

DA 2022-08-24

ER

PT J

AU Luo, Y

El Naqa, I

McShan, DL

Ray, D

Lohse, I

Matuszak, MM

Owen, D

Jolly, S

Lawrence, TS

Kong, FM

Ten Haken, RK

AF Luo, Yi

El Naqa, Issam

McShan, Daniel L.

Ray, Dipankar

Lohse, Ines

Matuszak, Martha M.

Owen, Dawn

Jolly, Shruti

Lawrence, Theodore S.

Kong, Feng-Ming (Spring)

Ten Haken, Randall K.

TI Unraveling biophysical interactions of radiation pneumonitis in

non-small-cell lung cancer via Bayesian network analysis

SO RADIOTHERAPY AND ONCOLOGY

LA English

DT Article

DE Lung cancer; Radiation pneumonitis; Bayesian network analysis;

Biophysical interactions

ID BREAST-CANCER; RISK; THERAPY; RADIOTHERAPY; MICROARRAY; EXPRESSION;

PREDICTION; PARAMETERS; RESPONSES; TOXICITY

AB Background: In non-small-cell lung cancer radiotherapy, radiation pneumonitis >= grade 2 (RP2) depends on patients' dosimetric, clinical, biological and genomic characteristics.

Methods: We developed a Bayesian network (BN) approach to explore its potential for interpreting biophysical signaling pathways influencing RP2 from a heterogeneous dataset including single nucleotide polymorphisms, micro RNAs, cytokines, clinical data, and radiation treatment plans before and during the course of radiotherapy. Model building utilized 79 patients (21 with RP2) with complete data, and model testing used 50 additional patients with incomplete data. A developed large-scale Markov blanket approach selected relevant predictors. Resampling by k-fold cross-validation determined the optimal BN structure. Area under the receiver-operating characteristics curve (AUC) measured performance.

Results: Pre- and during-treatment BNs identified biophysical signaling pathways from the patients' relevant variables to RP2 risk. Internal cross-validation for the pre-BN yielded an AUC = 0.82 which improved to 0.87 by incorporating during treatment changes. In the testing dataset, the pre- and during AUCs were 0.78 and 0.82, respectively.

Conclusions: Our developed BN approach successfully handled a high number of heterogeneous variables in a small dataset, demonstrating potential for unraveling relevant biophysical features that could enhance prediction of RP2, although the current observations would require further independent validation. (C) 2017 Elsevier B.V. All rights reserved.

C1 [Luo, Yi; El Naqa, Issam; McShan, Daniel L.; Ray, Dipankar; Lohse, Ines; Matuszak, Martha M.; Owen, Dawn; Jolly, Shruti; Lawrence, Theodore S.; Ten Haken, Randall K.] Univ Michigan, Dept Radiat Oncol, UH B2C432,SPC 5010,1500 East Med Ctr Dr, Ann Arbor, MI 48109 USA.

[Kong, Feng-Ming (Spring)] Indiana Univ, Dept Radiat Oncol, Indianapolis, IN 46204 USA.

RP Ten Haken, RK (通讯作者)，Univ Michigan, Dept Radiat Oncol, UH B2C432,SPC 5010,1500 East Med Ctr Dr, Ann Arbor, MI 48109 USA.

EM rth@med.umich.edu

RI Kong, Feng-Ming/Y-2825-2019; Naqa, Issam El/T-3066-2019

OI Kong, Feng-Ming/0000-0003-2652-098X; Naqa, Issam El/0000-0001-6023-1132;

Luo, Yi/0000-0003-2519-5900; Ten Haken, Randall/0000-0003-1331-0297

FU National Institutes of Health [P01 CA059827, R01 CA142840]; NATIONAL

CANCER INSTITUTE [P01CA059827, R01CA142840] Funding Source: NIH RePORTER

FX This work was supported by the National Institutes of Health [grant

numbers P01 CA059827, R01 CA142840]. The authors wish to thank Paul

Stanton, Nan Bi, MD, PhD, and Weili Wang MD, PhD for their work in

processing the cytokine, miRNA and SNP data. This work was presented in

part at ICTR-PHE 2016, 15-19 February 2016, CICG, Geneva, Switzerland.

CR Agrawal S, 2014, SOUTH ASIAN J CANCER, V3, P13, DOI 10.4103/2278-330X.126503

Aliferis Constantin F., 2003, AMIA ANN S P

Baumann M, 2016, NAT REV CANCER, V16, P234, DOI 10.1038/nrc.2016.18

Bentzen SM, 2000, ACTA ONCOL, V39, P337, DOI 10.1080/028418600750013113

CARSON WE, 1994, J EXP MED, V180, P1395, DOI 10.1084/jem.180.4.1395

Claude L, 2004, RADIOTHER ONCOL, V71, P175, DOI 10.1016/j.radonc.2004.02.005

Corani G, 2012, INTEL SYST REF LIBR, V23, P49

Damaraju S, 2006, CLIN CANCER RES, V12, P2545, DOI 10.1158/1078-0432.CCR-05-2703

Ebert N, 2015, RADIOTHER ONCOL, V117, P1, DOI 10.1016/j.radonc.2015.09.001

El Naqa I, 2014, WIRES DATA MIN KNOWL, V4, P327, DOI 10.1002/widm.1131

Flanders KC, 2003, AM J PATHOL, V163, P2247, DOI 10.1016/S0002-9440(10)63582-1

Friedman N, 1999, UNCERTAINTY IN ARTIFICIAL INTELLIGENCE, PROCEEDINGS, P196

Fukuyama T, 2007, CANCER SCI, V98, P1048, DOI 10.1111/j.1349-7006.2007.00507.x

Gadewadikar J., 2010, AFRICAN J MATH COMPU, V3, P225

Gevaert O, 2006, BIOINFORMATICS, V22, pE184, DOI 10.1093/bioinformatics/btl230

Guo LL, 2016, TUMOR BIOL, V37, P115, DOI 10.1007/s13277-015-4374-2

Kong FM, 2015, SEMIN RADIAT ONCOL, V25, P100, DOI 10.1016/j.semradonc.2014.12.003

Kouloulias V, 2013, ASIAN PAC J CANCER P, V14, P2717, DOI 10.7314/APJCP.2013.14.5.2717

Kwa SLS, 1998, RADIOTHER ONCOL, V48, P61, DOI 10.1016/S0167-8140(98)00020-6

Lee S, 2015, MED PHYS, V42

LOKKETANGEN A, 1995, AI COMMUN, V8, P78

Metz Charles E, 2006, J Am Coll Radiol, V3, P413, DOI 10.1016/j.jacr.2006.02.021

Park IK, 2007, MOL IMMUNOL, V44, P3283, DOI 10.1016/j.molimm.2007.02.024

Parker BJ, 2007, BMC BIOINFORMATICS, V8, DOI 10.1186/1471-2105-8-326

Pearl J., 1988, PROBABILISTIC REASON, V58, P721

Pellet JP, 2008, J MACH LEARN RES, V9, P1295

Perlich C., 2010, ACM SIGKDD EXPLOR NE, V12, P11

Provatopoulou X, 2008, ANTICANCER RES, V28, P2421

Rancati T, 2003, RADIOTHER ONCOL, V67, P275, DOI 10.1016/S0167-8140(03)00119-1

RODEMANN HP, 1995, RADIOTHER ONCOL, V35, P83, DOI 10.1016/0167-8140(95)01540-W

Rodrigues G, 2004, RADIOTHER ONCOL, V71, P127, DOI 10.1016/j.radonc.2004.02.015

Schaue D, 2012, RADIAT RES, V178, P505, DOI 10.1667/RR3031.1

Schiller TW, 2010, NEUROCOMPUTING, V73, P1861, DOI 10.1016/j.neucom.2009.09.023

Shi AH, 2010, RADIAT ONCOL, V5, DOI 10.1186/1748-717X-5-35

Slattery ML, 2011, CANCER EPIDEM BIOMAR, V20, P57, DOI 10.1158/1055-9965.EPI-10-0843

Waldmann TA, 2006, NAT REV IMMUNOL, V6, P595, DOI 10.1038/nri1901

Weng HL, 2007, J HEPATOL, V46, P295, DOI 10.1016/j.jhep.2006.09.014

NR 37

TC 38

Z9 39

U1 1

U2 13

PU ELSEVIER IRELAND LTD

PI CLARE

PA ELSEVIER HOUSE, BROOKVALE PLAZA, EAST PARK SHANNON, CO, CLARE, 00000,

IRELAND

SN 0167-8140

J9 RADIOTHER ONCOL

JI Radiother. Oncol.

PD APR

PY 2017

VL 123

IS 1

BP 85

EP 92

DI 10.1016/j.radonc.2017.02.004

PG 8

WC Oncology; Radiology, Nuclear Medicine & Medical Imaging

WE Science Citation Index Expanded (SCI-EXPANDED)

SC Oncology; Radiology, Nuclear Medicine & Medical Imaging

GA EU0RR

UT WOS:000400719500013

PM 28237401

OA Green Accepted

DA 2022-08-24

ER

PT J

AU Cui, SN

Luo, Y

Tseng, HH

Ten Haken, RK

El Naga, I

AF Cui, Sunan

Luo, Yi

Tseng, Huan-Hsin

Ten Haken, Randall K.

El Naga, Issam

TI Combining handcrafted features with latent variables in machine learning

for prediction of radiation-induced lung damage

SO MEDICAL PHYSICS

LA English

DT Article

DE deep neural networks; feature selection; machine learning; radiotherapy

outcome modeling

ID RADIOTHERAPY OUTCOMES; FEATURE-SELECTION; NEURAL-NETWORK; DOSE-VOLUME;

PNEUMONITIS; CANCER; MODEL; IRRADIATION

AB Purpose There has been burgeoning interest in applying machine learning methods for predicting radiotherapy outcomes. However, the imbalanced ratio of a large number of variables to a limited sample size in radiation oncology constitutes a major challenge. Therefore, dimensionality reduction methods can be a key to success. The study investigates and contrasts the application of traditional machine learning methods and deep learning approaches for outcome modeling in radiotherapy. In particular, new joint architectures based on variational autoencoder (VAE) for dimensionality reduction are presented and their application is demonstrated for the prediction of lung radiation pneumonitis (RP) from a large-scale heterogeneous dataset. Methods A large-scale heterogeneous dataset containing a pool of 230 variables including clinical factors (e.g., dose, KPS, stage) and biomarkers (e.g., single nucleotide polymorphisms (SNPs), cytokines, and micro-RNAs) in a population of 106 nonsmall cell lung cancer (NSCLC) patients who received radiotherapy was used for modeling RP. Twenty-two patients had grade 2 or higher RP. Four methods were investigated, including feature selection (case A) and feature extraction (case B) with traditional machine learning methods, a VAE-MLP joint architecture (case C) with deep learning and lastly, the combination of feature selection and joint architecture (case D). For feature selection, Random forest (RF), Support Vector Machine (SVM), and multilayer perceptron (MLP) were implemented to select relevant features. Specifically, each method was run for multiple times to rank features within several cross-validated (CV) resampled sets. A collection of ranking lists were then aggregated by top 5% and Kemeny graph methods to identify the final ranking for prediction. A synthetic minority oversampling technique was applied to correct for class imbalance during this process. For deep learning, a VAE-MLP joint architecture where a VAE aimed for dimensionality reduction and an MLP aimed for classification was developed. In this architecture, reconstruction loss and prediction loss were combined into a single loss function to realize simultaneous training and weights were assigned to different classes to mitigate class imbalance. To evaluate the prediction performance and conduct comparisons, the area under receiver operating characteristic curves (AUCs) were performed for nested CVs for both handcrafted feature selections and the deep learning approach. The significance of differences in AUCs was assessed using the DeLong test of U-statistics. Results An MLP-based method using weight pruning (WP) feature selection yielded the best performance among the different hand-crafted feature selection methods (case A), reaching an AUC of 0.804 (95% CI: 0.761-0.823) with 29 top features. A VAE-MLP joint architecture (case C) achieved a comparable but slightly lower AUC of 0.781 (95% CI: 0.737-0.808) with the size of latent dimension being 2. The combination of handcrafted features (case A) and latent representation (case D) achieved a significant AUC improvement of 0.831 (95% CI: 0.805-0.863) with 22 features (P-value = 0.000642 compared with handcrafted features only (Case A) and P-value = 0.000453 compared to VAE alone (Case C)) with an MLP classifier.

Conclusion The potential for combination of traditional machine learning methods and deep learning VAE techniques has been demonstrated for dealing with limited datasets in modeling radiotherapy toxicities. Specifically, latent variables from a VAE-MLP joint architecture are able to complement handcrafted features for the prediction of RP and improve prediction over either method alone.(c) 2019 American Association of Physicists in Medicine

C1 [Cui, Sunan] Univ Michigan, Appl Phys Program, Ann Arbor, MI 48109 USA.

[Luo, Yi; Tseng, Huan-Hsin; Ten Haken, Randall K.; El Naga, Issam] Univ Michigan, Dept Radiat Oncol, Ann Arbor, MI 48109 USA.

RP Cui, SN (通讯作者)，Univ Michigan, Appl Phys Program, Ann Arbor, MI 48109 USA.

EM sunan@umich.edu

RI cui, sunan/AAA-3286-2020; Naqa, Issam El/T-3066-2019

OI cui, sunan/0000-0002-8846-9449; Naqa, Issam El/0000-0001-6023-1132

FU National Institutes of Health (NIH) [P01-CA059827, R37-CA222215];

Rackham Predoctoral fellowship; NATIONAL CANCER INSTITUTE [P01CA059827,

R37CA222215] Funding Source: NIH RePORTER

FX This work was partly supported by National Institutes of Health (NIH)

(P01-CA059827 and R37-CA222215) and Rackham Predoctoral fellowship. The

authors have no conflicts to disclose.

CR Baldi P, 2012, P ICML WORKSH UNS TR, P37

Bengio Y, 2013, IEEE T PATTERN ANAL, V35, P1798, DOI 10.1109/TPAMI.2013.50

Bentzen SM, 2000, ACTA ONCOL, V39, P337, DOI 10.1080/028418600750013113

Bentzen SM, 2010, INT J RADIAT ONCOL, V76, pS3, DOI 10.1016/j.ijrobp.2009.09.040

BREIMAN L, 2001, MACH LEARN, V0045

Chawla NV, 2002, J ARTIF INTELL RES, V16, P321, DOI 10.1613/jair.953

Chen SF, 2007, MED PHYS, V34, P3420, DOI 10.1118/1.2759601

Chen S, 2007, MED PHYS, V34, P3808, DOI 10.1118/1.2776669

Cho K., 2014, ARXIV14061078, DOI DOI 10.3115/V1/D14-1179

Chollet F., 2015, KERAS

Collins GS, 2015, CIRCULATION, V131, P211, DOI [10.1161/CIRCULATIONAHA.114.014508, 10.7326/M14-0697, 10.1016/j.jclinepi.2014.11.010, 10.1186/s12916-014-0241-z, 10.1136/bmj.g7594, 10.1002/bjs.9736]

Conitzer V., 2006, AAAI, V6, P620

Damaraju S, 2006, CLIN CANCER RES, V12, P2545, DOI 10.1158/1078-0432.CCR-05-2703

DELONG ER, 1988, BIOMETRICS, V44, P837, DOI 10.2307/2531595

Demler OV, 2012, STAT MED, V31, P2577, DOI 10.1002/sim.5328

El Naqa I, 2006, PHYS MED BIOL, V51, P5719, DOI 10.1088/0031-9155/51/22/001

El Naqa I, 2006, INT J RADIAT ONCOL, V64, P1275, DOI 10.1016/j.ijrobp.2005.11.022

El Naqa I., 2018, GUIDE OUTCOME MODELI

El Naqa I, 2009, PHYS MED BIOL, V54, pS9, DOI 10.1088/0031-9155/54/18/S02

Fukuyama T, 2007, CANCER SCI, V98, P1048, DOI 10.1111/j.1349-7006.2007.00507.x

Goodfellow I, 2016, ADAPT COMPUT MACH LE, P1

Guo LL, 2016, TUMOR BIOL, V37, P115, DOI 10.1007/s13277-015-4374-2

Guyon Isabelle, 2003, J MACH LEARN RES, V3, P1157, DOI DOI 10.1162/153244303322753616

Kingma D, 2014, ARXIV

Kingma DP, 2013, ARXIV PREPRINT ARXIV

Kong FM, 2007, SEMIN RADIAT ONCOL, V17, P108, DOI 10.1016/j.semradonc.2006.11.007

Kouloulias V, 2013, ASIAN PAC J CANCER P, V14, P2717, DOI 10.7314/APJCP.2013.14.5.2717

Krizhevsky A., 2012, ADV NEURAL INFORM PR, V25, DOI DOI 10.1145/3065386

KUTCHER GJ, 1989, INT J RADIAT ONCOL, V16, P1623, DOI 10.1016/0360-3016(89)90972-3

LeCun Y, 2015, NATURE, V521, P436, DOI 10.1038/nature14539

Lee S, 2015, MED PHYS, V42, P2421, DOI 10.1118/1.4915284

Lin SL, 2010, WILEY INTERDISCIP RE, V2, P555, DOI 10.1002/wics.111

Luo Y, 2017, RADIOTHER ONCOL, V123, P85, DOI 10.1016/j.radonc.2017.02.004

Lyman JT, 1985, TOLERANCE DOSES TREA, DOI [10.2172/6934260, DOI 10.2172/6934260]

Marks LB, 2010, INT J RADIAT ONCOL, V76, pS10, DOI 10.1016/j.ijrobp.2009.07.1754

Mitchell Stuart, 2011, PULP LINEAR PROGRAMM

NIEMIERKO A, 1993, INT J RADIAT ONCOL, V25, P135, DOI 10.1016/0360-3016(93)90156-P

Pedregosa F., 2011, J MACH LEARN RES, V12, P2825

Saeys Y, 2007, BIOINFORMATICS, V23, P2507, DOI 10.1093/bioinformatics/btm344

Silver D, 2016, NATURE, V529, P484, DOI 10.1038/nature16961

Slattery ML, 2011, CANCER EPIDEM BIOMAR, V20, P57, DOI 10.1158/1055-9965.EPI-10-0843

Stavrev P, 2005, INT J RADIAT BIOL, V81, P77, DOI 10.1080/09553000400027910

Su M, 2005, MED PHYS, V32, P318, DOI 10.1118/1.1835611

Tong S, 2002, J MACH LEARN RES, V2, P45, DOI 10.1162/153244302760185243

Verikas A, 2002, PATTERN RECOGN LETT, V23, P1323, DOI 10.1016/S0167-8655(02)00081-8

Yacoub M., 1997, INTELLIGENT ENG SYST, V7, P527

Yang JB, 2009, IEEE T NEURAL NETWOR, V20, P1911, DOI 10.1109/TNN.2009.2032543

NR 47

TC 12

Z9 13

U1 5

U2 24

PU WILEY

PI HOBOKEN

PA 111 RIVER ST, HOBOKEN 07030-5774, NJ USA

SN 0094-2405

EI 2473-4209

J9 MED PHYS

JI Med. Phys.

PD MAY

PY 2019

VL 46

IS 5

BP 2497

EP 2511

DI 10.1002/mp.13497

PG 15

WC Radiology, Nuclear Medicine & Medical Imaging

WE Science Citation Index Expanded (SCI-EXPANDED)

SC Radiology, Nuclear Medicine & Medical Imaging

GA HX7AV

UT WOS:000467556800054

PM 30891794

OA Green Published, Green Accepted

DA 2022-08-24

ER

PT J

AU Kim, MS

Park, HY

Kho, BG

Park, CK

Oh, IJ

Kim, YC

Kim, S

Yun, JS

Song, SY

Na, KJ

Jeong, JU

Yoon, MS

Ahn, SJ

Yoo, SW

Kang, SR

Kwon, SY

Bom, HS

Jang, WY

Kim, IY

Lee, JE

Jeong, WG

Kim, YH

Lee, T

Choi, YD

AF Kim, Min-Seok

Park, Ha-Young

Kho, Bo-Gun

Park, Cheol-Kyu

Oh, In-Jae

Kim, Young-Chul

Kim, Seok

Yun, Ju-Sik

Song, Sang-Yun

Na, Kook-Joo

Jeong, Jae-Uk

Yoon, Mee Sun

Ahn, Sung-Ja

Yoo, Su Woong

Kang, Sae-Ryung

Kwon, Seong Young

Bom, Hee-Seung

Jang, Woo-Youl

Kim, In-Young

Lee, Jong-Eun

Jeong, Won-Gi

Kim, Yun-Hyeon

Lee, Taebum

Choi, Yoo-Duk

TI Artificial intelligence and lung cancer treatment decision: agreement

with recommendation of multidisciplinary tumor board

SO TRANSLATIONAL LUNG CANCER RESEARCH

LA English

DT Article

DE Watson for Oncology (WFO); multidisciplinary tumor board; lung cancer

ID WATSON; EPIDEMIOLOGY; ONCOLOGY

AB Background: IBM Watson for Oncology (WFO) is a cognitive computing system helping physicians quickly identify key information in a patient's medical record, surface relevant evidence, and explore treatment options. This study assessed the possibility of using WFO for clinical treatment in lung cancer patients.

Methods: We evaluated the level of agreement between WFO and multidisciplinary team (MDT) for lung cancer. From January to December 2018, newly diagnosed lung cancer cases in Chonnam National University Hwasun Hospital were retrospectively examined using WFO version 18.4 according to four treatment categories (surgery, radiotherapy, chemoradiotherapy, and palliative care). Treatment recommendations were considered concordant if the MDT recommendations were designated 'recommended' by WFO. Concordance between MDT and WFO was analyzed by Cohen's kappa value.

Results: In total, 405 (male 340, female 65) cases with different histology (adenocarcinoma 157, squamous cell carcinoma 132, small cell carcinoma 94, others 22 cases) were enrolled. Concordance between MDT and WFO occurred in 92.4% (k=0.881, P<0.001) of all cases, and concordance differed according to clinical stages. The strength of agreement was very good in stage IV non-small cell lung carcinoma (NSCLC) (100%, k=1.000) and extensive disease small cell lung carcinoma (SCLC) (100%, k=1.000). In stage I NSCLC, the agreement strength was good (92.4%, k=0.855). The concordance was moderate in stage III NSCLC (80.8%, k=0.622) and relatively low in stage II NSCLC (83.3%, k=0.556) and limited disease SCLC (84.6%, k=0.435). There were discordant cases in surgery (7/57, 12.3%), radiotherapy (2/12, 16.7%), and chemoradiotherapy (15/129, 11.6%), but no discordance in metastatic disease patients.

Conclusions: Treatment recommendations made by WFO and MDT were highly concordant for lung cancer cases especially in metastatic stage. However, WFO was just an assisting tool in stage I-III NSCLC and limited disease SCLC; so, patient-doctor relationship and shared decision making may be more important in this stage.

C1 [Kim, Min-Seok; Park, Ha-Young; Kho, Bo-Gun; Park, Cheol-Kyu; Oh, In-Jae; Kim, Young-Chul; Kim, Seok; Yun, Ju-Sik; Song, Sang-Yun; Na, Kook-Joo; Jeong, Jae-Uk; Yoon, Mee Sun; Ahn, Sung-Ja; Yoo, Su Woong; Kang, Sae-Ryung; Kwon, Seong Young; Bom, Hee-Seung; Jang, Woo-Youl; Kim, In-Young; Jeong, Won-Gi; Choi, Yoo-Duk] Chonnam Natl Univ, Hwasun Hosp, Lung & Esophageal Canc Clin, Hwasun, South Korea.

[Kim, Min-Seok; Park, Ha-Young; Kho, Bo-Gun; Park, Cheol-Kyu; Oh, In-Jae; Kim, Young-Chul] Chonnam Natl Univ, Med Sch, Dept Internal Med, Gwangju, South Korea.

[Kim, Seok; Yun, Ju-Sik; Song, Sang-Yun; Na, Kook-Joo] Chonnam Natl Univ, Med Sch, Dept Thorac Surg, Gwangju, South Korea.

[Jeong, Jae-Uk; Yoon, Mee Sun; Ahn, Sung-Ja] Chonnam Natl Univ, Med Sch, Dept Radiat Oncol, Gwangju, South Korea.

[Yoo, Su Woong; Kang, Sae-Ryung; Kwon, Seong Young; Bom, Hee-Seung] Chonnam Natl Univ, Med Sch, Dept Nucl Med, Gwangju, South Korea.

[Jang, Woo-Youl; Kim, In-Young] Chonnam Natl Univ, Med Sch, Dept Neurosurg, Gwangju, South Korea.

[Lee, Jong-Eun; Jeong, Won-Gi; Kim, Yun-Hyeon] Chonnam Natl Univ, Med Sch, Dept Radiol, Gwangju, South Korea.

[Lee, Taebum; Choi, Yoo-Duk] Chonnam Natl Univ, Med Sch, Dept Pathol, Gwangju, South Korea.

RP Oh, IJ (通讯作者)，Chonnam Natl Univ, Hwasun Hosp, Dept Internal Med, 322 Seoyang Ro, Hwasun 58128, Jeonnam, South Korea.

EM droij@jnu.ac.kr

RI Kwon, Seong Young/AAU-3101-2021; Oh, In-Jae/AAG-5919-2020

OI Kwon, Seong Young/0000-0002-2832-896X; Oh, In-Jae/0000-0003-4837-1321

FU Chonnam National University Hwasun Hospital Institute for Biomedical

Science [HCRI19025]

FX This study was supported by grants (HCRI19025) from the Chonnam National

University Hwasun Hospital Institute for Biomedical Science.

CR Adamson AS, 2019, NEW ENGL J MED, V381, P2285, DOI 10.1056/NEJMp1907407

Ahmed MN, 2017, IEEE PULSE, V8, P4, DOI 10.1109/MPUL.2017.2678098

[Anonymous], 2017, LANCET, V390, P2739, DOI 10.1016/S0140-6736(17)31540-4

Ardila D, 2019, NAT MED, V25, P954, DOI 10.1038/s41591-019-0447-x

Chen Y, 2016, CLIN THER, V38, P688, DOI 10.1016/j.clinthera.2015.12.001

Hamet P, 2017, METABOLISM, V69, pS36, DOI 10.1016/j.metabol.2017.01.011

Han HH, 2017, MICROSURG, V37, P49, DOI 10.1002/micr.22463

Kweon Sun-Seog, 2018, Chonnam Med J, V54, P90, DOI 10.4068/cmj.2018.54.2.90

Liu CY, 2018, J MED INTERNET RES, V20, DOI 10.2196/11087

Makedon F, 2006, ONCOL REP, V15, P971, DOI 10.3892/or.15.4.971

Malin JL, 2013, J ONCOL PRACT, V9, P155, DOI 10.1200/JOP.2013.001021

Oh IJ, 2017, RADIAT ONCOL J, V35, P16, DOI 10.3857/roj.2017.00108

OKEN MM, 1982, AM J CLIN ONCOL-CANC, V5, P649, DOI 10.1097/00000421-198212000-00014

Park JY, 2016, TUBERC RESPIR DIS, V79, P58, DOI 10.4046/trd.2016.79.2.58

SCHMIDT C, 2017, JNCI J NATL CANCER I, V109, DOI DOI 10.1093/jnci/djx113

Shin A, 2017, CANCER RES TREAT, V49, P616, DOI 10.4143/crt.2016.178

Siegel RL, 2022, CA-CANCER J CLIN, V72, P7, DOI [10.3322/caac.21332, 10.3322/caac.21708, 10.3322/caac.21551]

Somashekhar SP, 2018, ANN ONCOL, V29, P418, DOI 10.1093/annonc/mdx781

Xu YW, 2019, CLIN CANCER RES, V25, P3266, DOI 10.1158/1078-0432.CCR-18-2495

Zhou N, 2019, ONCOLOGIST, V24, P812, DOI 10.1634/theoncologist.2018-0255

NR 20

TC 13

Z9 15

U1 2

U2 7

PU AME PUBL CO

PI SHATIN

PA FLAT-RM C 16F, KINGS WING PLAZA 1, NO 3 KWAN ST, SHATIN, HONG KONG

00000, PEOPLES R CHINA

SN 2218-6751

EI 2226-4477

J9 TRANSL LUNG CANCER R

JI Transl. Lung Cancer Res.

PD JUN

PY 2020

VL 9

IS 3

BP 507

EP 514

DI 10.21037/tlcr.2020.04.11

PG 8

WC Oncology; Respiratory System

WE Science Citation Index Expanded (SCI-EXPANDED)

SC Oncology; Respiratory System

GA MJ1WM

UT WOS:000547884300012

PM 32676314

OA Green Published, gold

DA 2022-08-24

ER

PT J

AU Coroller, TP

Agrawal, V

Narayan, V

Hou, Y

Grossmann, P

Lee, SW

Mak, RH

Aerts, HJWL

AF Coroller, Thibaud P.

Agrawal, Vishesh

Narayan, Vivek

Hou, Ying

Grossmann, Patrick

Lee, Stephanie W.

Mak, Raymond H.

Aerts, Hugo J. W. L.

TI Radiomic phenotype features predict pathological response in non-small

cell lung cancer

SO RADIOTHERAPY AND ONCOLOGY

LA English

DT Article

DE Radiomics; Pathological response; NSCLC; Biomarkers; Quantitative

imaging

ID GROSS TUMOR VOLUME; NEOADJUVANT THERAPY; PROGNOSTIC-FACTOR; FDG-PET;

SURVIVAL; CHEMOTHERAPY; TEXTURE; RADIOTHERAPY; RESECTION; MODELS

AB Background and purpose: Radiomics can quantify tumor phenotype characteristics non-invasively by applying advanced imaging feature algorithms. In this study we assessed if pre-treatment radiomics data are able to predict pathological response after neoadjuvant chemoradiation in patients with locally advanced non-small cell lung cancer (NSCLC).

Materials and Methods: 127 NSCLC patients were included in this study. Fifteen radiomic features selected based on stability and variance were evaluated for its power to predict pathological response. Predictive power was' evaluated using area under the curve (AUC). Conventional imaging features (tumor volume and diameter) were used for comparison.

Results: Seven features were predictive for pathologic gross residual disease (AUC > 0.6, p-value < 0.05), and one for pathologic complete response (AUC = 0.63, p-value = 0.01). No conventional imaging features were predictive (range AUC = 0.51-0.59, p-value > 0.05). Tumors that did not respond well to neoadjuvant chemoradiation were more likely to present a rounder shape (spherical disproportionality, AUC = 0.63, p-value = 0.009) and heterogeneous texture (LoG 5 mm 3D - GLCM entropy, AUC = 0.61, p-value = 0.03).

Conclusion: We identified predictive radiomic features for pathological response, although no conventional features were significantly predictive. This study demonstrates that radiomics can provide valuable clinical information, and performed better than conventional imaging features. (C) 2016 Elsevier Ireland Ltd. All rights reserved.

C1 [Coroller, Thibaud P.; Agrawal, Vishesh; Narayan, Vivek; Hou, Ying; Grossmann, Patrick; Lee, Stephanie W.; Mak, Raymond H.; Aerts, Hugo J. W. L.] Harvard Med Sch, Brigham & Womens Hosp, Dana Farber Canc Inst, Dept Radiat Oncol, Boston, MA USA.

[Aerts, Hugo J. W. L.] Harvard Med Sch, Brigham & Womens Hosp, Dana Farber Canc Inst, Dept Radiol, Boston, MA USA.

RP Coroller, TP (通讯作者)，Harvard Med Sch, Brigham & Womens Hosp, Dana Farber Canc Inst, 450 Brookline Ave,JF518, Boston, MA 02115 USA.

EM tcoroller@lroc.harvard.edu

RI Aerts, Hugo/P-6350-2015; Aerts, Hugo/ABF-2821-2020

OI Aerts, Hugo/0000-0002-2122-2003; Aerts, Hugo/0000-0002-2122-2003; Mak,

Raymond/0000-0002-8754-0565; Coroller, Thibaud/0000-0001-7662-8724

FU National Institutes of Health (NIH-USA) [U24CA194354, U01CA190234]; Kaye

Scholar Award; Brigham and Women's Hospital Department of Radiation

Oncology Clinical Translational Grant; NATIONAL CANCER INSTITUTE

[U01CA190234, U24CA194354] Funding Source: NIH RePORTER

FX Authors acknowledge financial support from the National Institutes of

Health (NIH-USA U24CA194354, and NIH-USA U01CA190234). This project was

partially funded by the Kaye Scholar Award and the Brigham and Women's

Hospital Department of Radiation Oncology Clinical Translational Grant.

CR Aerts M, 2014, NAT COMMUN, V5, DOI 10.1038/ncomms4789

Albain KS, 2009, LANCET, V374, P379, DOI 10.1016/S0140-6736(09)60737-6

Alexander BM, 2011, INT J RADIAT ONCOL, V79, P1381, DOI 10.1016/j.ijrobp.2009.12.060

Bradley JD, 2002, INT J RADIAT ONCOL, V52, P49, DOI 10.1016/S0360-3016(01)01772-2

Cerfolio RJ, 2004, ANN THORAC SURG, V78, P1903, DOI 10.1016/j.athoracsur.2004.06.102

Core Team R, 2013, R LANG ENV STAT COMP

Coroller TP, 2015, RADIOTHER ONCOL, V114, P345, DOI 10.1016/j.radonc.2015.02.015

Edge SB, 2010, ANN SURG ONCOL, V17, P1471, DOI 10.1245/s10434-010-0985-4

Eisenhauer EA, 2009, EUR J CANCER, V45, P228, DOI 10.1016/j.ejca.2008.10.026

Fox MJ, 2015, J MAGN RESON IMAGING

Gentleman RC, 2004, GENOME BIOL, V5, DOI 10.1186/gb-2004-5-10-r80

Gillies RJ, 2015, RADIOLOGY, DOI DOI 10.1148/RADIOL.2015151169

Haibe-Kains B, 2008, BIOINFORMATICS, V24, P2200, DOI 10.1093/bioinformatics/btn374

Hellmann MD, 2014, LANCET ONCOL, V15, pE42, DOI 10.1016/S1470-2045(13)70334-6

Isobe K, 2012, ASIA-PAC J CLIN ONCO, V8, P260, DOI 10.1111/j.1743-7563.2012.01529.x

Kuhn M, 2008, J STAT SOFTW, V28, P1, DOI 10.18637/jss.v028.i05

Kumar V, 2012, MAGN RESON IMAGING, V30, P1234, DOI 10.1016/j.mri.2012.06.010

Lambin P, 2012, EUR J CANCER, V48, P441, DOI 10.1016/j.ejca.2011.11.036

Lee J, 2016, AM J NEURORADIOL, V37, P37, DOI 10.3174/ajnr.A4534

Leijenaar RTH, 2015, ACTA ONCOL, V54, P1423, DOI 10.3109/0284186X.2015.1061214

Leijenaar RTH, 2013, ACTA ONCOL, V52, P1391, DOI 10.3109/0284186X.2013.812798

Lubner MG, 2015, ABDOM IMAGING, V40, P2331, DOI 10.1007/s00261-015-0438-4

Mouillet G, 2012, J THORAC ONCOL, V7, P841, DOI 10.1097/JTO.0b013e31824c7d92

National Comprehensive Cancer Network, 2015, NCCN GUID NONSM CELL

Parmar C, 2015, SCI REP-UK, V5, DOI 10.1038/srep13087

Parmar C, 2015, SCI REP-UK, V5, DOI 10.1038/srep11044

Parmar C, 2014, PLOS ONE, V9, DOI 10.1371/journal.pone.0102107

Pickles MD, 2016, INVEST RADIOL, V51, P177, DOI 10.1097/RLI.0000000000000222

Pieper S, 2004, IEEE INT S BIOMED IM, V26, P632, DOI DOI 10.1109/ISBI.2004.1398617

Poettgen C, 2007, ONCOLOGY-BASEL, V73, P316, DOI 10.1159/000134474

Ravanelli M, 2013, EUR RADIOL, V23, P3450, DOI 10.1007/s00330-013-2965-0

Schroder MS, 2011, BIOINFORMATICS, V27, P3206, DOI 10.1093/bioinformatics/btr511

Siegel R, 2014, CA-CANCER J CLIN, V64, P9, DOI [10.3322/caac.21208, 10.3322/caac.21254, 10.1001/jamaoto.2014.2530, 10.1136/bmj.g1502]

Stinchcombe TE, 2006, LUNG CANCER, V52, P67, DOI 10.1016/j.lungcan.2005.11.008

van Meerbeeck JP, 2007, JNCI-J NATL CANCER I, V99, P442, DOI 10.1093/jnci/djk093

Velazquez ER, 2012, RADIOTHER ONCOL, V105, P167, DOI 10.1016/j.radonc.2012.09.023

Werner-Wasik M, 2001, INT J RADIAT ONCOL, V51, P56, DOI 10.1016/S0360-3016(01)01615-7

Zhao BS, 2009, RADIOLOGY, V252, P263, DOI 10.1148/radiol.2522081593

NR 38

TC 193

Z9 209

U1 5

U2 64

PU ELSEVIER IRELAND LTD

PI CLARE

PA ELSEVIER HOUSE, BROOKVALE PLAZA, EAST PARK SHANNON, CO, CLARE, 00000,

IRELAND

SN 0167-8140

EI 1879-0887

J9 RADIOTHER ONCOL

JI Radiother. Oncol.

PD JUN

PY 2016

VL 119

IS 3

BP 480

EP 486

DI 10.1016/j.radonc.2016.04.004

PG 7

WC Oncology; Radiology, Nuclear Medicine & Medical Imaging

WE Science Citation Index Expanded (SCI-EXPANDED)

SC Oncology; Radiology, Nuclear Medicine & Medical Imaging

GA DR7JH

UT WOS:000380075400017

PM 27085484

OA Green Accepted

DA 2022-08-24

ER

PT J

AU Bai, X

Shan, GP

Chen, M

Wang, BB

AF Bai, Xue

Shan, Guoping

Chen, Ming

Wang, Binbing

TI Approach and assessment of automated stereotactic radiotherapy planning

for early stage non-small-cell lung cancer

SO BIOMEDICAL ENGINEERING ONLINE

LA English

DT Article

DE Machine learning; Non-small-cell lung cancer radiotherapy planning;

Stereotactic body radiotherapy; Machine learning

ID MODULATED RADIATION-THERAPY; BEAM ORIENTATION OPTIMIZATION;

MULTIOBJECTIVE OPTIMIZATION; IMRT; PROSTATE; QUALITY; ARC; ALGORITHM;

SYSTEM; GENERATION

AB Background Intensity-modulated radiotherapy (IMRT) and volumetric-modulated arc therapy (VMAT) are standard physical technologies of stereotactic body radiotherapy (SBRT) that are used for patients with non-small-cell lung cancer (NSCLC). The treatment plan quality depends on the experience of the planner and is limited by planning time. An automated planning process can save time and ensure a high-quality plan. This study aimed to introduce and demonstrate an automated planning procedure for SBRT for patients with NSCLC based on machine-learning algorithms. The automated planning was conducted in two steps: (1) determining patient-specific optimized beam orientations; (2) calculating the organs at risk (OAR) dose achievable for a given patient and setting these dosimetric parameters as optimization objectives. A model was developed using data of historical expertise plans based on support vector regression. The study cohort comprised patients with NSCLC who were treated using SBRT. A training cohort (N = 125) was used to calculate the beam orientations and dosimetric parameters for the lung as functions of the geometrical feature of each case. These plan-geometry relationships were used in a validation cohort (N = 30) to automatically establish the SBRT plan. The automatically generated plans were compared with clinical plans established by an experienced planner. Results All 30 automated plans (100%) fulfilled the dose criteria for OARs and planning target volume (PTV) coverage, and were deemed acceptable according to evaluation by experienced radiation oncologists. An automated plan increased the mean maximum dose for ribs (31.6 +/- 19.9 Gy vs. 36.6 +/- 18.1 Gy, P < 0.05). The minimum, maximum, and mean dose; homogeneity index; conformation index to PTV; doses to other organs; and the total monitor units showed no significant differences between manual plans established by experts and automated plans (P > 0.05). The hands-on planning time was reduced from 40-60 min to 10-15 min. Conclusion An automated planning method using machine learning was proposed for NSCLC SBRT. Validation results showed that the proposed method decreased planning time without compromising plan quality. Plans generated by this method were acceptable for clinical use.

C1 [Bai, Xue; Shan, Guoping; Chen, Ming; Wang, Binbing] Zhejiang Canc Hosp, Zhejiang Key Lab Radiat Oncol, Dept Radiat Phys, Hangzhou 310022, Zhejiang, Peoples R China.

RP Wang, BB (通讯作者)，Zhejiang Canc Hosp, Zhejiang Key Lab Radiat Oncol, Dept Radiat Phys, Hangzhou 310022, Zhejiang, Peoples R China.

EM wangbb@zjcc.org.cn

FU National Key Research and Development Program of China [2017YFC0113201];

Zhejiang Provincial Natural Science Foundation of China [LSY19H180002];

Medical Science and Technology Program of Zhejiang Province [2017PY013,

2018PY005]; Key Laboratory of Radiation Physics and Technology (Sichuan

University), Ministry of Education [2018SCURPT09]

FX This study was supported in part by the National Key Research and

Development Program of China (2017YFC0113201), the Zhejiang Provincial

Natural Science Foundation of China (LSY19H180002), the Medical Science

and Technology Program of Zhejiang Province (2017PY013 and 2018PY005),

and the Key Laboratory of Radiation Physics and Technology (Sichuan

University), Ministry of Education (2018SCURPT09).

CR Aerts HJWL, 2014, NAT COMMUN, V5, DOI 10.1038/ncomms5006

Awad M., 2015, EFFICIENT LEARNING M, P39, DOI [DOI 10.1007/978-1-4302-5990-9_4, 10.1007/978-1-4302-5990-9_3]

Bangert M, 2010, PHYS MED BIOL, V55, P6023, DOI 10.1088/0031-9155/55/19/025

Bedford JL, 2009, MED PHYS, V36, P5128, DOI 10.1118/1.3240488

Bortfeld T, 2006, PHYS MED BIOL, V51, pR363, DOI 10.1088/0031-9155/51/13/R21

Breedveld S, 2007, PHYS MED BIOL, V52, P6339, DOI 10.1088/0031-9155/52/20/016

Breedveld S, 2012, MED PHYS, V39, P951, DOI 10.1118/1.3676689

Brock J, 2012, CLIN ONCOL-UK, V24, P68, DOI 10.1016/j.clon.2011.02.003

Cagni E, 2017, PHYS MEDICA, V36, P38, DOI 10.1016/j.ejmp.2017.03.002

Chang JY, 2015, LANCET ONCOL, V16, P630, DOI 10.1016/S1470-2045(15)70168-3

Craft D, 2007, INT J RADIAT ONCOL, V69, P1600, DOI 10.1016/j.ijrobp.2007.08.019

Das IJ, 2008, JNCI-J NATL CANCER I, V100, P300, DOI 10.1093/jnci/djn020

Fushiki T, 2011, ESTIMATION PREDICTIO, P137

Good D, 2013, INT J RADIAT ONCOL, V87, P176, DOI 10.1016/j.ijrobp.2013.03.015

Hodapp N, 2012, STRAHLENTHER ONKOL, V188, P97, DOI 10.1007/s00066-011-0015-x

Holt A, 2011, INT J RADIAT ONCOL, V81, P1560, DOI 10.1016/j.ijrobp.2010.09.014

Hou Q, 2003, MED PHYS, V30, P2360, DOI 10.1118/1.1601911

Jiang F, 2017, P 5 INT C BIOINF COM, P59, DOI [DOI 10.1145/3035012.3035022, 10.1145/3035012.3035022.]

Krayenbuehl J, 2015, RADIAT ONCOL, V10, DOI 10.1186/s13014-015-0533-2

Ma ZQ, 2019, PHYS MED BIOL, V64, DOI 10.1088/1361-6560/aaf5da

Masi K, 2015, MED PHYS, V42, P3457, DOI 10.1118/1.4924892

Men K, 2017, MED PHYS, V44, P6377, DOI 10.1002/mp.12602

Mitchell RA, 2017, J APPL CLIN MED PHYS, V18, P18, DOI 10.1002/acm2.12006

Moore KL, 2011, INT J RADIAT ONCOL, V81, P545, DOI 10.1016/j.ijrobp.2010.11.030

Mutanga TF, 2012, INT J RADIAT ONCOL, V83, P400, DOI 10.1016/j.ijrobp.2011.05.049

Nelms BE, 2012, PRACT RADIAT ONCOL, V2, P296, DOI 10.1016/j.prro.2011.11.012

Nwankwo O, 2015, RADIAT ONCOL, V10, DOI 10.1186/s13014-015-0416-6

Nwankwo O, 2014, PHYS MED BIOL, V59, P5575, DOI 10.1088/0031-9155/59/18/5575

Otto K, 2008, MED PHYS, V35, P310, DOI 10.1118/1.2818738

Paddick I, 2000, J NEUROSURG, V93, P219, DOI 10.3171/jns.2000.93.supplement_3.0219

Pardo-Montero J, 2010, MED PHYS, V37, P2606, DOI 10.1118/1.3427410

Pardo-Montero J, 2009, MED PHYS, V36, P3292, DOI 10.1118/1.3151806

Pedregosa F., 2011, J MACH LEARN RES, V12, P2825

Peter M, 2015, J RADIOTHER PRACT, V14, P260, DOI 10.1017/S1460396915000126

Petit SF, 2012, RADIOTHER ONCOL, V102, P38, DOI 10.1016/j.radonc.2011.05.025

Pugachev A, 2001, INT J RADIAT ONCOL, V50, P551, DOI 10.1016/S0360-3016(01)01502-4

Smola AJ, 1998, ALGORITHMICA, V22, P211, DOI 10.1007/PL00013831

Song Y, 2016, RADIOTHER ONCOL, V119, P531, DOI 10.1016/j.radonc.2016.04.010

Tol JP, 2015, RADIAT ONCOL, V10, DOI 10.1186/s13014-015-0388-6

Tol JP, 2015, INT J RADIAT ONCOL, V91, P612, DOI 10.1016/j.ijrobp.2014.11.014

van Baardwijk A, 2012, RADIOTHER ONCOL, V105, P145, DOI 10.1016/j.radonc.2012.09.008

Wang JZ, 2015, MED PHYS, V42, P1005, DOI 10.1118/1.4906252

Wang QY, 2019, J MAGN RESON IMAGING, V49, P825, DOI 10.1002/jmri.26265

Wu BB, 2013, MED PHYS, V40, DOI 10.1118/1.4788671

Wu BB, 2011, INT J RADIAT ONCOL, V79, P1241, DOI 10.1016/j.ijrobp.2010.05.026

Wu BB, 2009, MED PHYS, V36, P5497, DOI 10.1118/1.3253464

Yang YD, 2013, MED PHYS, V40, DOI 10.1118/1.4769424

Zhang XD, 2006, MED PHYS, V33, P2935, DOI 10.1118/1.2214171

Zhu XF, 2011, MED PHYS, V38, P719, DOI 10.1118/1.3539749

NR 49

TC 8

Z9 9

U1 0

U2 3

PU BMC

PI LONDON

PA CAMPUS, 4 CRINAN ST, LONDON N1 9XW, ENGLAND

EI 1475-925X

J9 BIOMED ENG ONLINE

JI Biomed. Eng. Online

PD OCT 16

PY 2019

VL 18

IS 1

AR 101

DI 10.1186/s12938-019-0721-7

PG 15

WC Engineering, Biomedical

WE Science Citation Index Expanded (SCI-EXPANDED)

SC Engineering

GA JE5GJ

UT WOS:000490719700002

PM 31619263

OA gold, Green Published

DA 2022-08-24

ER

PT J

AU Cui, SN

Ten Haken, RK

El Naqa, I

AF Cui, Sunan

Ten Haken, Randall K.

El Naqa, Issam

TI Integrating Multiomics Information in Deep Learning Architectures for

Joint Actuarial Outcome Prediction in Non-Small Cell Lung Cancer

Patients After Radiation Therapy

SO INTERNATIONAL JOURNAL OF RADIATION ONCOLOGY BIOLOGY PHYSICS

LA English

DT Article

ID LOCAL TUMOR-CONTROL; FDG-PET; RADIOTHERAPY; MACHINE; MODEL; PNEUMONITIS;

CONCURRENT; IMPUTATION; SURVIVAL

AB Purpose: Novel actuarial deep learning neural network (ADNN) architectures are proposed for joint prediction of radiation therapy outcomes-radiation pneumonitis (RP) and local control (LC)-in stage III non-small cell lung cancer (NSCLC) patients. Unlike normal tissue complication probability/tumor control probability models that use dosimetric information solely, our proposed models consider complex interactions among multiomics information including positron emission tomography (PET) radiomics, cytokines, and miRNAs. Additional time-to-event information is also used in the actuarial prediction.

Methods and Materials: Three architectures were investigated: ADNN-DVH considered dosimetric information only; ADNN-com integrated multiomics information; and ADNN-com-joint combined RP2 (RP grade >= 2) and LC prediction. In these architectures, differential dose-volume histograms (DVHs) were fed into 1D convolutional neural networks (CNN) for extracting reduced representations. Variational encoders were used to learn representations of imaging and biological data. Reduced representations were fed into Surv-Nets to predict time-to-event probabilities for RP2 and LC independently and jointly by incorporating time information into designated loss functions.

Results: Models were evaluated on 117 retrospective patients and were independently tested on 25 newly accrued patients prospectively. A multi-institutional RTOG0617 data set of 327 patients was used for external validation. ADNN-DVH yielded cross-validated c-indexes (95% confidence intervals) of 0.660 (0.630-0.690) for RP2 prediction and 0.727 (0.700-0.753) for LC prediction, outperforming a generalized Lyman model for RP2 (0.613 [0.583-0.643]) and a generalized log-logistic model for LC (0.569 [0.545-0.594]). The independent internal test and external validation yielded similar results. ADNN-com achieved an even better performance than ADNN-DVH on both cross-validation and independent internal test. Furthermore, ADNN-com-joint, which yielded performance similar to ADNN-com, realized joint prediction with c-indexes of 0.705 (0.676-0.734) for RP2 and 0.740 (0.714-0.765) for LC and achieved an area under a free-response receiving operator characteristic curve (AU-FROC) of 0.729 (0.697-0.773) for the joint prediction of RP2 and LC.

Conclusion: Novel deep learning architectures that integrate multiomics information outperformed traditional normal tissue complication probability/tumor control probability models in actuarial prediction of RP2 and LC. (C) 2021 Elsevier Inc. All rights reserved.

C1 [Cui, Sunan; Ten Haken, Randall K.; El Naqa, Issam] Univ Michigan, Dept Radiat Oncol, Ann Arbor, MI 48109 USA.

[Cui, Sunan] Univ Michigan, Appl Phys Program, Ann Arbor, MI 48109 USA.

RP Cui, SN (通讯作者)，Univ Michigan, Dept Radiat Oncol, Ann Arbor, MI 48109 USA.; Cui, SN (通讯作者)，Univ Michigan, Appl Phys Program, Ann Arbor, MI 48109 USA.

EM sunan@umich.edu

RI cui, sunan/AAA-3286-2020

OI cui, sunan/0000-0002-8846-9449

FU National Institutes of Health (NIH) [P01 CA059827, R01-CA233487]

FX This work was partly supported by grants from National Institutes of

Health (NIH) grants P01 CA059827 and R01-CA233487.

CR [Anonymous], 2020, BBMLE TOOLS GEN MAXI

[Anonymous], 2008, J ICRU, V8, P31

Blagus R, 2013, BMC BIOINFORMATICS, V14, DOI 10.1186/1471-2105-14-106

Boldrini L, 2019, FRONT ONCOL, V9, DOI 10.3389/fonc.2019.00977

Bradley, CANC IMAGING ARCHIVE

Bradley JD, 2015, LANCET ONCOL, V16, P187, DOI 10.1016/S1470-2045(14)71207-0

Ching T, 2018, PLOS COMPUT BIOL, V14, DOI 10.1371/journal.pcbi.1006076

Choi NC, 2002, INT J RADIAT ONCOL, V54, P1024, DOI 10.1016/S0360-3016(02)03038-9

Clark K, 2013, J DIGIT IMAGING, V26, P1045, DOI 10.1007/s10278-013-9622-7

Collins GS, 2015, CIRCULATION, V131, P211, DOI [10.1161/CIRCULATIONAHA.114.014508, 10.7326/M14-0697, 10.1016/j.jclinepi.2014.11.010, 10.1186/s12916-014-0241-z, 10.1136/bmj.g7594, 10.1002/bjs.9736]

COX DR, 1972, J R STAT SOC B, V34, P187

Cui SN, 2020, MED PHYS, V47, pE127, DOI 10.1002/mp.14140

Cui SN, 2019, MED PHYS, V46, P2497, DOI 10.1002/mp.13497

Cui S, 2019, IEEE T RADIAT PLASMA, V3, P242, DOI 10.1109/TRPMS.2018.2884134

DELONG ER, 1988, BIOMETRICS, V44, P837, DOI 10.2307/2531595

El Naqa I., 2018, GUIDE OUTCOME MODELI

El Naqa I, 2018, MED PHYS, V45, pE834, DOI 10.1002/mp.12811

El Naqa I, 2009, PHYS MED BIOL, V54, pS9, DOI 10.1088/0031-9155/54/18/S02

Fiorino C, 2019, MODELLING RADIOTHERA

Gensheimer MF, 2019, PEERJ, V7, DOI 10.7717/peerj.6257

Grad-CAM, 2017, VISUAL EXPLANATIONS

HANLEY JA, 1982, RADIOLOGY, V143, P29, DOI 10.1148/radiology.143.1.7063747

He K., 2016, DEEP RESIDUAL LEARNI, DOI [10.1109/CVPR.2016.90, DOI 10.1109/CVPR.2016.90]

Hicks RJ, 2004, INT J RADIAT ONCOL, V60, P412, DOI 10.1016/j.ijrobp.2004.03.036

Holback C, 2016, RADIOLOGY DATA CANC

Huang G, 2017, PROC CVPR IEEE, P2261, DOI 10.1109/CVPR.2017.243

Isaksson LJ, 2020, FRONT ONCOL, V10, DOI 10.3389/fonc.2020.00790

Jeong HJ, 2002, NUCL MED COMMUN, V23, P865, DOI 10.1097/00006231-200209000-00010

Kainthola A, 2017, FRONT IMMUNOL, V8, DOI 10.3389/fimmu.2017.00506

Kingma D, 2014, ARXIV

Klement RJ, 2014, INT J RADIAT ONCOL, V88, P732, DOI 10.1016/j.ijrobp.2013.11.216

Kong FM, 2005, INT J RADIAT ONCOL, V63, P324, DOI 10.1016/j.ijrobp.2005.02.010

Kumar P, 2016, CANC IMAGING ARCHIVE

Kundrat P, 2018, GUIDE OUTCOME MODELI

Li C, 2018, ADVERSARIAL TIME TO

Li XA, 2012, MED PHYS, V39, P1386, DOI 10.1118/1.3685447

Luo Y, 2020, MED PHYS, V47, pE178, DOI 10.1002/mp.13570

Luo Yi, 2019, BJR Open, V1, P20190021, DOI 10.1259/bjro.20190021

Luo Y, 2018, MED PHYS, V45, P3980, DOI 10.1002/mp.13029

LYMAN JT, 1985, RADIAT RES, V104, pS13, DOI 10.2307/3576626

Massa F, 2019, ADV NEURAL INFORM PR, V32

Murphy M.J., 2015, MACHINE LEARNING RAD, P3, DOI [10.1007/978-3-319-18305-3_1, DOI 10.1007/978-3-319-18305-3_1]

Naqa, 2018, GUIDE OUTCOME MODELI

Nguyen CD, 2017, EMERG THEMES EPIDEMI, V14, DOI 10.1186/s12982-017-0062-6

Pan SJ, 2010, IEEE T KNOWL DATA EN, V22, P1345, DOI 10.1109/TKDE.2009.191

Rahman MG, 2013, KNOWL-BASED SYST, V53, P51, DOI 10.1016/j.knosys.2013.08.023

Seppenwoolde Y, 2004, INT J RADIAT ONCOL, V60, P748, DOI 10.1016/j.ijrobp.2004.04.037

Socinski MA, 2001, CANCER, V92, P1213, DOI 10.1002/1097-0142(20010901)92:5<1213::AID-CNCR1440>3.0.CO;2-0

Tucker SL, 2008, INT J RADIAT ONCOL, V72, P568, DOI 10.1016/j.ijrobp.2008.04.053

Tyldesley S, 2001, INT J RADIAT ONCOL, V49, P973, DOI 10.1016/S0360-3016(00)01401-2

Uno H, 2011, STAT MED, V30, P1105, DOI 10.1002/sim.4154

Vaidya M, 2012, RADIOTHER ONCOL, V102, P239, DOI 10.1016/j.radonc.2011.10.014

Vallieres M, 2015, PHYS MED BIOL, V60, P5471, DOI 10.1088/0031-9155/60/14/5471

Velec M, 2017, INT J RADIAT ONCOL, V97, P939, DOI 10.1016/j.ijrobp.2017.01.221

Velling M, 2014, AUTOENCODING VARIATI

Zhang SC, 2012, J SYST SOFTWARE, V85, P2541, DOI 10.1016/j.jss.2012.05.073

Zwanenburg A, 2020, RADIOLOGY, V295, P328, DOI 10.1148/radiol.2020191145

NR 57

TC 6

Z9 6

U1 2

U2 7

PU ELSEVIER SCIENCE INC

PI NEW YORK

PA STE 800, 230 PARK AVE, NEW YORK, NY 10169 USA

SN 0360-3016

EI 1879-355X

J9 INT J RADIAT ONCOL

JI Int. J. Radiat. Oncol. Biol. Phys.

PD JUL 1

PY 2021

VL 110

IS 3

BP 893

EP 904

DI 10.1016/j.ijrobp.2021.01.042

EA JUN 2021

PG 12

WC Oncology; Radiology, Nuclear Medicine & Medical Imaging

WE Science Citation Index Expanded (SCI-EXPANDED)

SC Oncology; Radiology, Nuclear Medicine & Medical Imaging

GA SM0NA

UT WOS:000657308400038

PM 33539966

OA Bronze, Green Accepted

DA 2022-08-24

ER

PT J

AU Dercle, L

Fronheiser, M

Lu, L

Du, SY

Hayes, W

Leung, DK

Roy, A

Wilkerson, J

Guo, PZ

Fojo, AT

Schwartz, LH

Zhao, BS

AF Dercle, Laurent

Fronheiser, Matthew

Lu, Lin

Du, Shuyan

Hayes, Wendy

Leung, David K.

Roy, Amit

Wilkerson, Julia

Guo, Pingzhen

Fojo, Antonio T.

Schwartz, Lawrence H.

Zhao, Binsheng

TI Identification of Non-Small Cell Lung Cancer Sensitive to Systemic

Cancer Therapies Using Radiomics

SO CLINICAL CANCER RESEARCH

LA English

DT Article

ID FACTOR RECEPTOR MUTATION; COMPUTED-TOMOGRAPHY SCANS; VOLUMETRIC

MEASUREMENT; RESPONSE EVALUATION; CT CHARACTERISTICS; TUMOR

MEASUREMENTS; RADIATION-THERAPY; TEXTURE ANALYSIS; FEATURES; EGFR

AB Purpose: Using standard-of-care CT images obtained from patients with a diagnosis of non-small cell lung cancer (NSCLC), we defined radiomics signatures predicting the sensitivity of tumors to nivolumab, docetaxel, and gefitinib.

Experimental Design: Data were collected prospectively and analyzed retrospectively across multicenter clinical trials [nivolumab, n = 92, CheckMate017 (NCT01642004), Check-Mate063 (NCT01721759); docetaxel, n = 50, CheckMate017; gefitinib, n = 46, (NCT00588445)]. Patients were randomized to training or validation cohorts using either a 4:1 ratio (nivolumab: 72T:20V) or a 2:1 ratio (docetaxel: 32T:18V; gefitinib: 31T:15V) to ensure an adequate sample size in the validation set. Radiomics signatures were derived from quantitative analysis of early tumor changes from baseline to first on-treatment assessment. For each patient, 1,160 radiomics features were extracted from the largest measurable lung lesion. Tumors were classified as treatment sensitive or insensitive; reference standard was median progression-free survival (NCT01642004, NCT01721759) or surgery (NCT00588445). Machine learning was implemented to select up to four features to develop a radiomics signature in the training datasets and applied to each patient in the validation datasets to classify treatment sensitivity.

Results: The radiomics signatures predicted treatment sensitivity in the validation dataset of each study group with AUC (95 confidence interval): nivolumab, 0.77 (0.55-1.00); docetaxel, 0.67 (0.37-0.96); and gefitinib, 0.82 (0.53-0.97). Using serial radiographic measurements, the magnitude of exponential increase in signature features deciphering tumor volume, invasion of tumor boundaries, or tumor spatial heterogeneity was associated with shorter overall survival.

Conclusions: Radiomics signatures predicted tumor sensitivity to treatment in patients with NSCLC, offering an approach that could enhance clinical decision-making to continue systemic therapies and forecast overall survival.

C1 [Dercle, Laurent; Lu, Lin; Guo, Pingzhen; Schwartz, Lawrence H.; Zhao, Binsheng] Columbia Univ, Med Ctr, New York Presbyterian Hosp, Dept Radiol, New York, NY USA.

[Dercle, Laurent] Univ Paris Saclay, Gustave Roussy, Villejuif, France.

[Fronheiser, Matthew; Du, Shuyan; Hayes, Wendy; Leung, David K.] Bristol Myers Squibb, Translat Med, Princeton, NJ USA.

[Roy, Amit] Bristol Myers Squibb, Clin Pharmacol & Pharmacometr, Princeton, NJ USA.

[Wilkerson, Julia] NCI, NIH, Bethesda, MD 20892 USA.

[Fojo, Antonio T.] Columbia Univ, New York Presbyterian Hosp, New York, NY USA.

[Fojo, Antonio T.] James J Peters VA Med Ctr, New York, NY USA.

RP Dercle, L (通讯作者)，Columbia Univ, Med Ctr, 168th St, New York, NY 10032 USA.

EM laurent.dercle@gmail.com

RI Dercle, Laurent/C-9740-2018

OI Dercle, Laurent/0000-0002-1322-0710

FU NIH [U01 CA225431]; Bristol-Myers Squibb; Fondation Philanthropia;

Fondation Nuovo-Soldati

FX Authors acknowledge financial support from the NIH (U01 CA225431) and

Bristol-Myers Squibb. L. Dercle's work was partially funded by grants

from Fondation Philanthropia and Fondation Nuovo-Soldati. The content is

solely the responsibility of the authors and does not necessarily

represent the funding sources.

CR Aerts HJWL, 2016, SCI REP-UK, V6, DOI 10.1038/srep33860

Al-Kadi OS, 2008, IEEE T BIO-MED ENG, V55, P1822, DOI 10.1109/TBME.2008.919735

BREIMAN L, 2001, MACH LEARN, V0045

Chang K, 2016, NEURO-ONCOLOGY, V18, P1680, DOI 10.1093/neuonc/now086

Choi CM, 2015, RADIOLOGY, V275, P272, DOI 10.1148/radiol.14140848

Chow DS, 2014, AM J NEURORADIOL, V35, P498, DOI 10.3174/ajnr.A3724

Coroller TP, 2016, RADIOTHER ONCOL, V119, P480, DOI 10.1016/j.radonc.2016.04.004

Coroller TP, 2015, RADIOTHER ONCOL, V114, P345, DOI 10.1016/j.radonc.2015.02.015

Cunliffe A, 2015, INT J RADIAT ONCOL, V91, P1048, DOI 10.1016/j.ijrobp.2014.11.030

Dercle L, 2020, JNCI J NATL CANC I

Dercle L, 2017, JCO CLIN CANCER INFO, V1, DOI 10.1200/CCI.17.00108

Eisenhauer EA, 2009, EUR J CANCER, V45, P228, DOI 10.1016/j.ejca.2008.10.026

Emaminejad N, 2016, IEEE T BIO-MED ENG, V63, P1034, DOI 10.1109/TBME.2015.2477688

Fried DV, 2014, INT J RADIAT ONCOL, V90, P834, DOI 10.1016/j.ijrobp.2014.07.020

Grossmann P, 2017, ELIFE, V6, DOI 10.7554/eLife.23421

Grove O, 2015, PLOS ONE, V10, DOI 10.1371/journal.pone.0118261

Ha R, 2016, CURR PROBL DIAGN RAD, V45, P297, DOI 10.1067/j.cpradiol.2016.02.003

Ha R, 2016, QUANT IMAG MED SURG, V6, P144, DOI 10.21037/qims.2016.03.03

Hanahan D, 2011, CELL, V144, P646, DOI 10.1016/j.cell.2011.02.013

Hsu JS, 2014, J THORAC IMAG, V29, P357, DOI 10.1097/RTI.0000000000000116

Huang Q, 2018, J MED IMAGING, V5, DOI 10.1117/1.JMI.5.1.011005

Huang YQ, 2016, RADIOLOGY, V281, P947, DOI 10.1148/radiol.2016152234

Kadota K, 2014, AM J SURG PATHOL, V38, P1118, DOI 10.1097/PAS.0000000000000246

Kim TJ, 2016, ANN THORAC SURG, V101, P473, DOI 10.1016/j.athoracsur.2015.07.062

Koshkin VS, 2016, J CLIN ONCOL, V34, P3680, DOI 10.1200/JCO.2016.68.1858

Lambin P, 2017, NAT REV CLIN ONCOL, V14, P749, DOI 10.1038/nrclinonc.2017.141

Lee HJ, 2013, RADIOLOGY, V268, P254, DOI 10.1148/radiol.13112553

Li YJ, 2018, SCI REP-UK, V8, DOI 10.1038/s41598-018-36421-0

Limkin EJ, 2017, ANN ONCOL, V28, P1191, DOI 10.1093/annonc/mdx034

Liu F, 2010, J THORAC ONCOL, V5, P879, DOI 10.1097/JTO.0b013e3181dd0ef1

Liu Y, 2016, CLIN LUNG CANCER, V17, P441, DOI 10.1016/j.cllc.2016.02.001

Liu Y, 2016, RADIOLOGY, V280, P271, DOI 10.1148/radiol.2016151455

Mattonen SA, 2016, INT J RADIAT ONCOL, V94, P1121, DOI 10.1016/j.ijrobp.2015.12.369

Novello S, 2016, ANN ONCOL, V27, pv1, DOI 10.1093/annonc/mdw326

Obuchowski NA, 2005, AM J ROENTGENOL, V184, P364, DOI 10.2214/ajr.184.2.01840364

Oxnard GR, 2011, J CLIN ONCOL, V29, P3114, DOI 10.1200/JCO.2010.33.7071

Ozkan E, 2015, AM J ROENTGENOL, V205, P1016, DOI 10.2214/AJR.14.14147

Rizzo S, 2016, EUR RADIOL, V26, P32, DOI 10.1007/s00330-015-3814-0

Seymour L, 2017, LANCET ONCOL, V18, pE143, DOI 10.1016/S1470-2045(17)30074-8

Shi Z, 2017, SCI REP-UK, V7, DOI 10.1038/s41598-017-00511-2

Sun R, 2018, LANCET ONCOL, V19, P1180, DOI 10.1016/S1470-2045(18)30413-3

Tan Y, 2013, MED PHYS, V40, DOI 10.1118/1.4815174

Terranova N, 2018, CPT-PHARMACOMET SYST, V7, P228, DOI 10.1002/psp4.12284

Trebeschi S, 2017, J CLIN ONCOL, V35, DOI 10.1200/JCO.2017.35.15_suppl.e14520

Wilkerson J, 2017, LANCET ONCOL, V18, P143, DOI 10.1016/S1470-2045(16)30633-7

Wu WM, 2016, FRONT ONCOL, V6, DOI 10.3389/fonc.2016.00071

Yamamoto S, 2014, RADIOLOGY, V272, P568, DOI 10.1148/radiol.14140789

Yang Y, 2015, LUNG CANCER, V87, P272, DOI 10.1016/j.lungcan.2014.12.016

Yoon HJ, 2015, MEDICINE, V94, DOI 10.1097/MD.0000000000001753

Zhao BS, 2016, SCI REP-UK, V6, DOI 10.1038/srep23428

Zhao BS, 2010, CLIN CANCER RES, V16, P4647, DOI 10.1158/1078-0432.CCR-10-0125

Zhao BS, 2009, RADIOLOGY, V252, P263, DOI 10.1148/radiol.2522081593

Zhou JY, 2015, EUR RADIOL, V25, P1257, DOI 10.1007/s00330-014-3516-z

NR 53

TC 44

Z9 45

U1 4

U2 14

PU AMER ASSOC CANCER RESEARCH

PI PHILADELPHIA

PA 615 CHESTNUT ST, 17TH FLOOR, PHILADELPHIA, PA 19106-4404 USA

SN 1078-0432

EI 1557-3265

J9 CLIN CANCER RES

JI Clin. Cancer Res.

PD MAY

PY 2020

VL 26

IS 9

BP 2151

EP 2162

DI 10.1158/1078-0432.CCR-19-2942

PG 12

WC Oncology

WE Science Citation Index Expanded (SCI-EXPANDED)

SC Oncology

GA LL8LT

UT WOS:000531806800010

PM 32198149

OA Green Accepted

DA 2022-08-24

ER

PT J

AU Cheng, J

Pan, Y

Huang, W

Huang, K

Cui, YH

Hong, WH

Wang, LL

Ni, D

Tan, PX

AF Cheng, Jun

Pan, Yi

Huang, Wei

Huang, Kun

Cui, Yanhai

Hong, Wenhui

Wang, Lingling

Ni, Dong

Tan, Peixin

TI Differentiation between immune checkpoint inhibitor-related and

radiation pneumonitis in lung cancer by CT radiomics and machine

learning

SO MEDICAL PHYSICS

LA English

DT Article

DE CT radiomics; immune checkpoint inhibitor-related pneumonitis; lung

cancer; machine learning; radiation pneumonitis

ID RADIOGRAPHIC PATTERNS; RADIOTHERAPY; CHEMORADIOTHERAPY; CHEMORADIATION;

PEMBROLIZUMAB; CHEMOTHERAPY; CONCURRENT; MUTATIONS; RISK

AB Purpose Consolidation immunotherapy after completion of chemoradiotherapy has become the standard of care for unresectable locally advanced non-small cell lung cancer and can induce potentially severe and life-threatening adverse events, including both immune checkpoint inhibitor-related pneumonitis (CIP) and radiation pneumonitis (RP), which are very challenging for radiologists to diagnose. Differentiating between CIP and RP has significant implications for clinical management such as the treatments for pneumonitis and the decision to continue or restart immunotherapy. The purpose of this study is to differentiate between CIP and RP by a CT radiomics approach. Methods We retrospectively collected the CT images and clinical information of patients with pneumonitis who received immune checkpoint inhibitor (ICI) only (n = 28), radiotherapy (RT) only (n = 31), and ICI+RT (n = 14). Three kinds of radiomic features (intensity histogram, gray-level co-occurrence matrix [GLCM] based, and bag-of-words [BoW] features) were extracted from CT images, which characterize tissue texture at different scales. Classification models, including logistic regression, random forest, and linear SVM, were first developed and tested in patients who received ICI or RT only with 10-fold cross-validation and further tested in patients who received ICI+RT using clinicians' diagnosis as a reference. Results Using 10-fold cross-validation, the classification models built on the intensity histogram features, GLCM-based features, and BoW features achieved an area under curve (AUC) of 0.765, 0.848, and 0.937, respectively. The best model was then applied to the patients receiving combination treatment, achieving an AUC of 0.896. Conclusions This study demonstrates the promising potential of radiomic analysis of CT images for differentiating between CIP and RP in lung cancer, which could be a useful tool to attribute the cause of pneumonitis in patients who receive both ICI and RT.

C1 [Cheng, Jun; Hong, Wenhui; Wang, Lingling; Ni, Dong] Shenzhen Univ, Hlth Sci Ctr, Sch Biomed Engn, Natl Reg Key Technol Engn Lab Med Ultrasound,Guan, Shenzhen, Peoples R China.

[Cheng, Jun; Ni, Dong] Shenzhen Univ, Med Ultrasound Image Comp MUSIC Lab, Shenzhen, Peoples R China.

[Cheng, Jun; Ni, Dong] Shenzhen Univ, Marshall Lab Biomed Engn, Shenzhen, Peoples R China.

[Pan, Yi; Huang, Wei; Tan, Peixin] Guangdong Acad Med Sci, Guangdong Prov Peoples Hosp, Dept Radiat Oncol, Guangzhou, Peoples R China.

[Huang, Kun] Indiana Univ Sch Med, Dept Biostat & Hlth Data Sci, Indianapolis, IN 46202 USA.

[Huang, Kun] Regenstrief Inst Hlth Care, Indianapolis, IN USA.

[Cui, Yanhai] Guangdong Acad Med Sci, Guangdong Prov Peoples Hosp, Dept Radiol, Guangzhou, Peoples R China.

RP Ni, D (通讯作者)，Sch Biomed Engn, 1066 Xueyuan Ave, Shenzhen 518055, Peoples R China.; Tan, PX (通讯作者)，Dept Radiat Oncol, 106 Zhongshan 2nd Rd, Guangzhou 510080, Peoples R China.

EM nidong@szu.edu.cn; tpxsaxin@163.com

FU National Natural Science Foundation of China [61901275]; Guangzhou

Science and Technology Plan Foundation [2021-02-01-04-1002-0017];

Shenzhen University Startup Fund [2019131]; National Key R&D Program of

China [2019YFC0118300]; Shenzhen Peacock Plan [KQTD2016053112051497,

KQJSCX20180328095606003]; Medical Scientific Research Foundation of

Guangdong Province, China [B2018031, B2020024]; National Natural Science

Foundation of Guangdong Provincial People's Hospital [8210032051]

FX The authors thank Haiyan Tu, Biao Huang, and Jine Zhang from Guangdong

Provincial People's Hospital, China, for retrieving and reviewing the

chest CT scans of patients. This study was supported by National Natural

Science Foundation of China (61901275), Guangzhou Science and Technology

Plan Foundation (2021-02-01-04-1002-0017), Shenzhen University Startup

Fund (2019131), National Key R&D Program of China (2019YFC0118300),

Shenzhen Peacock Plan (KQTD2016053112051497 and

KQJSCX20180328095606003), Medical Scientific Research Foundation of

Guangdong Province, China (B2018031 and B2020024), and Supporting

start-up funds of National Natural Science Foundation of Guangdong

Provincial People's Hospital (8210032051). The funding sources have no

involvement in the study.

CR Antonia SJ, 2018, NEW ENGL J MED, V379, P2342, DOI 10.1056/NEJMoa1809697

Antonia SJ, 2017, NEW ENGL J MED, V377, P1919, DOI 10.1056/NEJMoa1709937

Beig N, 2019, RADIOLOGY, V290, P783, DOI 10.1148/radiol.2018180910

Bera Kaustav, 2018, Am Soc Clin Oncol Educ Book, V38, P1008, DOI 10.1200/EDBK_199747

Bernchou U, 2017, RADIOTHER ONCOL, V123, P93, DOI 10.1016/j.radonc.2017.02.001

Bledsoe TJ, 2017, CLIN CHEST MED, V38, P201, DOI 10.1016/j.ccm.2016.12.004

Bradley J, 2006, CANC TREAT, V128, P43

Bradley JD, 2015, LANCET ONCOL, V16, P187, DOI 10.1016/S1470-2045(14)71207-0

Brahmer JR, 2018, J CLIN ONCOL, V36, P1714, DOI 10.1200/JCO.2017.77.6385

Cadranel J, 2019, EUR RESPIR REV, V28, DOI 10.1183/16000617.0058-2019

Chen AP, 2019, AM J ROENTGENOL, V213, P134, DOI 10.2214/AJR.18.20591

Chen X, 2020, INT J RADIAT ONCOL, V108, pS163

Cheng J, 2015, PLOS ONE, V10, DOI 10.1371/journal.pone.0115339

Cho JY, 2018, LUNG CANCER, V125, P150, DOI 10.1016/j.lungcan.2018.09.015

Chuzi S, 2017, CANCER MANAG RES, V9, P207, DOI 10.2147/CMAR.S136818

Coroller TP, 2015, RADIOTHER ONCOL, V114, P345, DOI 10.1016/j.radonc.2015.02.015

Curran WJ, 2011, J NATL CANCER I, V103, P1452, DOI 10.1093/jnci/djr325

Gandhi L, 2018, NEW ENGL J MED, V378, P2078, DOI 10.1056/NEJMoa1801005

Jiang YM, 2021, ANN SURG, V274, pE1153, DOI 10.1097/SLA.0000000000003778

Khorrami M, 2019, LUNG CANCER, V135, P1, DOI 10.1016/j.lungcan.2019.06.020

Lin SH, 2020, J THORAC ONCOL, V15, P248, DOI 10.1016/j.jtho.2019.10.024

Naidoo J, 2020, CLIN LUNG CANCER, V21, pE435, DOI 10.1016/j.cllc.2020.02.025

Naidoo J, 2017, J CLIN ONCOL, V35, P709, DOI 10.1200/JCO.2016.68.2005

Nishino M, 2016, CLIN CANCER RES, V22, P6051, DOI 10.1158/1078-0432.CCR-16-1320

Nishino M, 2016, JAMA ONCOL, V2, P1607, DOI 10.1001/jamaoncol.2016.2453

Peters S, 2019, LUNG CANCER, V133, P83, DOI 10.1016/j.lungcan.2019.05.001

Reck M, 2016, NEW ENGL J MED, V375, P1823, DOI 10.1056/NEJMoa1606774

Rizzo S, 2016, EUR RADIOL, V26, P32, DOI 10.1007/s00330-015-3814-0

SAITO G, 2020, J CLIN ONCOL, V38

Schoenfeld JD, 2019, J IMMUNOTHER CANCER, V7, DOI 10.1186/s40425-019-0583-3

Shaverdian N, 2017, LANCET ONCOL, V18, P895, DOI 10.1016/S1470-2045(17)30380-7

Sun R, 2018, LANCET ONCOL, V19, P1180, DOI 10.1016/S1470-2045(18)30413-3

Thomas R, 2020, LUNG CANCER, V145, P132, DOI 10.1016/j.lungcan.2020.03.023

Tirumani SH, 2015, CANCER IMMUNOL RES, V3, P1185, DOI 10.1158/2326-6066.CIR-15-0102

Torheim T, 2014, IEEE T MED IMAGING, V33, P1648, DOI 10.1109/TMI.2014.2321024

Uthoff J, 2019, TRANSL LUNG CANCER R, V8, P979, DOI 10.21037/tlcr.2019.12.19

Velazquez ER, 2017, CANCER RES, V77, P3922, DOI 10.1158/0008-5472.CAN-17-0122

Voong KR, 2019, CLIN LUNG CANCER, V20, pE470, DOI 10.1016/j.cllc.2019.02.018

Wang SL, 2008, J THORAC ONCOL, V3, P277, DOI 10.1097/JTO.0b013e3181653ca6

Xu X, 2019, J HEPATOL, V70, P1133, DOI 10.1016/j.jhep.2019.02.023

Xu YW, 2019, CLIN CANCER RES, V25, P3266, DOI 10.1158/1078-0432.CCR-18-2495

NR 41

TC 3

Z9 3

U1 2

U2 2

PU WILEY

PI HOBOKEN

PA 111 RIVER ST, HOBOKEN 07030-5774, NJ USA

SN 0094-2405

EI 2473-4209

J9 MED PHYS

JI Med. Phys.

PD MAR

PY 2022

VL 49

IS 3

BP 1547

EP 1558

DI 10.1002/mp.15451

EA JAN 2022

PG 12

WC Radiology, Nuclear Medicine & Medical Imaging

WE Science Citation Index Expanded (SCI-EXPANDED)

SC Radiology, Nuclear Medicine & Medical Imaging

GA ZP2EX

UT WOS:000747296500001

PM 35026041

OA Green Published, hybrid

DA 2022-08-24

ER

PT J

AU Chao, HH

Valdes, G

Luna, JM

Heskel, M

Berman, AT

Solberg, TD

Simone, CB

AF Chao, Hann-Hsiang

Valdes, Gilmer

Luna, Jose M.

Heskel, Marina

Berman, Abigail T.

Solberg, Timothy D.

Simone, Charles B.

TI Exploratory analysis using machine learning to predict for chest wall

pain in patients with stage I non-small-cell lung cancer treated with

stereotactic body radiation therapy

SO JOURNAL OF APPLIED CLINICAL MEDICAL PHYSICS

LA English

DT Article

DE chest wall pain; dosimetry; machine learning; non-small-cell lung

cancer; SBRT

ID INDUCED RIB FRACTURES; ABLATIVE RADIOTHERAPY; DECISION-SUPPORT;

RISK-FACTORS; STRATEGIES; TOXICITY

AB Background and purpose: Chest wall toxicity is observed after stereotactic body radiation therapy (SBRT) for peripherally located lung tumors. We utilize machine learning algorithms to identify toxicity predictors to develop dose volume constraints.

Materials and methods: Twenty-five patient, tumor, and dosimetric features were recorded for 197 consecutive patients with Stage I NSCLC treated with SBRT, 11 of whom (5.6%) developed CTCAEv4 grade >= 2 chest wall pain. Decision tree modeling was used to determine chest wall syndrome (CWS) thresholds for individual features. Significant features were determined using independent multivariate methods. These methods incorporate out-of-bag estimation using Random forests (RF) and bootstrapping (100 iterations) using decision trees.

Results: Univariate analysis identified rib dose to 1 cc < 4000 cGy (P = 0.01), chest wall dose to 30 cc < 1900 cGy (P = 0.035), rib Dmax < 5100 cGy (P = 0.05) and lung dose to 1000 cc < 70 cGy (P = 0.039) to be statistically significant thresholds for avoiding CWS. Subsequent multivariate analysis confirmed the importance of rib dose to 1 cc, chest wall dose to 30 cc, and rib Dmax. Using learning-curve experiments, the dataset proved to be self-consistent and provides a realistic model for CWS analysis.

Conclusions: Using machine learning algorithms in this first of its kind study, we identify robust features and cutoffs predictive for the rare clinical event of CWS. Additional data in planned subsequent multicenter studies will help increase the accuracy of multivariate analysis.

C1 [Chao, Hann-Hsiang; Valdes, Gilmer; Luna, Jose M.; Heskel, Marina; Berman, Abigail T.; Solberg, Timothy D.] Univ Penn, Dept Radiat Oncol, Philadelphia, PA 19104 USA.

[Valdes, Gilmer; Solberg, Timothy D.] Univ Calif San Francisco, Dept Radiat Oncol, San Francisco, CA 94143 USA.

[Simone, Charles B.] Univ Maryland, Sch Med, Dept Radiat Oncol, Baltimore, MD 21201 USA.

RP Simone, CB (通讯作者)，Univ Maryland, Sch Med, Dept Radiat Oncol, Baltimore, MD 21201 USA.

EM charlessimone@umm.edu

RI Luna, Jose Marcio/ABG-1296-2020

OI , Timothy/0000-0001-8829-7774; Simone, Charles/0000-0002-0867-3694;

Luna, Jose/0000-0002-5513-022X

CR Andolino DL, 2011, INT J RADIAT ONCOL, V80, P692, DOI 10.1016/j.ijrobp.2010.03.020

[Anonymous], 2001, SPRINGE SER STAT N

Aoki M, 2015, RADIAT ONCOL, V10, DOI 10.1186/s13014-015-0406-8

Asai K, 2012, INT J RADIAT ONCOL, V84, P768, DOI 10.1016/j.ijrobp.2012.01.027

Baumann BC, 2016, J SURG ONCOL, V114, P65, DOI 10.1002/jso.24268

Bongers EM, 2011, J THORAC ONCOL, V6, P2052, DOI 10.1097/JTO.0b013e3182307e74

Breiman L, 2001, MACH LEARN, V45, P27

Breiman L., 2004, 670 UC BERK

Chang JY, 2015, LANCET ONCOL, V16, P630, DOI 10.1016/S1470-2045(15)70168-3

Creach KM, 2012, RADIOTHER ONCOL, V104, P23, DOI 10.1016/j.radonc.2012.01.014

Darcy AM, 2016, JAMA-J AM MED ASSOC, V315, P551, DOI 10.1001/jama.2015.18421

Dunlap NE, 2010, INT J RADIAT ONCOL, V76, P796, DOI 10.1016/j.ijrobp.2009.02.027

El Naqa I, 2010, ACTA ONCOL, V49, P1363, DOI 10.3109/02841861003649224

Hastie T., 2009, ELEMENTS STAT LEARNI, V2nd ed.

Kim SS, 2013, LUNG CANCER, V79, P161, DOI 10.1016/j.lungcan.2012.10.011

Kimsey F, 2016, SEMIN RADIAT ONCOL, V26, P129, DOI 10.1016/j.semradonc.2015.11.003

Lambin P, 2013, RADIOTHER ONCOL, V109, P159, DOI 10.1016/j.radonc.2013.07.007

Lo SS, 2013, CLIN ONCOL-UK, V25, P378, DOI 10.1016/j.clon.2013.01.003

Luna JM, 2017, ARXIV171106793

Mutter RW, 2012, INT J RADIAT ONCOL, V82, P1783, DOI 10.1016/j.ijrobp.2011.03.053

Nambu A, 2013, BMC CANCER, V13, DOI 10.1186/1471-2407-13-68

Nambu A, 2011, RADIAT ONCOL, V6, DOI 10.1186/1748-717X-6-137

Pettersson N, 2009, RADIOTHER ONCOL, V91, P360, DOI 10.1016/j.radonc.2009.03.022

Shirvani SM, 2012, INT J RADIAT ONCOL, V84, P1060, DOI 10.1016/j.ijrobp.2012.07.2354

Simone CB, 2015, ANN TRANSL MED, V3, DOI 10.3978/j.issn.2305-5839.2015.07.26

Simone CB, 2013, CHEST, V143, P1784, DOI 10.1378/chest.12-2580

Stam B, 2017, RADIOTHER ONCOL, V123, P176, DOI 10.1016/j.radonc.2017.01.004

Stephans KL, 2012, INT J RADIAT ONCOL, V82, P974, DOI 10.1016/j.ijrobp.2010.12.002

Taremi M, 2012, RADIAT ONCOL, V7, DOI 10.1186/1748-717X-7-159

Thibault I, 2016, CLIN ONCOL-UK, V28, P28, DOI 10.1016/j.clon.2015.06.009

Valdes G, 2016, MED PHYS, V43, P4323, DOI 10.1118/1.4953835

Valdes G, 2017, RADIOTHER ONCOL, V125, P392, DOI 10.1016/j.radonc.2017.10.014

Valdes G, 2017, J APPL CLIN MED PHYS, V18, P279, DOI 10.1002/acm2.12161

Valdes G, 2016, SCI REP-UK, V6, DOI 10.1038/srep37854

Valdes G, 2016, PHYS MED BIOL, V61, P6105, DOI 10.1088/0031-9155/61/16/6105

Valdes G, 2015, J APPL CLIN MED PHYS, V16, P322, DOI 10.1120/jacmp.v16i4.5363

Verma V, 2017, CLIN LUNG CANCER, V18, P675, DOI 10.1016/j.cllc.2017.03.009

Verma V, 2017, INT J RADIAT ONCOL, V97, P362, DOI 10.1016/j.ijrobp.2016.10.041

Woody NM, 2012, INT J RADIAT ONCOL, V83, P427, DOI 10.1016/j.ijrobp.2011.06.1971

Zheng XP, 2014, INT J RADIAT ONCOL, V90, P603, DOI 10.1016/j.ijrobp.2014.05.055

NR 40

TC 8

Z9 8

U1 0

U2 7

PU WILEY

PI HOBOKEN

PA 111 RIVER ST, HOBOKEN 07030-5774, NJ USA

SN 1526-9914

J9 J APPL CLIN MED PHYS

JI J. Appl. Clin. Med. Phys

PD SEP

PY 2018

VL 19

IS 5

BP 539

EP 546

DI 10.1002/acm2.12415

PG 8

WC Radiology, Nuclear Medicine & Medical Imaging

WE Science Citation Index Expanded (SCI-EXPANDED)

SC Radiology, Nuclear Medicine & Medical Imaging

GA GS5GL

UT WOS:000443685500026

PM 29992732

OA Green Published, gold

DA 2022-08-24

ER

PT J

AU Yan, MM

Wang, WD

AF Yan, Mengmeng

Wang, Weidong

TI A radiomics model of predicting tumor volume change of patients with

stage III non-small cell lung cancer after radiotherapy

SO SCIENCE PROGRESS

LA English

DT Article

DE Radiomics; lung cancer; medical knowledge; precision medicine

AB To predict the volume change of stage III NSCLC after radiotherapy with 60 Gy.

This retrospective study included two independent cohorts, a train cohort of 192 patients, and a test cohort of 31 patients. We developed a radiomics model based on radiomics features and clinical variables. LIFEx package was used to extract radiomics texture features from CT images. The classification method was logistic regression analysis and feature selection was performed by correlation coefficients. Performance metrics of logistic regression include accuracy, precision, the receiver operating characteristic curves, and recall.

The combination features of clinical variables and radiomics can predict the tumor volume change after radiotherapy with 88.7% accuracy (88.6% precision, 88.7% recall, and 88.7% ROC area).

Radiomics features combined with medical knowledge have a great potential to predict accurately tumor volume change of stage III NSCLC after radiotherapy with 60 Gy.

C1 [Yan, Mengmeng] Urban Vocat Coll Sichuan, Chengdu, Peoples R China.

[Yan, Mengmeng] Univ Elect Sci & Technol China, Sch Med, Chengdu, Peoples R China.

[Wang, Weidong] Sichuan Canc Hosp & Inst, Dept Radiat Oncol, 55,Sect 4,South Renmin Rd, Chengdu 610041, Peoples R China.

[Wang, Weidong] Radiat Oncol Key Lab Sichuan Prov, Chengdu, Peoples R China.

RP Wang, WD (通讯作者)，Sichuan Canc Hosp & Inst, Dept Radiat Oncol, 55,Sect 4,South Renmin Rd, Chengdu 610041, Peoples R China.

EM 18380171863@163.com

FU National Key Research and Development program [2017YFC0113904]

FX The author(s) disclosed receipt of the following financial support for

the research, authorship, and/or publication of this article: This study

was supported by National Key Research and Development program

(2017YFC0113904).

CR Aerts HJWL, 2016, SCI REP-UK, V6, DOI 10.1038/srep33860

Ather S, 2020, CLIN RADIOL, V75, P13, DOI 10.1016/j.crad.2019.04.017

Avanzo M, 2020, MED PHYS, V47, pE185, DOI 10.1002/mp.13678

Avanzo M, 2020, FRONT ONCOL, V10, DOI 10.3389/fonc.2020.00490

Avanzo M, 2020, STRAHLENTHER ONKOL, V196, P879, DOI 10.1007/s00066-020-01625-9

Buckley AM, 2020, NAT REV GASTRO HEPAT, V17, P298, DOI 10.1038/s41575-019-0247-2

Bulens P, 2020, RADIOTHER ONCOL, V142, P246, DOI 10.1016/j.radonc.2019.07.033

Costa B, 2020, EBIOMEDICINE, V51, DOI 10.1016/j.ebiom.2019.11.039

de Jong EEC, 2019, EUR J CANCER, V120, P107, DOI 10.1016/j.ejca.2019.07.023

Du Y, 2019, CANCER LETT, V466, P13, DOI 10.1016/j.canlet.2019.08.009

Ferreira JR, 2020, INT J COMPUT ASS RAD, V15, P163, DOI 10.1007/s11548-019-02093-y

Forghani R, 2019, COMPUT STRUCT BIOTEC, V17, P995, DOI 10.1016/j.csbj.2019.07.001

Fornacon-Wood I, 2020, LUNG CANCER, V146, P197, DOI 10.1016/j.lungcan.2020.05.028

Galldiks N, 2020, NEURO-ONCOLOGY, V22, P17, DOI 10.1093/neuonc/noz147

Ger RB, 2019, PLOS ONE, V14, DOI 10.1371/journal.pone.0222509

Gurtner K, 2020, INT J CANCER, V147, P472, DOI 10.1002/ijc.32598

Hyun SH, 2019, CLIN NUCL MED, V44, P956, DOI 10.1097/RLU.0000000000002810

Isaksson LJ, 2020, FRONT ONCOL, V10, DOI 10.3389/fonc.2020.00790

Koyasu S, 2020, ANN NUCL MED, V34, P49, DOI 10.1007/s12149-019-01414-0

Lee G, 2020, KOREAN J RADIOL, V21, P159, DOI 10.3348/kjr.2019.0630

Mohammadi H, 2020, INT J RADIAT ONCOL, V106, P496, DOI 10.1016/j.ijrobp.2019.11.013

Nioche C, 2017, J NUCL MED, V58

Rogers W, 2020, BRIT J RADIOL, V93, DOI 10.1259/bjr.20190948

Sanz-Santos J, 2020, RESPIROLOGY, V25, P37, DOI 10.1111/resp.13901

Schwartz DL, 2020, HEMATOL ONCOL CLIN N, V34, P91, DOI 10.1016/j.hoc.2019.08.019

Scott JG, 2017, LANCET ONCOL, V18, P202, DOI 10.1016/S1470-2045(16)30648-9

Shboul ZA, 2019, FRONT NEUROSCI-SWITZ, V13, DOI 10.3389/fnins.2019.00966

Song WL, 2020, J MAGN RESON IMAGING, V52, P461, DOI 10.1002/jmri.26977

Tiwari P, 2020, INT J RADIAT BIOL, V96, P360, DOI 10.1080/09553002.2020.1694193

van Laar M, 2020, RADIOTHER ONCOL, V151, P152, DOI 10.1016/j.radonc.2020.07.030

Witten IH, 2011, MOR KAUF D, P1

Xiong QQ, 2020, CLIN TRANSL ONCOL, V22, P50, DOI 10.1007/s12094-019-02109-8

Yan MM, 2020, FRONT ONCOL, V10, DOI 10.3389/fonc.2020.00602

Zhao LN, 2020, EUR RADIOL, V30, P537, DOI 10.1007/s00330-019-06211-x

Zwanenburg A, ARXIV161207003 CORN

NR 35

TC 0

Z9 0

U1 0

U2 2

PU SAGE PUBLICATIONS LTD

PI LONDON

PA 1 OLIVERS YARD, 55 CITY ROAD, LONDON EC1Y 1SP, ENGLAND

SN 0036-8504

EI 2047-7163

J9 SCI PROGRESS-UK

JI Sci. Prog.

PD JAN

PY 2021

VL 104

IS 1

AR 0036850421997295

DI 10.1177/0036850421997295

PG 10

WC Education, Scientific Disciplines; Multidisciplinary Sciences

WE Science Citation Index Expanded (SCI-EXPANDED)

SC Education & Educational Research; Science & Technology - Other Topics

GA QV2DS

UT WOS:000627787800001

PM 33687294

OA gold

DA 2022-08-24

ER

PT J

AU Jiao, ZC

Li, HM

Xiao, Y

Dorsey, J

Simone, CB

Feigenberg, S

Kao, G

Fan, Y

AF Jiao, Zhicheng

Li, Hongming

Xiao, Ying

Dorsey, Jay

Simone, Charles B.

Feigenberg, Steven

Kao, Gary

Fan, Yong

TI Integration of Deep Learning Radiomics and Counts of Circulating Tumor

Cells Improves Prediction of Outcomes of Early Stage NSCLC Patients

Treated With Stereotactic Body Radiation Therapy

SO INTERNATIONAL JOURNAL OF RADIATION ONCOLOGY BIOLOGY PHYSICS

LA English

DT Article

ID LUNG-CANCER PATIENTS; TREATMENT RESPONSE; SURVIVAL; RADIOTHERAPY;

BIOMARKERS; PROGNOSIS; SIGNATURE; MEDICINE; FEATURES; DISEASE

AB Purpose: We develop a deep learning (DL) radiomics model and integrate it with circulating tumor cell (CTC) counts as a clinically useful prognostic marker for predicting recurrence outcomes of early-stage (ES) non-small cell lung cancer (NSCLC) patients treated with stereotactic body radiation therapy (SBRT).

Methods and Materials: A cohort of 421 NSCLC patients was used to train a DL model for gleaning informative imaging features from computed tomography (CT) data. The learned imaging features were optimized on a cohort of 98 ES-NSCLC patients treated with SBRT for predicting individual patient recurrence risks by building DL models on CT data and clinical measures. These DL models were validated on the third cohort of 60 ES-NSCLC patients treated with SBRT to predict recurrent risks and stratify patients into subgroups with distinct outcomes in conjunction with CTC counts.

Results: The DL model obtained a concordance-index of 0.880 (95% confidence interval, 0.879-0.881). Patient subgroups with low and high DL risk scores had significantly different recurrence outcomes (P = 3.5e-04). The integration of DL risk scores and CTC measures identified 4 subgroups of patients with significantly different risks of recurrence (chi(2) = 20.11, P = 1.6e-04). Patients with positive CTC measures were associated with increased risks of recurrence that were significantly different from patients with negative CTC measures (P = 0.0447).

Conclusions: In this first-ever study integrating DL radiomics models and CTC counts, our results suggested that this integration improves patient stratification compared with either imagining data or CTC measures alone in predicting recurrence outcomes for patients treated with SBRT for ES-NSCLC. (C) 2021 Elsevier Inc. All rights reserved.

C1 [Jiao, Zhicheng; Li, Hongming; Fan, Yong] Univ Penn, Dept Radiol, Perelman Sch Med, Philadelphia, PA 19104 USA.

[Xiao, Ying; Dorsey, Jay; Feigenberg, Steven; Kao, Gary] Univ Penn, Dept Radiat Oncol, Perelman Sch Med, Philadelphia, PA 19104 USA.

[Simone, Charles B.] New York Proton Ctr, New York, NY USA.

[Simone, Charles B.] Mem Sloan Kettering Canc Ctr, Dept Radiat Oncol, 1275 York Ave, New York, NY 10021 USA.

RP Fan, Y (通讯作者)，Univ Penn, Dept Radiol, Perelman Sch Med, Philadelphia, PA 19104 USA.

EM yong.fan@pennmedicine.upenn.edu

FU National Cancer Institute of the National Institutes of Health

[CA223358]

FX This study was supported by the National Cancer Institute of the

National Institutes of Health under award number CA223358.

CR Aboutalib SS, 2018, CLIN CANCER RES, V24, P5902, DOI 10.1158/1078-0432.CCR-18-1115

Aceto N, 2014, CELL, V158, P1110, DOI 10.1016/j.cell.2014.07.013

Aerts HJWL, 2014, NAT COMMUN, V5, DOI 10.1038/ncomms5006

Chaudharyl K, 2018, CLIN CANCER RES, V24, P1248, DOI 10.1158/1078-0432.CCR-17-0853

Coroller TP, 2015, RADIOTHER ONCOL, V114, P345, DOI 10.1016/j.radonc.2015.02.015

Danila DC, 2007, CLIN CANCER RES, V13, P7053, DOI 10.1158/1078-0432.CCR-07-1506

Dorsey JF, 2015, CANCER-AM CANCER SOC, V121, P139, DOI 10.1002/cncr.28975

Ettinger DS, 2012, J NATL COMPR CANC NE, V10, P1236, DOI 10.6004/jnccn.2012.0130

FLEMING TR, 1981, COMMUN STAT A-THEOR, V10, P763, DOI 10.1080/03610928108828073

Frick MA, 2020, CLIN CANCER RES, V26, P2372, DOI 10.1158/1078-0432.CCR-19-2158

Frick MA, 2018, INT J RADIAT ONCOL, V102, P536, DOI 10.1016/j.ijrobp.2018.06.041

Henschke CI, 2006, NEW ENGL J MED, V355, P1763, DOI 10.1056/NEJMoa060476

Hosny A, 2018, PLOS MED, V15, DOI 10.1371/journal.pmed.1002711

Hou JM, 2012, J CLIN ONCOL, V30, P525, DOI 10.1200/JCO.2010.33.3716

Howlander N, 2019, SEER CANC STAT REV C

Huang YQ, 2016, RADIOLOGY, V281, P947, DOI 10.1148/radiol.2016152234

Jiao ZC, 2021, INT J RADIAT ONCOL, V109, P1647, DOI 10.1016/j.ijrobp.2020.12.014

Kang L, 2015, STAT MED, V34, P685, DOI 10.1002/sim.6370

Kapadia NS, 2017, ANN THORAC SURG, V104, P1881, DOI 10.1016/j.athoracsur.2017.06.065

Kong FM, 2017, TRANSL LUNG CANCER R, V6, P713, DOI 10.21037/tlcr.2017.09.11

Kovalchik SA, 2013, NEW ENGL J MED, V369, P245, DOI 10.1056/NEJMoa1301851

Krebs MG, 2011, J CLIN ONCOL, V29, P1556, DOI 10.1200/JCO.2010.28.7045

Lambin P, 2012, EUR J CANCER, V48, P441, DOI 10.1016/j.ejca.2011.11.036

LeCun Y, 2015, NATURE, V521, P436, DOI 10.1038/nature14539

Lee G, 2017, EUR J RADIOL, V86, P297, DOI 10.1016/j.ejrad.2016.09.005

Li HM, 2019, I S BIOMED IMAGING, P846, DOI 10.1109/ISBI.2019.8759301

Li HM, 2018, RADIOTHER ONCOL, V129, P218, DOI 10.1016/j.radonc.2018.06.025

Lim C, 2017, CURR ONCOL, V24, P103, DOI 10.3747/co.24.3495

Lin DY, 2007, LIFETIME DATA ANAL, V13, P471, DOI 10.1007/s10985-007-9048-y

Liu HF, 2020, IEEE T BIO-MED ENG, V67, P2735, DOI 10.1109/TBME.2020.2969839

MacArthur KM, 2014, CANCER RES, V74, P2152, DOI 10.1158/0008-5472.CAN-13-0813

MARTINI N, 1975, J THORAC CARDIOV SUR, V70, P606

Mukherjee P, 2020, NAT MACH INTELL, V2, P274, DOI 10.1038/s42256-020-0173-6

Palma D, 2010, J CLIN ONCOL, V28, P5153, DOI 10.1200/JCO.2010.30.0731

Pignon JP, 2008, J CLIN ONCOL, V26, P3552, DOI 10.1200/JCO.2007.13.9030

Shah JL, 2017, SEMIN RADIAT ONCOL, V27, P218, DOI 10.1016/j.semradonc.2017.03.001

Siegel RL, 2022, CA-CANCER J CLIN, V72, P7, DOI [10.3322/caac.21332, 10.3322/caac.21708, 10.3322/caac.21551]

Timmerman RD, 2018, JAMA ONCOL, V4, P1263, DOI 10.1001/jamaoncol.2018.1251

Vachani A, 2017, AM J RESP CRIT CARE, V195, P1150, DOI 10.1164/rccm.201702-0433CI

van Griethuysen JJM, 2017, CANCER RES, V77, pE104, DOI 10.1158/0008-5472.CAN-17-0339

Videtic GMM, 2017, PRACT RADIAT ONCOL, V7, P295, DOI 10.1016/j.prro.2017.04.014

Xi IL, 2020, CLIN CANCER RES, V26, P1944, DOI 10.1158/1078-0432.CCR-19-0374

Xu YW, 2019, CLIN CANCER RES, V25, P3266, DOI 10.1158/1078-0432.CCR-18-2495

Zhou B, 2016, PROC CVPR IEEE, P2921, DOI 10.1109/CVPR.2016.319

NR 44

TC 0

Z9 0

U1 4

U2 4

PU ELSEVIER SCIENCE INC

PI NEW YORK

PA STE 800, 230 PARK AVE, NEW YORK, NY 10169 USA

SN 0360-3016

EI 1879-355X

J9 INT J RADIAT ONCOL

JI Int. J. Radiat. Oncol. Biol. Phys.

PD MAR 15

PY 2022

VL 112

IS 4

BP 1045

EP 1054

DI 10.1016/j.ijrobp.2021.11.006

PG 10

WC Oncology; Radiology, Nuclear Medicine & Medical Imaging

WE Science Citation Index Expanded (SCI-EXPANDED)

SC Oncology; Radiology, Nuclear Medicine & Medical Imaging

GA ZG5QP

UT WOS:000760312800026

PM 34775000

DA 2022-08-24

ER

PT J

AU Schildkraut, JS

Prosser, N

Savakis, A

Gomez, J

Nazareth, D

Singh, AK

Malhotra, HK

AF Schildkraut, J. S.

Prosser, N.

Savakis, A.

Gomez, J.

Nazareth, D.

Singh, A. K.

Malhotra, H. K.

TI Level-set segmentation of pulmonary nodules in megavolt electronic

portal images using a CT prior

SO MEDICAL PHYSICS

LA English

DT Article

DE level-set segmentation; electronic portal imaging device; pulmonary

nodule; computed tomography; radiation oncology; computer-aided

detection; digitally reconstructed radiograph; graphical processing unit

ID GUIDED RADIATION-THERAPY; TUMOR-TRACKING; RADIOTHERAPY; MARKERS; SYSTEM

AB Purpose: Pulmonary nodules present unique problems during radiation treatment due to nodule position uncertainty that is caused by respiration. The radiation field has to be enlarged to account for nodule motion during treatment. The purpose of this work is to provide a method of locating a pulmonary nodule in a megavolt portal image that can be used to reduce the internal target volume (ITV) during radiation therapy. A reduction in the ITV would result in a decrease in radiation toxicity to healthy tissue.

Methods: Eight patients with nonsmall cell lung cancer were used in this study. CT scans that include the pulmonary nodule were captured with a GE Healthcare LightSpeed RT 16 scanner. Megavolt portal images were acquired with a Varian Trilogy unit equipped with an AS1000 electronic portal imaging device. The nodule localization method uses grayscale morphological filtering and level-set segmentation with a prior. The treatment-time portion of the algorithm is implemented on a graphical processing unit.

Results: The method was retrospectively tested on eight cases that include a total of 151 megavolt portal image frames. The method reduced the nodule position uncertainty by an average of 40% for seven out of the eight cases. The treatment phase portion of the method has a subsecond execution time that makes it suitable for near-real-time nodule localization.

Conclusions: A method was developed to localize a pulmonary nodule in a megavolt portal image. The method uses the characteristics of the nodule in a prior CT scan to enhance the nodule in the portal image and to identify the nodule region by level-set segmentation. In a retrospective study, the method reduced the nodule position uncertainty by an average of 40% for seven out of the eight cases studied. (C) 2010 American Association of Physicists in Medicine. [DOI: 10.1118/1.3495538]

C1 [Schildkraut, J. S.] Carestream Hlth Inc, Rochester, NY 14615 USA.

[Prosser, N.; Savakis, A.] Rochester Inst Technol, Rochester, NY 14623 USA.

[Gomez, J.; Nazareth, D.; Singh, A. K.; Malhotra, H. K.] Roswell Pk Canc Ctr, Buffalo, NY 14263 USA.

RP Schildkraut, JS (通讯作者)，Carestream Hlth Inc, Rochester, NY 14615 USA.

EM jay.schildkraut@carestreamhealth.com

CR Gierga DP, 2005, INT J RADIAT ONCOL, V61, P1551, DOI 10.1016/j.ijrobp.2004.12.013

Harada T, 2002, CANCER, V95, P1720, DOI 10.1002/cncr.10856

Letourneau D, 2005, RADIOTHER ONCOL, V75, P279, DOI 10.1016/j.radonc.2005.03.001

Lin T, 2009, PHYS MED BIOL, V54, P981, DOI 10.1088/0031-9155/54/4/011

Liu HS, 2003, MED PHYS, V30, P103, DOI 10.1118/1.1533748

PLUEMPITIWIRIYA.C, 2004, P IEEE INT S BIOIMAG

SCHILDKRAUT JS, 2009, P SPIE MED IM C ORL

SCHILDKRAUT JS, 2009, P AAPM 51 ANN M AN C

SCHILDKRAUT JS, 2008, P SCI C STER BOD RAD

Shirato H, 2000, INT J RADIAT ONCOL, V48, P1187, DOI 10.1016/S0360-3016(00)00748-3

SUSSMAN M, 1994, J COMPUT PHYS, V114, P146, DOI 10.1006/jcph.1994.1155

van Ginneken B, 2001, IEEE T MED IMAGING, V20, P1228, DOI 10.1109/42.974918

Vigneault E, 1997, INT J RADIAT ONCOL, V37, P205, DOI 10.1016/S0360-3016(96)00341-0

Xing L, 2006, MED DOSIM, V31, P91, DOI 10.1016/j.meddos.2005.12.004

NR 14

TC 5

Z9 5

U1 0

U2 6

PU AMER ASSOC PHYSICISTS MEDICINE AMER INST PHYSICS

PI MELVILLE

PA STE 1 NO 1, 2 HUNTINGTON QUADRANGLE, MELVILLE, NY 11747-4502 USA

SN 0094-2405

J9 MED PHYS

JI Med. Phys.

PD NOV

PY 2010

VL 37

IS 11

BP 5703

EP 5710

DI 10.1118/1.3495538

PG 8

WC Radiology, Nuclear Medicine & Medical Imaging

WE Science Citation Index Expanded (SCI-EXPANDED)

SC Radiology, Nuclear Medicine & Medical Imaging

GA 674MA

UT WOS:000283747600016

PM 21158282

DA 2022-08-24

ER

PT J

AU Deist, TM

Dankers, FJWM

Valdes, G

Wijsman, R

Hsu, IC

Oberije, C

Lustberg, T

van Soest, J

Hoebers, F

Jochems, A

El Naqa, I

Wee, L

Morin, O

Raleigh, DR

Bots, W

Kaanders, JH

Belderbos, J

Kwint, M

Solberg, T

Monshouwer, R

Bussink, J

Dekker, A

Lambin, P

AF Deist, Timo M.

Dankers, Frank J. W. M.

Valdes, Gilmer

Wijsman, Robin

Hsu, I-Chow

Oberije, Cary

Lustberg, Tim

van Soest, Johan

Hoebers, Frank

Jochems, Arthur

El Naqa, Issam

Wee, Leonard

Morin, Olivier

Raleigh, David R.

Bots, Wouter

Kaanders, Johannes H.

Belderbos, Jose

Kwint, Margriet

Solberg, Timothy

Monshouwer, Rene

Bussink, Johan

Dekker, Andre

Lambin, Philippe

TI Machine learning algorithms for outcome prediction in

(chemo)radiotherapy: An empirical comparison of classifiers

SO MEDICAL PHYSICS

LA English

DT Article

DE classification; machine learning; outcome prediction; predictive

modeling; radiotherapy

ID CELL LUNG-CANCER; MODULATED RADIATION-THERAPY; ACUTE ESOPHAGEAL

TOXICITY; SURVIVAL PREDICTION; RADIOTHERAPY; MODEL; VALIDATION;

BIOMARKERS

AB PurposeMachine learning classification algorithms (classifiers) for prediction of treatment response are becoming more popular in radiotherapy literature. General Machine learning literature provides evidence in favor of some classifier families (random forest, support vector machine, gradient boosting) in terms of classification performance. The purpose of this study is to compare such classifiers specifically for (chemo)radiotherapy datasets and to estimate their average discriminative performance for radiation treatment outcome prediction.

MethodsWe collected 12 datasets (3496 patients) from prior studies on post-(chemo)radiotherapy toxicity, survival, or tumor control with clinical, dosimetric, or blood biomarker features from multiple institutions and for different tumor sites, that is, (non-)small-cell lung cancer, head and neck cancer, and meningioma. Six common classification algorithms with built-in feature selection (decision tree, random forest, neural network, support vector machine, elastic net logistic regression, LogitBoost) were applied on each dataset using the popular open-source R package caret. The R code and documentation for the analysis are available online (). All classifiers were run on each dataset in a 100-repeated nested fivefold cross-validation with hyperparameter tuning. Performance metrics (AUC, calibration slope and intercept, accuracy, Cohen's kappa, and Brier score) were computed. We ranked classifiers by AUC to determine which classifier is likely to also perform well in future studies. We simulated the benefit for potential investigators to select a certain classifier for a new dataset based on our study (pre-selection based on other datasets) or estimating the best classifier for a dataset (set-specific selection based on information from the new dataset) compared with uninformed classifier selection (random selection).

ResultsRandom forest (best in 6/12 datasets) and elastic net logistic regression (best in 4/12 datasets) showed the overall best discrimination, but there was no single best classifier across datasets. Both classifiers had a median AUC rank of 2. Preselection and set-specific selection yielded a significant average AUC improvement of 0.02 and 0.02 over random selection with an average AUC rank improvement of 0.42 and 0.66, respectively.

ConclusionRandom forest and elastic net logistic regression yield higher discriminative performance in (chemo)radiotherapy outcome and toxicity prediction than other studied classifiers. Thus, one of these two classifiers should be the first choice for investigators when building classification models or to benchmark one's own modeling results against. Our results also show that an informed preselection of classifiers based on existing datasets can improve discrimination over random selection.

C1 [Deist, Timo M.; Jochems, Arthur; Lambin, Philippe] Maastricht Univ, Med Ctr, Sch Oncol & Dev Biol, D Lab Decis Support Precis Med,GROW, Univ Singel 40, NL-6229 ER Maastricht, Netherlands.

[Deist, Timo M.; Dankers, Frank J. W. M.; Oberije, Cary; Jochems, Arthur] Maastricht Univ, Med Ctr, Sch Oncol & Dev Biol, Dept Radiat Oncol,GROW, Maastricht, Netherlands.

[Dankers, Frank J. W. M.; Wijsman, Robin; Bots, Wouter; Kaanders, Johannes H.; Monshouwer, Rene; Bussink, Johan] Radboud Univ Nijmegen, Med Ctr, Dept Radiat Oncol, Nijmegen, Netherlands.

[Valdes, Gilmer; Hsu, I-Chow; Morin, Olivier; Raleigh, David R.; Solberg, Timothy] Univ Calif San Francisco, Dept Radiat Oncol, San Francisco, CA USA.

[Lustberg, Tim; van Soest, Johan; Hoebers, Frank; Dekker, Andre] Maastricht Univ, Med Ctr, Sch Oncol & Dev Biol, Dept Radiat Oncol MAASTRO,GROW, Maastricht, Netherlands.

[El Naqa, Issam] Univ Michigan, Dept Radiat Oncol, Ann Arbor, MI 48109 USA.

[Belderbos, Jose; Kwint, Margriet] Antoni van Leeuwenhoek Hosp, Netherlands Canc Inst, Dept Radiat Oncol, Amsterdam, Netherlands.

[Bots, Wouter] Inst Hyperbar Oxygen IvHG, Arnhem, Netherlands.

RP Deist, TM (通讯作者)，Maastricht Univ, Med Ctr, Sch Oncol & Dev Biol, D Lab Decis Support Precis Med,GROW, Univ Singel 40, NL-6229 ER Maastricht, Netherlands.; Deist, TM (通讯作者)，Maastricht Univ, Med Ctr, Sch Oncol & Dev Biol, Dept Radiat Oncol,GROW, Maastricht, Netherlands.

EM t.deist@maastrichtuniversity.nl

RI Naqa, Issam El/T-3066-2019; Monshouwer, R./L-4527-2015; Dekker,

Andre/AAE-4830-2019; Wee, Leonard/AAH-3548-2019; Oberije,

Cary/ABA-6178-2020; Dankers, Frank/M-6658-2015; Bussink, Jan/N-3584-2014

OI Naqa, Issam El/0000-0001-6023-1132; Dekker, Andre/0000-0002-0422-7996;

Wee, Leonard/0000-0003-1612-9055; Oberije, Cary/0000-0003-0749-5117; ,

Timothy/0000-0001-8829-7774; Hoebers, Frank/0000-0002-4317-9181;

Bussink, Johan/0000-0002-5751-4796; van Soest,

Johan/0000-0003-2548-0330; Lambin, Philippe/0000-0001-7961-0191

FU ERC [694812 - Hypoximmuno]; QuIC-ConCePT project; Innovative Medicine

Initiative Joint Undertaking (IMI JU) [115151]; Dutch Technology

Foundation STW [10696 DuCAT, P14-19 Radiomics STRaTegy]; Technology

Programme of the Ministry of Economic Affairs; EU 7th Framework Program

(ARTFORCE) [257144]; EU 7th Framework Program (REQUITE) [601826]; SME

Phase 2 (RAIL) [673780]; EURO-STARS (SeDI); European Program H2020

(BD2Decide) [PHC30-689715]; European Program H2020 (Immuno-SABR)

[733008]; European Program H2020 (PREDICT - ITN) [766276]; European

Program H2020 (CLEARLY) [TRANSCAN-FP-045]; Interreg V-A Euregio

Meuse-Rhine ("Euradiomics"); Kankeronderzoekfonds Limburg from the

Health Foundation Limburg; Alpe d'HuZes-KWF (DESIGN); Zuyderland-MAASTRO

grant; Dutch Cancer Society; KWF-TraIT2HealthRI; Province

Limburg-LIME-Personal Health Train; NFU-Data4LifeSciences; Varian

Medical Systems-SAGE ROO; EURO-STARS (CloudAtlas); EURO-STARS (DART);

EURO-STARS (DECIDE); NATIONAL CANCER INSTITUTE [P01CA059827] Funding

Source: NIH RePORTER

FX Authors acknowledge financial support from ERC advanced grant

(ERC-ADG-2015, no. 694812 - Hypoximmuno) and the QuIC-ConCePT project,

which is partly funded by EFPIa companies and the Innovative Medicine

Initiative Joint Undertaking (IMI JU) under grant agreement no. 115151.

This research is also supported by the Dutch Technology Foundation STW

(grant no. 10696 DuCAT & no. P14-19 Radiomics STRaTegy), which is the

applied science division of NWO, and the Technology Programme of the

Ministry of Economic Affairs. Authors also acknowledge financial support

from the EU 7th Framework Program (ARTFORCE - no. 257144, REQUITE - no.

601826), SME Phase 2 (RAIL - no. 673780), EURO-STARS (SeDI, CloudAtlas,

DART, DECIDE), the European Program H2020 (BD2Decide - PHC30-689715,

Immuno-SABR - no. 733008, PREDICT - ITN - no. 766276, CLEARLY -

TRANSCAN-FP-045), Interreg V-A Euregio Meuse-Rhine ("Euradiomics"),

Kankeronderzoekfonds Limburg from the Health Foundation Limburg, Alpe

d'HuZes-KWF (DESIGN), the Zuyderland-MAASTRO grant and the Dutch Cancer

Society, KWF-TraIT2HealthRI, Province Limburg-LIME-Personal Health

Train, NFU-Data4LifeSciences, Varian Medical Systems-SAGE & ROO.

CR Belderbos J, 2005, RADIOTHER ONCOL, V75, P157, DOI 10.1016/j.radonc.2005.03.021

Bots WTC, 2017, HEAD NECK-J SCI SPEC, V39, P1122, DOI 10.1002/hed.24733

BREIMAN L, 2001, MACH LEARN, V0045

Caruana R, 2015, KDD'15: PROCEEDINGS OF THE 21ST ACM SIGKDD INTERNATIONAL CONFERENCE ON KNOWLEDGE DISCOVERY AND DATA MINING, P1721, DOI 10.1145/2783258.2788613

Carvalho S, 2016, DATA PROGNOSTIC VALU, DOI [10. 17195/candat. 2016. 04. 1, DOI 10.17195/CANDAT.2016.04.1]

Carvalho S, 2016, RADIOTHER ONCOL, V119, P487, DOI 10.1016/j.radonc.2016.04.024

Deist TM, CODE MACHINE LEARNIN

Egelmeer AGTM, 2011, RADIOTHER ONCOL, V100, P108, DOI 10.1016/j.radonc.2011.06.023

Fernandez-Delgado M, 2014, J MACH LEARN RES, V15, P3133

Friedman J, 2010, J STAT SOFTW, V33, P1, DOI 10.18637/jss.v033.i01

Hastie T., 2009, ELEMENTS STAT LEARNI, V2nd ed.

James G., 2013, INTRO STAT LEARNING, V112

Janssens GO, 2012, J CLIN ONCOL, V30, P1777, DOI 10.1200/JCO.2011.35.9315

Jochems A, 2017, INT J RADIAT ONCOL, V99, P344, DOI 10.1016/j.ijrobp.2017.04.021

Karatzoglou A., 2004, J STAT SOFTW, V11, P1, DOI [10.18637/jss.v011.i09, 10.18637/jss. v011.i09, DOI 10.18637/JSS.V011.I09]

Kuhn M., 2016, CARET CLASSIFICATION

Kwint M, 2012, INT J RADIAT ONCOL, V84, pE223, DOI 10.1016/j.ijrobp.2012.03.027

Lambin P, 2013, RADIOTHER ONCOL, V109, P159, DOI 10.1016/j.radonc.2013.07.007

Lambin P, 2013, NAT REV CLIN ONCOL, V10, P27, DOI 10.1038/nrclinonc.2012.196

Lavesson N., 2006, P 21 NAT C ART INT B, P395

Lustberg T, 2016, ONCOTARGET, V7, P37288, DOI 10.18632/oncotarget.8755

Oberije C, 2015, DATA VALIDATED PREDI, DOI [10. 5072/candat. 2015. 02, DOI 10.5072/CANDAT.2015.02]

Oberije C, 2015, INT J RADIAT ONCOL, V92, P935, DOI 10.1016/j.ijrobp.2015.02.048

Olling Karina, 2018, Tech Innov Patient Support Radiat Oncol, V5, P16, DOI 10.1016/j.tipsro.2018.01.002

Olson RS, 2017, BIOCOMPUTING 2018, P192, DOI [10. 1142/9789813235533_0018, DOI 10.1142/9789813235533_0018]

Parmar C, 2015, SCI REP-UK, V5, DOI 10.1038/srep13087

Steyerberg EW, 2010, EPIDEMIOLOGY, V21, P128, DOI 10.1097/EDE.0b013e3181c30fb2

Therneau T, 2017, RPART RECURSIVE PART

Tuszynski J, 2014, CATOOLS TOOLS MOVING

Valdes G, 2016, SCI REP-UK, V6, DOI 10.1038/srep37854

Venables WN, 2002, MODERN APPL STAT S, DOI DOI 10.1007/978-0-387-21706-2

Wainer J, 2016, ARXIV160600930

Wijsman R, 2017, INT J RADIAT ONCOL, V99, P434, DOI 10.1016/j.ijrobp.2017.04.011

Wijsman R, 2015, RADIOTHER ONCOL, V117, P49, DOI 10.1016/j.radonc.2015.08.010

NR 34

TC 119

Z9 120

U1 5

U2 25

PU WILEY

PI HOBOKEN

PA 111 RIVER ST, HOBOKEN 07030-5774, NJ USA

SN 0094-2405

EI 2473-4209

J9 MED PHYS

JI Med. Phys.

PD JUL

PY 2018

VL 45

IS 7

BP 3449

EP 3459

DI 10.1002/mp.12967

PG 11

WC Radiology, Nuclear Medicine & Medical Imaging

WE Science Citation Index Expanded (SCI-EXPANDED)

SC Radiology, Nuclear Medicine & Medical Imaging

GA GM5VJ

UT WOS:000438211400059

PM 29763967

OA Green Published, hybrid, Green Accepted

DA 2022-08-24

ER

PT J

AU Barabino, E

Rossi, G

Pamparino, S

Fiannacca, M

Caprioli, S

Fedeli, A

Zullo, L

Vagge, S

Cittadini, G

Genova, C

AF Barabino, Emanuele

Rossi, Giovanni

Pamparino, Silvia

Fiannacca, Martina

Caprioli, Simone

Fedeli, Alessandro

Zullo, Lodovica

Vagge, Stefano

Cittadini, Giuseppe

Genova, Carlo

TI Exploring Response to Immunotherapy in Non-Small Cell Lung Cancer Using

Delta-Radiomics

SO CANCERS

LA English

DT Article

DE radiomics; NSCLC; delta-radiomics; immunotherapy; antiPD1; immune

checkpoint inhibitor; predictive value

ID CRITERIA

AB Simple Summary The aim of the study is to identify radiomic features capable of predicting the response to immunotherapy. Delta-radiomics can foresees the comparison between subsequent CT scans and therefore allows to predict the changes that occurred during the treatment. In this study, the individual lesions of patients with advanced non-small cell lung cancer treated with immunotherapy were analyzed. The study aims to discover the features that predict the response to immune checkpoint inhibitors. Delta-radiomics is a branch of radiomics in which features are confronted after time or after introducing an external factor (such as treatment with chemotherapy or radiotherapy) to extrapolate prognostic data or to monitor a certain condition. Immune checkpoint inhibitors (ICIs) are currently revolutionizing the treatment of non-small cell lung cancer (NSCLC); however, there are still many issues in defining the response to therapy. Contrast-enhanced CT scans of 33 NSCLC patients treated with ICIs were analyzed; altogether, 43 lung lesions were considered. The radiomic features of the lung lesions were extracted from CT scans at baseline and at first reassessment, and their variation (delta, Delta) was calculated by means of the absolute difference and relative reduction. This variation was related to the final response of each lesion to evaluate the predictive ability of the variation itself. Twenty-seven delta features have been identified that are able to discriminate radiologic response to ICIs with statistically significant accuracy. Furthermore, the variation of nine features significantly correlates with pseudo-progression.

C1 [Barabino, Emanuele] Osped Santa Corona ASL 2 Savonese, Intervent Angiog, I-17027 Pietra Ligure, Italy.

[Rossi, Giovanni; Zullo, Lodovica] IRCCS Osped Policlin San Martino, UOC Oncol Med 2, I-16132 Genoa, Italy.

[Rossi, Giovanni] Univ Sassari, Dept Med Surg & Expt Sci, I-07100 Sassari, Italy.

[Pamparino, Silvia; Fiannacca, Martina; Caprioli, Simone] Univ Genoa, Osped Policlin San Martino, Dept Hlth Sci DISSAL, I-16128 Genoa, Italy.

[Fedeli, Alessandro] Univ Genoa, Dept Elect Elect Telecommun Engn & Naval Architec, I-16145 Genoa, Italy.

[Vagge, Stefano] IRCCS Osped Policlin San Martino, Dept Radiat Oncol, I-16132 Genoa, Italy.

[Cittadini, Giuseppe] IRCCS Osped Policlin San Martino, UO Radiol Gen, I-16132 Genoa, Italy.

[Genova, Carlo] IRCCS Osped Policlin San Martino, UOC Clin Oncol Med, I-16132 Genoa, Italy.

[Genova, Carlo] Univ Genoa, Fac Med & Chirurg, Dipartimento Med Interna & Specialita Med DiMi, I-16132 Genoa, Italy.

RP Rossi, G (通讯作者)，IRCCS Osped Policlin San Martino, UOC Oncol Med 2, I-16132 Genoa, Italy.; Rossi, G (通讯作者)，Univ Sassari, Dept Med Surg & Expt Sci, I-07100 Sassari, Italy.

EM emanuele.barabino@gmail.com; giovanni.rossi.1689@gmail.com;

pamparinosilvia@gmail.com; fiannacca.martina@tiscali.it;

simone.caprioli11@gmail.com; alessandro.fedeli@unige.it;

lodozullo@gmail.com; stefano.vagge@hsanmartino.it;

giuseppe.cittadini@hsanmartino.it; carlo.genova@hsanmartino.it

RI Genova, Carlo/J-8931-2016

OI Genova, Carlo/0000-0003-3690-8582; Caprioli, Simone/0000-0002-6017-9968;

Barabino, Emanuele/0000-0001-6407-5878; rossi,

giovanni/0000-0001-8432-5408; Pamparino, Silvia/0000-0002-5193-4490;

Zullo, Lodovica/0000-0002-1659-788X; vagge, stefano/0000-0002-8212-7069

FU Italian Ministry of Health; Bristol Myers Squibb [CA209-828]

FX Funding from the Italian Ministry of Health (5 x 1000 funds; Ricerca

Corrente 2018-2020) and Bristol Myers Squibb (CA209-828) were received

for this research.

CR Dercle L, 2020, CLIN CANCER RES, V26, P2151, DOI 10.1158/1078-0432.CCR-19-2942

Doroshow DB, 2019, CLIN CANCER RES, V25, P4592, DOI 10.1158/1078-0432.CCR-18-1538

Fave X, 2017, SCI REP-UK, V7, DOI 10.1038/s41598-017-00665-z

Fedorov A, 2012, MAGN RESON IMAGING, V30, P1323, DOI 10.1016/j.mri.2012.05.001

Flavell RR, 2020, INT J RADIAT ONCOL, V108, P242, DOI 10.1016/j.ijrobp.2020.06.025

Khorrami M, 2020, CANCER IMMUNOL RES, V8, P108, DOI 10.1158/2326-6066.CIR-19-0476

Kim Hae-Young, 2014, Restor Dent Endod, V39, P74, DOI 10.5395/rde.2014.39.1.74

Lambin P, 2012, EUR J CANCER, V48, P441, DOI 10.1016/j.ejca.2011.11.036

Liu Y, 2021, FRONT ONCOL, V11, DOI 10.3389/fonc.2021.657615

Lu S, 2019, JAMA ONCOL, V5, P1195, DOI 10.1001/jamaoncol.2019.1549

Ma YQ, 2020, FRONT ONCOL, V10, DOI 10.3389/fonc.2020.01017

Matzner-Lober E, 2018, DIAGN INTERV IMAG, V99, P269, DOI 10.1016/j.diii.2018.04.011

Miar A, 2020, CANCER RES, V80, P5245, DOI 10.1158/0008-5472.CAN-19-2306

Nardone V, 2020, MED ONCOL, V37, DOI 10.1007/s12032-020-01359-9

Nasief H, 2019, NPJ PRECIS ONCOL, V3, DOI 10.1038/s41698-019-0096-z

Plautz TE, 2019, MED PHYS, V46, P1663, DOI 10.1002/mp.13395

Ribas A, 2009, CLIN CANCER RES, V15, P7116, DOI 10.1158/1078-0432.CCR-09-2376

Rossi G, 2021, CANCER RES, V81, P724, DOI 10.1158/0008-5472.CAN-20-0999

Selno ATH, 2020, AGING-US, V12, P23478, DOI 10.18632/aging.202343

Seymour L, 2017, LANCET ONCOL, V18, pE143, DOI 10.1016/S1470-2045(17)30074-8

Tazdait M, 2018, EUR J CANCER, V88, P38, DOI 10.1016/j.ejca.2017.10.017

Testa U, 2018, CANCERS, V10, DOI 10.3390/cancers10080248

Tomaszewski MR, 2021, RADIOLOGY, V298, P505, DOI [10.1148/radiol.2021202553, 10.1148/radiol.2021219005]

van Griethuysen JJM, 2017, CANCER RES, V77, pE104, DOI 10.1158/0008-5472.CAN-17-0339

Vargha A, 1998, J EDUC BEHAV STAT, V23, P170, DOI 10.3102/10769986023002170

NR 25

TC 2

Z9 2

U1 3

U2 3

PU MDPI

PI BASEL

PA ST ALBAN-ANLAGE 66, CH-4052 BASEL, SWITZERLAND

EI 2072-6694

J9 CANCERS

JI Cancers

PD JAN

PY 2022

VL 14

IS 2

AR 350

DI 10.3390/cancers14020350

PG 15

WC Oncology

WE Science Citation Index Expanded (SCI-EXPANDED)

SC Oncology

GA YN9AD

UT WOS:000747542300001

PM 35053513

OA Green Published, gold

DA 2022-08-24

ER

PT J

AU Chan, ST

Ruan, D

Shaverdian, N

Raghavan, G

Cao, M

Lee, P

AF Chan, Shawna T.

Ruan, Dan

Shaverdian, Narek

Raghavan, Govind

Cao, Minsong

Lee, Percy

TI Effect of Radiation Doses to the Heart on Survival for Stereotactic

Ablative Radiotherapy for Early-stage Non-Small-cell Lung Cancer: An

Artificial Neural Network Approach

SO CLINICAL LUNG CANCER

LA English

DT Article

DE Cardiac substructure dosimetry; Deep learning; Early-stage lung cancer;

Stereotactic body radiotherapy; Survivorship

ID CARDIOVASCULAR-DISEASE; CARDIAC TOXICITY; ESCALATION TRIALS;

BREAST-CANCER; LARGE COHORT; LONG-TERM; THERAPY; CONCURRENT; MORTALITY;

LYMPHOMA

AB The effect of the cardiac radiation dose in patients with early-stage non-small-cell lung cancer (NSCLC) undergoing stereotactic ablative radiotherapy is incompletely understood. In the present retrospective analysis of 112 adults, an increased cardiac radiation dose was associated with decreased survival in those with NSCLC. Radiation-induced cardiac toxicity could contribute to morbidity and mortality in those with early-stage NSCLC. Thus, efforts to minimize the cardiac radiation dose and cardiac follow-up protocols should be explored.

Introduction: The cardiac radiation dose is an important predictor of cardiac toxicity and overall survival (OS) for patients with locally advanced non-small-cell lung cancer (NSCLC). However, radiation-induced cardiac toxicity among patients with early-stage NSCLC who have undergone stereotactic ablative radiotherapy (SABR) has been less well-characterized. Our objective was to assess the associations between cardiac radiation dosimetry and OS in patients with early-stage NSCLC undergoing SABR. Materials and Methods: From 2009 to 2014, 153 patients with early-stage NSCLC had undergone SABR at a single institution. The maximum dose, mean dose, V-10Gy, V-25Gy, and V-50Gy to 15 cardiac substructures and the whole heart were analyzed for their association with OS using the Kaplan-Meier method. An artificial neural network (ANN) analysis was performed to modulate confounding behaviors of dosimetric variables to predict for OS. Results: A total of 112 patients were included in the present analysis. The right ventricle (RV) V-10Gy most negatively predicted for OS, such that patients who had received a RV V-10Gy dose < 4% had significantly longer OS than patients who had received a RV V-10Gy does > 4% (5.3 years vs. 2.4 years). On ANN analysis, 74 input features, including cardiac dosimetry parameters, predicted for survival with a test accuracy of 64.7%. A repeat ANN analysis using dosimetry to dose neutral structure confirmed the predictive power of cardiac dosimetry. Conclusion: Cardiac dosimetry to subvolumes of the heart was associated with decreased OS in patients with early-stage NSCLC undergoing SABR. These data support the importance of minimizing the radiation dose to cardiac substructures. Further prioritizing the heart as an organ at risk might be warranted. Additionally, cardiac follow-up should be considered.

C1 [Chan, Shawna T.; Ruan, Dan; Shaverdian, Narek; Raghavan, Govind; Cao, Minsong; Lee, Percy] Univ Calif Los Angeles, Sch Med, Dept Radiat Oncol, 200 Ucla Med Plaza,B265, Los Angeles, CA 90095 USA.

[Chan, Shawna T.] Univ Calif Irvine, Sch Med, Irvine, CA 92717 USA.

[Shaverdian, Narek] Mem Sloan Kettering Canc Ctr, Dept Radiat Oncol, 1275 York Ave, New York, NY 10021 USA.

[Lee, Percy] Univ Calif Los Angeles, Jonsson Comprehens Canc Ctr, Los Angeles, CA 90024 USA.

[Lee, Percy] Univ Texas MD Anderson Canc Ctr, Dept Radiat Oncol, Houston, TX 77030 USA.

RP Lee, P (通讯作者)，Univ Calif Los Angeles, Sch Med, Dept Radiat Oncol, 200 Ucla Med Plaza,B265, Los Angeles, CA 90095 USA.

EM percylee@mednet.ucla.edu

OI Chan, Shawna/0000-0003-0066-079X

CR Abe O, 2005, LANCET, V366, P2087

Belliere A, 2009, CANCER RADIOTHER, V13, P298, DOI 10.1016/j.canrad.2009.04.004

Bradley JD, 2015, LANCET ONCOL, V16, P187, DOI 10.1016/S1470-2045(14)71207-0

Carver JR, 2007, J CLIN ONCOL, V25, P3991, DOI 10.1200/JCO.2007.10.9777

Chang JY, 2014, INT J RADIAT ONCOL, V88, P1120, DOI 10.1016/j.ijrobp.2014.01.022

Chin KM, 2005, CORONARY ARTERY DIS, V16, P13, DOI 10.1097/00019501-200502000-00003

Contreras JA, 2018, RADIOTHER ONCOL, V128, P498, DOI 10.1016/j.radonc.2018.05.017

Darby SC, 2013, NEW ENGL J MED, V368, P987, DOI 10.1056/NEJMoa1209825

Darby SC, 2010, INT J RADIAT ONCOL, V76, P656, DOI 10.1016/j.ijrobp.2009.09.064

Dess RT, 2017, J CLIN ONCOL, V35, P1395, DOI 10.1200/JCO.2016.71.6142

Evans JD, 2013, RADIOTHER ONCOL, V109, P82, DOI 10.1016/j.radonc.2013.07.021

GYENES G, 1994, INT J RADIAT ONCOL, V28, P1235, DOI 10.1016/0360-3016(94)90500-2

Hardy D, 2010, ANN ONCOL, V21, P1825, DOI 10.1093/annonc/mdq042

Hatakenaka M, 2012, INT J RADIAT ONCOL, V83, pE67, DOI 10.1016/j.ijrobp.2011.12.018

Holmes JA, 2017, JNCI CANCER SPECT, V1, DOI 10.1093/jncics/pkx003

Hooning MJ, 2007, JNCI-J NATL CANCER I, V99, P365, DOI 10.1093/jnci/djk064

Jones GC, 2015, CLIN LUNG CANCER, V16, P413, DOI 10.1016/j.cllc.2015.04.001

Kong FM, 2011, 1106ACRIN6697 RTOG

Kong FM, ATLASES ORGANS RISK

MACKAY DJC, 1992, NEURAL COMPUT, V4, P415, DOI 10.1162/neco.1992.4.3.415

Maraldo MV, 2015, LANCET HAEMATOL, V2, pE492, DOI 10.1016/S2352-3026(15)00153-2

Marks LB, 2005, INT J RADIAT ONCOL, V63, P214, DOI 10.1016/j.ijrobp.2005.01.029

McLaughlin VV, 2005, EUR RESPIR J, V25, P244, DOI 10.1183/09031936.05.00054804

MENDES LA, 1994, AM HEART J, V128, P301, DOI 10.1016/0002-8703(94)90483-9

Mieth B, 2016, SCI REP-UK, V6, DOI 10.1038/srep36671

Milano MT, 2009, RADIOTHER ONCOL, V91, P301, DOI 10.1016/j.radonc.2009.03.005

Modh A, 2014, INT J RADIAT ONCOL, V90, P1168, DOI 10.1016/j.ijrobp.2014.08.008

Nellessen U, 2010, CHEMOTHERAPY, V56, P147, DOI 10.1159/000313528

Ning MS, 2017, INT J RADIAT ONCOL, V99, P70, DOI 10.1016/j.ijrobp.2017.05.022

Raghavan G, 2018, CLIN LUNG CANCER, V19, pE759, DOI 10.1016/j.cllc.2018.05.008

Reshko LB, 2018, J THORAC DIS, V10, P2346, DOI 10.21037/jtd.2018.04.42

Rygiel K, 2017, J CANCER RES THER, V13, P186, DOI 10.4103/0973-1482.187303

Shirvani SM, 2014, JAMA SURG, V149, P1244, DOI 10.1001/jamasurg.2014.556

Stam B, 2017, RADIOTHER ONCOL, V123, P370, DOI 10.1016/j.radonc.2017.04.017

Stewart Merrill H, 2017, Curr Treat Options Cardiovasc Med, V19, P53, DOI 10.1007/s11936-017-0550-6

Tembhekar AR, 2017, CLIN LUNG CANCER, V18, P293, DOI 10.1016/j.cllc.2016.12.007

Timmerman R, 2006, ACTA ONCOL, V45, P779, DOI 10.1080/02841860600902213

Timmerman R, 2010, JAMA-J AM MED ASSOC, V303, P1070, DOI 10.1001/jama.2010.261

Tukenova M, 2010, J CLIN ONCOL, V28, P1308, DOI 10.1200/JCO.2008.20.2267

van Nimwegen FA, 2015, JAMA INTERN MED, V175, P1007, DOI 10.1001/jamainternmed.2015.1180

Verma V, 2018, RADIOTHER ONCOL, V128, P492, DOI 10.1016/j.radonc.2018.06.011

Videtic GMM, 2017, PRACT RADIAT ONCOL, V7, P295, DOI 10.1016/j.prro.2017.04.014

Vivekanandan S, 2017, INT J RADIAT ONCOL, V99, P51, DOI 10.1016/j.ijrobp.2017.04.026

Voelkel NF, 2006, CIRCULATION, V114, P1883, DOI 10.1161/CIRCULATIONAHA.106.632208

Wang K, 2017, RADIOTHER ONCOL, V125, P293, DOI 10.1016/j.radonc.2017.10.001

Wang K, 2017, J CLIN ONCOL, V35, P1387, DOI 10.1200/JCO.2016.70.0229

Wen SW, 2019, J INVEST SURG, V32, P27, DOI 10.1080/08941939.2017.1370519

Yu JB, 2015, CANCER-AM CANCER SOC, V121, P2341, DOI 10.1002/cncr.29359

NR 48

TC 5

Z9 5

U1 0

U2 5

PU CIG MEDIA GROUP, LP

PI DALLAS

PA 3500 MAPLE AVENUE, STE 750, DALLAS, TX 75219-3931 USA

SN 1525-7304

EI 1938-0690

J9 CLIN LUNG CANCER

JI Clin. Lung Cancer

PD MAR

PY 2020

VL 21

IS 2

BP 136

EP +

DI 10.1016/j.cllc.2019.10.010

PG 10

WC Oncology

WE Science Citation Index Expanded (SCI-EXPANDED)

SC Oncology

GA KS7GD

UT WOS:000518474100017

PM 31932217

DA 2022-08-24

ER

PT J

AU Tseng, HH

Luo, Y

Cui, S

Chien, JT

Ten Haken, RK

El Naqa, I

AF Tseng, Huan-Hsin

Luo, Yi

Cui, Sunan

Chien, Jen-Tzung

Ten Haken, Randall K.

El Naqa, Issam

TI Deep reinforcement learning for automated radiation adaptation in lung

cancer

SO MEDICAL PHYSICS

LA English

DT Article

DE adaptive radiotherapy; deep learning; lung cancer; reinforcement

learning

ID BREAST; TISSUE; RADIOTHERAPY; IRRADIATION; RESPONSES; MODEL

AB Purpose: To investigate deep reinforcement learning (DRL) based on historical treatment plans for developing automated radiation adaptation protocols for nonsmall cell lung cancer (NSCLC) patients that aim to maximize tumor local control at reduced rates of radiation pneumonitis grade 2 (RP2).

Methods: In a retrospective population of 114 NSCLC patients who received radiotherapy, a three-component neural networks framework was developed for deep reinforcement learning (DRL) of dose fractionation adaptation. Large-scale patient characteristics included clinical, genetic, and imaging radiomics features in addition to tumor and lung dosimetric variables. First, a generative adversarial network (GAN) was employed to learn patient population characteristics necessary for DRL training from a relatively limited sample size. Second, a radiotherapy artificial environment (RAE) was reconstructed by a deep neural network (DNN) utilizing both original and synthetic data (by GAN) to estimate the transition probabilities for adaptation of personalized radiotherapy patients' treatment courses. Third, a deep Q-network (DQN) was applied to the RAE for choosing the optimal dose in a response-adapted treatment setting. This multicomponent reinforcement learning approach was benchmarked against real clinical decisions that were applied in an adaptive dose escalation clinical protocol. In which, 34 patients were treated based on avid PET signal in the tumor and constrained by a 17.2% normal tissue complication probability (NTCP) limit for RP2. The uncomplicated cure probability (P+) was used as a baseline reward function in the DRL.

Results: Taking our adaptive dose escalation protocol as a blueprint for the proposed DRL (GAN + RAE + DQN) architecture, we obtained an automated dose adaptation estimate for use at similar to 2/3 of the way into the radiotherapy treatment course. By letting the DQN component freely control the estimated adaptive dose per fraction (ranging from 1-5 Gy), the DRL automatically favored dose escalation/de-escalation between 1.5 and 3.8 Gy, a range similar to that used in the clinical protocol. The same DQN yielded two patterns of dose escalation for the 34 test patients, but with different reward variants. First, using the baseline P+ reward function, individual adaptive fraction doses of the DQN had similar tendencies to the clinical data with an RMSE = 0.76 Gy; but adaptations suggested by the DQN were generally lower in magnitude (less aggressive). Second, by adjusting the P+ reward function with higher emphasis on mitigating local failure, better matching of doses between the DQN and the clinical protocol was achieved with an RMSE = 0.5 Gy. Moreover, the decisions selected by the DQN seemed to have better concordance with patients eventual outcomes. In comparison, the traditional temporal difference (TD) algorithm for reinforcement learning yielded an RMSE = 3.3 Gy due to numerical instabilities and lack of sufficient learning.

Conclusion: We demonstrated that automated dose adaptation by DRL is a feasible and a promising approach for achieving similar results to those chosen by clinicians. The process may require customization of the reward function if individual cases were to be considered. However, development of this framework into a fully credible autonomous system for clinical decision support would require further validation on larger multi-institutional datasets. (C) 2017 American Association of Physicists in Medicine.

C1 [Tseng, Huan-Hsin; Luo, Yi; Cui, Sunan; Chien, Jen-Tzung; Ten Haken, Randall K.; El Naqa, Issam] Univ Michigan, Dept Radiat Oncol, Ann Arbor, MI 48109 USA.

[Chien, Jen-Tzung] Natl Chiao Tung Univ, Dept Elect & Comp Engn, Hsinchu, Taiwan.

RP El Naqa, I (通讯作者)，Univ Michigan, Dept Radiat Oncol, Ann Arbor, MI 48109 USA.

EM ielnaqa@med.umich.edu

RI cui, sunan/AAA-3286-2020; Naqa, Issam El/T-3066-2019

OI cui, sunan/0000-0002-8846-9449; Naqa, Issam El/0000-0001-6023-1132; Luo,

Yi/0000-0003-2519-5900; Chien, Jen-Tzung/0000-0003-3466-8941

FU National Institutes of Health [P01 CA059827]; NATIONAL CANCER INSTITUTE

[P01CA059827] Funding Source: NIH RePORTER

FX The authors thank Dr. Kong and Dr. Jolly for their help in providing the

lung testing datasets for the study and Julia Pakela for the careful

proofreading of our manuscript. This work was supported in part by the

National Institutes of Health P01 CA059827.

CR Abadi M., 2016, ARXIV 160304467

AGREN A, 1990, INT J RADIAT ONCOL, V19, P1077, DOI 10.1016/0360-3016(90)90037-K

Bellemare MG, 2013, J ARTIF INTELL RES, V47, P253, DOI 10.1613/jair.3912

Bentzen SM, 2000, ACTA ONCOL, V39, P337, DOI 10.1080/028418600750013113

Bradley JD, 2015, LANCET ONCOL, V16, P187, DOI 10.1016/S1470-2045(14)71207-0

Brockman G., 2016, OPENAI GYM

Clevert D.-A., ARXIV151107289

Dalmis MU, 2017, MED PHYS, V44, P533, DOI 10.1002/mp.12079

Deshmane SL, 2009, J INTERF CYTOK RES, V29, P313, DOI 10.1089/jir.2008.0027

Eisbruch A, USING FDG PET ACQUIR

El Naqa I, 2017, PHYS MED BIOL, V62, pR179, DOI 10.1088/1361-6560/aa7c55

El Naqa I, 2014, CLIN TRANSL IMAGING, V2, P305, DOI 10.1007/s40336-014-0063-1

Falou O, 2013, TRANSL ONCOL, V6, P17, DOI 10.1593/tlo.12412

Galloway M., 1975, COMPUT VISION GRAPH, V4, P172, DOI [10.1016/S0146-664X(75)80008-6, DOI 10.1016/S0146-664X(75)80008-6]

Goh V, 2011, RADIOLOGY, V261, P165, DOI 10.1148/radiol.11110264

Goller C, 1996, IEEE IJCNN, P347, DOI 10.1109/ICNN.1996.548916

Goodfellow IJ, 2014, ADV NEUR IN, V27, P2672

HORNIK K, 1989, NEURAL NETWORKS, V2, P359, DOI 10.1016/0893-6080(89)90020-8

Ibragimov B, 2017, MED PHYS, V44, P547, DOI 10.1002/mp.12045

Jaffray DA, 2012, NAT REV CLIN ONCOL, V9, P688, DOI 10.1038/nrclinonc.2012.194

Kim M, 2009, PHYS MED BIOL, V54, P4455, DOI 10.1088/0031-9155/54/14/007

Kingma D, ARXIV1412698

Kong FM, 2017, JAMA ONCOL, V3, P1358, DOI 10.1001/jamaoncol.2017.0982

KUTCHER GJ, 1989, INT J RADIAT ONCOL, V16, P1623, DOI 10.1016/0360-3016(89)90972-3

Laffey JG, 2002, ANESTHESIOLOGY, V97, P215

LeCun Y, 2015, NATURE, V521, P436, DOI 10.1038/nature14539

Letterio JJ, 1998, ANNU REV IMMUNOL, V16, P137, DOI 10.1146/annurev.immunol.16.1.137

Lowry R., 2014, CONCEPTS APPL INFERE

Luo Y, 2017, RADIOTHER ONCOL, V123, P85, DOI 10.1016/j.radonc.2017.02.004

Mnih V., 2013, P ADV NEUR INF PROC, P1

Mnih V, 2015, NATURE, V518, P529, DOI 10.1038/nature14236

Mohri M., 2012, FDN MACHINE LEARNING

Naqa IEl, THEORY APPL

Ng A.Y., 2000, P 17 INT C MACH LEAR, P663, DOI DOI 10.2460/AJVR.67.2.323

Nguyen DHT, 2013, P NATL ACAD SCI USA, V110, P6712, DOI 10.1073/pnas.1221526110

Oh JH, 2011, PHYS MED BIOL, V56, P1635, DOI 10.1088/0031-9155/56/6/008

Ramirez MF, 2013, ANESTHESIOLOGY, V3, P133, DOI DOI 10.4236/0JANES.2013.33031

Sadeghi-Naini A, 2015, MED PHYS, V42, P6130, DOI 10.1118/1.4931603

Sadeghi-Naini A, 2013, MED PHYS, V40, DOI 10.1118/1.4812683

Sadeghi-Naini A, 2013, CLIN CANCER RES, V19, P2163, DOI 10.1158/1078-0432.CCR-12-2965

Schaue D, 2012, RADIAT RES, V178, P505, DOI 10.1667/RR3031.1

Sovik A, 2007, PHYS MEDICA, V23, P100, DOI 10.1016/j.ejmp.2007.09.001

Srivastava N, 2014, J MACH LEARN RES, V15, P1929

Sutton R.S., 1998, REINFORCEMENT LEARNI, V1

Tsitsiklis JN, 1997, ADV NEUR IN, V9, P1075

Vallieres M, 2015, PHYS MED BIOL, V60, P5471, DOI 10.1088/0031-9155/60/14/5471

Vapnik V., 1999, NATURE STAT LEARNING

Vincent RD, 2016, ASA SIAM SER STAT AP, P263

Witney TH, 2010, BRIT J CANCER, V103, P1400, DOI 10.1038/sj.bjc.6605945

NR 49

TC 78

Z9 84

U1 6

U2 52

PU WILEY

PI HOBOKEN

PA 111 RIVER ST, HOBOKEN 07030-5774, NJ USA

SN 0094-2405

EI 2473-4209

J9 MED PHYS

JI Med. Phys.

PD DEC

PY 2017

VL 44

IS 12

BP 6690

EP 6705

DI 10.1002/mp.12625

PG 16

WC Radiology, Nuclear Medicine & Medical Imaging

WE Science Citation Index Expanded (SCI-EXPANDED)

SC Radiology, Nuclear Medicine & Medical Imaging

GA FW5SQ

UT WOS:000425379200055

PM 29034482

OA Green Published, Green Accepted

DA 2022-08-24

ER

PT J

AU Zhou, ZG

Folkert, M

Cannon, N

Iyengar, P

Westover, K

Zhang, YY

Choy, H

Timmerman, R

Yan, JS

Xie, XJ

Jiang, S

Wang, J

AF Zhou, Zhiguo

Folkert, Michael

Cannon, Nathan

Iyengar, Puneeth

Westover, Kenneth

Zhang, Yuanyuan

Choy, Hak

Timmerman, Robert

Yan, Jingsheng

Xie, Xian-J.

Jiang, Steve

Wang, Jing

TI Predicting distant failure in early stage NSCLC treated with SBRT using

clinical parameters

SO RADIOTHERAPY AND ONCOLOGY

LA English

DT Article

DE Distant failure; SBRT; Clinical parameter; Machine learning; Feature

selection

ID CELL LUNG-CANCER; STEREOTACTIC BODY RADIOTHERAPY; RADIATION-THERAPY;

CLONAL SELECTION; CLASSIFICATION; PET

AB Purpose/objective: The aim of this study is to predict early distant failure in early stage non-small cell lung cancer (NSCLC) treated with stereotactic body radiation therapy (SBRT) using clinical parameters by machine learning algorithms.

Materials/methods: The dataset used in this work includes 81 early stage NSCLC patients with at least 6 months of follow-up who underwent SBRT between 2006 and 2012 at a single institution. The clinical parameters (n = 18) for each patient include demographic parameters, tumor characteristics, treatment fraction schemes, and pretreatment medications. Three predictive models were constructed based on different machine learning algorithms: (1) artificial neural network (ANN), (2) logistic regression (LR) and (3) support vector machine (SVM). Furthermore, to select an optimal clinical parameter set for the model construction, three strategies were adopted: (1) clonal selection algorithm (CSA) based selection strategy; (2) sequential forward selection (SFS) method; and (3) statistical analysis (SA) based strategy. 5-cross validation is used to validate the performance of each predictive model. The accuracy was assessed by area under the receiver operating characteristic (ROC) curve (AUC), sensitivity and specificity of the system was also evaluated.

Results: The AUCs for ANN, LR and SVM were 0.75, 0.73, and 0.80, respectively. The sensitivity values for ANN, LR and SVM were 71.2%, 72.9% and 83.1%, while the specificity values for ANN, LR and SVM were 59.1%, 63.6% and 63.6%, respectively. Meanwhile, the CSA based strategy outperformed SFS and SA in terms of AUC, sensitivity and specificity.

Conclusions: Based on clinical parameters, the SVM with the CSA optimal parameter set selection strategy achieves better performance than other strategies for predicting distant failure in lung SBRT patients. (C) 2016 Elsevier Ireland Ltd. All rights reserved.

C1 [Zhou, Zhiguo; Folkert, Michael; Cannon, Nathan; Iyengar, Puneeth; Westover, Kenneth; Zhang, Yuanyuan; Choy, Hak; Timmerman, Robert; Jiang, Steve; Wang, Jing] UT Southwestern Med Ctr, Dept Radiat Oncol, Dallas, TX 75390 USA.

[Yan, Jingsheng; Xie, Xian-J.] UT Southwestern Med Ctr, Dept Clin Sci, Dallas, TX USA.

RP Wang, J (通讯作者)，UT Southwestern Med Ctr, Dept Radiat Oncol, Dallas, TX 75390 USA.

EM jing.wang@utsouthwestern.edu

RI Westover, Ken/AAZ-1795-2020; Wang, Jing/N-7332-2019

OI Westover, Ken/0000-0003-3653-5923; Zhang, Yuanyuan/0000-0001-9230-7314;

Wang, Jing/0000-0002-8491-4146

FU Cancer Prevention and Research Institute of Texas [RP130109]; American

Cancer Society [RSG-13-326-01-CCE, ACS-IRG-02-196]; US National Health

Institute [R01 EB020366]; NATIONAL INSTITUTE OF BIOMEDICAL IMAGING AND

BIOENGINEERING [R01EB020366] Funding Source: NIH RePORTER

FX The authors acknowledge funding support from the Cancer Prevention and

Research Institute of Texas (RP130109), the American Cancer Society

(RSG-13-326-01-CCE and ACS-IRG-02-196) and US National Health Institute

(R01 EB020366). The authors would like to thank Dr. Damiana Chiavolini

for editing the manuscript.

CR Aberle DR, 2011, NEW ENGL J MED, V365, P395, DOI 10.1056/NEJMoa1102873

Breslow N E, 1980, IARC Sci Publ, P5

Chang CC, 2011, ACM T INTEL SYST TEC, V2, DOI 10.1145/1961189.1961199

Chawla NV, 2002, J ARTIF INTELL RES, V16, P321, DOI 10.1613/jair.953

Chi A, 2010, RADIOTHER ONCOL, V94, P1, DOI 10.1016/j.radonc.2009.12.008

Clarke K, 2012, RADIOTHER ONCOL, V104, P62, DOI 10.1016/j.radonc.2012.04.019

CORTES C, 1995, MACH LEARN, V20, P273, DOI 10.1023/A:1022627411411

de Castro LN, 2002, IEEE T EVOLUT COMPUT, V6, P239, DOI 10.1109/TEVC.2002.1011539

Ettinger DS, 2015, J NATL COMPR CANC NE, V13, P515, DOI 10.6004/jnccn.2015.0071

Freedman D., 2009, STAT MODELS THEORY P

Hoyer M, 2008, RADIOTHER ONCOL, V87, P1, DOI 10.1016/j.radonc.2008.03.004

Jain A, 1997, IEEE T PATTERN ANAL, V19, P153, DOI 10.1109/34.574797

Kazmierska J, 2008, RADIOTHER ONCOL, V86, P211, DOI 10.1016/j.radonc.2007.10.019

Khan J, 2001, NAT MED, V7, P673, DOI 10.1038/89044

Lambin P, 2013, RADIOTHER ONCOL, V109, P159, DOI 10.1016/j.radonc.2013.07.007

Liu HW, 2015, RADIOTHER ONCOL, V117, P71, DOI 10.1016/j.radonc.2015.08.027

Postmus PE, 2007, J THORAC ONCOL, V2, P686, DOI 10.1097/JTO.0b013e31811f4703

ROCHESTER N, 1956, IRE T INFORM THEOR, V2, P80, DOI 10.1109/TIT.1956.1056810

Roelofs E, 2014, RADIOTHER ONCOL, V110, P370, DOI 10.1016/j.radonc.2013.11.001

Senthi S, 2012, LANCET ONCOL, V13, P802, DOI 10.1016/S1470-2045(12)70242-5

Timmerman RD, 2014, INT J RADIAT ONCOL, V90, pS30, DOI 10.1016/j.ijrobp.2014.05.135

Timmerman R, 2010, JAMA-J AM MED ASSOC, V303, P1070, DOI 10.1001/jama.2010.261

van Baardwijk A, 2012, RADIOTHER ONCOL, V105, P145, DOI 10.1016/j.radonc.2012.09.008

van Stiphout RGPM, 2011, RADIOTHER ONCOL, V98, P126, DOI 10.1016/j.radonc.2010.12.002

Zhang H, 2014, INT J RADIAT ONCOL, V88, P195, DOI 10.1016/j.ijrobp.2013.09.037

Zhang L, 2007, IEEE T GEOSCI REMOTE, V45, P4172, DOI 10.1109/TGRS.2007.905311

Zhang X, 2011, EURASIP J WIREL COMM, DOI 10.1155/2011/765143

Zhou ZG, 2013, BMC MED INFORM DECIS, V13, DOI 10.1186/1472-6947-13-123

NR 28

TC 30

Z9 30

U1 0

U2 6

PU ELSEVIER IRELAND LTD

PI CLARE

PA ELSEVIER HOUSE, BROOKVALE PLAZA, EAST PARK SHANNON, CO, CLARE, 00000,

IRELAND

SN 0167-8140

EI 1879-0887

J9 RADIOTHER ONCOL

JI Radiother. Oncol.

PD JUN

PY 2016

VL 119

IS 3

BP 501

EP 504

DI 10.1016/j.radonc.2016.04.029

PG 4

WC Oncology; Radiology, Nuclear Medicine & Medical Imaging

WE Science Citation Index Expanded (SCI-EXPANDED)

SC Oncology; Radiology, Nuclear Medicine & Medical Imaging

GA DR7JH

UT WOS:000380075400020

PM 27156652

OA Green Accepted

DA 2022-08-24

ER

PT J

AU Yu, W

Tang, C

Hobbs, BP

Li, X

Koay, EJ

Wistuba, II

Sepesi, B

Behrens, C

Canales, JR

Cuentas, ERP

Erasmus, JJ

Court, LE

Chang, JY

AF Yu, Wen

Tang, Chad

Hobbs, Brian P.

Li, Xiao

Koay, Eugene J.

Wistuba, Ignacio I.

Sepesi, Boris

Behrens, Carmen

Canales, Jaime Rodriguez

Cuentas, Edwin Roger Parra

Erasmus, Jeremy J.

Court, Laurence E.

Chang, Joe Y.

TI Development and Validation of a Predictive Radiomics Model for Clinical

Outcomes in Stage I Non-small Cell Lung Cancer

SO INTERNATIONAL JOURNAL OF RADIATION ONCOLOGY BIOLOGY PHYSICS

LA English

DT Article

ID STEREOTACTIC ABLATIVE RADIOTHERAPY; BODY RADIATION-THERAPY; LOCAL

RADIATION; FEATURES; CT; RECURRENCE; MELANOMA; TUMOR

AB Purpose: To develop and validate a radiomics signature that can predict the clinical outcomes for patients with stage I non-small cell lung cancer (NSCLC).

Methods and Materials: We retrospectively analyzed contrast-enhanced computed tomography images of patients from a training cohort (n = 147) treated with surgery and an independent validation cohort (n = 295) treated with stereotactic ablative radiation therapy. Twelve radiomics features with established strategies for filtering and preprocessing were extracted. The random survival forests (RSF) method was used to build models from subsets of the 12 candidate features based on their survival relevance and generate a mortality risk index for each observation in the training set. An optimal model was selected, and its ability to predict clinical outcomes was evaluated in the validation set using predicted mortality risk indexes.

Results: The optimal RSF model, consisting of 2 predictive features, kurtosis and the gray level co-occurrence matrix feature homogeneity2, allowed for significant risk stratification (log-rank P <. 0001) and remained an independent predictor of overall survival after adjusting for age, tumor volume and histologic type, and Karnofsky performance status (hazard ratio [HR] 1.27; P < 2e-16) in the training set. The resultant mortality risk indexes were significantly associated with overall survival in the validation set (log-rank P = .0173; HR 1.02, P = .0438). They were also significant for distant metastasis (log-rank P <. 05; HR 1.04, P = .0407) and were borderline significant for regional recurrence on univariate analysis (log-rank P < .05; HR 1.04, P = .0617).

Conclusions: Our radiomics model accurately predicted several clinical outcomes and allowed pretreatment risk stratification in stage I NSCLC, allowing the choice of treatment to be tailored to each patient's individual risk profile. (C) 2017 Elsevier Inc. All rights reserved.

C1 [Yu, Wen; Tang, Chad; Koay, Eugene J.; Chang, Joe Y.] Univ Texas MD Anderson Canc Ctr, Dept Radiat Oncol, Unit 1422,1400 Pressler St, Houston, TX 77030 USA.

[Hobbs, Brian P.; Li, Xiao] Univ Texas MD Anderson Canc Ctr, Dept Biostat, Houston, TX 77030 USA.

[Wistuba, Ignacio I.; Canales, Jaime Rodriguez; Cuentas, Edwin Roger Parra] Univ Texas MD Anderson Canc Ctr, Dept Translat & Mol Pathol, Houston, TX 77030 USA.

[Sepesi, Boris] Univ Texas MD Anderson Canc Ctr, Dept Thorac & Cardiovasc Surg, Houston, TX 77030 USA.

[Behrens, Carmen] Univ Texas MD Anderson Canc Ctr, Dept Thorac Head & Neck Med Oncol, Houston, TX 77030 USA.

[Erasmus, Jeremy J.] Univ Texas MD Anderson Canc Ctr, Dept Diagnost Radiol, Houston, TX 77030 USA.

[Court, Laurence E.] Univ Texas MD Anderson Canc Ctr, Dept Radiat Phys, 1515 Holcombe Blvd, Houston, TX 77030 USA.

[Yu, Wen] Shanghai Jiao Tong Univ, Shanghai Chest Hosp, Dept Radiat Oncol, Shanghai, Peoples R China.

RP Chang, JY (通讯作者)，Univ Texas MD Anderson Canc Ctr, Dept Radiat Oncol, Unit 1422,1400 Pressler St, Houston, TX 77030 USA.; Court, LE (通讯作者)，Univ Texas MD Anderson Canc Ctr, Dept Radiat Phys, 1515 Holcombe Blvd, Houston, TX 77030 USA.

EM LECourt@mdanderson.org; jychang@mdanderson.org

OI Court, Laurence/0000-0002-3241-6145

FU National Natural Science Foundation of China [81502645]; Western

Medicine Guiding Program - Science and Technology Commission of Shanghai

Municipality [14411968800]; Cancer Center Support (Core) grant from the

National Cancer Institute [CA016672]; NATIONAL CANCER INSTITUTE

[P30CA016672] Funding Source: NIH RePORTER

FX W.Y. was supported by the National Natural Science Foundation of China

(grant 81502645) and Western Medicine Guiding Program funded by the

Science and Technology Commission of Shanghai Municipality (grant

14411968800).; The present study was supported in part by Cancer Center

Support (Core) grant CA016672 from the National Cancer Institute to The

University of Texas MD Anderson Cancer Center.

CR Aerts HJWL, 2014, NAT COMMUN, V5, DOI 10.1038/ncomms5006

Ambler G, 2012, STAT MED, V31, P1150, DOI 10.1002/sim.4371

Bernstein MB, 2016, NAT REV CLIN ONCOL, V13, P516, DOI 10.1038/nrclinonc.2016.30

Chang JY, 2015, J THORAC ONCOL, V10, P577, DOI 10.1097/JTO.0000000000000453

Chang JY, 2012, RADIAT ONCOL, V7, DOI 10.1186/1748-717X-7-152

Chen X, 2012, GENOMICS, V99, P323, DOI 10.1016/j.ygeno.2012.04.003

Fave X, 2016, TRANSL CANCER RES, V5, P349, DOI 10.21037/tcr.2016.07.11

Fried DV, 2014, INT J RADIAT ONCOL, V90, P834, DOI 10.1016/j.ijrobp.2014.07.020

Gillies RJ, 2016, RADIOLOGY, V278, P563, DOI 10.1148/radiol.2015151169

HARALICK RM, 1973, IEEE T SYST MAN CYB, VSMC3, P610, DOI 10.1109/TSMC.1973.4309314

Hiniker SM, 2012, TRANSL ONCOL, V5, P404, DOI 10.1593/tlo.12280

Huynh E, 2016, RADIOTHER ONCOL, V120, P258, DOI 10.1016/j.radonc.2016.05.024

Ishwaran H, 2008, ANN APPL STAT, V2, P841, DOI 10.1214/08-AOAS169

Kamiya A, 2014, JPN J RADIOL, V32, P14, DOI 10.1007/s11604-013-0264-y

Lee YJ, 2009, BLOOD, V114, P589, DOI 10.1182/blood-2009-02-206870

Lugade AA, 2005, J IMMUNOL, V174, P7516, DOI 10.4049/jimmunol.174.12.7516

Mattonen SA, 2016, INT J RADIAT ONCOL, V94, P1121, DOI 10.1016/j.ijrobp.2015.12.369

Mattonen SA, 2015, J MED IMAGING, V2, DOI 10.1117/1.JMI.2.4.041010

Paul D, 2017, COMPUT MED IMAG GRAP, V60, P42, DOI 10.1016/j.compmedimag.2016.12.002

Postow MA, 2012, NEW ENGL J MED, V366, P925, DOI 10.1056/NEJMoa1112824

Schreibmann E, 2016, MED PHYS, V43, P3374, DOI 10.1118/1.4955779

Senthi S, 2012, LANCET ONCOL, V13, P802, DOI 10.1016/S1470-2045(12)70242-5

Singh VP, 2016, INT J COMPUTER SCI I, V14, P82

Taylor JMG, 2011, J THORAC ONCOL, V6, P1974, DOI 10.1097/JTO.0b013e318233d835

Timmerman R, 2010, JAMA-J AM MED ASSOC, V303, P1070, DOI 10.1001/jama.2010.261

Wang XH, 2017, CANCER RES, V77, P839, DOI 10.1158/0008-5472.CAN-15-3142

Yang ZG, 2001, AM J ROENTGENOL, V176, P1399, DOI 10.2214/ajr.176.6.1761399

Zhang Lifei, 2015, Med Phys, V42, P1341, DOI 10.1118/1.4908210

Zhang YC, 2017, SCI REP-UK, V7, DOI 10.1038/srep46349

Zhou Z, 2016, MED PHYS, V43, P3383, DOI 10.1118/1.4955817

NR 30

TC 31

Z9 32

U1 2

U2 21

PU ELSEVIER SCIENCE INC

PI NEW YORK

PA STE 800, 230 PARK AVE, NEW YORK, NY 10169 USA

SN 0360-3016

EI 1879-355X

J9 INT J RADIAT ONCOL
[truncated: 1,026,250 more chars]
